# Fluorescence lifetime multiplexing with fluorogen activating protein FAST variants

**DOI:** 10.1038/s42003-024-06501-1

**Published:** 2024-07-02

**Authors:** Yulia A. Bogdanova, Ilya D. Solovyev, Nadezhda S. Baleeva, Ivan N. Myasnyanko, Anastasia A. Gorshkova, Dmitriy A. Gorbachev, Aidar R. Gilvanov, Sergey A. Goncharuk, Marina V. Goncharuk, Konstantin S. Mineev, Alexander S. Arseniev, Alexey M. Bogdanov, Alexander P. Savitsky, Mikhail S. Baranov

**Affiliations:** 1 Institute of Bioorganic Chemistry, Russian Academy of Sciences, Miklukho-Maklaya 16/10, 117997 Moscow, Russia; 2 A.N. Bach Institute of Biochemistry, Research Center of Biotechnology of the Russian Academy of Sciences, 119071 Moscow, Russia; 3 Pirogov Russian National Research Medical University, Ostrovitianov 1, Moscow, 117997 Russia; 4 Department of Photonics, İzmir Institute of Technology, 35430 İzmir, Turkey; 5 Department of Biology, Lomonosov Moscow State University, Moscow, 119991 Russia, 121205 Moscow, Russia; 6 Present Address: Goethe University Frankfurt, Frankfurt am Main, 60433 Germany

**Keywords:** Fluorescence imaging, Fluorescent proteins

## Abstract

In this paper, we propose a fluorescence-lifetime imaging microscopy (FLIM) multiplexing system based on the fluorogen-activating protein FAST. This genetically encoded fluorescent labeling platform employs FAST mutants that activate the same fluorogen but provide different fluorescence lifetimes for each specific protein-dye pair. All the proposed probes with varying lifetimes possess nearly identical and the smallest-in-class size, along with quite similar steady-state optical properties. In live mammalian cells, we target these chemogenetic tags to two intracellular structures simultaneously, where their fluorescence signals are clearly distinguished by FLIM. Due to the unique structure of certain fluorogens under study, their complexes with FAST mutants display a monophasic fluorescence decay, which may facilitate enhanced multiplexing efficiency by reducing signal cross-talks and providing optimal prerequisites for signal separation upon co-localized and/or spatially overlapped labeling.

## Abstract (web summary)

A genetically encoded labeling system uses smallest-in-class fluorogen-activating protein tags for time-resolved fluorescence multiplexed cellular imaging, offering monoexponential decay and potential for sophisticated fluorescence lifetime analysis.

## Introduction

In modern biology, it is often essential to observe multiple cellular processes simultaneously and in real time. Therefore, various imaging multiplexing systems are needed to achieve higher information density from a single specimen. Conventional multicolor fluorescent labeling certainly provides such an opportunity^1–3^, nevertheless, it has a principal constraint: the width of the visible light spectrum is limited and typically provides the reliable detection of signals from only 3-4 probes without overlap^4^. Fluorescence spectrum imaging and subsequent unmixing overcome the problem of spectral crosstalks^5^, but the method has been rarely used with live cells^6^ as it is computationally challenging.

A completely different and currently booming approach to imaging multiplexing is fluorescence-lifetime imaging microscopy (FLIM), which employs fluorescence lifetimes to differentiate between multiple fluorescent tags excited by the same light wavelength^7,8^. This technique can be utilized as label-free^9^, but additional chromophores diversify its possibilities. Specifically, a recent study demonstrated FLIM multiplexing using various combinations of synthetic probes with affinity for the cell organelles^7^. Although attractive due to its simplicity, this technique provides a rather limited range of targets and moderate specificity of targeting. As of yet, it has only utilized the fluorophores with bi- or triphasic fluorescence decay, whose time-resolved signals are more crosstalk-prone and challenging to unmix, especially in case of their spatial overlap. To address the probe targeting issue, genetically encoded labeling techniques are preferably applied in live cell imaging. GFP-family fluorescent proteins are single-component and fully genetically encoded tags, which are a probe of choice for numerous live-cell applications. However, they are rarely implemented in FLIM multiplexing since spectrally similar fluorescent proteins with comparable brightness usually have close fluorescence lifetimes values, with only a few exceptions^10,11^. One could assume that diverse self-labeling proteins, which along with a wide palette of complementary synthetic chromophores constitute a toolkit for chemogenetically-based imaging^12^, could have a substantial potential in FLIM multiplexing. Indeed, in the recent studies, the Halo-tag^13^ and SNAP-tag-based^7^ self-labeling probes have been implemented in such a manner. Noticeably, potential drawbacks of the Halo-tag system could be anticipated based on its molecular weight, which is even larger than that of fluorescent proteins (33 kDa vs. 27 kDa for GFP). The potential impact of this on cellular physiology and the behavior of the tagged proteins of interest has been documented^14–17^.

**Fig. 1:**
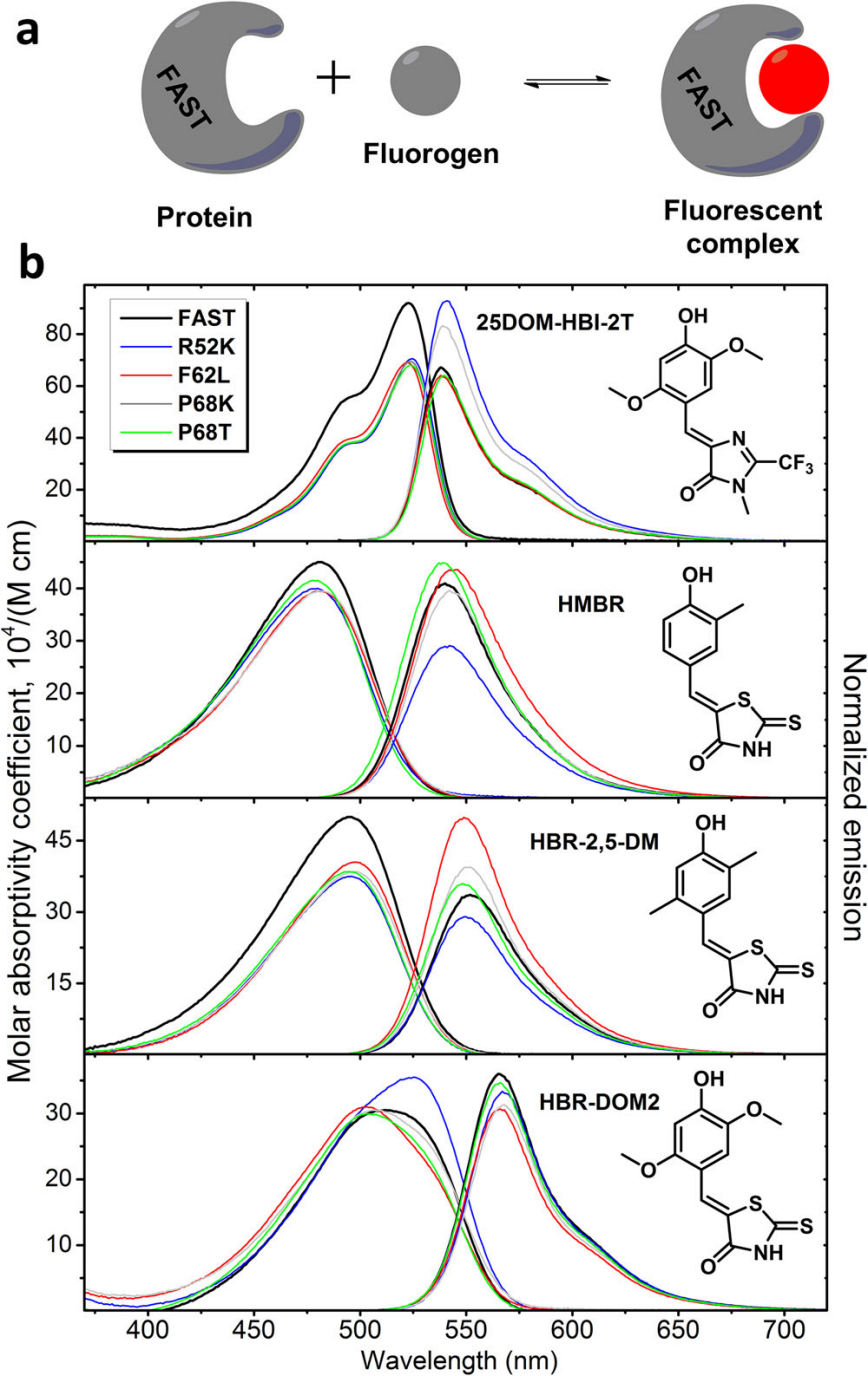
FAST fluorogens used in this study. **a** The general principle of fluorogen-activating protein action. **b** The absorption and emission spectra of various fluorogens in complexes with FAST protein and its mutants presented in the article. The absorption spectra are normalized to the protein/ligand complex concentration and represented in the molar absorptivity coefficient scale. The emission spectra are normalized to the fluorescence quantum yield.

In this regard, the genetically encoded fluorescent labeling platform based on fluorogen-activating proteins, which has recently come into the spotlight, may offer certain advantages^12,18–20^. Similarly to other chemogenetic tags, these proteins do not possess their own chromophore but bind an external fluorogen, which does not fluoresce in solution and becomes emissive only in a complex with a protein (Fig. 1a). Fluorogen-activating proteins are considerably smaller than the aforementioned genetically encoded tags and, due to the external nature and non-covalent binding of the fluorogen, are much more photostable. One of the well-known fluorogen-activating proteins is FAST (Fluorescence-Activating and Absorption-Shifting Tag), which has a molecular weight of only 14 kDa in contrast with 33 kDa and 27 kDa for Halo-tag and GFP, respectively^21^. FAST-based labeling is widely used in conventional fluorescence microscopy^22,23^, including multicolor imaging^24^ and in vivo visualization in the far-red region of the spectrum^25^. However its potential in FLIM has not yet been fully explored. In particular, previously, only in one study and only two variants of the FAST protein (iFAST and greenFAST with **HMBR** fluorogen) were successfully used for simultaneous labeling in FLIM microscopy^24^. Also, Gautier’s team suggested a novel FLIM multiplexing system based on the FAST protein variants in their recent preprint^26^ published during the peer-review process of our study.

In the present paper, we propose a genetically encoded system for multiplexed fluorescence lifetime microscopy based on the FAST protein. After testing diverse combinations of the FAST variants, previously engineered by a structure-guided protein design^27^, and several fluorogen candidates, we identified a set of protein-fluorogen pairs exhibiting well-distinguishable fluorescence lifetimes. These chemogenetic probes, nearly identical in size, color, and other steady-state optical properties, demonstrated effective performance in the mammalian cell expression system. Their fluorescence signals from two simultaneously labeled intracellular targets, including those with partial co-localization, were clearly distinguished by FLIM. With the proposed labeling system, it was also possible to co-visualize triplets of cellular targets, although the signal unmixing lacked reliability in that case.

## Results and Discussion

Investigating the 3D structure of the fluorogen-activating protein FAST and the nature of its interaction with various fluorogens, we created a series of its point mutants and found that even a single amino acid substitution frequently leads to dramatic changes in the properties of resulting complexes^27^. The fluorescence lifetime of chromophores strongly depends on the environment properties^28,29^, thus making the fluorogen-binding pocket (the environment for fluorogen) a perfect ground for the lifetime optimization. Therefore, in this study, we employed FAST protein variants (Supplementary Table S1) to investigate the impact of such substitutions not only on steady-state optical characteristics but also on the fluorescence lifetimes. These mutations were previously selected to occur in the mobile hot spots of FAST^27,30^, therefore it is likely that they could affect fluorogen mobility, relaxation of its excited state, and consequently, the fluorescence lifetime. For this research, we selected four previously proposed fluorogens that bind efficiently to the original FAST protein and have relatively similar spectra ranging from green to orange fluorescence - **HBR-DOM2**^30^, **HMBR**^21^, **HBR-2,5-DM**^21,31^, and **25DOM-HBI-2T**^32^(Fig. 1b).

### Fluorescence lifetime screening in bacteria

Using a custom-made Macro-FLIM device (Supplementary Picture S1), we performed a primary screening of these substances against two dozen variants of the FAST protein proposed earlier (Supplementary Tables S1–5)^27^. For this purpose, bacterial spots expressing the proteins were treated with fluorogen solutions and analyzed using the aforementioned device. Firstly, we identified dim colonies for certain fluorogens and excluded these proteins from consideration - T70V, V105I\V107I, R52E D65R, R52Y, R52F, R52E, R52L, P97T, R52D, P97T\T98G and V105I. Low brightness in such a screening indicates a high K_d_ and/or a low fluorescence quantum yield and/or a low extinction coefficient, which in total will hinder the use of such a pair in labeling. Subsequently, we selected the protein with the longest lifetime for the majority of fluorogens - F62L. Next, we selected variants with typically low lifetimes (P68T and R52K), as well as an intermediate variant - P68K. For different fluorogens, these dependencies could be slightly different, but overall, we were able to select four proteins with different lifetimes for all fluorogens at once.

### Photophysical properties of purified proteins binding various fluorogens

Using isolated proteins (Supplementary Fig. S1), we studied the properties of these protein complexes in more detail, together with the original FAST tag - Fig. 1, Table 1, Supplementary Figs. S2–29.

**Table 1:** Optical properties of **HBR-DOM2,** **HMBR, HBR-2,5-DM** and **25DOM-HBI-2T** in complexes with FAST mutants measured in vitro.^a^

Fluorogen	Variant^b^	K_d_, µM	ε, M^−1^·cm^−1^	FQY,^c^%	Brightness	τ_1_/τ_2_, ns	A_1_/A_2_, %
	FAST^31^	0.008	50000	29	14500	2.48/0.13	95/5
	F62L	0.120	40500	43	17250	2.33/0.16	94/6
	P68K	0.084	38500	34	13150	2.00/0.19	92/8
	P68T	0.090	38500	31	11800	1.75/0.17	91/9
	R52K	0.046	37500	25	9350	1.43/0.15	89/11
	FAST^27^	0.13	45000	31	13900	2.19/0.21	90/10
	F62L	0.25	39500	33	12850	2.05/0.18	93/7
	P68K	0.16	39500	30	11950	1.74/0.42	83/17
	P68T	0.19	41500	34	14200	1.55/0.28	89/11
	R52K	0.13	40000	22	8850	1.36/0.21	89/11
	FAST^30^	0.021	30500	54	16500	3.74/0.43	98/2
	R52K	0.035	35500	50	17700	3.55/0.22	98/2
	P68K	0.030	30500	47	14400	3.53/-	100/-
	P68T	0.036	30000	52	15600	3.30/-	100/-
	F62L	0.036	31000	46	14400	2.88/0.46	97/3
	FAST^32^	0.52	92000	21	19300	2.92/1.70	47/44^d^
	R52K	0.44	70500	29	20350	2.32/0.28	88/12
	F62L	0.97	69000	20	13450	1.98/0.19	84/16
	P68K	0.34	69500	26	18200	1.96/0.21	88/12
	P68T	0.52	68000	20	13350	1.72/0.19	85/15

^a^We represent data here as mean values to facilitate comprehension, full data can be found in the supporting information (Supplementary Tables S6–8, Supplementary Figs. S2–29).

^b^For each fluorogen, proteins are ordered according to the decreasing lifetime of the major component.

^c^Fluorescence quantum yield.

^d^An additional third component with τ = 0.145 ns (9%) was found.

A review of the data reveals that the parent FAST protein provides the longest fluorescence lifetimes for all the ligands. However, the parent protein demonstrates an extremely low dissociation constant for **HBR-2,5-DM** fluorogen. Such differences in affinity may hinder the use of this protein together with its mutants for multiplexing. One of the key features of exchangeable fluorogenic dyes is the high photostability of the label provided by the exchange of free and bound dye^22^. A lower constant complicates this exchange, which will obviously result in a photostability drop and, in turn, affect labeling versatility when multiplexing. Additionally, excessively tight binding will not allow washing the fluorogen off the complex with this protein if it is necessary. Moreover, the drastic difference in dissociation constants between the probes can lead to unequal saturation of individual proteins with fluorogens and, as a consequence, to considerable inconsistencies in signal intensity. Therefore, we excluded the original FAST protein from further consideration and cell experiments. In all other cases, the studied complexes were characterized by fairly similar dissociation constants. The F62L substitution reduced affinity for almost all complexes, probably due to the loss of effective pi-stacking with the benzylidene fragment of fluorogens. It is noteworthy that we did not find a direct correlation between dissociation constants and either fluorescence quantum yields or fluorescence lifetimes. This suggests that it is impossible to predict these parameters from each other, and also that efficient binding cannot guarantee either a high fluorescence quantum yield or long lifetimes. Previously^27–32^, we and other authors have already observed the lack of correlation between K_d_ and fluorescence quantum yield, possibly due the fact that better binding to the protein does not necessarily guarantee rigid conformational fixation of the entire molecule.

Depending on the ligand structure, the four proposed mutations (R52K, P68T, P68K, and F62L) had multidirectional effects on quantum yield and extinction coefficient (Table 1). However, in general, for all four protein variants and four fluorogens, the complexes were characterized by fairly similar brightness.

For almost all protein-fluorogen pairs, we observed biphasic fluorescence decay (Table 1). However, in most cases, the contribution of the second component was not pronounced. A noticeable deviation was observed only for the pair of the parent FAST protein with the fluorogen **25DOM-HBI-2T**, which confirms our decision to exclude this protein from further consideration. Importantly, although only two purely monoexponential protein-fluorogen pairs (P68T-**HBR-DOM2** and P68K-**HBR-DOM2)** were revealed by time-resolved spectroscopy of the purified proteins, 14 out of the 18 remaining pairs, whose fluorescence decays were fitted by a biexponential model, exhibited a minor fluorescent population with very short (100–300 ps) lifetimes. Considering the small relative contributions of these emissive species (less than 10% in 9 out of 18 pairs), one can suggest that at a lower photon count per pixel typical for FLIM (hundreds of photons at the peak vs. thousands of photons in the case of spectroscopy), they will become barely distinguishable, thus resulting in an apparent decay monophasicity. Apparently, the presence of two components may be associated with the flexibility of the chromophore and the presence of separate conformational forms. Among the potential sites of such flexibility, we can identify the exo-C = C bond of the imidazolone or the C-C bond of the arylidene fragments, which we have previously shown for other arylidene-azolones^33^. Obviously, such a change in geometry is difficult for the bulkier **HBR-DOM2** ligand, while compounds **HMBR** and **HBR-2,5-DM** undergo this process more easily.

### Structure-based analysis of fluorescence lifetimes

**Fig. 2:**
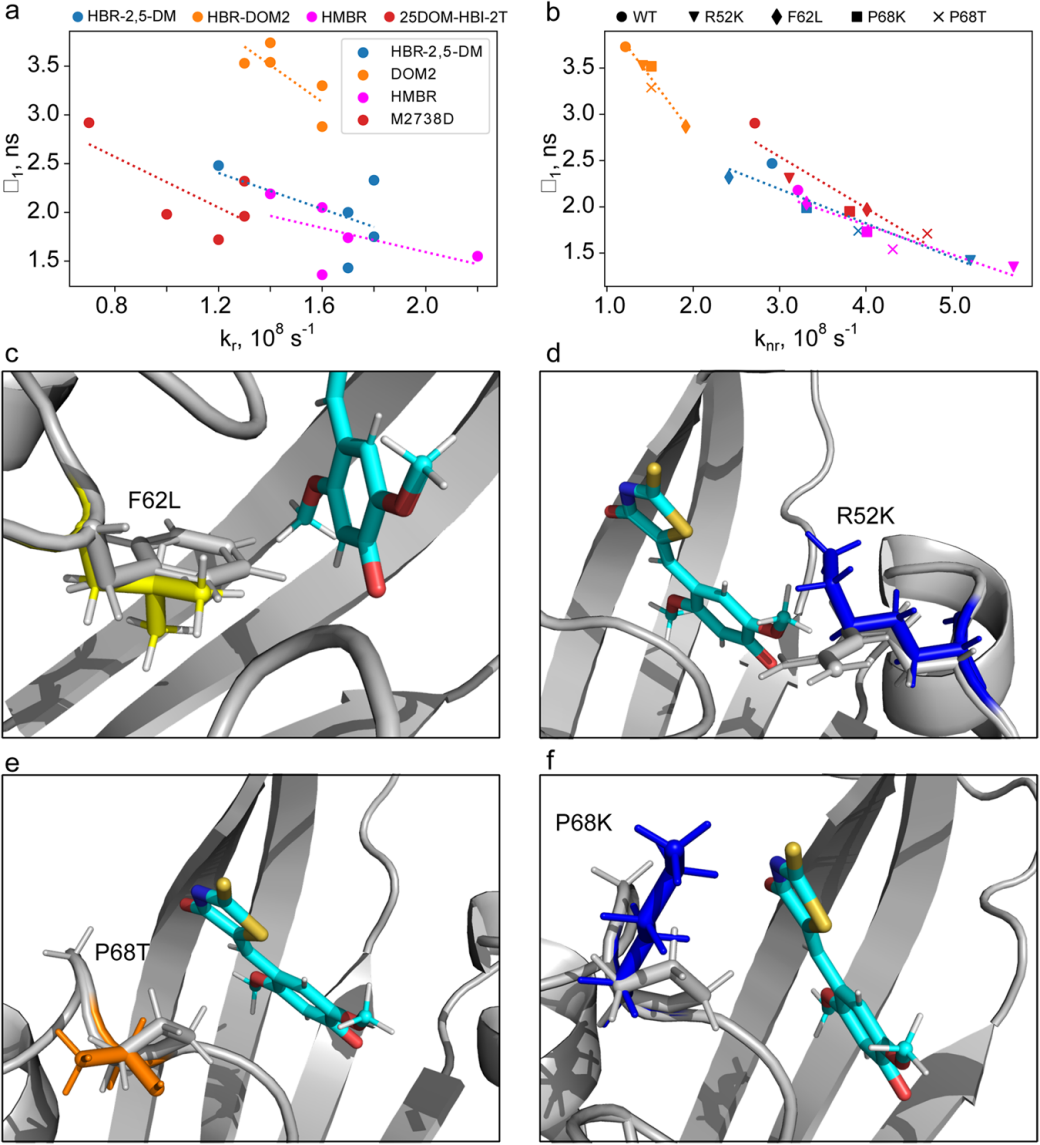
Structural basis of mutation-induced fluorescence lifetimes variation within the FAST variants. **a**, **b** Correlations between the lifetimes of major long-lived exponential components revealed after the measurement of FAST-fluorogens’ fluorescence decays (τ_1_ from Table 1) and the rates of radiative (**a**) and non-radiative (**b**) transitions calculated for individual protein-fluorogen pairs. The data sets include original FAST (circles) and its variants (R52K, triangles; F62L, diamonds; P68K, squares; and P68T, crosses) with four ligands: **HBR-2,5-DM** (blue), **HBR-DOM2** (orange), **HMBR** (magenta), and **25DOM-HBI-2T** (red). Dashed lines represent the correlation curve obtained for each ligand. **c**–**f** PyMOL generated models showing the possible effect of mutations, constructed based on the spatial structure of the nanoFAST/**HBR-DOM2** complex (PDB code A8O0)^35^. The sidechain of the original FAST amino acid is shown in gray, and the result of the mutation is colored. The ligand molecule is shown in cyan.

The fluorescence lifetime depends on two parameters: the rates of radiative and other nonradiative transitions (k_r_ and k_nr_). The first parameter depends mainly on the electronic structure of the fluorogen, while the second one is determined by several factors. However, in the absence of FRET, proton transfer, quenching, and aforementioned isomerization, the primary factor is the dynamics. The rates of transitions could be assessed for a given fluorescence lifetime and quantum yield (see the Analysis of fluorescence lifetimes section in the Methods)^34^, and in the case of our dataset, such an analysis suggests that the effect of FAST mutations on the lifetimes is most likely dominated by k_nr_ and, hence, by the dynamics of the fluorogen and its surroundings within the binding pocket (Fig. 2a, b). Therefore, the fluorescence lifetimes in particular protein/ligand complexes may be analyzed in terms of the possible changes in the local protein mobility introduced by the mutation under consideration. To gain a comprehensive view on the structural consequences of the found mutations, we generated a set of mutants in PyMOL for the available structure of the nanoFAST complex with **HBR-DOM2**^35^.

The F62L mutation affects the lifetimes of two ligands: **HBR-DOM2** and **25DOM-HBI-2T**. Unlike **HMBR** and **HBR-2,5-DM**, these two fluorogens bear the bulky methoxy groups at their benzyl ring and, thereby, occupy a much larger volume inside the pocket. Residue 62 is packed directly against the benzyl ring of the ligands in the available spatial structures of FAST and its derivative nanoFAST^35^. Apparently, Phe at position 62, which is larger than Leu (Fig. 2c), substantially stabilizes the packing of bulky ligands in the binding pocket, while it does not affect the packing of smaller methyl-substituted compounds. Besides, Phe can take part in π-stacking interactions with the ligand’s benzyl ring, and the presence of methoxy groups instead of methyl alters the shape of electron orbitals and thus could modify the configuration and strength of the π-stacking interactions.

A similar kind of dependence is observed for the R52K substitution (Fig. 2d), which, in contrast, destabilizes complexes with methyl-substituted fluorogens. In the nanoFAST/**HBR-DOM2** structure^35^, the R52 sidechain is in contact with the 5-methoxy group, which is absent in **HMBR** and **HBR-2,5-DM**. This suggests that for the less bulky methyl-substituted ligands, R52 might form the stabilizing polar contact with the rhodanine moiety. This is consistent with the previous mutagenesis study, which reported that R52 is an important residue, taking part in the π-cation interactions with the FAST fluorogens of a different class, homologous to **N871b**^27,30^. In this case, the substitution of more rigid Arg to the flexible Lys would destabilize the packing and decrease the lifetimes exclusively for methyl-substituted compounds.

The P68K and P68T mutations both decrease the fluorescence lifetimes for all the ligands (Fig. 2e, f). P68 is adjacent to G69 - one of the key residues of FAST^21,27,30^. According to the structure of the nanoFAST/**HBR-DOM2** complex, G69 is able to form favorable polar contacts with the nitrogen of arylidene-rhodanines. Therefore, it is very important to stabilize this region of the protein. Proline is one of the most rigid residues due to the restricted conformational space of its backbone. One could expect that any kind of substitution at P68 would enhance the dynamics at G69 and consequently decrease the lifetimes of HBR ligands.

To conclude, the analysis of the mutations that were previously shown to destabilize the dynamics of FAST complexes for rhodanine- and imidazolone-based ligands^27^ allows finding a set of mutants that provide substantially different fluorescence lifetimes for several compounds.

### Fluorescence lifetime measurements in mammalian cells

**Fig. 3:**
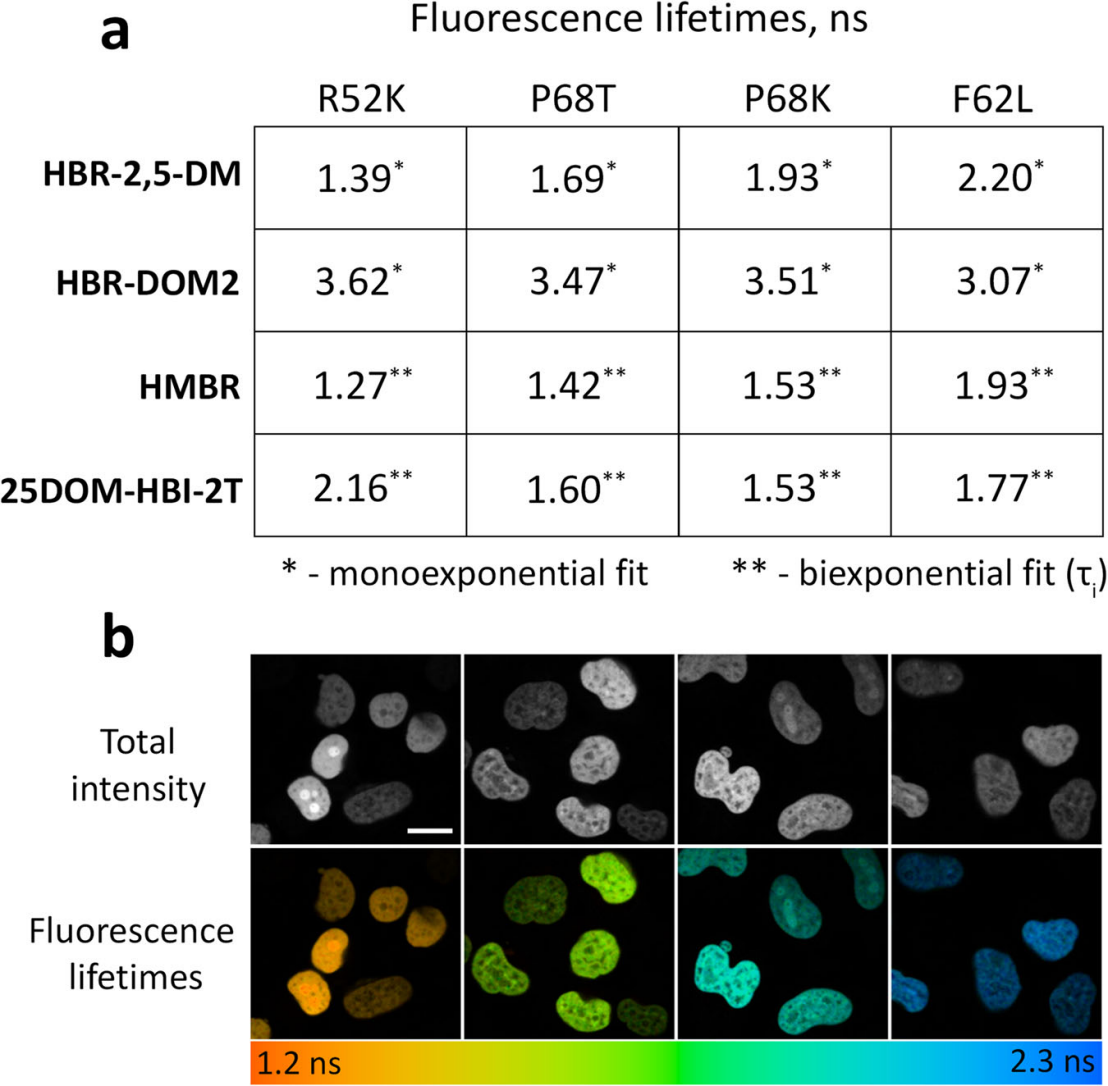
Fluorescence lifetimes of specific FAST-fluorogen complexes measured in cellulo. **a** Fluorescence lifetimes of fluorogens in complex with FAST variants expressed as H2B fuses in live HeLa cells (*n* = 16, see SI for more detail). The lifetimes shown in the table represent values averaged from multiple analyzed cells, and the full data can be found in the supporting information (Supplementary Tables S9, S11–26 and Supplementary Figs. S30–77). **b** Live-cell fluorescence lifetime microscopy of **HBR-2,5-DM** fluorogen with FAST variants R52K, P68T, P68K, and F62L (from left to right) expressed as H2B fuses (representative images, similar results in *n* = 46 (R52K), 47 (F62L), 45 (P68K), 48 (P68T) individual cells). Color-coding represents fluorescence lifetime obtained as a result of monoexponential fluorescence decay fitting. Scale bar 20 micrometers.

Since the lifetimes obtained in vitro may differ from those obtained in living cells under real FLIM conditions, we tested all selected FAST variants in cells expressing fusions of these proteins with H2B histones (Fig. 3, Supplementary Table S9, Supplementary Figs. S30–77). The nuclear localization of the probes, provided by the H2B-targeting, allowed a relatively uniform distribution of the fluorophore and high staining density, which are both favorable factors for reliable measurements of fluorescence decay kinetics in cellulo. Specifically, when a binning value of 4-5 was used for the decay data analysis, the kinetics of the fluorogen-activating proteins-H2B typically showed about 1000 photons at the peak, thereby allowing the usage of multicomponent fitting models (if needed) with adequate goodness (Supplementary Figs. S30–77, Supplementary Table S10). The fluorescence decay kinetics and lifetime values obtained differed from those measured in vitro (Supplementary Table S9). Notably, the emissions from the **HBR-2,5-DM** and **HBR-DOM2** ligands were more adequately fitted by a monoexponential function, yielding chi-square values comparable to or better than those from biexponential fitting (see the Methods section for more details and Supplementary Tables S9, S11–18 for mono- and biexponential fit comparisons). This likely results from the minimal contribution of the second component. **HMBR** and **25DOM-HBI-2T** retained the biexponential fluorescence decay pattern, while the fraction of the shorter exponent increased markedly compared to in vitro data (Supplementary Tables S19–26). The greatest changes in fluorescence lifetime values and decay kinetics were noted for **25DOM-HBI-2T**, suggesting its fluorescence is highly sensitive to environmental conditions. Phasor analysis of FLIM data corroborated the changes in fluorescence decay character and lifetimes revealed through the exponential fitting-based method. Specifically, centers of distribution of the fluorescent signal for monoexponential dyes **HBR-2,5-DM** and **HBR-DOM2** were located on the semicircle of the phasor space (Supplementary Fig. S78).

It should be noted that there is a large variety of data processing approaches in FLIM microscopy^36^. Nevertheless, in this study, we primarily utilized the approach based on exponential fitting of fluorescence decay data (both single- and two-component). Some of its results were cross-verified using the phasor approach, one of the most sought-after fitting-free methods. In the case of a multicomponent decay, the averaged lifetime obtained from the multicomponent fitting procedure typically can be presented in two ways, depending on how the components were averaged: the amplitude-weighted average lifetime (τ_m_) and the intensity-weighted average lifetime (τ_i_) (see the Methods section and especially the corresponding handbook^36^, pages 926-927 for more details). In various FLIM-related studies, both averaging methods are used for different purposes^37^. By definition, τ_i_ is close to lifetimes obtained by monoexponential fit or by modulation (frequency domain) techniques, while τ_m_ is rather proportional to the total fluorescence quantum efficiency. In the present study, we calculate both parameters (Supplementary Tables S9, S11–26) and present several images in both ways (Supplementary Figs. S30–77, S79). However, further in the main text we use only intensity-weighted average lifetimes for multicomponent decays.

In general, for each of the four substances, it would be possible to select a pair of FAST protein variants with different lifetimes distinguishable in FLIM. However, the broad distribution of lifetime values, revealed during the analysis of fluorescence signals from **HBR-DOM2** and **25DOM-HBI-2T** with all FAST variants (Supplementary Figs. S30–53), indicated their limited efficiency in FLIM multiplexing and prompted us to exclude these dyes from further experiments.

### Pairwise labeling and its analysis with FLIM

To examine how the lifetime multiplexing of other proposed pairs works, we genetically targeted the FAST mutants and co-expressed these fusion constructs in HeLa cells. The intracellular targets for the FAST variants to be visualized and multiplexed by FLIM were selected in a way to: (i) form a pronounced spatial staining pattern for each probe; (ii) to provide some degree of spatial overlap of the probes’ signals for further analysis. For pairwise multiplexing, the nuclear (targeted by histone 2B) and predominantly cytoplasmic albeit nuclei-adjacent^38^ vimentin-targeted localizations were used.

**Fig. 4:**
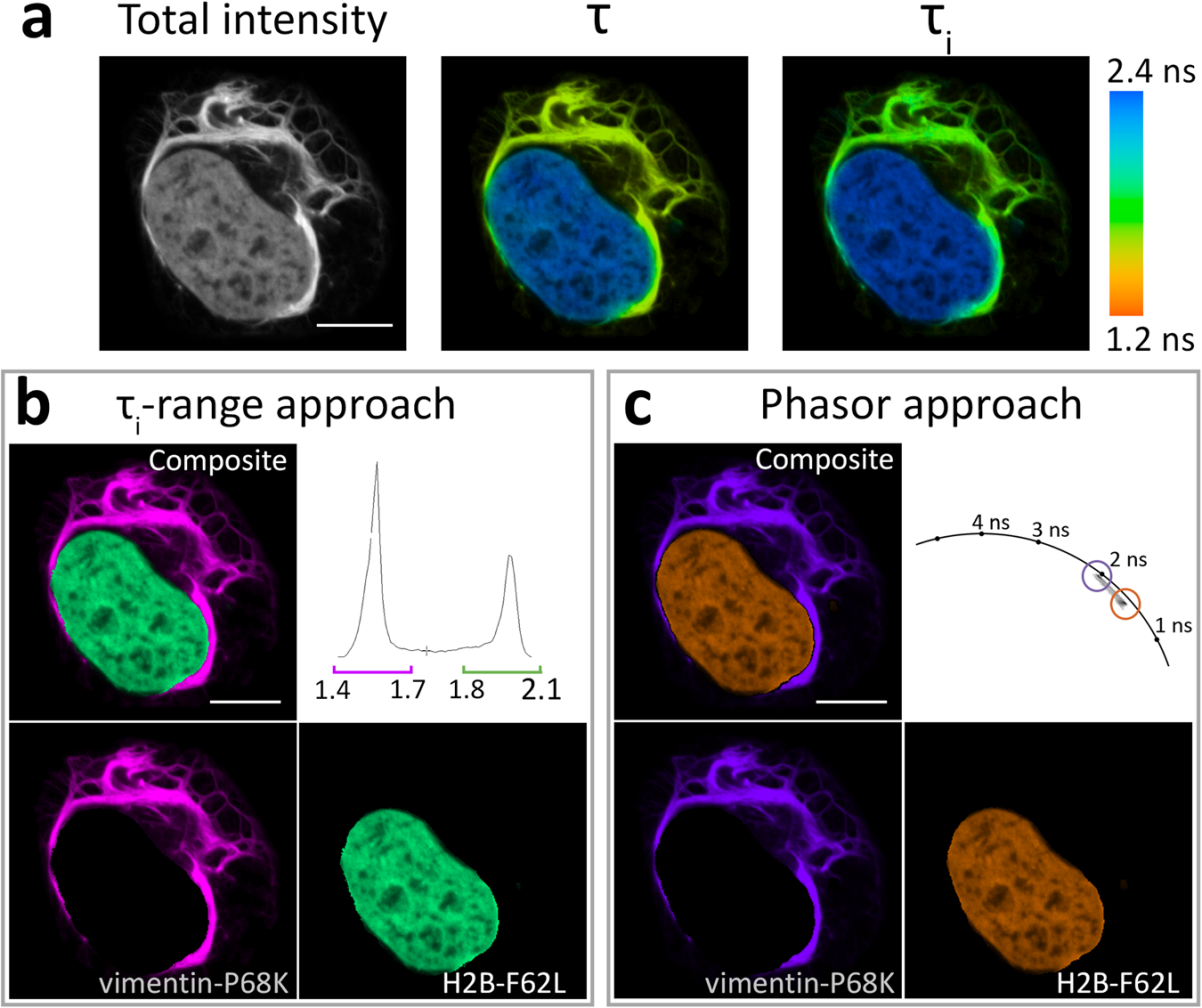
Example of the application of HMBR fluorogen in live-cell fluorescence lifetime multiplexing with two FAST variants. **a** Total intensity and color-coded FLIM images with mono- (τ) or biexponential (τ_i_) fit of **HMBR** in complexes with two FAST variants expressed simultaneously in live HeLa cells as H2B-F62L and vimentin-P68K fuses. Color-coding represents fluorescence lifetime (τ) or intensity-weighted average lifetime (τ_i_) and is specified on the right. **b** Result of color assignment to τ_i_ ranges specific to P68K or F62L complexes with **HMBR**, the composite and the individual localizations are shown. **c** Result of color assignment to phasor clusters circles specific to P68K or F62L complexes with **HMBR**, the composite and the individual localizations are shown. Representative image, similar results in *n* = 18 cells. More examples can be found in the supporting information (Supplementary Fig. S79). Scale bars 10 micrometers.

First, we successfully demonstrated such pairwise labeling for **HMBR** ligand, having inspected six specific pairs in total, with three of them showing well-pronounced signal contrast (one example in Fig. 4, other in Supplementary Fig. S79). Specifically, the average fluorescence lifetime histogram (τ_i_) for the **HMBR**-labeled cells expressing vimentin-P86K and H2B-F62L shows two distinct, non-overlapping peaks. Such τ_i_ distribution enables high-contrast differential coloring of the corresponding image regions (more details in the Methods section). Similarly, it was possible to clusterize the phasor plot for these cells using the lifetime clusters detected previously for **HMBR** with each FAST variant in H2B fusions (Supplementary Fig. S78), allowing the discrete visualization of localized variants.

Next, we performed dual-target imaging using the **HBR-2,5-DM** fluorogen. In the experiments involving histone labeling, this compound exhibited promising characteristics, behaving as a monoexponential fluorophore within its complexes with various FAST variants (Supplementary Tables S9, S11–14, Supplementary Figs. S66–77). It also demonstrated the most notable mutant-specific lifetime variances among the suggested compounds (Fig. 3A, Supplementary Table S9). Using the R52K, P68T, and F62L FAST variants and **HBR-2,5-DM**, we performed pairwise labeling of intracellular structures (Fig. 5, Supplementary Figs. S80–96). Similar to the previous experiment, the fluorescence lifetime distribution histogram revealed distinct peaks for different FAST variants, which, as expected, had even greater contrast in lifetime ranges. Importantly, in this case, we were able to utilize the τ value from the monoexponential fit with similar efficacy as the τ_i_ range to color-code the structures of interest, which, however, was likely possible not only because of the monoexponential decay of the corresponding complexes but also due to the minimal spatial overlap. The fitting-free phasor-clustering approach yielded a comparable result.

**Fig. 5:**
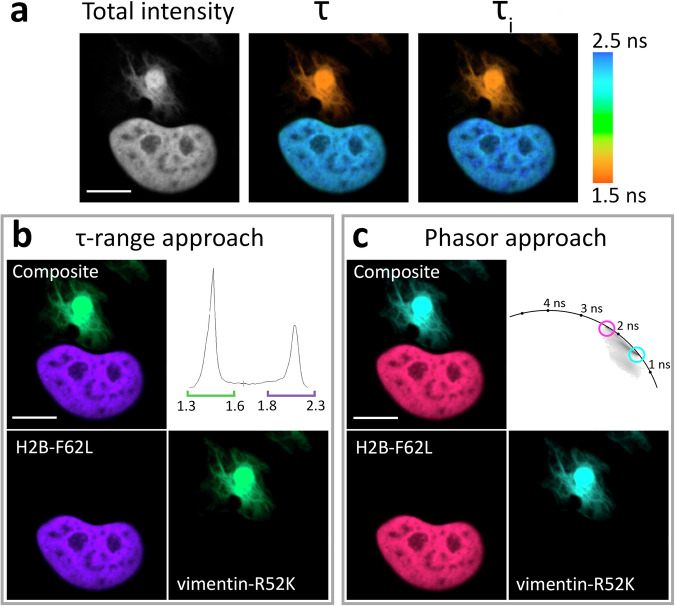
Example of application of HBR-2,5-DM fluorogen in live-cell fluorescence lifetime multiplexing with two FAST variants. **a** Total intensity and color-coded FLIM images with mono- (τ) or biexponential (τ_i_) fit of **HBR-2,5-DM** in complexes with two FAST variants expressed simultaneously in live HeLa cells as H2B-F62L and vimentin-R52K fusions. Color-coding represents fluorescence lifetime (τ) or intensity-weighted average lifetime (τ_i_) and is specified on the right. **b** Result of color assignment to τ ranges specific to R52K or F62L complexes with **HBR-2,5-DM**, the composite and the individual localizations are shown. **c** Result of color assignment to phasor clusters circles specific to R52K or F62L complexes with **HBR-2,5-DM**, the composite and the individual localizations are shown. Representative image, similar results in *n* = 18 cells. More data and other examples can be found in the supporting information (Supplementary Figs. S80–96). Scale bars 10 micrometers.

Considering the successful paired visualization of R52K, P68T, and F62L (Supplementary Figs. S80, S86, S92), we attempted a triple labeling with compound **HBR-2,5-DM**. Since the fluorescence lifetime can be highly sensitive to environmental changes, we first examined the photobehavior of this compound in several intracellular localizations other than the nucleus, where the FAST variants were targeted by fusing them with vimentin, IMS (intermembrane space of mitochondria), and β4Gal-T1 (beta-1,4-galactosyltransferase, Golgi apparatus). We revealed no dramatic differences between the fluorescence lifetimes measured in all four localizations (Supplementary Table S27) and proceeded to test more complex probe combinations.

### FLIM-based visualization of three intracellular targets

**Fig. 6:**
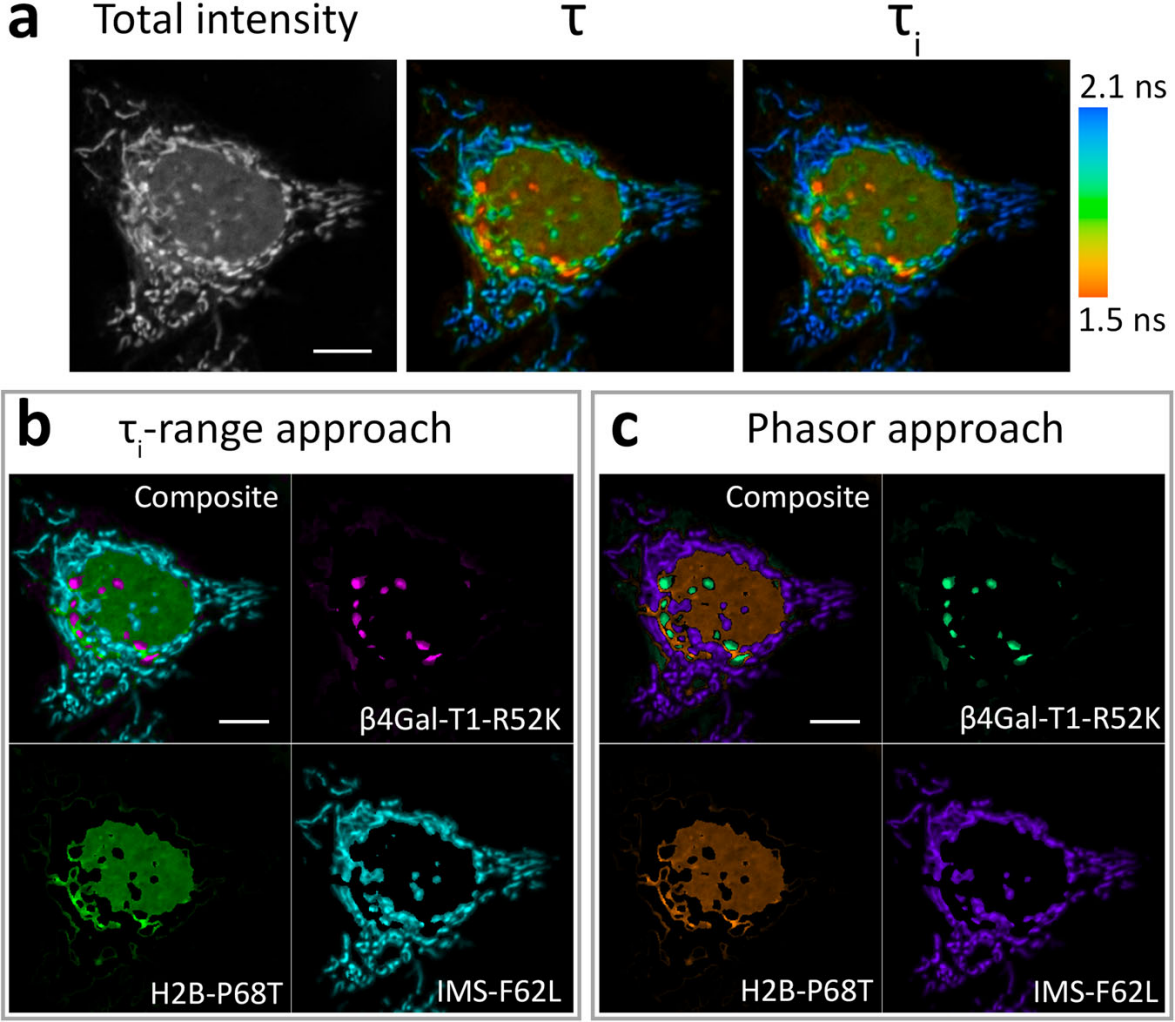
Example of application of HBR-2,5-DM fluorogen in live-cell fluorescence lifetime multiplexing with three FAST variants. **a** Total intensity and color-coded FLIM images with mono- (τ) or biexponential (τ_i_) fit of **HBR-2,5-DM** in complexes with three FAST variants expressed simultaneously in live HeLa cells as H2B-P68T, IMS-F62L (mitochondrial intermembrane space), and β4Gal-T1-R52K (Golgi apparatus) fuses. Color-coding represents fluorescence lifetime (τ) or intensity-weighted average lifetime (τ_i_) and is specified on the right. **b** Result of color assignment to τ_i_ ranges specific to R52K, P68T, or F62L complexes with **HBR-2,5-DM**, the composite and the individual localizations are shown. **c** Result of color assignment to phasor clusters circles specific to R52K, P68T, or F62L complexes with **HBR-2,5-DM**, the composite and the individual localizations are shown. Color-coding of τ_i_ ranges and phasor clusters are presented in Supplementary Fig. S98. Representative image, similar results in *n* = 25 cells. Scale bars 10 micrometers.

For triplet multiplexing, the nuclear, mitochondrial, and Golgi body targets were used, with all of them known to have slight spatial overlapping^37,39^. Generally, we succeeded in simultaneously visualizing three intracellular targets, each stained with **HBR-2,5-DM**, having typically collected signals of comparable amplitude from all probes in a single scan (Fig. 6). The differing fluorescence lifetimes of the probes allowed both the fitting-based and fitting-free procedures to clearly annotate the fluorescent signals of the targets in those areas of the image where they did not overlap. However, in the areas with co-localized signals, the color-coding performed by both approaches evidently resulted in similar artifacts (e.g., the mixture of R52K and F62L signals can be interpreted as P68T lifetime). We should also note that, despite the simultaneous presence of three protein variants with different lifetimes, the biexponential fit yielded the best results. On the contrary, when the triexponential model was used, we observed many areas with extremely low fit goodness (chi-square > 10) and irrelevant τ_i_ (Supplementary Fig. S97). Indeed, there are virtually no areas where all three components overlap, that makes triexponential fit redundant.

### Unmixing of the spatially overlapped signals

**Fig. 7:**
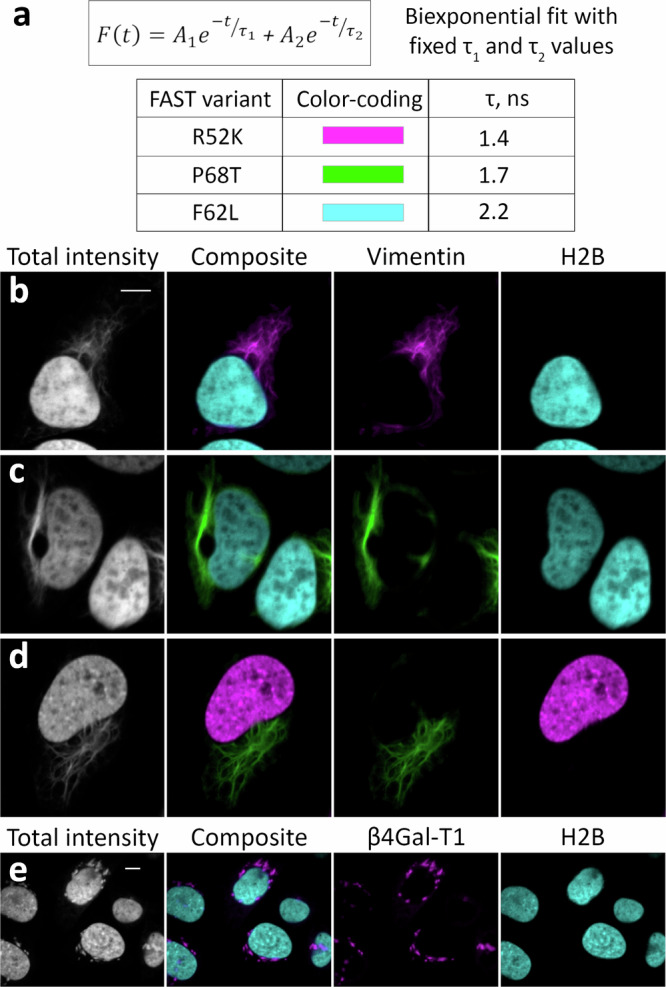
Signal separation in pairwise FLIM multiplexing of intracellularly targeted FAST variants in live HeLa cells. The **HBR-2,5-DM** fluorogen was used in all cases. The fitting-based approach with fixed τ from previously obtained data for corresponding complexes (see Methods for more detail) was applied to separate time-resolved fluorescence signals from differently localized FAST probes, including co-localized signals. **a** Color-coding legend. The lifetime values shown in the legend were obtained via the monoexponential fitting of FAST-R52K, P68T, and F62L expressed as H2B-fuses (Fig. 3a, Supplementary Table S9). Color brightness indicates the relative contribution (amplitude) of the respective exponential component calculated via the full-image biexponential fitting using the fixed lifetime values mentioned above. **b**–**e** Live-cell fluorescence lifetime microscopy of **HBR-2,5-DM** fluorogen in complexes with the genetically targeted FAST variants expressed in HeLa cells. Grayscale leftmost images show fluorescence intensity; composite images are designed to show the distribution (color) and amplitude (brightness) of two fluorescence populations calculated based on the full-image biexponential fitting; images named according to specific fusion partners are designed to show the distribution of individual fluorescence population represented within the image. **b** H2B-F62L and R52K-vimentin (representative image, similar results in *n* = 18 cells). **c** H2B-F62L and P68T-vimentin (representative image, similar results in *n* = 17 cells). **d** H2B-R52K and P68T-vimentin (representative image, similar results in *n* = 17 cells). **e** H2B-F62L and β4Gal-T1-R52K (Golgi apparatus) (representative image, similar results in *n* = 12). Scale bar 10 micrometers.

Noting that the activation of **HBR-2,5-DM** was characterized by the appearance of fluorescence with monoexponential decay (Fig. 3), we found it reasonable to check our ability to quantitatively analyze spatially overlapping signals upon multiplexing with this fluorogen. Particularly, we detected several regions with a pronounced overlap of signals from vimentin-R52K and mitochondria-F62L in FLIM images with triplet multiplexing and applied a fitting-based analysis to reveal the fluorescent components that indeed were found to correspond well to those determined in **HBR-2,5-DM**-R52K’s and **HBR-2.5-DM**-F62L’s single probe labeling (Supplementary Fig. S99). Inversely, in pairwise multiplexing using vimentin-R52K/H2B-F62L, vimentin-P68T/H2B-F62L, H2B-R52K/vimentin-P68T, and β4Gal-T1-R52K/H2B-F62L localizations, we succeeded in separating the signals (including the overlapped ones) based on the data from single probes’ monoexponential fitting (Fig. 7). For this purpose, we performed biexponential fitting with fixed τ_1_ and τ_2_ values corresponding to the lifetimes of the pairs used. The separation of color channels was made using the contribution of the corresponding components at each point (see Live cell Fluorescence lifetime imaging microscopy processing in the Methods section for more detail).

## Conclusion

In this paper, we proposed a FLIM multiplexing system based on variants of the fluorogen-activating protein FAST and successfully used it for the labeling of a wide variety of cellular components. The proposed genetically encoded tags are small, they are based on very similar proteins with only one amino acid substitutions, and utilize one external fluorogen for various lifetimes. The proposed system is smaller than most other genetically encoded tags, which should result in a weaker influence on the behavior of the labeled object. Moreover, the identical size of the labels we proposed will result in an identical effect on the labeled objects.

More importantly, at least eight examined protein-fluorogen pairs exhibited a monoexponential fluorescence decay upon FLIM, thus making a fitting-based analysis of their fluorescence data a potentially efficient approach, including its sophisticated modalities such as global fitting. We believe that the monophasicity of fluorescence decay represents an essential property of the probe’s signal for both fitting-free and fitting-based fluorescence lifetime multiplexing. Monophasic decay provides fewer cross-talks and greater potential for further signals analysis upon co-localized and/or spatially overlapped labeling compared to the bi- or triphasic decays in both fitting-based and fitting-free approaches^36,40,41^.

In the present study, we demonstrated the simultaneous imaging of two to three spatially separated intracellular targets, with the potential for quantitative analysis and separation of co-localized signals from two probes. However, we encountered difficulties with the unmixing of signals for triple labeling. This could be due to insufficient differences in fluorescence lifetimes within the triad, the contribution of autofluorescence from endogenous cellular fluorophores, or the methodological limitations of the tools we used. We anticipate that the use of more advanced equipment with reduced noise levels could facilitate the successful implementation of our system in more complex cases. Furthermore, a global analysis (e.g., see ref. ^42^; reviewed in ref. ^43^) of the FLIM data for partially overlapped signals from multiple monoexponential protein-fluorogen pairs could potentially allow the identification and separation of additional fluorescence populations localized at different cell structures, provided that the photon count and fluorescence lifetime difference are sufficient.

The data presented here on the multiplexing of two or three chemogenetic probes in a single spectral channel clearly demonstrate the proof-of-concept of our imaging approach. However, the potential for further development is evident, given that the total number of possible [FAST variant – fluorogen] combinations is virtually unlimited. These fluorophores could cover the entire visible spectrum and a wide range of fluorescence lifetimes, thus enabling dozens of cellular targets to be visualized. Although previously described multiplexing platforms based on SNAP- and Halo-tags^7,13^ are expected to possess the same advantage (high combination diversity), their performance in the fitting-based fluorescence lifetime multiplexing remains unclear. Among the HaloTag variants, only HaloTag7 and HaloTag9 were shown to produce predominantly monoexponential fluorescence decays (see Tables S8 & S15 in ref. ^13^), while all four FAST mutants (R52, P68T, P68K, F62L) tested in our study behaved this way. Interestingly (in contrast to the HaloTag system), the fluorescence homogeneity (decay monophasicity) in the case of the FAST platform was seemingly determined by the type of fluorogen rather than the protein structure. Apparently, arylidene-azolones are characterized by a very small number of main pathways for the release of excited state energy. In addition to fluorescence, they can undergo only the isomerization of the arylidene fragment (which is responsible for the second, smaller component of the lifetime of the complexes presented by us), which is markedly blocked by the protein environment.

In this contribution, we demonstrate only a single modality of the protein-fluorogen multiplexing technique, where one fluorogen binds several targeted FAST variants. Considering the chromophore exchange possibility, we might expect more sophisticated variants of the technique to be developed. Thus, in contrast to the system based on self-labeling proteins with covalent chromophore binding, our system (and possibly also another FAST-based multiplexing system described in the recent preprint^26^) could be combined with microfluidic or continuous-flow equipment to provide a gradual dye substitution accompanied by respective changes in the labeling patterns (specifically, fluorescence lifetime values and intracellular distribution of the dyes). This may also be important for long-term experiments in which the permanent presence of the dye and fluorescence is undesirable. Moreover, mosaic competitive staining, where several fluorogens compete for one or more FAST variants, could open up landmark possibilities for single-molecule FLIM (e.g., as probes for smFRET-related^44^ or PAINT-derived^45^ techniques, or as a kind of sensors probing the immediate environment of the label upon high-density labeling of large molecular complexes, where monitoring the change in fluorogen binding specificity can be a source of structural information). Finally, one can propose the engineering of fluorescence indicators, in which circularly permuted FAST variants with a permutation point located near the fluorogen-binding pocket are used.

## Methods

### General

All solid fluorogens were dissolved in DMSO (Sigma Aldrich, Molecular biology grade, #cat D8418) in 5 mM concentration, and stored in a dark place at −20 °C for no more than 3 months. UV-VIS spectra were recorded on a Varian Cary 100 spectrophotometer. Fluorescence excitation and emission spectra were recorded on an Agilent Cary Eclipse fluorescence spectrophotometer.

### Synthesis

(Z)-5-(4-hydroxy-2,5-dimethoxybenzylidene)-2-thioxothiazolidin-4-ones (**HMBR,** **HBR-2,5-DM** and **HBR-DOM2**)^30^ and (Z)-5-(4-hydroxy-2,5-dimethoxybenzylidene)-3-methyl-2-(trifluoromethyl)-3,5-dihydro-4H-imidazol-4-one (**25DOM-HBI-2T**)^32^ were taken from our laboratory stock.

### Plasmids

A pET24b(+) expression vector was used for in vitro screening and for protein production in *E. coli*. All genetic constructs coded in pET24b(+) expression vectors were purchased from the commercial source (Cloning Facility, Russia). Coding sequences were cloned into pET24b(+) backbone with C-terminal his-tag (GGGHHHHHH).

Coding sequences of FAST variants in Level 0 plasmids were also ordered from the commercial source (Cloning Facility, Russia). FAST variants R52K, F62L, P68T and P68K fused to localization-specific signals and proteins were cloned by Golden Gate assembly, following the MoClo syntax^46,47^. The constructs were put under a CMV promoter and possessed a SV40 poly(A) sequence. BpiI (BbsI) and Eco31I (BsaI) restriction endonucleases (Thermo Scientific, Waltham, MA, USA) and T4 DNA ligase (Evrogen, LK001) were used for the cloning procedure. H2B (originally from pTagRFP-H2B, Evrogen, FP368), vimentin (originally from pTagRFP-vimentin, Evrogen, FP380) and β4Gal-T1 (originally from pTagRFP-Golgi, Evrogen, FP367) coding level 0 plasmids were available inhouse. IMS-HyPer2 (Addgene plasmid no. 60248) was a gift from V. Belousov. IMS level 0 plasmid was constructed using standard cloning procedure. PTS1 sequence was added to the FAST R52K coding sequence during Golden Gate assembly as a pair of overlapping oligonucleotides with appropriate overhanging sticky ends. PTS1 sequence is equivalent to the pVenus-PTS1^48^.

### Fluorescence lifetime screening in Macro-FLIM imaging setup

The plasmids (pET24b(+)) were transformed into chemically competent BL21 (DE3) *E. coli* strain with heat-shock transformation. Then the strains were plated to Petri dishes with LB media supplemented with agar-agar and grown overnight at 37 °C. Then a single colony of each variant strain was dissolved in 50 ml of sterile Phosphate-Buffered Saline (PBS, pH 7.4, #cat E404-200TABS, Amresco) buffer solution, and 5 μL of the solution was put on the ampicillin agar plate to make 7 mm bacterial spot and grown overnight.

For the first lifetime screening of FAST variants in bacteria plated on Petri dishes, we adapted the Lambert Instruments FLIM Attachment (LIFA) setup for imaging, which is originally designed to measure the fluorescence lifetime on a microscope.

Based on this system, we assembled a macro-FLIM setup, which consists of a camera (Lambert Instruments^49^), a microscope light filter (Chroma LP 510 nm), C-mount to Nikon-F mount 3D-printed adapter, DSLR camera lens (Nikon 70−300 mm f/4.5-6.3 G ED VR AF-P DX), a Multi-LED light source (Lambert Instruments^50^) and a beam expander lens. This setup is shown in the Supplementary Picture S1. The setup allows to fully illuminate an assayed Petri dish by passing the blue light through the expander lens. The camera lens was placed in a way that the Petri dish occupied the entire field of view.

For calibration of macro-FLIM setup, 12*12 cm green fluorescent plastic slide custom-made in ThorLabs (4.2 ns lifetime) was used. The data was additionally verified using 10 uM fluorescein solution in 0.1 M Tris-HCl pH10 (4.02 ns lifetime).

LIFA software was used for control of camera and light source and image processing^51^

Imaging parameters:

Excitation light wavelength – 471 nm,

Fluorescence filter Chroma Long pass 510 nm

Modulation frequency – 20 MHz,

Number of phases – 12,

Gain – 2,

Exposure – 300 ms,

Number of frames per phase – 10.

For the imaging step, 10 μL of 10 μM solution (prepared from stock solutions in DMSO with 10 mM concentration by dissolution in PBS) of corresponding fluorogens were spilled onto each bacterial spots expressing FAST variants and original FAST protein right before the experiment. Next, using abovementioned Macro-FLIM setup, we performed the imaging and lifetime determination of each bacterial spot.

Fluorescence lifetime was determined in LIFA software. Regions of interest were carefully drawn around bacterial spots. Each region of interest contained around 300−500 pixels. Then average intensity, average phase lifetime, average modulation lifetime and standard deviation for each parameter were calculated. The obtained data are presented in Supplementary Tables S2−5.

### Proteins production and purification

FAST, FAST-R52K, FAST-F62L, FAST-P68K and FAST-P68T were produced similar to described earlier^30^. Proteins were expressed in *E. coli* BL21(DE3) cells and M9 minimal salts medium was used. The bacterial cells were cultivated at 37 °C in 2 l Erlenmeyer flasks (New Brunswick Innova 44 R shaker, 250 rpm) until OD600 ~ 0.6. Protein expression was induced by 0.25 mM isopropyl β-d-1-thiogalactopyranoside (IPTG) and after cultivation for 4–5 h, the cells were harvested by centrifugation at 7000 × g for 10 min at 4 °C and stored at −20 °C. The cells from 600 ml of M9 medium were resuspended in 30 ml of lysis buffer (20 mM Tris, pH 8.0, 500 mM NaCl, 20 mM imidazole) equipped with 200 μM phenylmethylsulfonyl fluoride and disrupted on ice by 15–20 circles of ultrasonication (Bandelin Sonoplus, GM 2200, titanium tapered tip KE 76) in pulse mode with an active interval of 25 s at 60% of power followed by cooling for 3 min. The clarified lysate (14,000 × g for 60 min at 4 °C) was filtered using a Millipore filter unit with 0.22 μm pore size and loaded into the column equipped with circa 5 ml Ni^2+^ Sepharose HP resin (GE) pre-equilibrated with a lysis buffer. The column was washed with 5–7 column volumes of immobilized metal ion affinity chromatography buffer (20 mM Tris, pH 8.0, 200 mM NaCl) with 20 mM imidazole followed by 5 column volumes of similar buffer with 50 mM imidazole. Target protein was eluted by immobilized metal ion affinity chromatography buffer with 500 mM imidazole. After SDS-PAGE analysis, fractions containing the pure target protein were combined and dialyzed against the 1xPBS buffer with 1 мМ EDTA overnight at 4 °C (Supplementary Fig. S1). If extra purification step was needed, the protein was concentrated up to 15 mg/ml by ultrafiltration (10 kDa MWCO, Amicon Ultra), clarified at 25,000 × g for 1 h and loaded to a Superdex 75 Tricorn 10/300 (GE) gel filtration column equilibrated in 1xPBS buffer. For NMR applications target protein was dialyzed against the NMR-buffer (20 mM NaPi, pH 7.0, 20 mM NaCl) overnight at 4 °C and concentrated up to 15 mg/ml.

### Fluorescence lifetime measurements in vitro

Fluorogens were mixed (from stock solutions in DMSO 10 mM) with proteins in PBS buffer at room temperature. Final concentrations were 10 μM and 0.2 μM for proteins and fluorogens respectively. Measurements were made using a time-resolved miniTau fluorescence spectrometer (Edinburgh Instruments, Livingston, UK) in a 20 ns (**HBR-2,5-DM,** **HMBR,** **25DOM-HBI-2T**) or 50 ns (**HBR-DOM2**) window divided into 1024 or 2048 time channels respectively. The fluorescence was excited using an EPL-450 picosecond laser (Edinburgh Instruments, Livingston, UK) with a central emission wavelength of 445.6 nm and a repetition rate of 20 MHz. The photons were counted in the spectral range of 525−575 nm. The data processing, visualization and determination of chi-square (Pearson’s test) were carried out using the Fluoracle 2.5.1 software (Edinburgh Instruments, Livingston, UK). Fluorescence decay curves are presented in Supplementary Figs. S2–21. In cases where fitting by deconvolution with the instrument response function (IRF-based fit) failed or gave unsatisfactory goodness after several attempts with exponential models of varying complexity, we used the so-called tail fit approach, which involves fitting of the data after the fluorescence peak only.

### Determination of affinity constants

The affinity constants for complexes [protein-fluorogen] were determined by spectrofluorometric titration of protein by fluorogen solutions with various concentrations on the Tecan Infinite 200 Pro M Nano dual mode plate reader. The protein concentration was 0.10 µM. Least squares fit (line) gave the dissociation constants K_d_ presented in Supplementary Table S7.

The titration experiments were performed at 25 °C in pH 7.4 PBS. Fitting was performed using Origin 8.6 software.

### Spectra of complexes

Optical properties of complexes were investigated using 5 µM solutions for absorption spectra registration and 0.5 µM for emission spectra registration in PBS buffer (pH 7.4, #cat E404-200TABS, Amresco).

### Extinction coefficients determination

The fluorogens solutions were mixed with a protein solution in PBS buffer (pH 7.4, #cat E404-200TABS, Amresco). The final concentration of fluorogens was 5 µM. Proteins were added in such amount that lead fraction of the protein:fluorogen complex ≥97%. The total protein concentration for each complex was calculated using the equation:

1 $$[{\Pr }_{0}]=\frac{{K}_{d}\times \left(\alpha \times \left[{{Chr}}_{0}\right]\right)}{\left[{{Chr}}_{0}\right]-\left(\alpha \times \left[{{Chr}}_{0}\right]\right)}+(\alpha \times \left[{{Chr}}_{0}\right])$$

where, *K*_*d*_ – dissociation constant, [*Chr*_*0*_] – total fluorogen concentration, α - fraction of the protein:fluorogen complex.

This equation is the result of rearranging of two equations for the dissociation constant and equilibrium concentration of the protein:fluorogen complex.

2 $${K}_{d}=\frac{(\left[{\Pr }_{0}\right]-\left[{Complex}\right])\times (\left[{{Chr}}_{0}\right]-\left[{Complex}\right])}{[{Complex}]}$$

3 $$\left[{Complex}\right]=\alpha \times \left[{{Chr}}_{0}\right]$$

where, *K*_*d*_ – dissociation constant, [*Chr*_*0*_] – total fluorogen concentration, [Pr_0_] – total protein concentration, [Complex] – equilibrium concentration of the protein:fluorogen complex, α - fraction of the protein:fluorogen complex.

The molar extinction coefficient was calculated by the formula:

4 $${{{{{\rm{\varepsilon }}}}}}=\frac{A}{{cl}}$$

where *A* is the absorbance intensity at maxima, *c* is the molar concentration of complexes, *l* is the pathlength.

### Fluorescence quantum yields determination

Fluorescence quantum yields for complexes were calculated according to the procedure described in the literature^52^ with the use of Rodamine 6 G as standard. The fluorogens concentration was 5 µM for absorption and 1.67, 0.5, and 0.167 µM for emission, proteins were added in such concentration that lead α > 97% (see Eq. 1). The quantum yield was calculated by the formula:

5 $${\Phi }_{x}={\Phi }_{{st}}\times \frac{{F}_{x}}{{F}_{{st}}}\times \frac{{f}_{{st}}}{{f}_{x}}\times \frac{{n}_{x}^{2}}{{n}_{{st}}^{2}}$$

where *F* is the area under the emission peak, *f* is the absorption factor (see below), *n* is the refractive index of the solvent, Φ is the quantum yield, the subscript *x* corresponds to the novel compounds, the subscript *st* – for standards.

6 $$f=1-{10}^{-A}$$

where *A* is absorbance at the excitation wavelength.

### Analysis of fluorescence lifetimes

The fluorescence lifetimes and fluorescence quantum yields were used to extract the rates of radiative (k_r_) and non-radiative (k_nr_) transitions, following the equations:

7 $${k}_{r}=\frac{\Phi }{\tau }$$

8 $${k}_{{nr}}=\frac{1-\Phi }{\tau }$$

where Φ is the quantum yield and τ is the lifetime of fluorescence, corresponding to τ_1_ measured in vitro, see Table 1.

To analyze the structural basis of how mutations affect the fluorescence lifetime of FAST/ligand complexes, the spatial structure of nanoFAST/**HBR-DOM2** complex, PDB code 8AO0, was visualized in PyMOL (Schrödinger LLC). This structure was selected, because the ligand, **HBR-DOM2**, is one of the ligands that were tested in the current study. The mutations were introduced using the standard mutagenesis wizard of PyMOL and one of the possible rotamers with the fewest steric clashes was selected to be depicted. The sequence numbering was adapted to the numbering of FAST protein, by adding 26 to the residue number in nanoFAST. Aminoacid sequences of nanoFAST and FAST are identical, excluding the removal of the first 26 amino acids.

### Live-cell fluorescence lifetime imaging microscopy

HeLa Kyoto cells (origin: EMBL, RRID: CVCL_1922) were obtained from previously established stock of our laboratory. Cells were seeded onto 35 mm glass-bottomed culture dish (SPL Life Sciences, Gyeonggi-do, Korea) and grown in the DMEM medium (PanEco, Moscow, Russia) with 10% (*v/v*) FBS (fetal bovine serum; Sigma, St. Louis, MO, USA) containing 50 U/mL penicillin and 50 μg/mL streptomycin (PanEco) (DMEM complete) at 37 °C and 5% CO_2_ for 24 h before transfection.

Transient transfection was performed using polyethylenimine, PEI (#23966-1, Polysciences, USA). DMEM complete was changed for Opti-MEM 1 h before transfection procedure. 3 μL of PEI were mixed with 250 μL of Opti-MEM per dish, in a separate tube 1.0 μg of plasmid DNA was mixed with 250 μL of Opti-MEM for expression of H2B-FAST variant fusion. 4,5 μL of PEI and 1,5 μg of plasmid DNA mixture were used in case of co-transfection with two plasmids, and 6 μL of PEI and 2 μg of DNA in case of three-plasmid co-transfection. PEI-containing mixture was incubated for 5 min, following which PEI- and DNA-containing media were mixed and incubated for 20 min. PEI-DNA mixture was added to cells dropwise, cells were incubated for 3 hours and transfection media was replaced with DMEM complete. The cells were incubated under the same conditions for 24–48 h before imaging.

FLIM of live HeLa Kyoto cells was performed in 2 mL of Hanks’ Balanced Salt Solution (PanEco) with 10 mM HEPES (Sigma) and 5 μM fluorogen (added from 10 mM DMSO stock solution) at room temperature using a Nikon TE-2000U microscope with a Nikon 100x S Fluor 0.5–1.3 oil iris objective, equipped with the Becker&Hickl DCS-120 scanning confocal module and HPM-100-40 or PMC-100-1 detector. For fluorescence excitation, a Fianium WhiteLase SC-450-6 laser at a repetition rate of 60 MHz (**HBR-DOM2)** or 40 MHz (**HBR-2,5-DM,** **HMBR** and **25DOM-HBI-2T**) was used. Average input laser power was 1.5 mW, 488 nm laser line was generated by AOTF. To precisely adjust irradiation intensity continuously variable neutral density filters were used. Fluorescence emission signal was filtered by HQ495LP + HQ525/50 (**HBR-2,5-DM** and **HMBR**) filter set (Chroma) or HQ495LP + 580bp40 (**HBR-DOM2** and **25DOM-HBI-2T**) filter set (Omega Optical).

### Live-cell fluorescence lifetime imaging microscopy processing

SPCM Data Acquisition Software (SPC-150 v.9.87) (Becker & Hickl, Germany) was used to control abovementioned Becker & Hickl TCSPC devices and to acquire the data. SPCM produces files in .sdt format that contain time correlated single photon counting instrumentation parameters and measurement data. SPCImage software 8.6 and 8.9 (Becker & Hickl, Germany) were used for fluorescence decay analysis of this data. Data acquisition and analysis were performed separately on different computers, therefore M1 SPC-150 Emulation v.9.87 was used to import .sdt files to SPCImage 8.6 or 8.9 (Main>Send Data to SPCImage).

The fluorescence decay curve can be described by the equation:

9 $$F\left(t\right)={\sum }_{k=1}^{n}{A}_{k}{e}^{-t/{{{{{{\rm{\tau }}}}}}}_{k}}$$

Where F(t) is the fluorescence intensity as a function of time, τ_k_ is the fluorescence lifetime of the *k-*th decay component and A_*k*_ is the component amplitude.

SPCImage software allows such fitting at each pixel using a similar equation containing from one to three components whose parameters can be adjusted in the “Multiexponential Decay” window. Maximum likelihood estimation method of fitting was used. Pearson’s chi-squared test value is used to measure goodness of fit. The quality of fit can be also estimated visually using residuals – graphical representation of deviations between photon data and fit-trace. Both parameters can be found in the “Decay-Graph” window. All FLIM data were exponentially fitted using the algorithm based on the deconvolution with instrument response function. We used a synthetic instrument response function produced automatically by SPCImage, since the fluorescence lifetimes under analysis were all well greater than the pulse width. To improve the quality of the fit, we also use a procedure called binning. Binning means the summation of photons from neighboring pixels for fitting procedure. Thus, for every pixel of the image, it uses not only the photons in this pixel but also the photons in the pixels around. The ‘square’ binning strategy was used. More information about this and other procedures can be found in corresponding guides^36,53^.

Selected parameters can be applied for a fitting in all pixels using the “Calculating of decay matrix” command. A_*k*_, τ_k_ and Chi-square are determined in every pixel of the image as well as amplitude-weighted average lifetimes (τ_m_, which determines color-coding of FLIM image in the software by default) and intensity-weighted average lifetime (τ_i_). The intensity of color in every pixel in this default picture is determined by the number of the photons in it.

Amplitude-weighted average lifetime (τ_m_) weights each lifetime component (τ_k_) by their amplitude coefficients (A_*k*_):

10 $${{{{{{\rm{\tau }}}}}}}_{m}=\frac{{\sum }_{k=1}^{n}{A}_{k}{{{{{{\rm{\tau }}}}}}}_{k}}{{\sum }_{k=1}^{n}{A}_{k}}$$

Intensity-weighted average lifetime (τ_i_) weights each lifetime component (τ_k_) by their integral intensities. The integral intensity of a lifetime component is the product of its lifetime and its amplitude (A_*k*_):

11 $${{{{{{\rm{\tau }}}}}}}_{m}=\frac{{\sum }_{k=1}^{n}{A}_{k}{{{{{{\rm{\tau }}}}}}}_{k}2}{{\sum }_{k=1}^{n}{A}_{k}{{{{{{\rm{\tau }}}}}}}_{k}}$$

To determine the most suitable fitting model, a comparative analysis of the fitting quality provided by single-component and two-component exponential models was carried out for each fluorogen-protein pair. Considering the relatively large typical number of photons per decay, we believed that provided there is adequate overlay of the exponential curve on the raw data and no pronounced functional dependence in the distribution of residuals, a chi-square value less than or equal to 1.2 can be considered a sign of nearly perfect fitting goodness. Importantly, when obtaining fits of comparable quality by exponential models of different complexity, the simpler model was always preferred.

More specific protocols for data processing and images generation are presented below.

Data processing for Fig. 3a, Supplementary Tables S9–27

1. Blue crosshair was moved to the region of the cell with an even and medium intensity signal.
2. The binning factor (n) was set to 4-5 (“Bin” in the “Decay-Graph” window). The range of time channels used for fitting was defined by adjusting T1 and T2 values so they included the whole decay curve and excluded the baseline regions. Threshold parameter in the “Decay-Graph” window was set to 50–150. For fitting of decay data of FAST pairs with **HBR-2,5-DM** the option with no binning (“Bin” was set to zero, Supplementary Table S10) was also performed, threshold in this case was set to 5.
3. Both mono- and biexponential fits were used for comparison in Supplementary Tables S9, S11–26. The number of exponential components were set in a “Multiexponential Decay” window to 1 or 2 respectively. Supplementary Tables S10, S27 contain monoexponential fit data only.
4. Several random cells were selected for analysis from 4-6 fields of view in each experiment.
5. Fitting results (τ, τ_m_, τ_i_, τ_1_, τ_2_, A_1_, A_2_) were analyzed using Origin 8.6 Software.

Data processing for Supplementary Figs. S30-S77, S80, S83, S86, S89, S92

1. Blue crosshair was moved to the region of the cell with an even and medium intensity signal.
2. The binning factor (n) was set to 4-5 (“Bin” in the “Decay-Graph” window). The range of time channels used for fitting was defined by adjusting T1 and T2 values so they included the whole decay curve and excluded the baseline regions. Threshold parameter in the “Decay-Graph” window was set to 50−150.
3. The decay matrix was generated, using the corresponding command (Calculate>Decay matrix in main menu) with monoexponential (default) fitting. Screenshots of software windows were made for corresponding figures.
4. The number of exponential components were raised to two in a “Multiexponential Decay” window and the new decay matrix was generated in τ_m_ color-coding (default). Screenshots of software windows were made for corresponding figures.
5. Color-coding mode was changed to τ_i_ (Options>Color, “Coding of”). Screenshots of software windows were made for corresponding figures.

Data processing for Figs. 3b, 4a, 5a, 6a, and Supplementary Figs. S79, S95, S96

1. Steps 1-2 from previous protocol were made identically.
2. The decay matrix was generated, using the corresponding command (Calculate>Decay matrix in main menu) with monoexponential (default) fitting. For Figs. 3b, 4a, 5a, 6a, and Supplementary Figs. S95, S96 resulting FLIM images were exported in .tiff format (File>Export…, “Color coded image” and “Gray-scale image”)
3. The number of exponential components were raised to two in a “Multiexponential Decay” window and the new decay matrix was generated in τ_m_ color-coding (default). For Supplementary Fig. S79 resulting FLIM images were exported in .tiff format (File>Export…, “Color coded image” and “Gray-scale image).
4. Color-coding mode was changed to τ_i_ (Options>Color, “Coding of”). For Figs. 4a, 5a, 6a, and Supplementary Figs. S79, S95, S96 resulting FLIM images were exported in .tiff format (File>Export…, “Color coded image”).
5. All exported figures were prepared for publication in FIJI 1.53t.

Data processing for Figs. 4b, 5b, 6b, and Supplementary Figs. S95, S96

1. Steps 1-2 from protocol for Supplementary Figs. S30–77, S80, S83, S86, S89, S92 were made identically.

- For Fig. 5b, and Supplementary Figs. S95, 96 further actions were carried out using monoexponential fitting, the decay matrix was generated, using the corresponding command (Calculate>Decay matrix in main menu) with monoexponential (default) fitting.
- For Figs. 4b and 6b the number of exponential components were raised to two in a “Multiexponential Decay” window and the decay matrix was generated, color-coding mode was changed to τ_i_ (Options>Color, “Coding of”).

3. The color-coding was set to discrete mode (Options>Color, “Mode discrete”) that allows to define specific ranges of τ/τ_i_ for separate Red, Green and Blue channels.
4. The range of τ/τ_i_ values was assigned to each separate color channel (Options>Color). Ranges were chosen in such a way that each included one previously calculated FAST variant lifetime (the data obtained on H2B-FAST fuses) and located symmetrically around the photon peak in the histogram (presented in Distribution window above Decay-Graph window); τ/τ_i_ ranges were set in a way that they did not overlap. In case of separation of 2 localized FAST variants expressed in one cell, one color channel was left empty. At this step all photons outside the probe-specific τ/τ_i_ ranges were discarded.
5. Resulting FLIM images were exported in .tiff format and imported to FIJI 1.53t as RGB Color type files.
6. RGB files were transformed to RGB stack (Image>Type>RGB stack) and images from stack were separated (Image>Stacks>Stack to Images). This procedure resulted in three images; each image contained pixels that had a τ/τ_i_ from an assigned range (in case of 2 localized FAST variants one color channel was empty).
7. Each image from the original stack was transformed to 16-bit files (Image>Type>16 bit) and color-coded in a colorblind friendly palette (Image>LUT).

Data processing for Figs. 4c, 5c, 6c, and Supplementary Figs. S78, S95, S96 (Phasor approach)

1. The binning factor (n) was set to 3 (“Bin” in the “Decay-Graph” window). The range of time channels used for fitting was defined by adjusting T1 and T2 values so they included the whole decay curve and excluded the baseline regions. Threshold parameter in the “Decay-Graph” window was set to 50. Next, the decay matrix was generated, using the corresponding command (Calculate>Decay matrix in main menu).
2. Phasor plot was calculated (“Phasor plot” button). Screenshots (Supplementary Fig. S78) were made on this step.
3. The color-coding was set to discrete mode (Options>Color, “Mode discrete”) and the range of Red color was set from 1 to 3000 ps, so all τ became colored Red, Blue and Green channels were left empty.
4. The box “Select cluster” was marked and the first cluster was selected. This selection was based on cluster position on phasor plot obtained for cells with FAST variant, expressed as H2B fuse stained with **HBR-2,5-DM** (Supplementary Fig. S78).
5. The image was exported in .tiff format.
6. The range of Green channel was set from 1 to 3000 ps and Red and Blue channels were left empty.
7. Steps 4-5 were repeated for the second cluster.
8. For Fig. 6C the range of Blue channel was set from 1 to 3000 ps and Red and Green channels were left empty.
9. For Fig. 6C steps 4-5 were repeated for the third cluster.
10. All exported figures were prepared for publication in FIJI 1.53t

Data processing for Fig. 7, and Supplementary Figs. S81, S82, S84, S85, S87, S88, S90, S91, S93, S94

1. Steps 1-2 from protocol for figures Supplementary Figs. S30-77, S80, S83, S86, S89, S92 were made identically.
2. The number of exponential components were raised to two in a “Multiexponential Decay” window. Each component lifetime value was fixed in lifetime value obtained for cells with FAST variant, expressed as H2B fuse stained with **HBR-2,5-DM** (Supplementary Table S9). Next, the decay matrix was generated, using the corresponding command (Calculate>Decay matrix in main menu). For Supplementary S81, S82, S84, S85, S87, S88, S90, S91, S93, S94 screenshots of software windows were made in τ_m_ (default) and τ_i_ (Options>Color, “Coding of”) color-coding modes.
3. Matrices containing values of each exponential component amplitude (A1, A2) in every pixel and intensity were exported as .asc files (File>Export…, “a1[%]”, “a2[%]” and “Pixel Intensities’).
4. Matrices were imported to FIJI 1.53t (File>Import>Text Image…) as 32 bit files.
5. All pixels with values < 0.1% in “a1[%]” and “a2[%]” images were converted to not-a-number (NaN) (Image>Adjust>Threshold…).
6. ‘Pixel Intensity’ image was multiplied by “a1[%]” or “a2[%]” images (Process>Image Calculator…) to generate two images that contained only pixels with τ_1_ or τ_2_ lifetimes. These images were color-coded in a colorblind friendly palette (Image>LUT).

### Statistics and reproducibility

When calculating the dissociation constant values, the averages of three independent measurements were taken. When calculating the quantum yields of fluorescence, the averages of nine independent measurements (for three samples with various concentrations and at three different wavelengths) were taken. When calculating the fluorescence lifetime values in mammalian cells, the averages of sixteen independent measurements were taken. These data are presented as the mean ± standard deviation. For determining the fluorescence lifetime values of complexes in aqueous solution by the TCSPC method, measurements were conducted in duplicate without averaging the obtained values. In the case of FLIM imaging experiments, including multiplexed imaging, we present representative images that reflect patterns obtained in three biological replicates for a number of cells *n* = 12–48. Specific n values are indicated in the caption of the corresponding figures. Exponential fitting was performed using the Weighted least squares (WLS) method, and the goodness of fit was assessed using Pearson’s criterion, as described above in the section Live cell Fluorescence lifetime imaging microscopy processing. FLIM data processing was performed in SPCImage software 8.6 and 8.9 (Becker & Hickl, Germany). Other data processing was performed using Origin 8.6 software.

### Reporting summary

Further information on research design is available in the Nature Portfolio Reporting Summary linked to this article.

### Supplementary information

Supplementary Information

Description of Additional Supplementary Materials

Supplementary Data 1

Reporting Summary

## Data Availability

The authors declare that the data supporting the findings of this study are available within the paper and its Supplementary Information files. Source data of all data presented in graphs within the figures are provided with this paper. Should any raw data files be needed in another format they are available from the corresponding author upon reasonable request. Source data are provided as Supplementary Data 1.
