# Supplementary material for: Fluorescence lifetime multiplexing with fluorogen activating protein FAST variants
Source: Commun Biol. 2024 Jul 2;7:799. doi: 10.1038/s42003-024-06501-1 (PMC11219735; doi:10.1038/s42003-024-06501-1)
Supplement: Supplementary file 1 — Supplementary Information [file 42003_2024_6501_MOESM1_ESM.pdf]

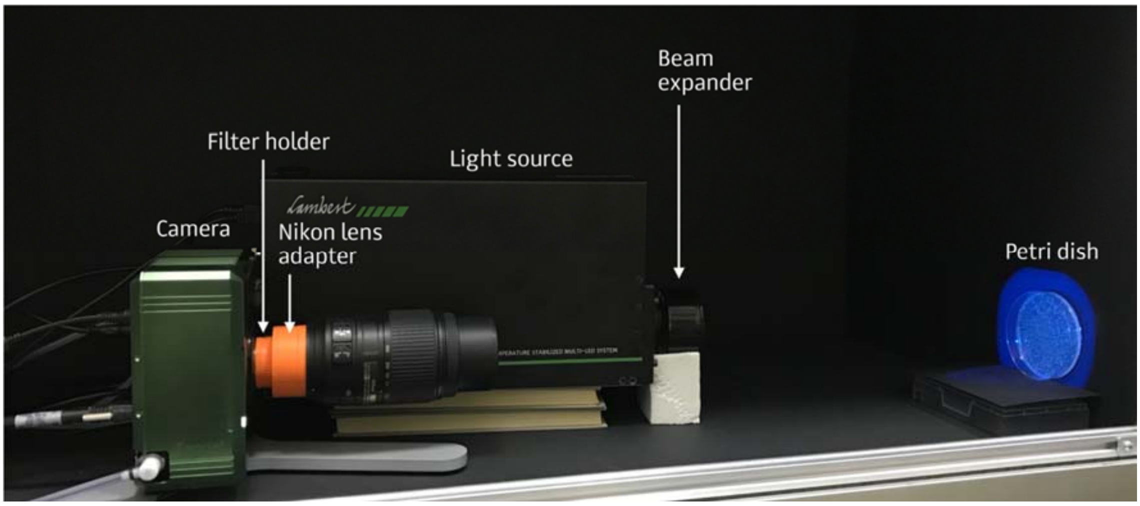

Picture S1. Macro-FLIM setup. 'Camera' and 'light source' are the parts of a Lambert Instruments FLIM Attachment (LIFA) set. Camera is a liquid-cooled siFLIM-optimized Toggel camera; light source is a Multi-LED high-frequency modulated module. 'Filter holder' and 'Nikon lens adapter' are the custom-designed and 3D-printed parts providing an emission filter (Chroma LP 510 nm) positioning, objective lens mounting and a proper focusing of acquired light on the camera's sensor (back focus value). 'Beam expander' is a custom-matched achromatic lens adjusting light beam focusing to a specimen size of approximately 10x10 cm.

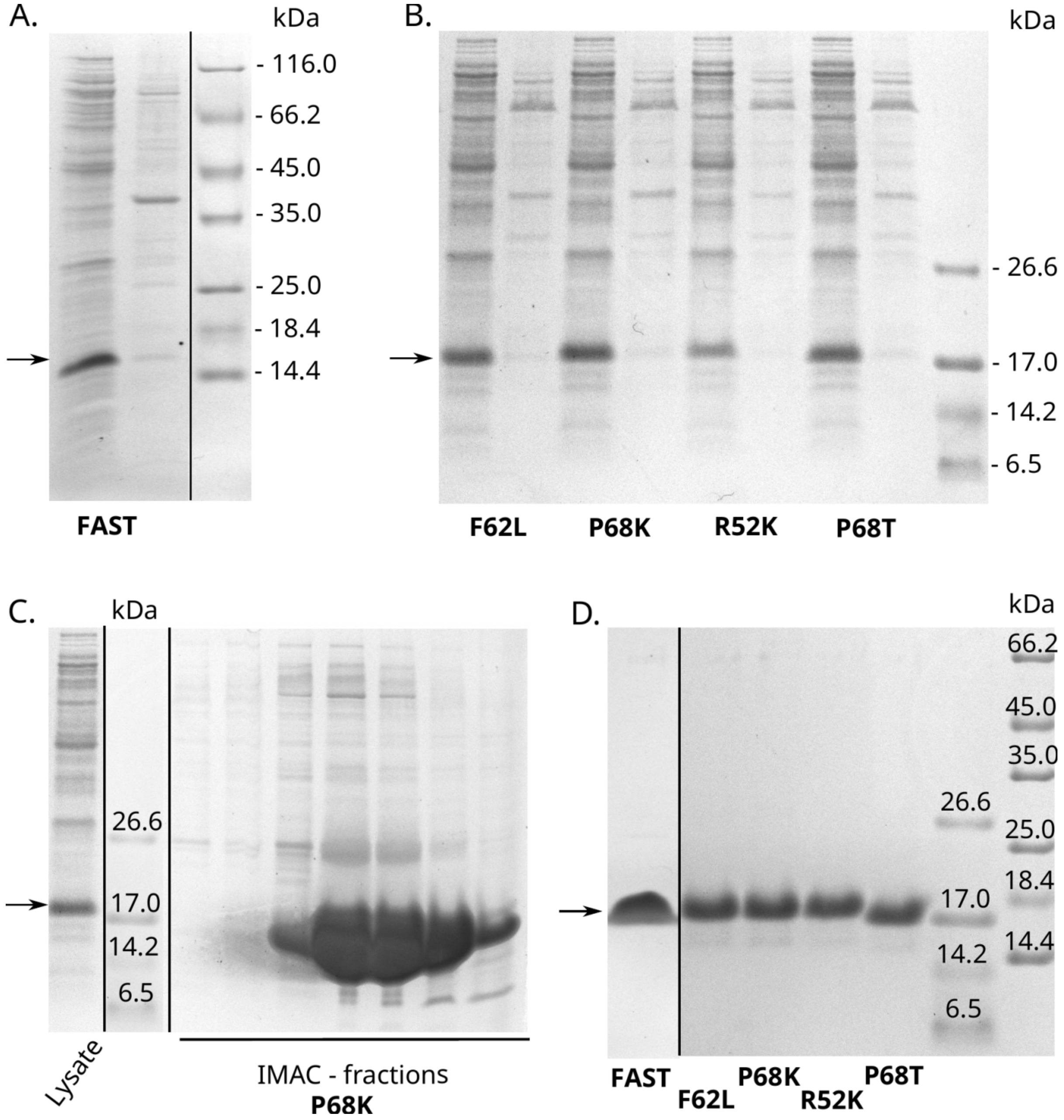

Figure S1. Bacterial expression and purification summary. **A, B** - Cellular accumulation of FAST and variants. Pairs of soluble/insoluble cellular protein 25 μL M9 equivalents are applied to Tris-Glycine (**A**) or Tris-Tricine (**B**) SDS-PAGE**. C** - Summary of purification. FAST-P68K was taken as an example. Cellular lysate in equivalent of 25 μL M9 and main immobilized metal affinity chromatography (IMAC) fractions are shown**, D** - The aliquots of 2.5 μg of purified protein samples are applied on Tris-Glycine (FAST) or Tris-Tricine (variants) SDS-PAGE. The target protein bands are indicated by arrows. Protein molecular weight markers used: (116.0, 66.2, 45.0, 35.0, 25.0, 18.4, 14.4 kDa) or (26.6, 17.0, 14.2, 6.5 kDa). Molecular weight of the wild type of FAST is 14.7 kDa.

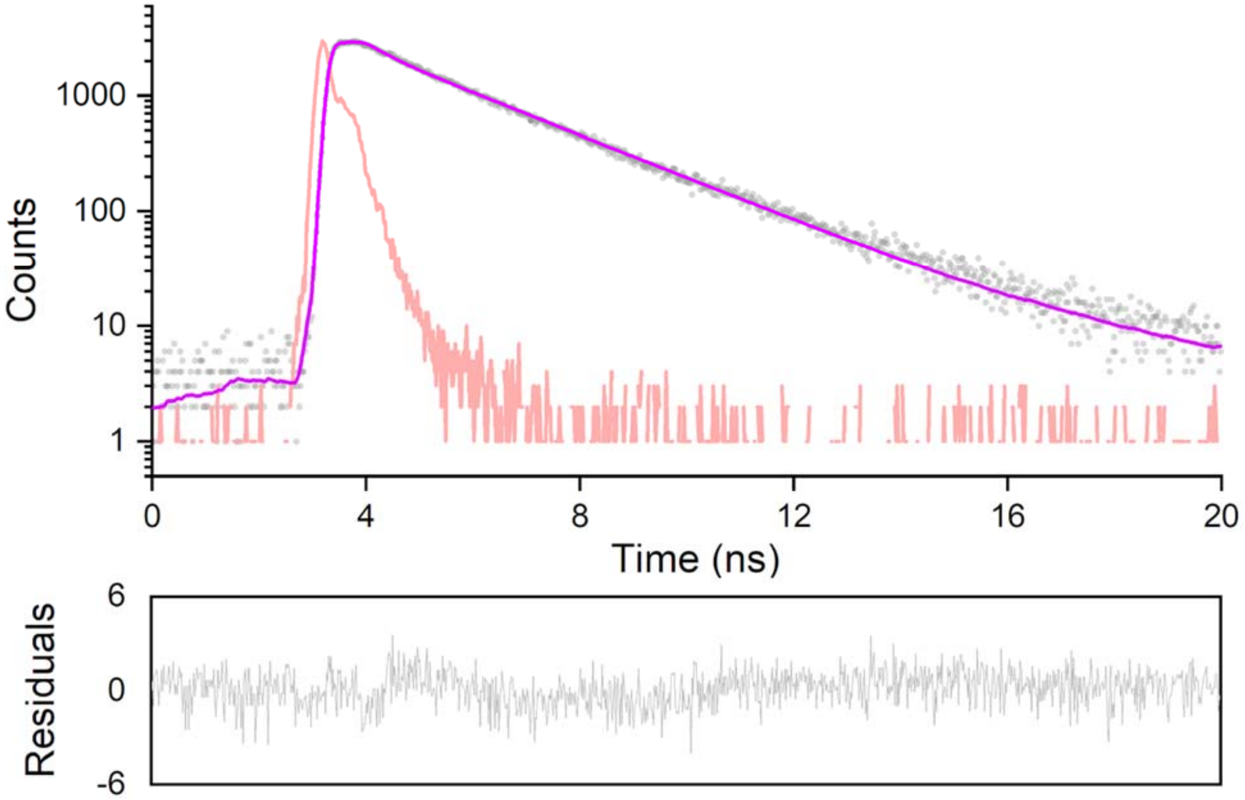

Figure S2. Fluorescence decay kinetics of the **25DOM-HBI-2T** chromophore bound by the R52K FAST variant. Gray dots represent experimental decay data (photon arrivals), violet line shows exponential fit of the data, red curve denotes instrument response function (IRF). Residuals of fitting results are shown below.

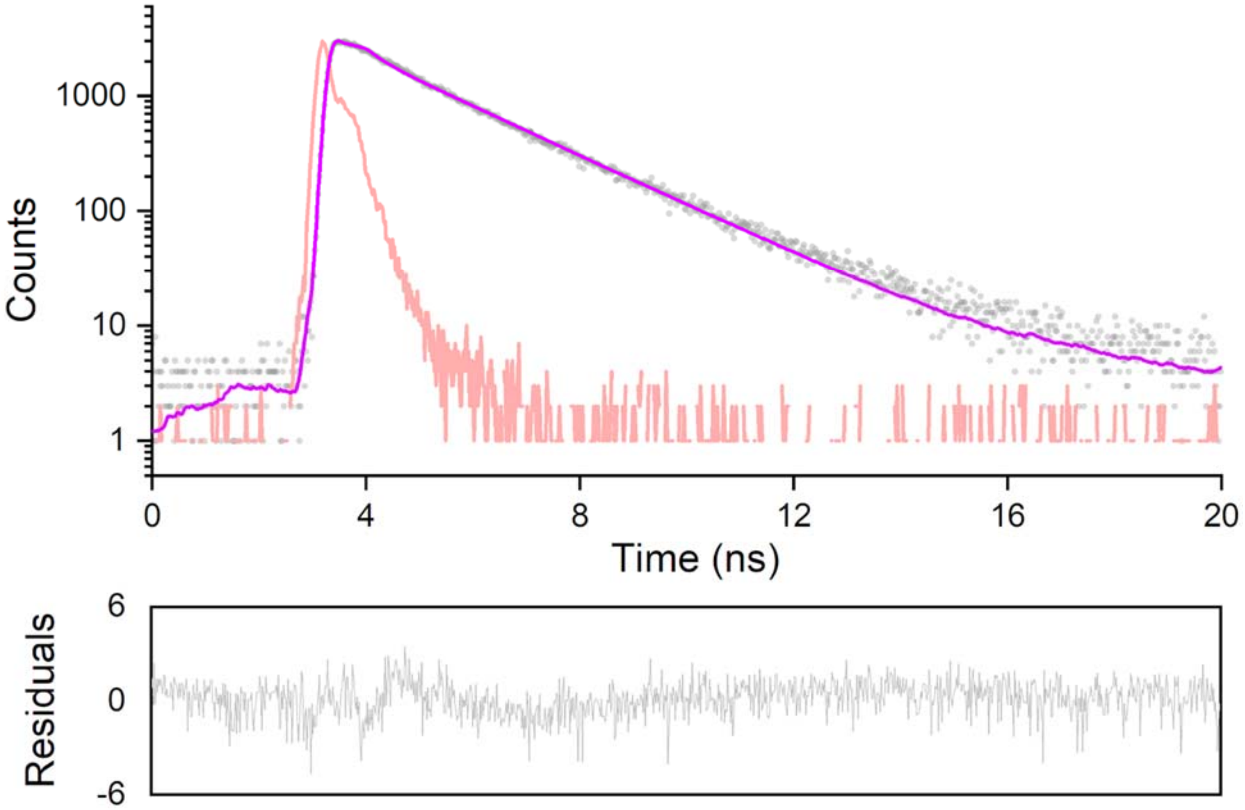

Figure S3. Fluorescence decay kinetics of the **25DOM-HBI-2T** chromophore bound by the F62L FAST variant. Gray dots represent experimental decay data (photon arrivals), violet line shows exponential fit of the data, red curve denotes instrument response function (IRF). Residuals of fitting results are shown below.

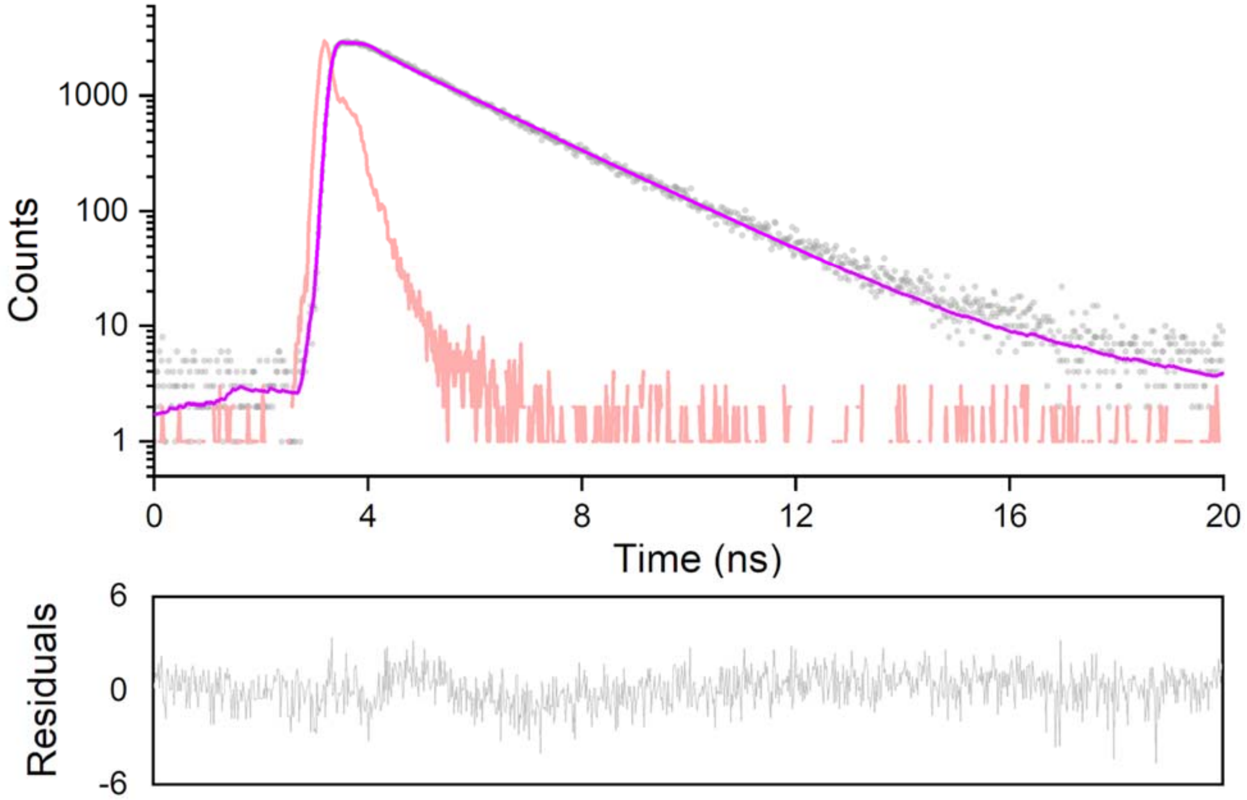

Figure S4. Fluorescence decay kinetics of the **25DOM-HBI-2T** chromophore bound by the P68K FAST variant. Gray dots represent experimental decay data (photon arrivals), violet line shows exponential fit of the data, red curve denotes instrument response function (IRF). Residuals of fitting results are shown below.

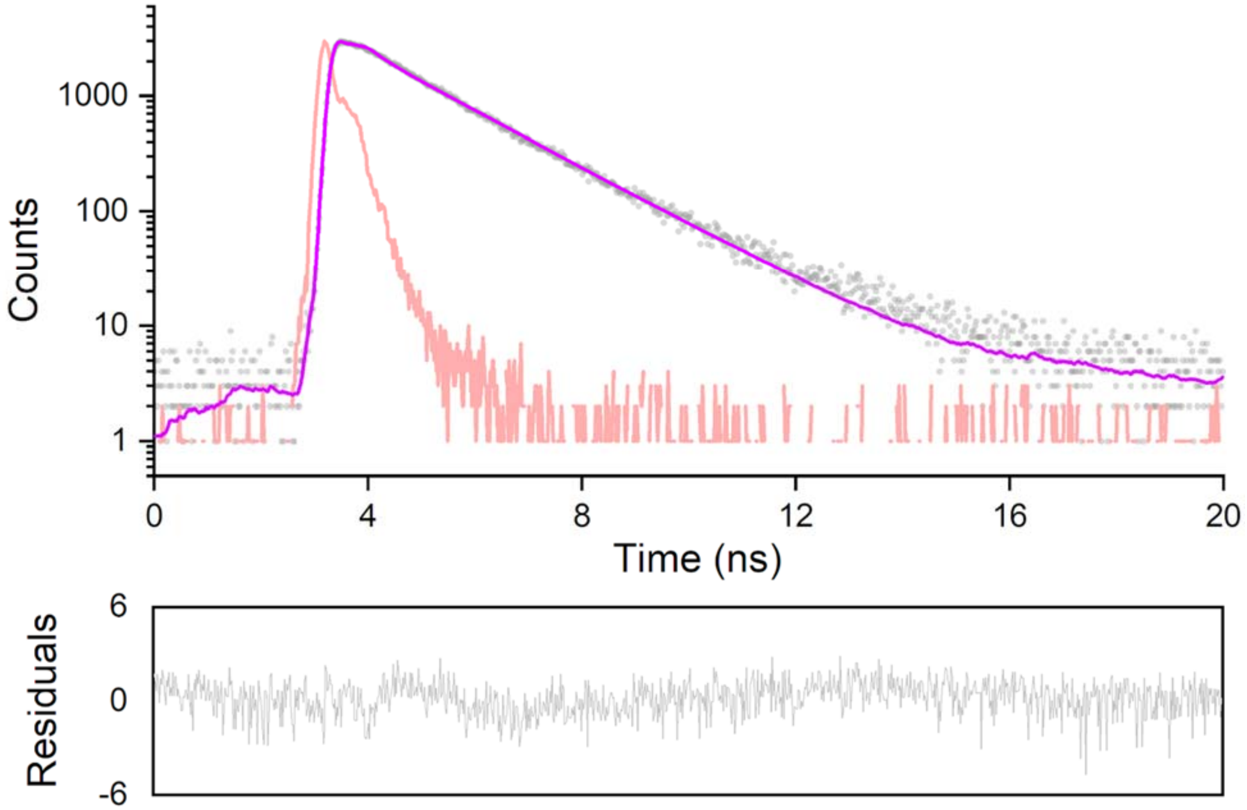

Figure S5. Fluorescence decay kinetics of the **25DOM-HBI-2T** chromophore bound by the P68T FAST variant. Gray dots represent experimental decay data (photon arrivals), violet line shows exponential fit of the data, red curve denotes instrument response function (IRF). Residuals of fitting results are shown below.

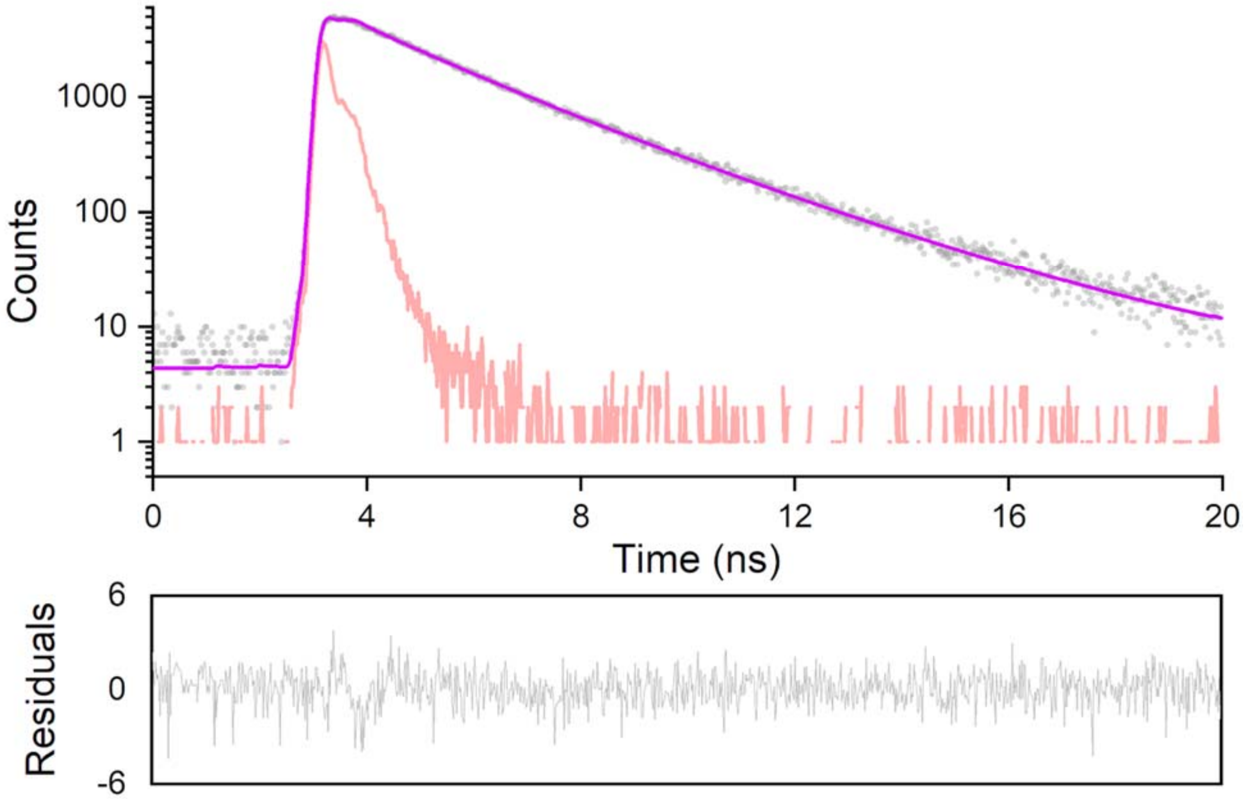

Figure S6. Fluorescence decay kinetics of the **25DOM-HBI-2T** chromophore bound by the wild type FAST variant. Gray dots represent experimental decay data (photon arrivals), violet line shows exponential fit of the data, red curve denotes instrument response function (IRF). Residuals of fitting results are shown below.

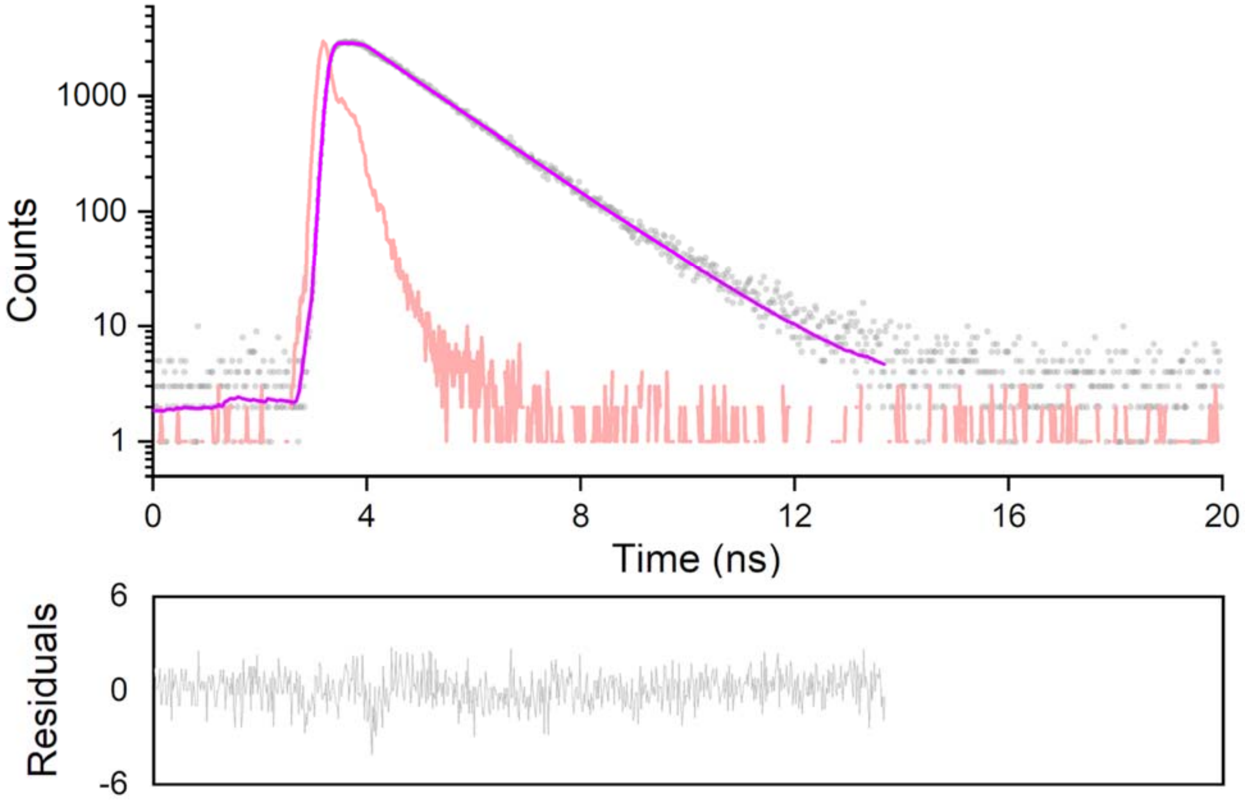

Figure S7. Fluorescence decay kinetics of the **HMBR** chromophore bound by the R52K FAST variant. Gray dots represent experimental decay data (photon arrivals), violet line shows exponential fit of the data, red curve denotes instrument response function (IRF). Residuals of fitting results are shown below.

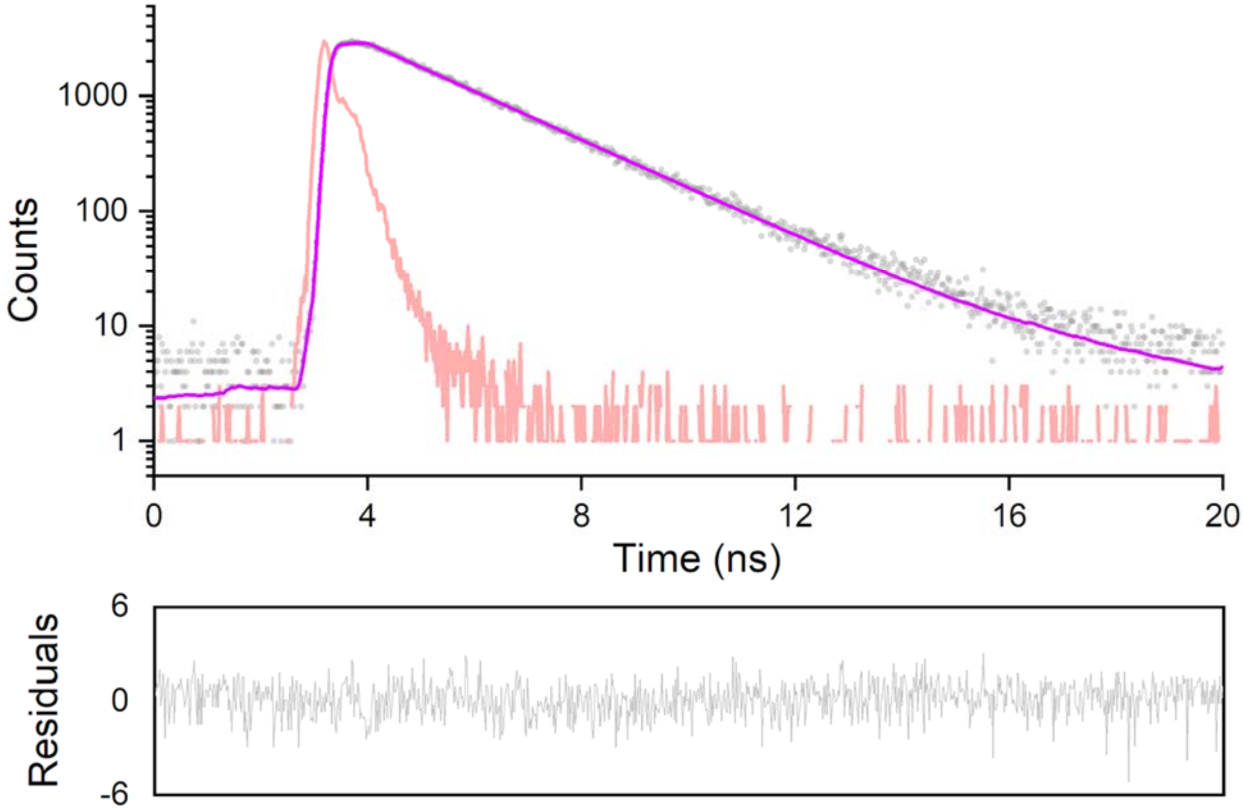

Figure S8. Fluorescence decay kinetics of the **HMBR** chromophore bound by the P62L FAST variant. Gray dots represent experimental decay data (photon arrivals), violet line shows exponential fit of the data, red curve denotes instrument response function (IRF). Residuals of fitting results are shown below.

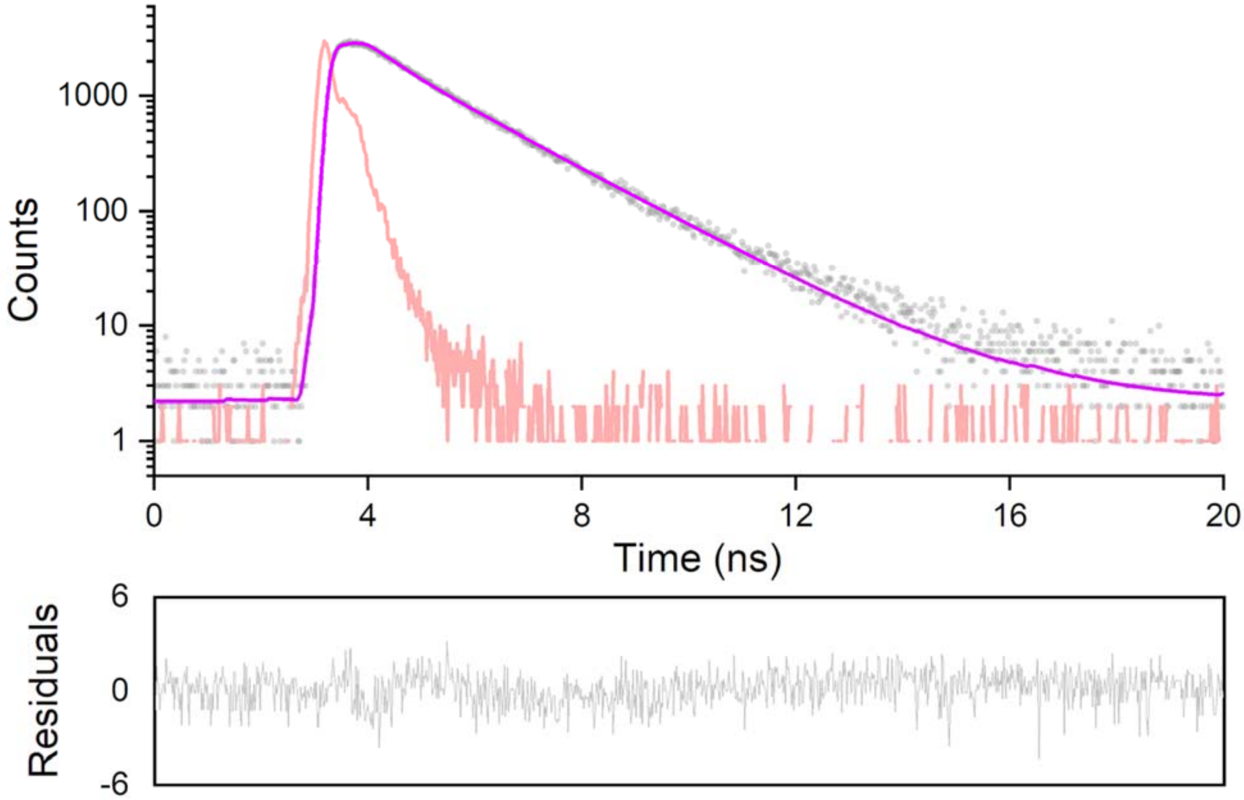

Figure S9. Fluorescence decay kinetics of the **HMBR** chromophore bound by the P68K FAST variant. Gray dots represent experimental decay data (photon arrivals), violet line shows exponential fit of the data, red curve denotes instrument response function (IRF). Residuals of fitting results are shown below.

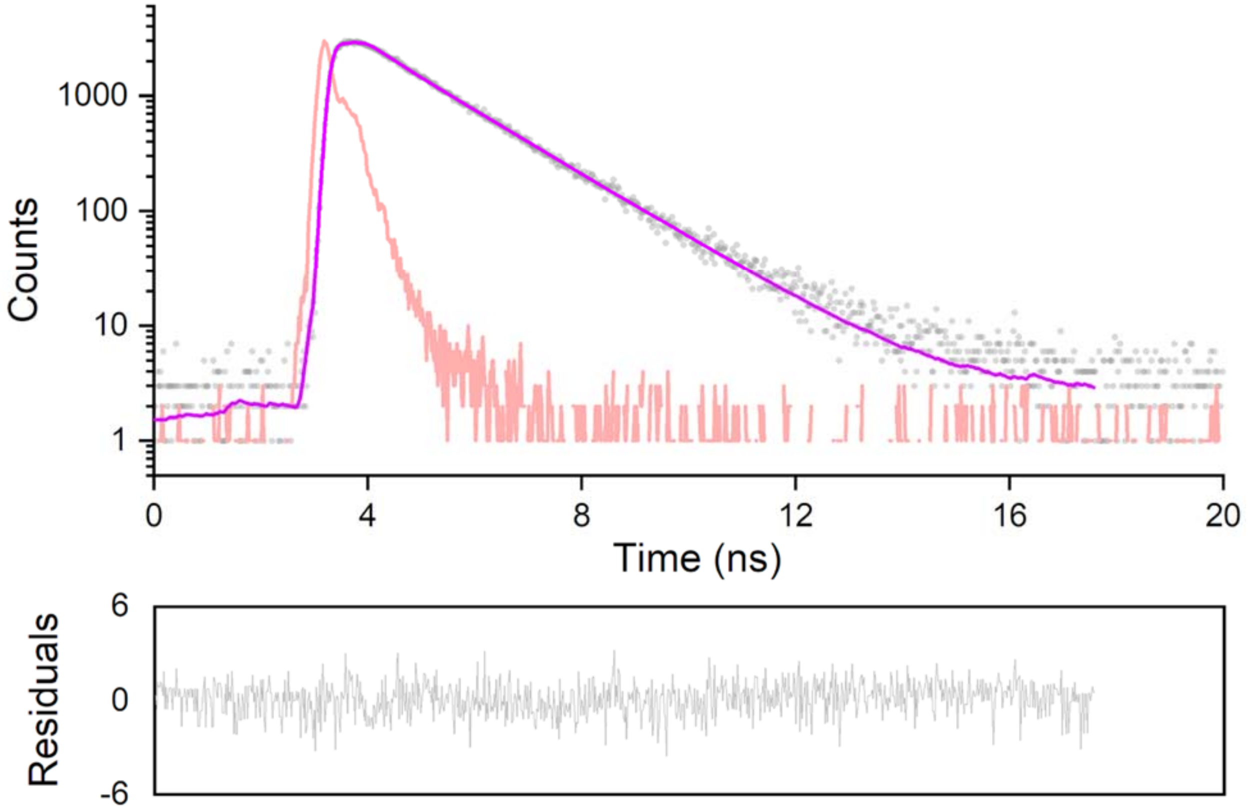

Figure S10. Fluorescence decay kinetics of the **HMBR** chromophore bound by the P68T FAST variant. Gray dots represent experimental decay data (photon arrivals), violet line shows exponential fit of the data, red curve denotes instrument response function (IRF). Residuals of fitting results are shown below.

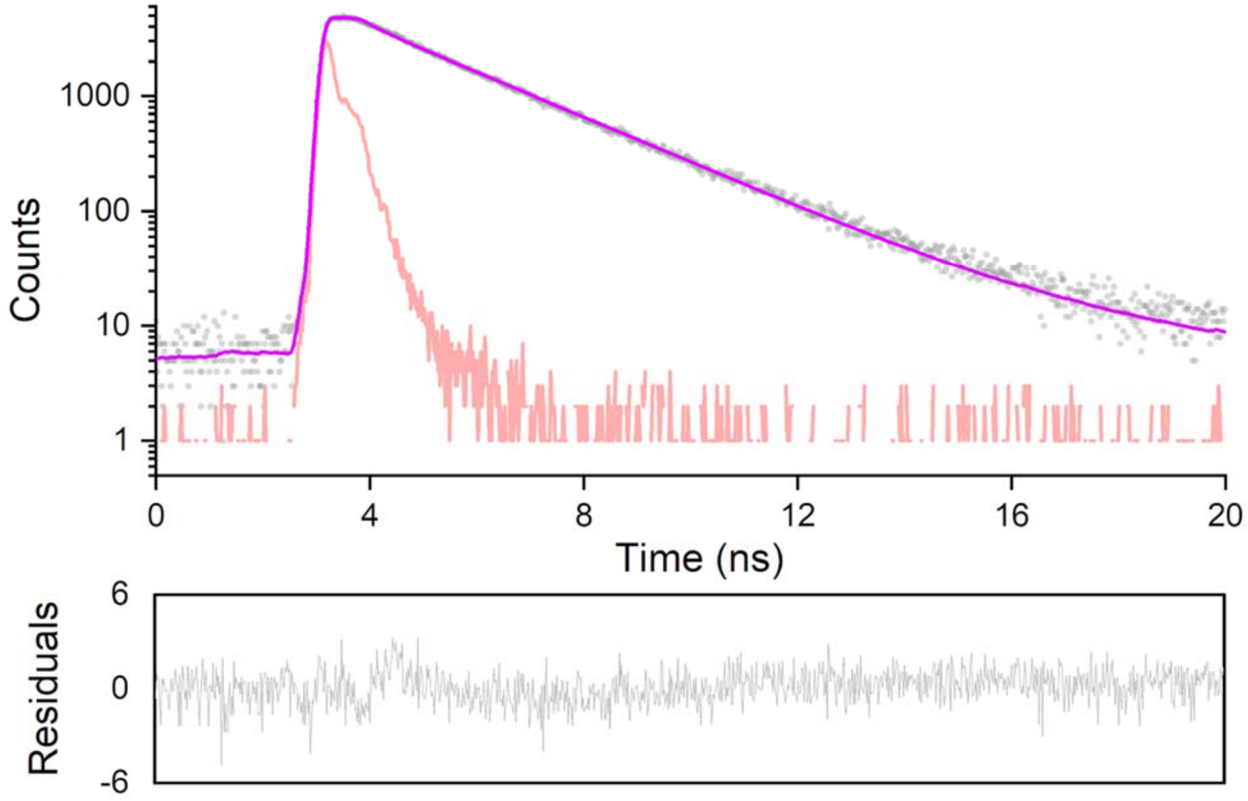

Figure S11. Fluorescence decay kinetics of the **HMBR** chromophore bound by the wild type FAST variant. Gray dots represent experimental decay data (photon arrivals), violet line shows exponential fit of the data, red curve denotes instrument response function (IRF). Residuals of fitting results are shown below.

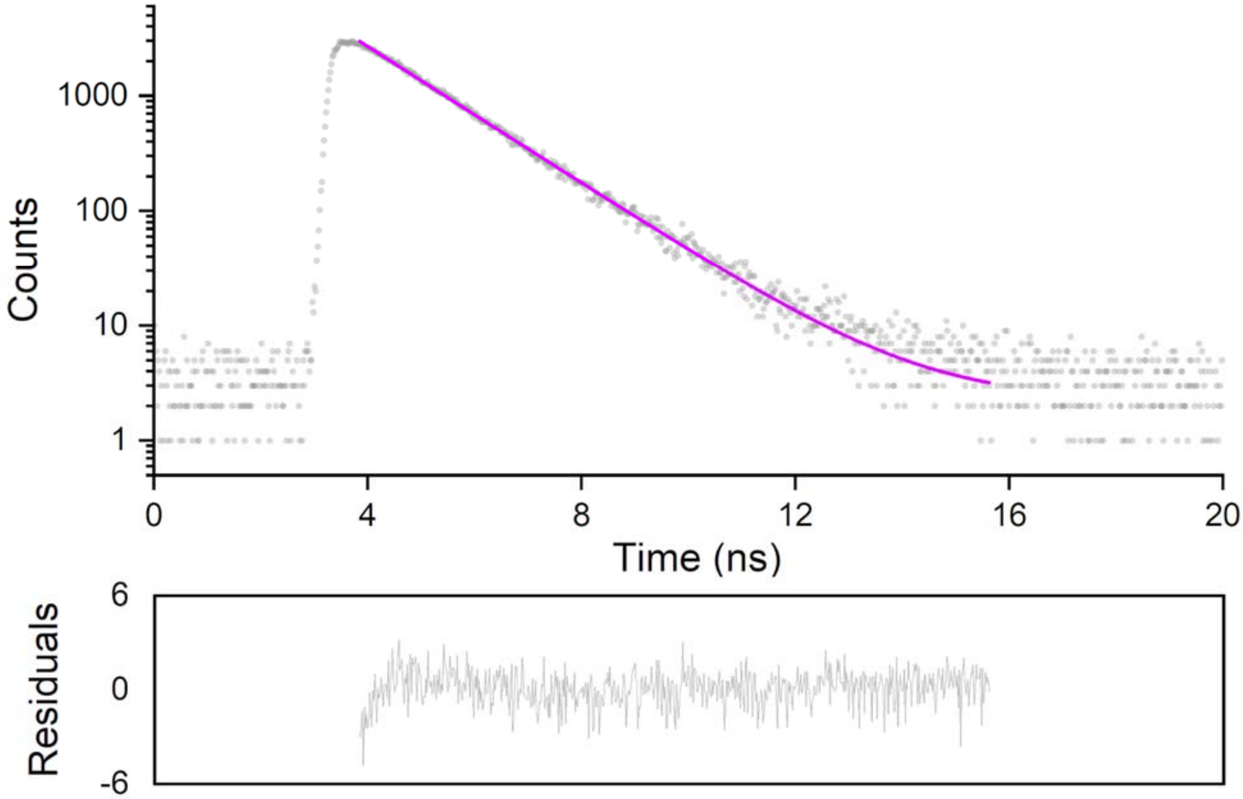

Figure S12. Fluorescence decay kinetics of the **HBR-2,5-DM** chromophore bound by the R52K FAST variant. Gray dots represent experimental decay data (photon arrivals), violet line shows exponential fit of the data. Residuals of fitting results are shown below.

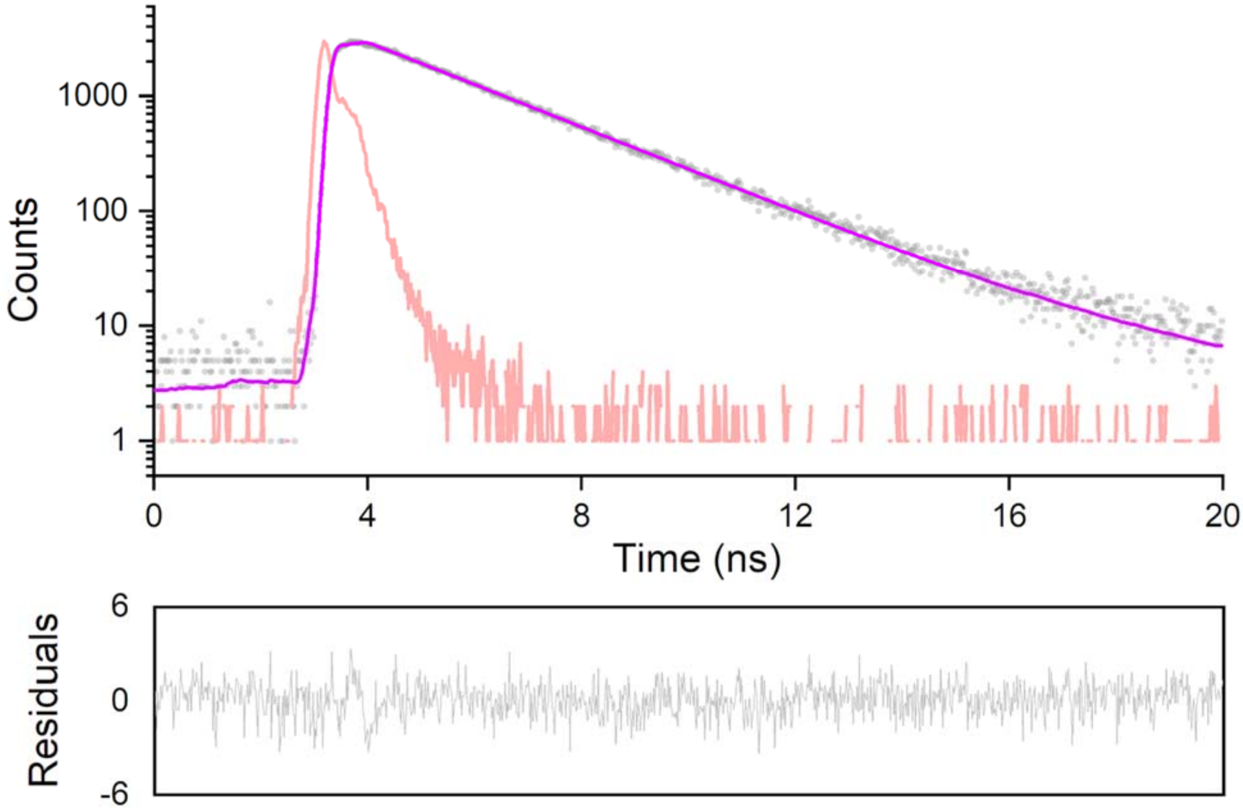

Figure S13. Fluorescence decay kinetics of the **HBR-2,5-DM** chromophore bound by the F62L FAST variant. Gray dots represent experimental decay data (photon arrivals), violet line shows exponential fit of the data, red curve denotes instrument response function (IRF). Residuals of fitting results are shown below.

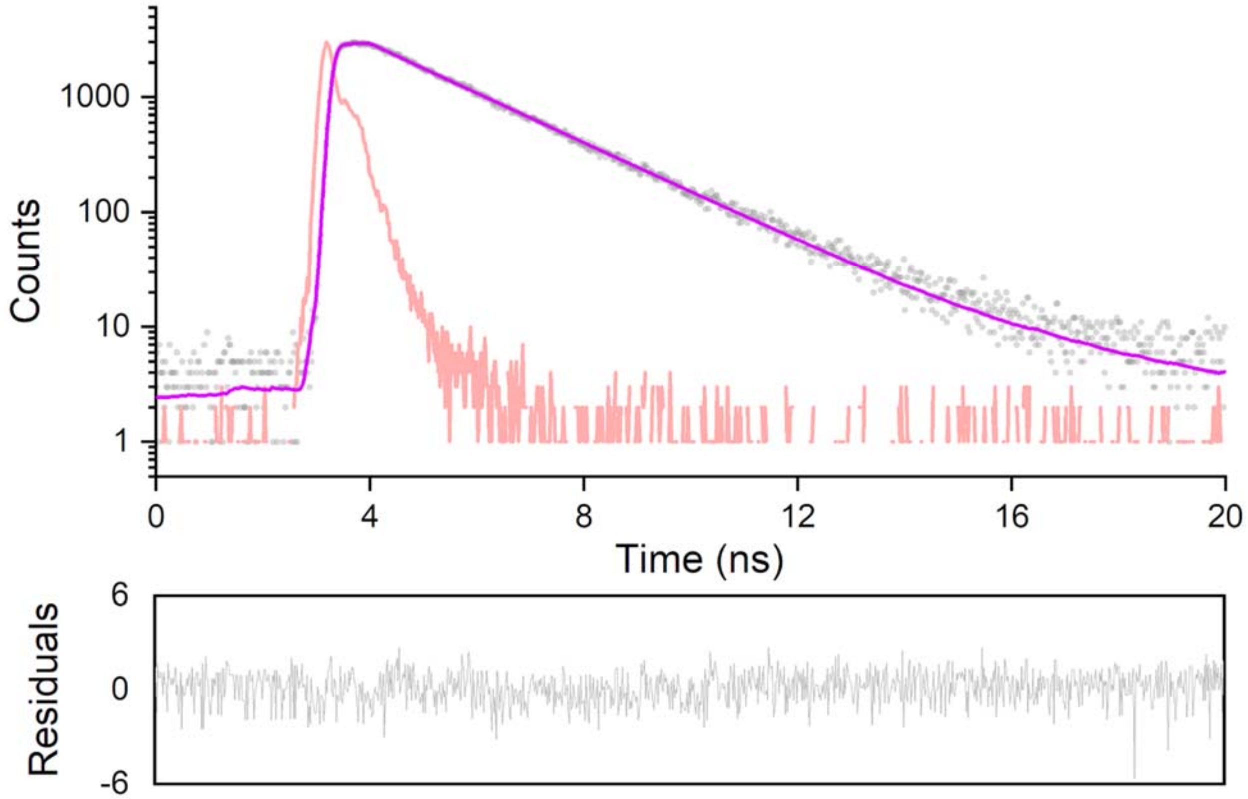

Figure S14. Fluorescence decay kinetics of the **HBR-2,5-DM** chromophore bound by the P68K FAST variant. Gray dots represent experimental decay data (photon arrivals), violet line shows exponential fit of the data, red curve denotes instrument response function (IRF). Residuals of fitting results are shown below.

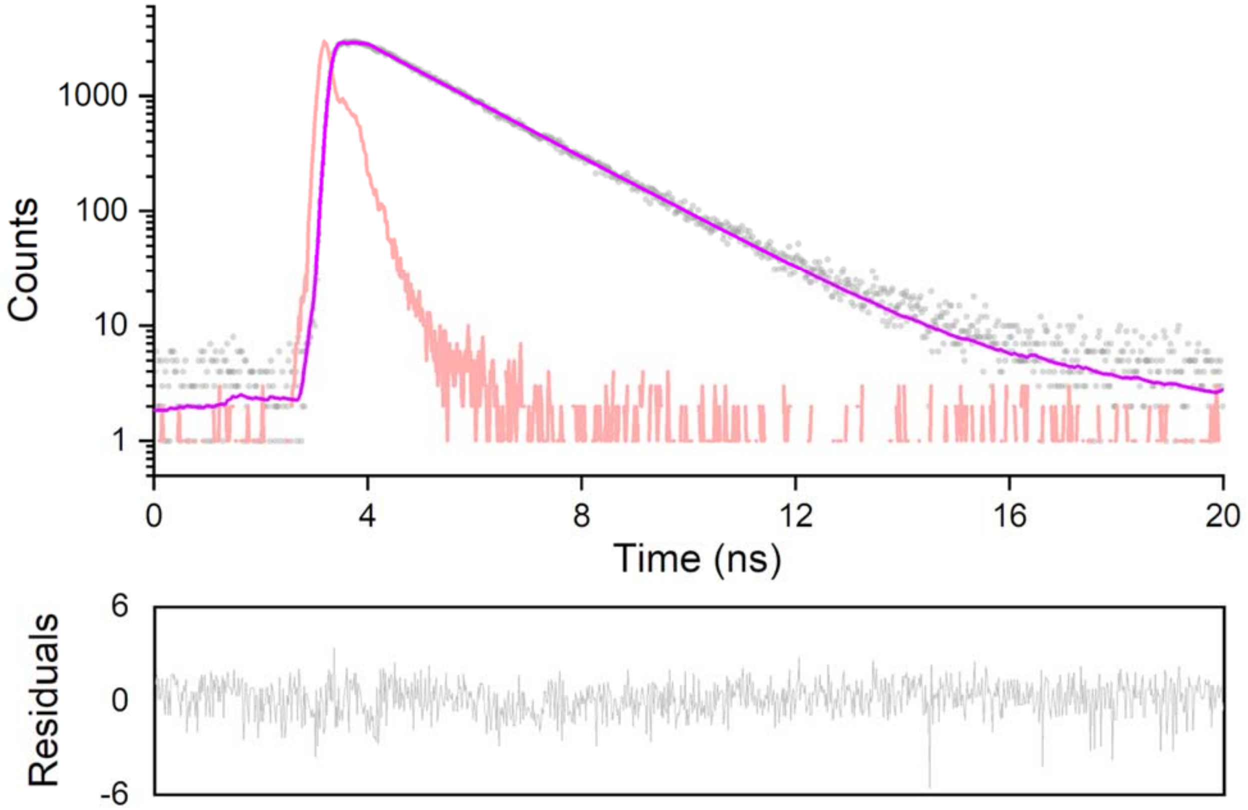

Figure S15. Fluorescence decay kinetics of the **HBR-2,5-DM** chromophore bound by the P68T FAST variant. Gray dots represent experimental decay data (photon arrivals), violet line shows exponential fit of the data, red curve denotes instrument response function (IRF). Residuals of fitting results are shown below.

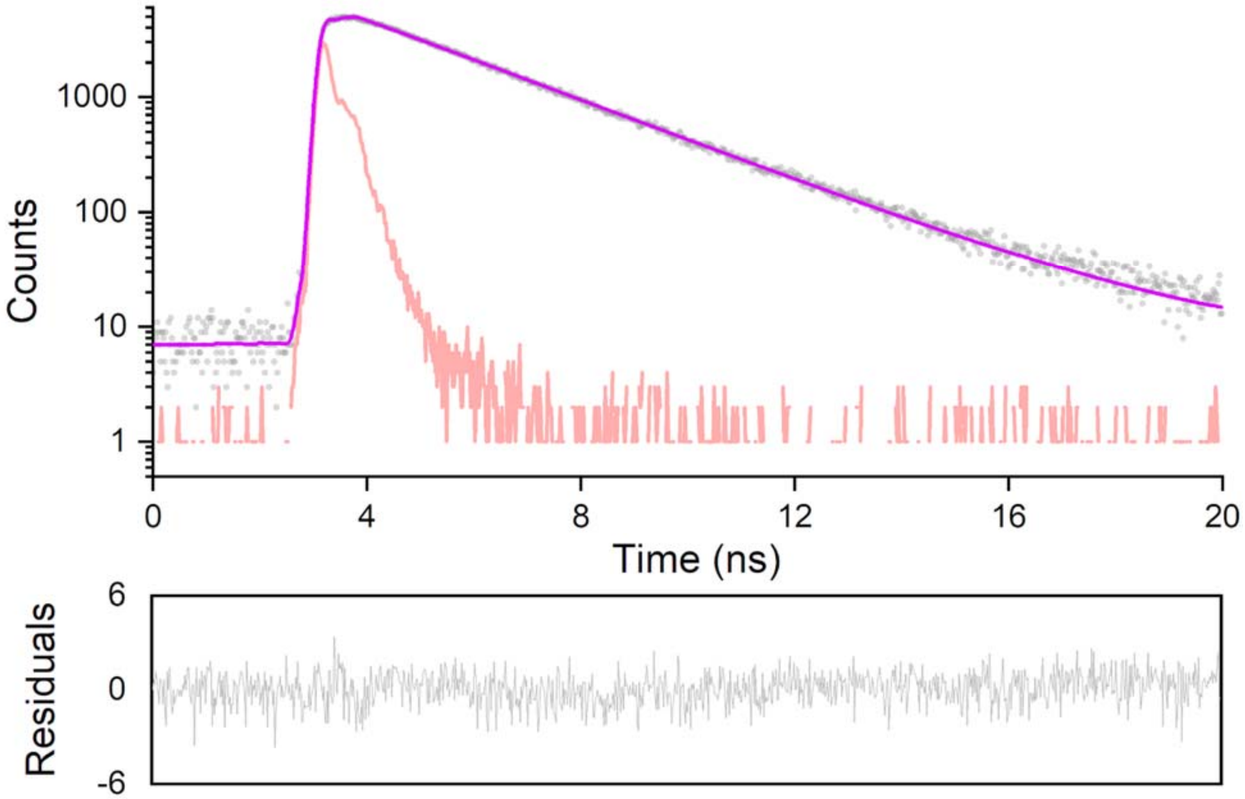

Figure S16. Fluorescence decay kinetics of the **HBR-2,5-DM** chromophore bound by the wild type FAST variant. Gray dots represent experimental decay data (photon arrivals), violet line shows exponential fit of the data, red curve denotes instrument response function (IRF). Residuals of fitting results are shown below.

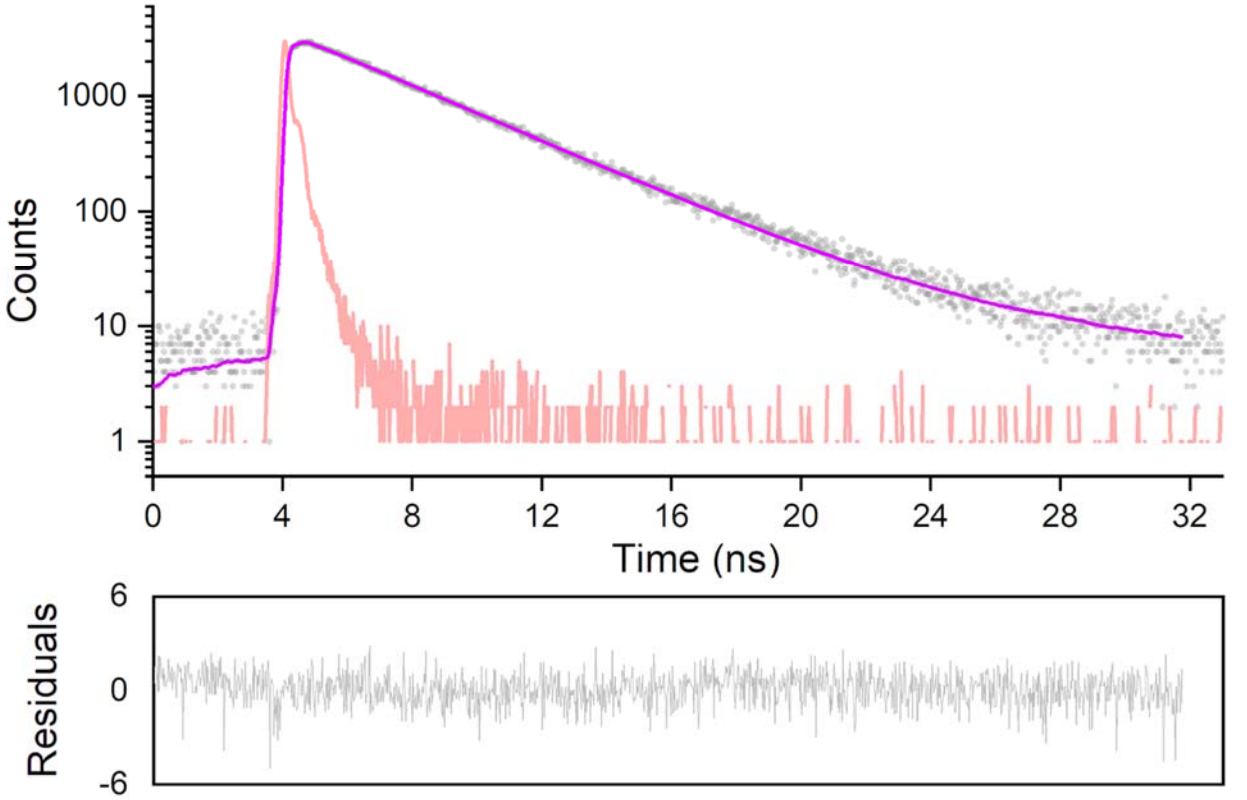

Figure S17. Fluorescence decay kinetics of the **HBR-DOM2** chromophore bound by the R52K FAST variant. Gray dots represent experimental decay data (photon arrivals), violet line shows exponential fit of the data, red curve denotes instrument response function (IRF). Residuals of fitting results are shown below.

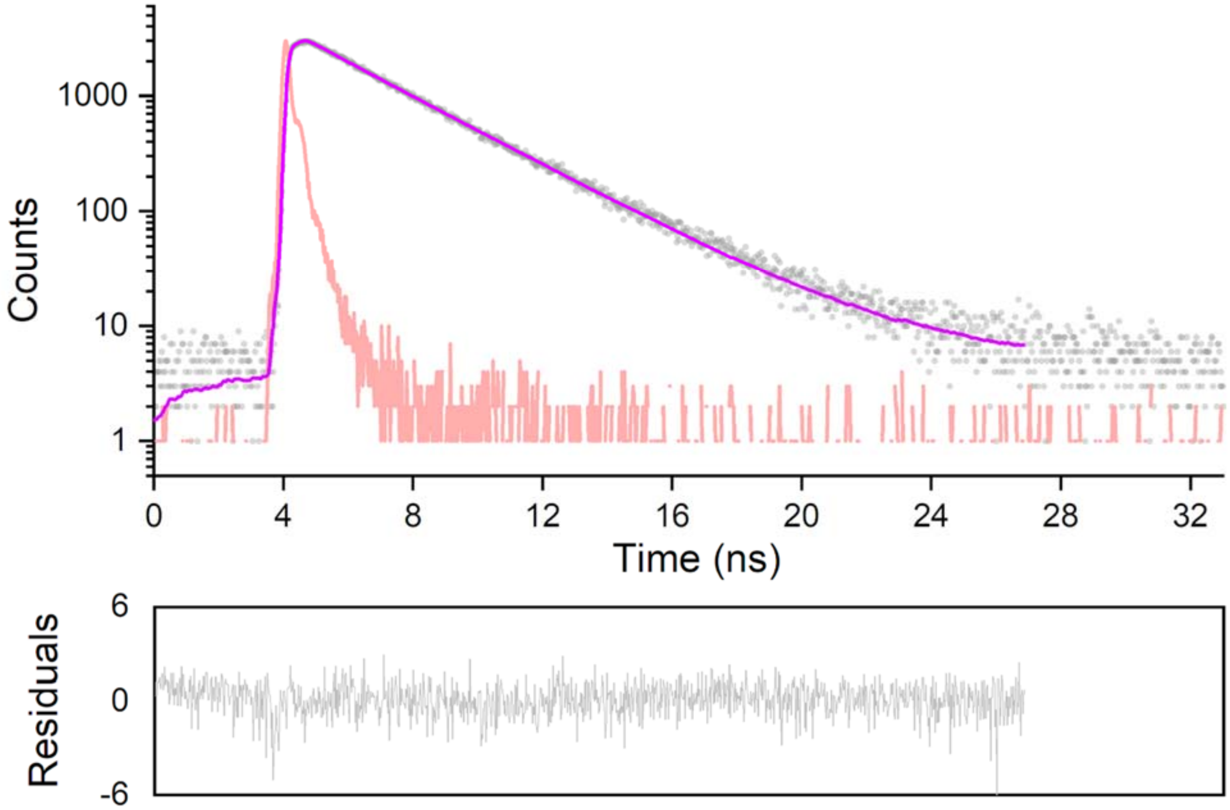

Figure S18. Fluorescence decay kinetics of the **HBR-DOM2** chromophore bound by the F62L FAST variant. Gray dots represent experimental decay data (photon arrivals), violet line shows exponential fit of the data, red curve denotes instrument response function (IRF). Residuals of fitting results are shown below.

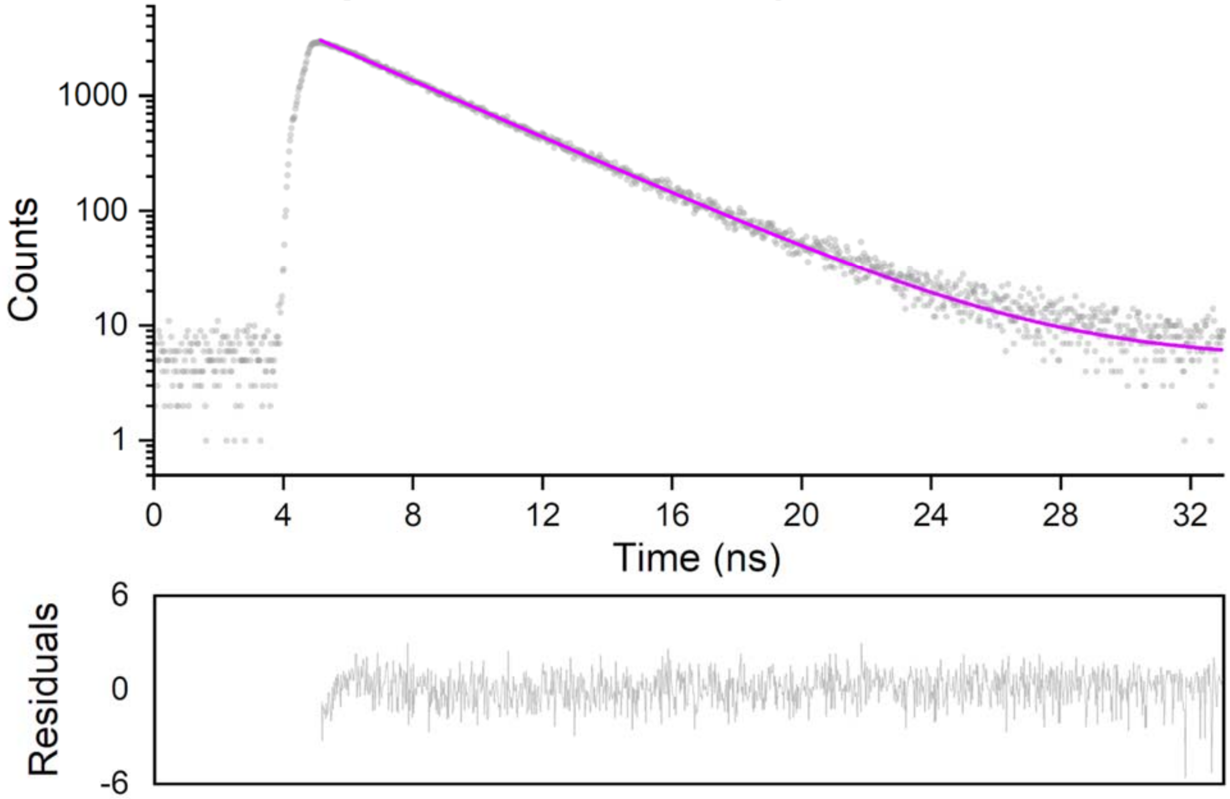

Figure S19. Fluorescence decay kinetics of the **HBR-DOM2** chromophore bound by the P68K FAST variant. Gray dots represent experimental decay data (photon arrivals), violet line shows exponential fit of the data. Residuals of fitting results are shown below.

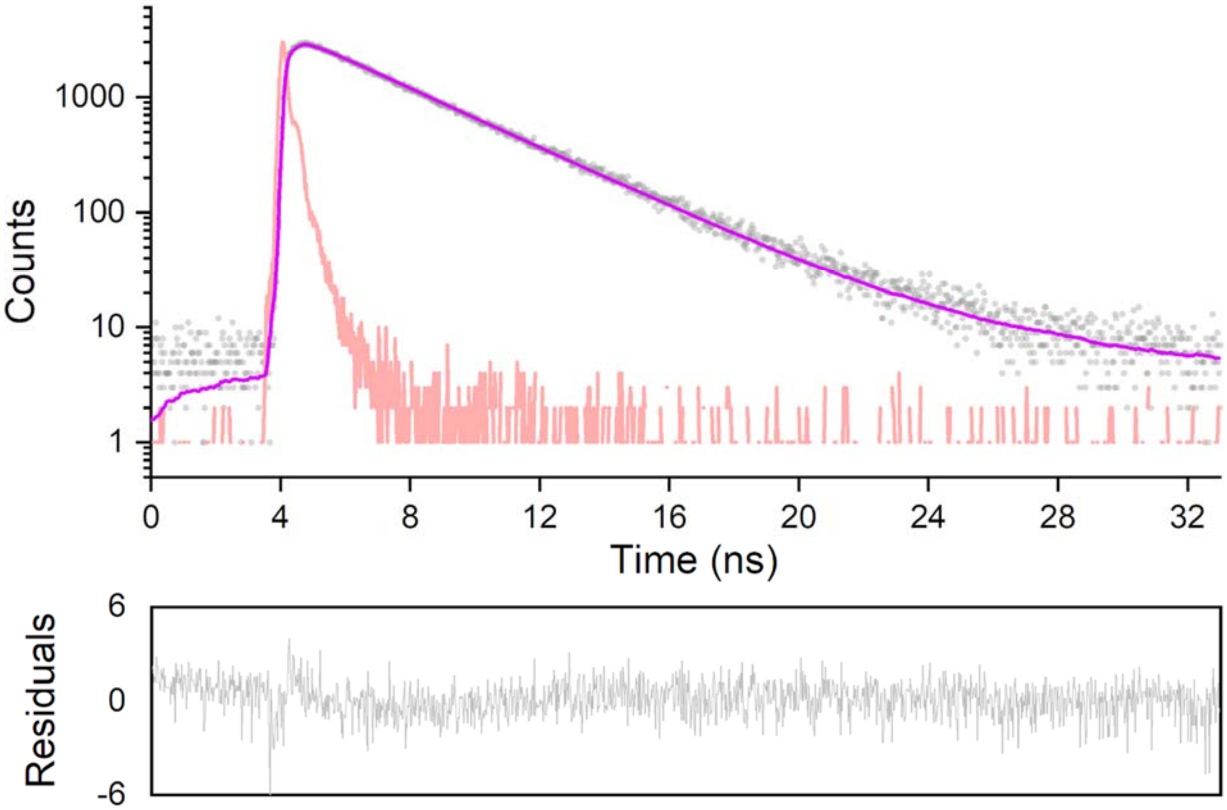

Figure S20. Fluorescence decay kinetics of the **HBR-DOM2** chromophore bound by the P68T FAST variant. Gray dots represent experimental decay data (photon arrivals), violet line shows exponential fit of the data, red curve denotes instrument response function (IRF). Residuals of fitting results are shown below.

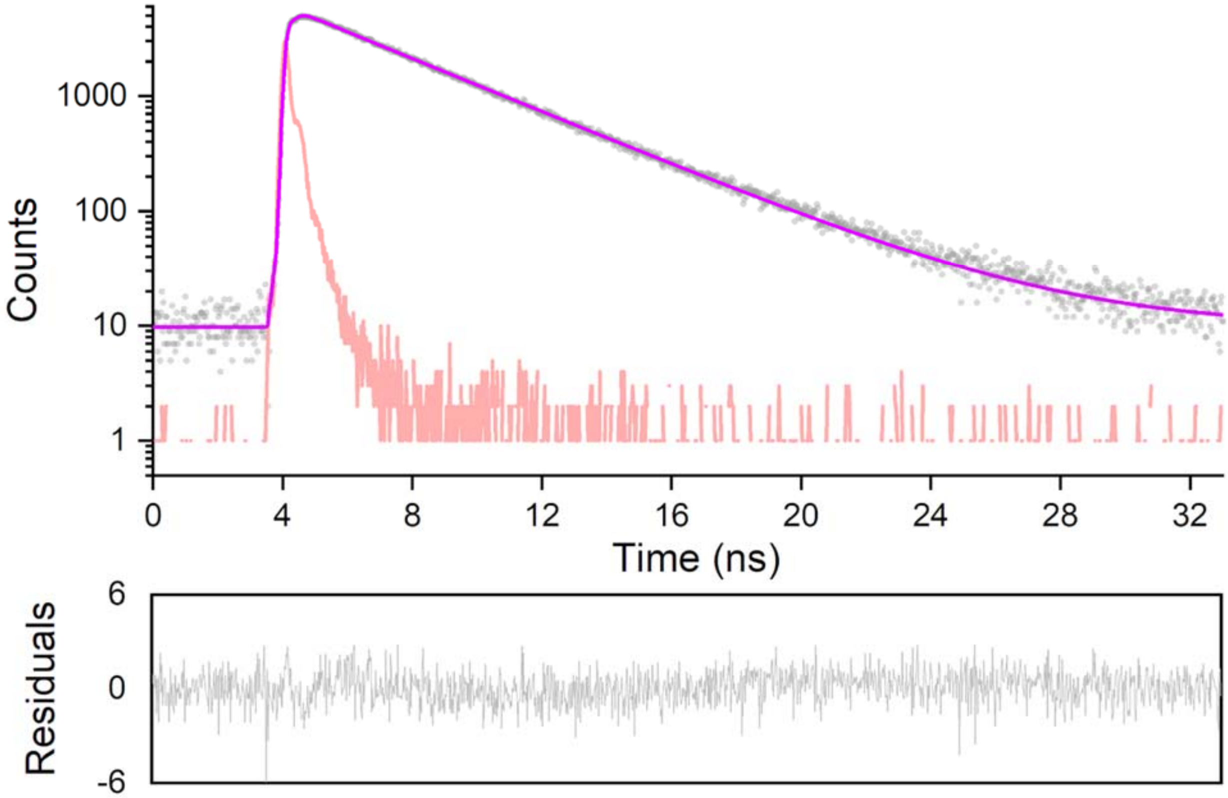

Figure S21. Fluorescence decay kinetics of the **HBR-DOM2** chromophore bound by the wild type FAST variant. Gray dots represent experimental decay data (photon arrivals), violet line shows exponential fit of the data, red curve denotes instrument response function (IRF). Residuals of fitting results are shown below.

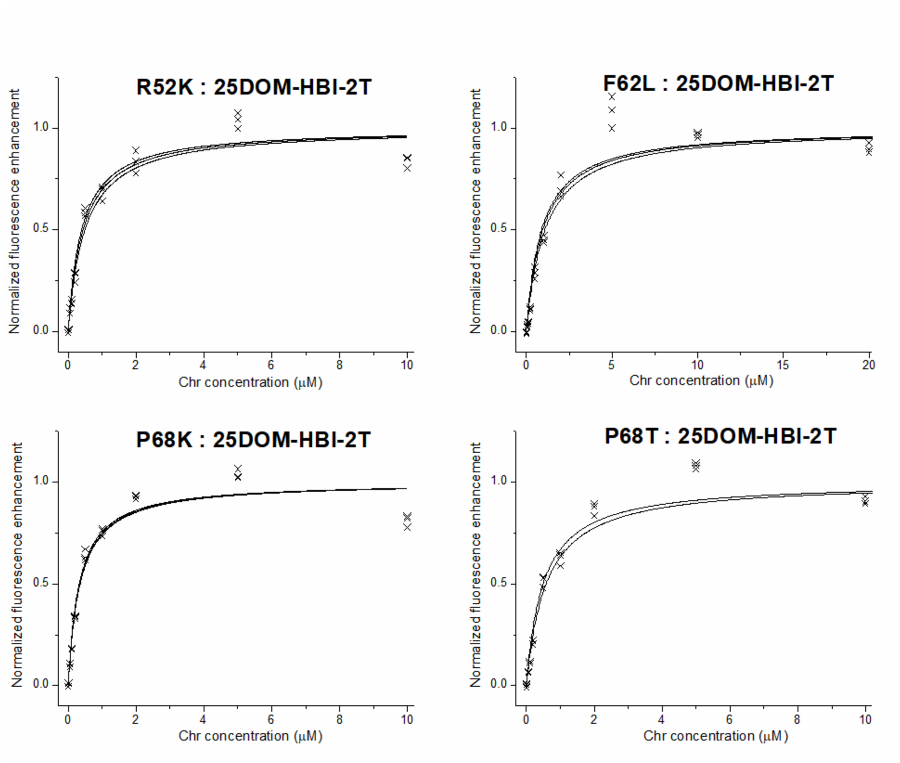

Figure S22. Titration curves observed for **25DOM-HBI-2T** complexes with FAST variants.

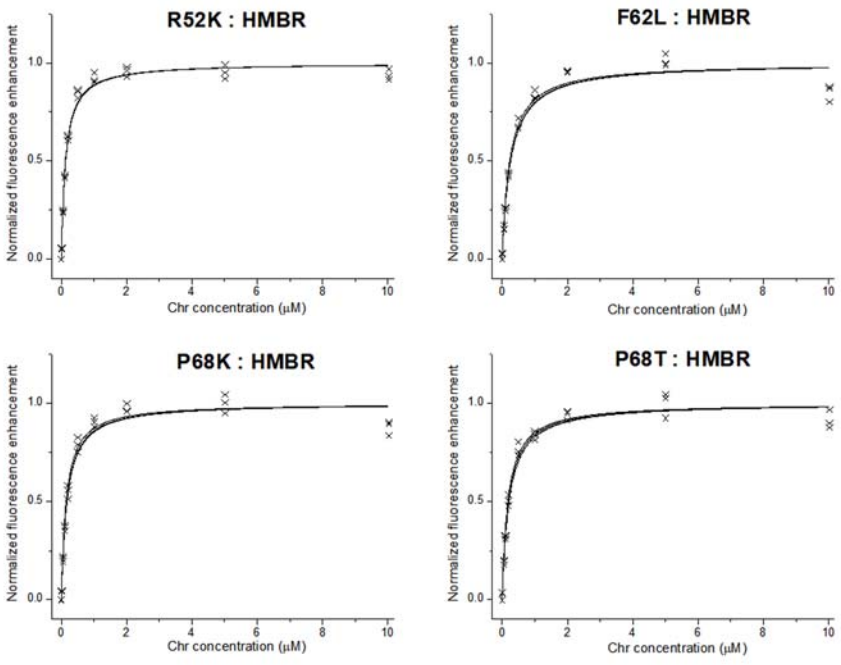

Figure S23. Titration curves observed for **HMBR** complexes with FAST variants.

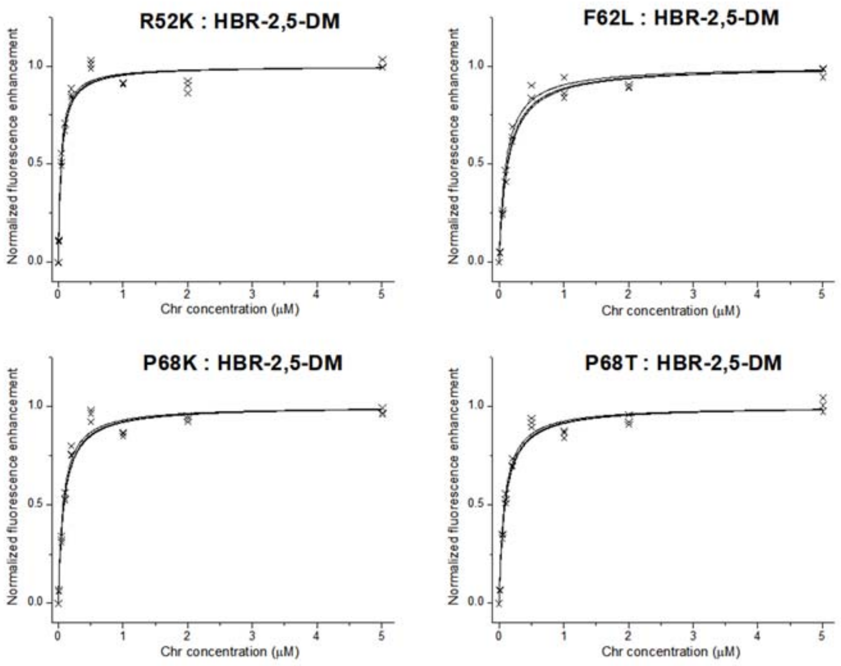

Figure S24. Titration curves observed for **HBR-2,5-DM** complexes with FAST variants.

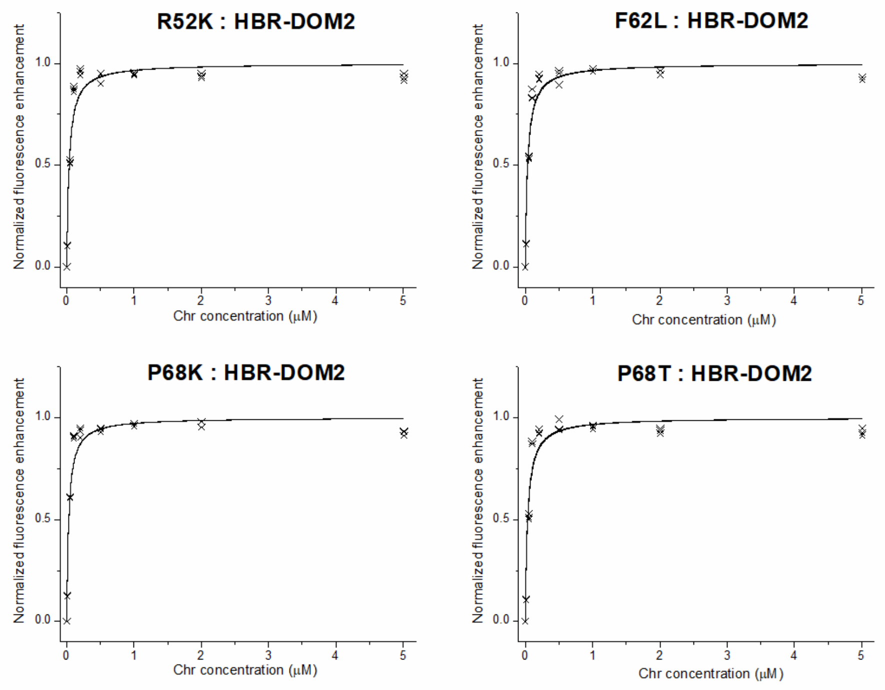

Figure S25. Titration curves observed for **HBR-DOM2** complexes with FAST variants.

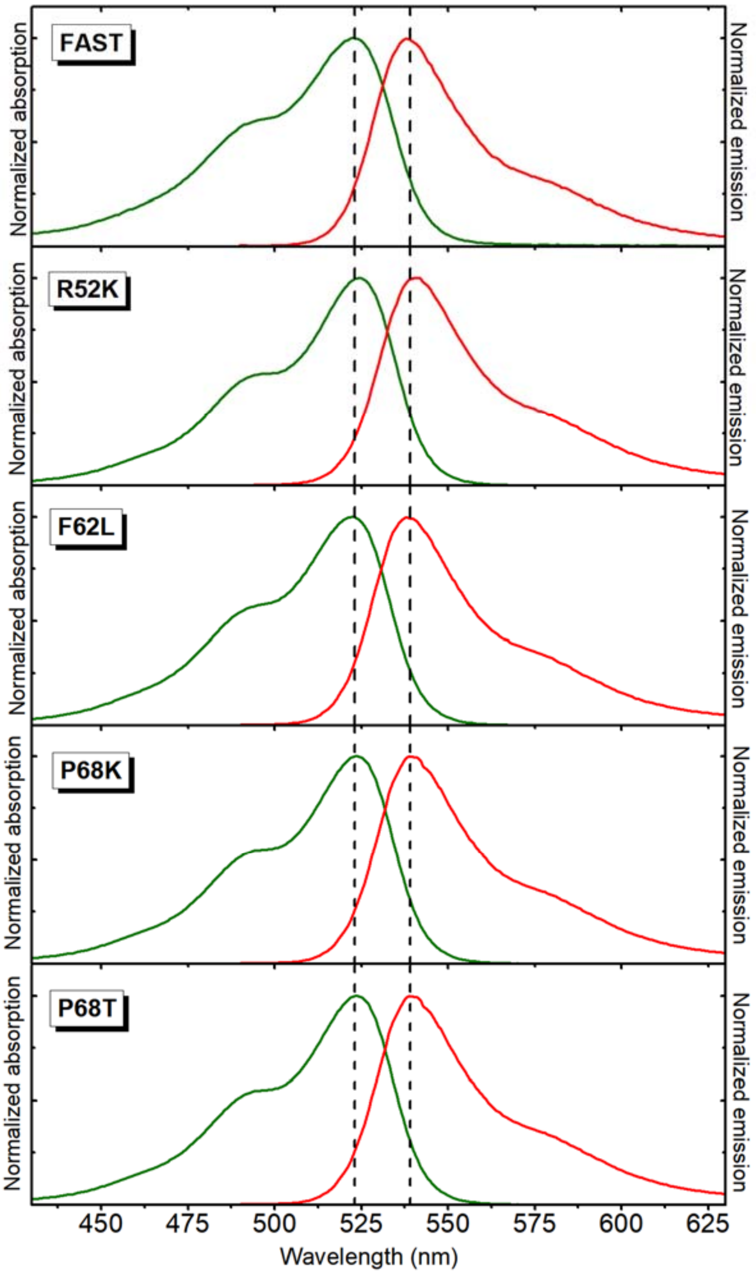

Figure S26. Fluorescence (red) and absorption (green) spectra of **25DOM-HBI-2T** complexes with FAST variants.

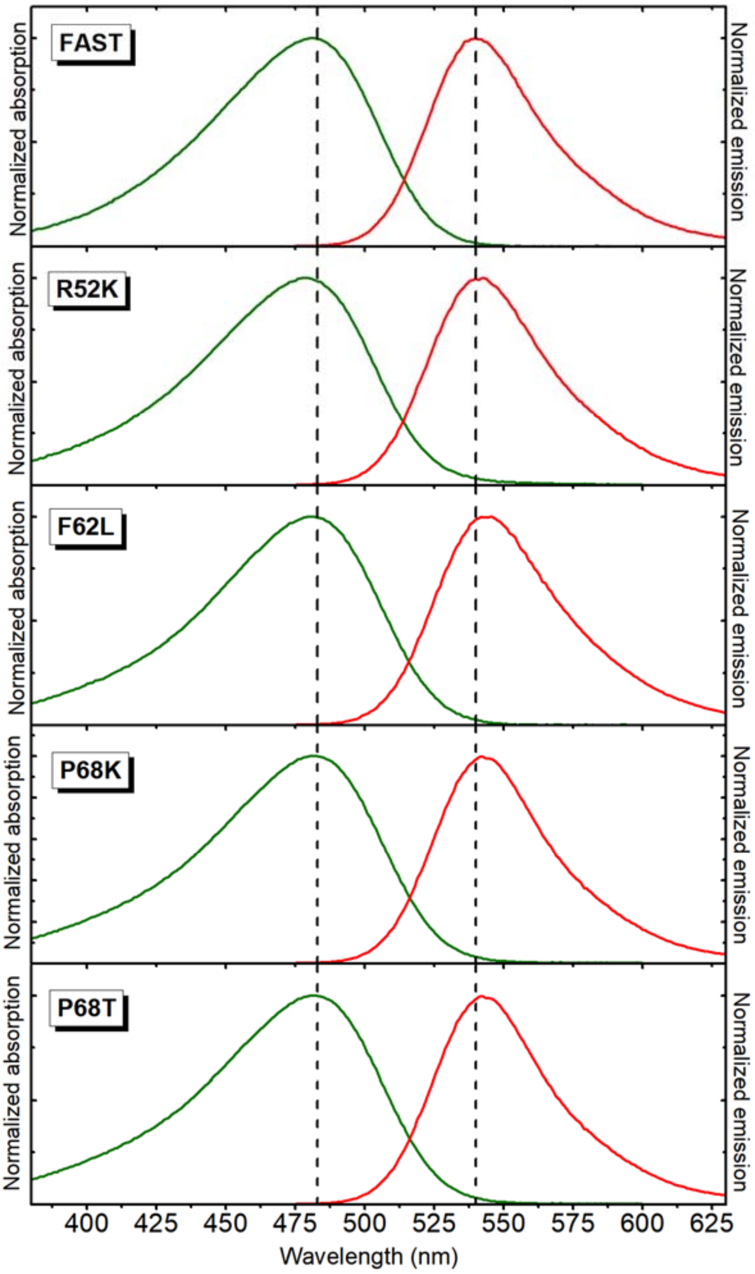

Figure S27. Fluorescence (red) and absorption (green) spectra of **HMBR** complexes with FAST variants.

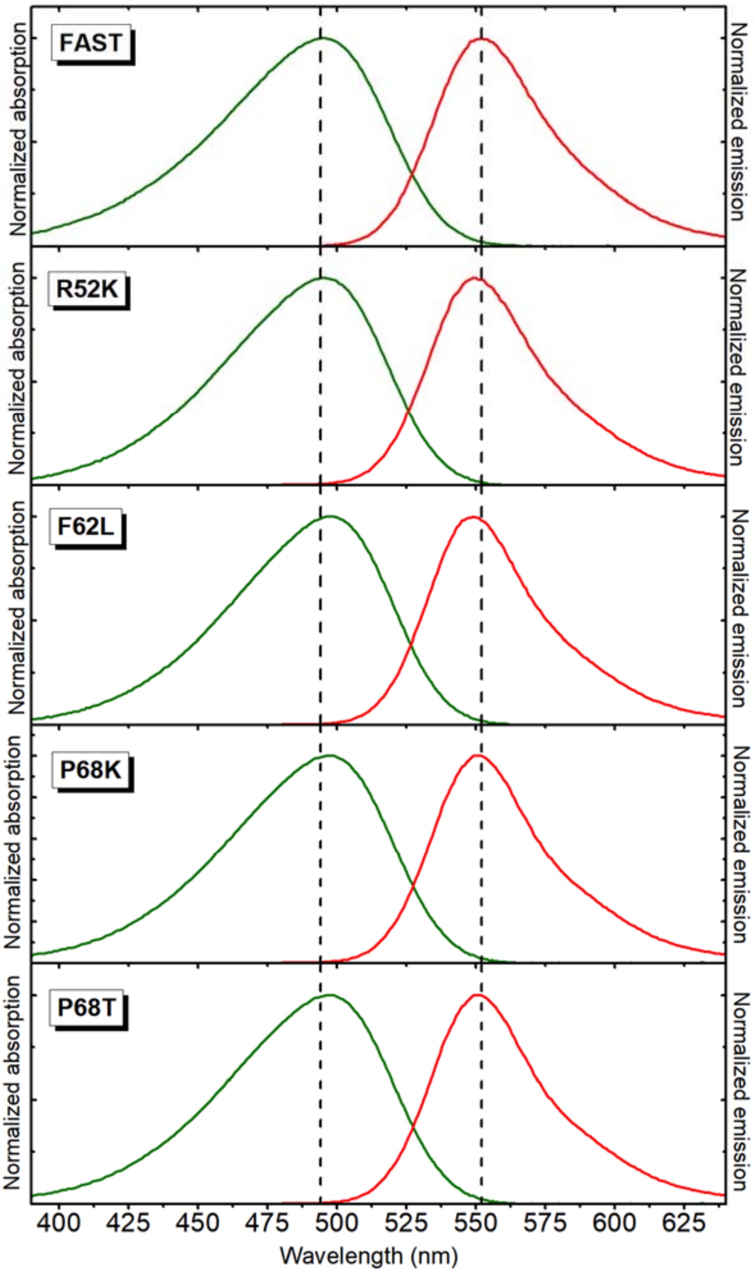

Figure S28. Fluorescence (red) and absorption (green) spectra of **HBR-2,5-DM** complexes with FAST mutants.

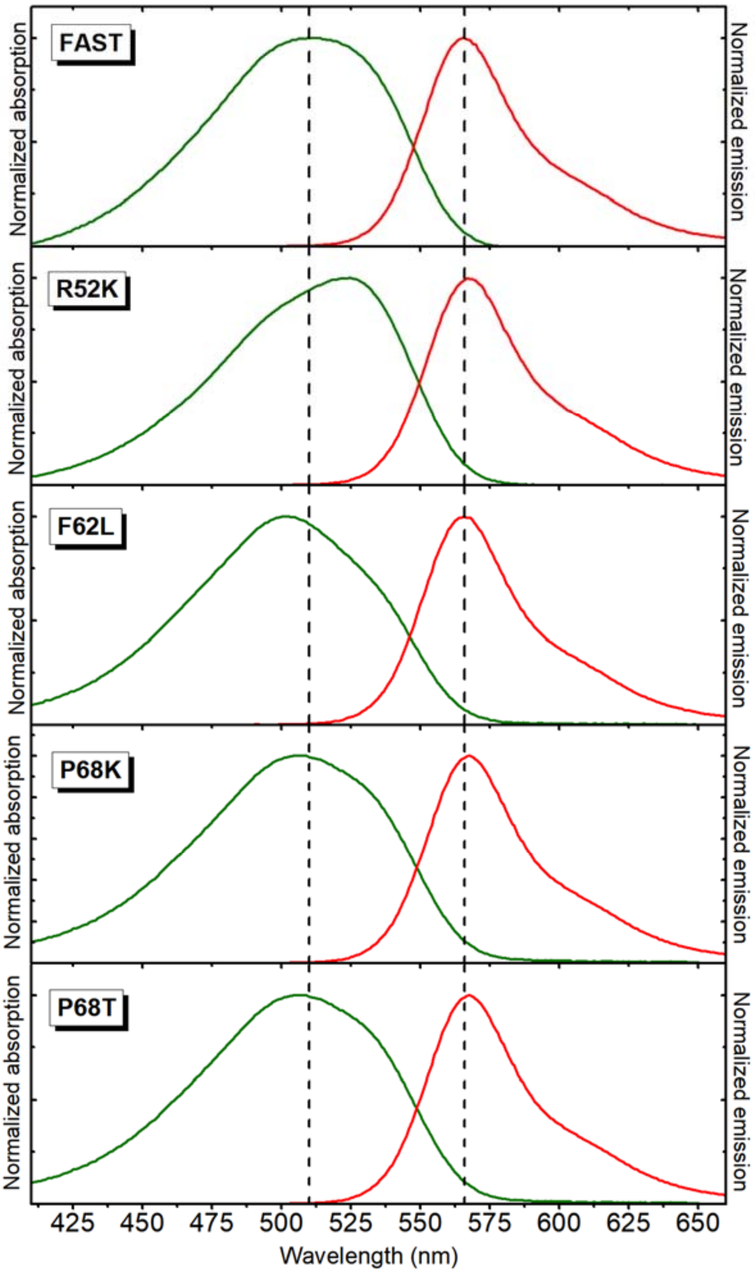

Figure S29. Fluorescence (red) and absorption (green) spectra of **HBR-DOM2** complexes with FAST mutants.

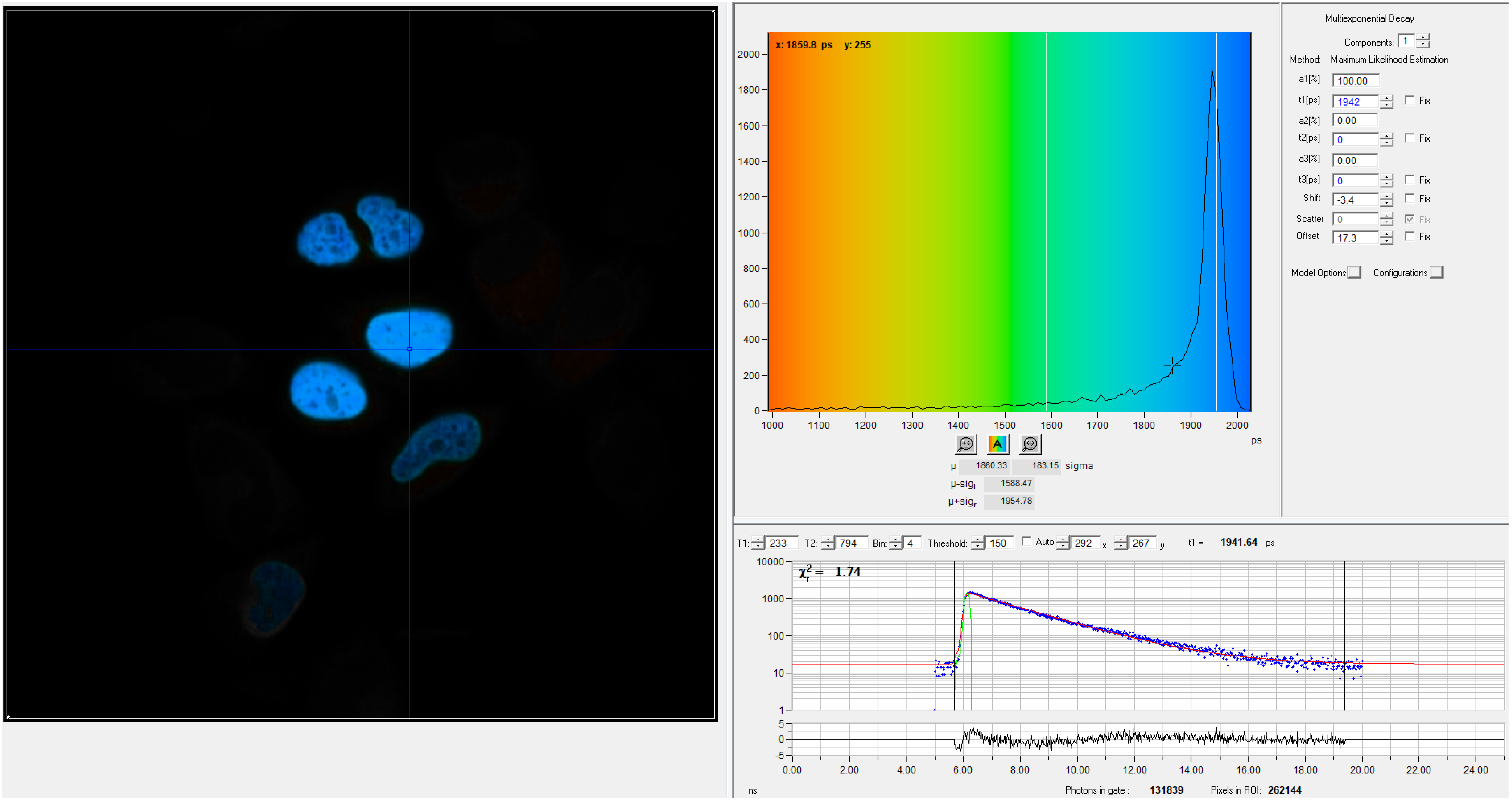

Figure S30. R52K FAST + **25DOM-HBI-2T**; monoexponential fit; τ color-coding. FLIM scan and corresponding time-resolved fluorescence data analysis of live HeLa cells expressing the R52K FAST variant fused to histone-2B (H2B) and stained with the **25DOM-HBI-2T** fluorogen. A screenshot from Becker & Hickl SPCImage data acquisition and analysis window is shown. Monoexponential fitting of decay data has been performed. On the left panel, there is a FLIM image of HeLa nuclei color-coded according to fluorescence lifetime in each pixel (τ). A histogram on the upper right panel displays the distribution of τ and color legend. The table next to it (rightmost) represents a monoexponential fitting model used to fit the data and fitting results. On the lower right panel, there are experimental decay data (blue dots), monoexponential fit of the data (red line), instrument response function (IRF) (green line) and fitting residuals (shown in black below the main data plot).

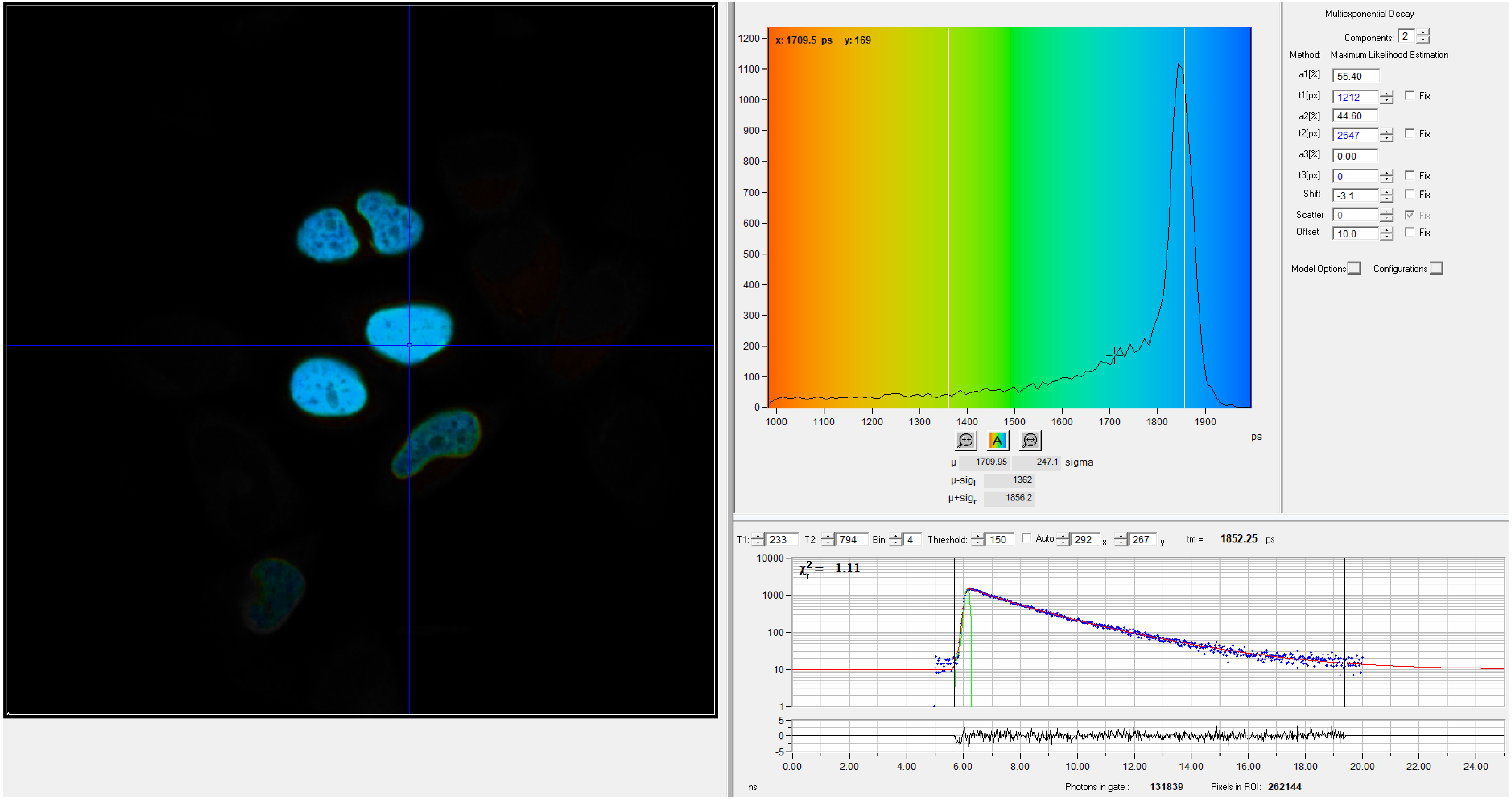

Figure S31. R52K FAST + **25DOM-HBI-2T**; biexponential fit; τ_m color-coding. FLIM scan and corresponding time-resolved fluorescence data analysis of live HeLa cells expressing the R52K FAST variant fused to histone-2B (H2B) and stained with the **25DOM-HBI-2T** fluorogen. A screenshot from Becker & Hickl SPCImage data acquisition and analysis window is shown. Biexponential fitting of decay data has been performed.On the left panel, there is a FLIM image of HeLa nuclei color-coded according to amplitude-weighted average fluorescence lifetime in each pixel (τ_m). A histogram on the upper right panel displays the distribution of τ_m and color legend. The table next to it (rightmost) represents a biexponential fitting model used to fit the data and fitting results. On the lower right panel, there are experimental decay data (blue dots), biexponential fit of the data (red line), instrument response function (IRF) (green line) and fitting residuals (shown in black below the main data plot).

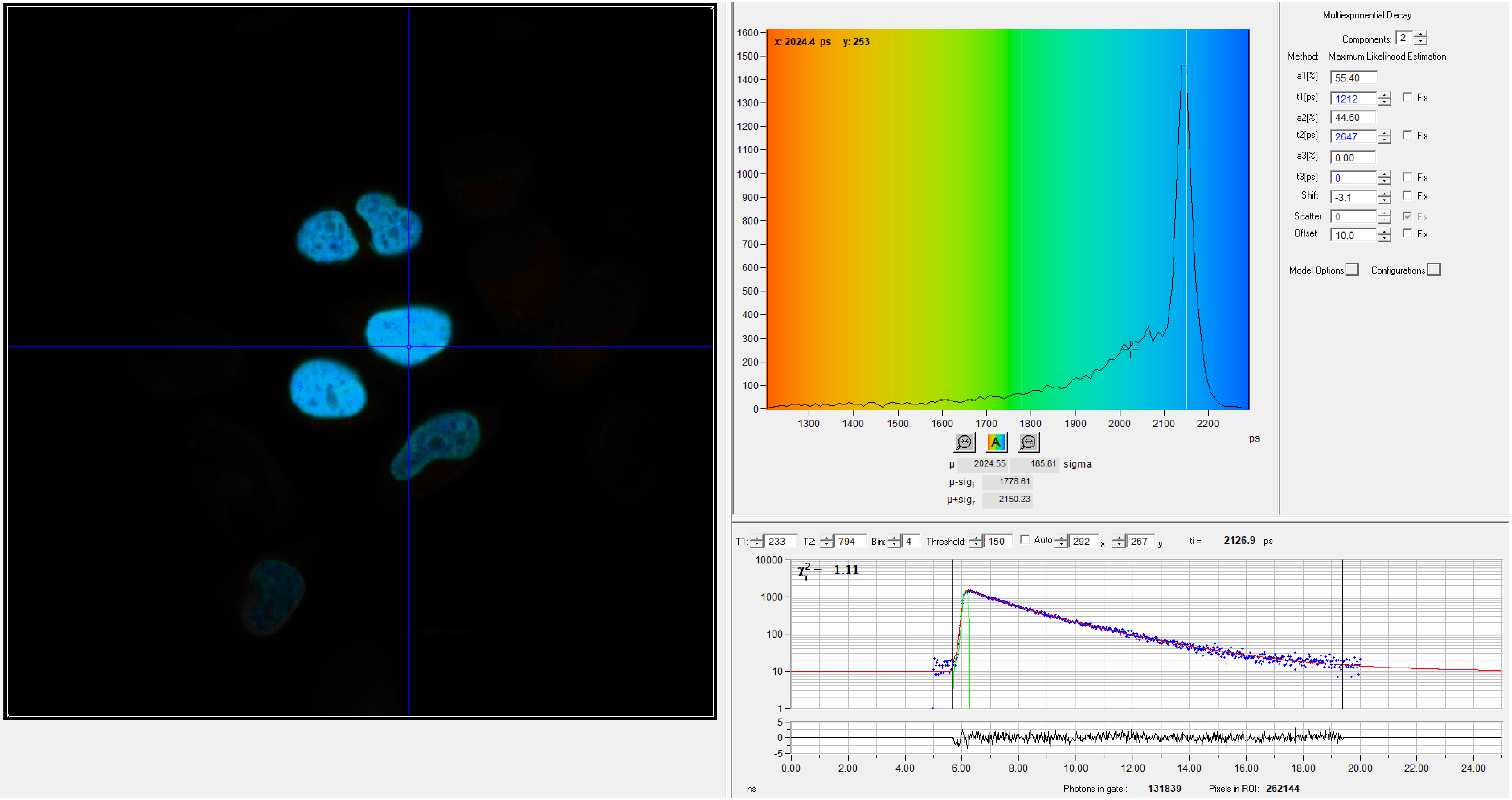

Figure S32. R52K FAST + **25DOM-HBI-2T**; biexponential fit; τ_i color-coding. FLIM scan and corresponding time-resolved fluorescence data analysis of live HeLa cells expressing the R52K FAST variant fused to histone-2B (H2B) and stained with the **25DOM-HBI-2T** fluorogen. A screenshot from Becker & Hickl SPCImage data acquisition and analysis window is shown. Biexponential fitting of decay data has been performed. On the left panel, there is a FLIM image of HeLa nuclei color-coded according to intensity-weighted average fluorescence lifetime in each pixel (τ_i). A histogram on the upper right panel displays the distribution of τ_i and color legend. The table next to it (rightmost) represents a biexponential fitting model used to fit the data and fitting results. On the lower right panel, there are experimental decay data (blue dots), biexponential fit of the data (red line), instrument response function (IRF) (green line) and fitting residuals (shown in black below the main data plot).

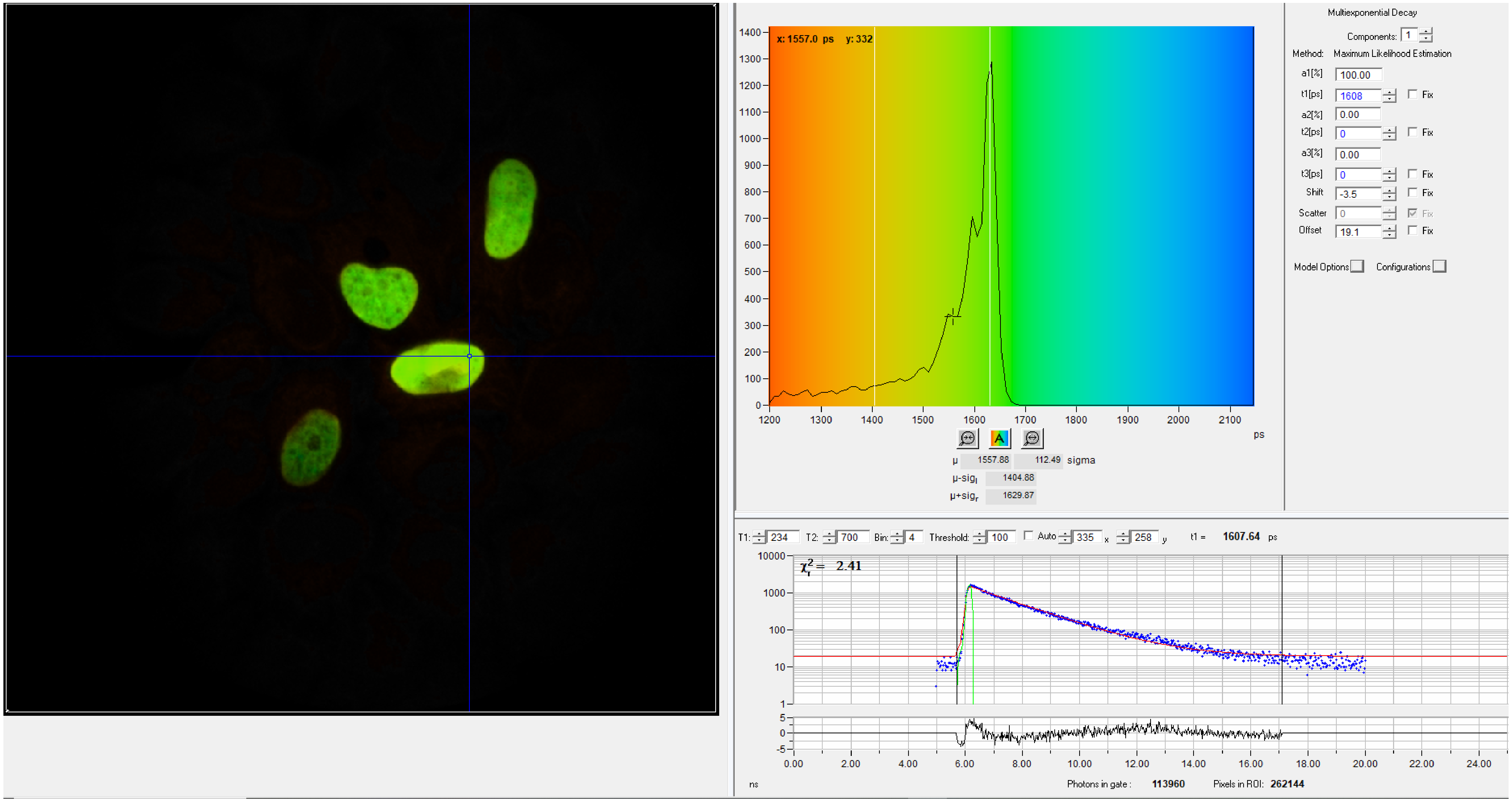

Figure S33. F62L FAST + **25DOM-HBI-2T**; monoexponential fit; τ color-coding. FLIM scan and corresponding time-resolved fluorescence data analysis of live HeLa cells expressing the F62L FAST variant fused to histone-2B (H2B) and stained with the **25DOM-HBI-2T** fluorogen. A screenshot from Becker & Hickl SPCImage data acquisition and analysis window is shown. Monoexponential fitting of decay data has been performed. On the left panel, there is a FLIM image of HeLa nuclei color-coded according to fluorescence lifetime in each pixel (τ). A histogram on the upper right panel displays the distribution of τ and color legend. The table next to it (rightmost) represents a monoexponential fitting model used to fit the data and fitting results. On the lower right panel, there are experimental decay data (blue dots), monoexponential fit of the data (red line), instrument response function (IRF) (green line) and fitting residuals (shown in black below the main data plot).

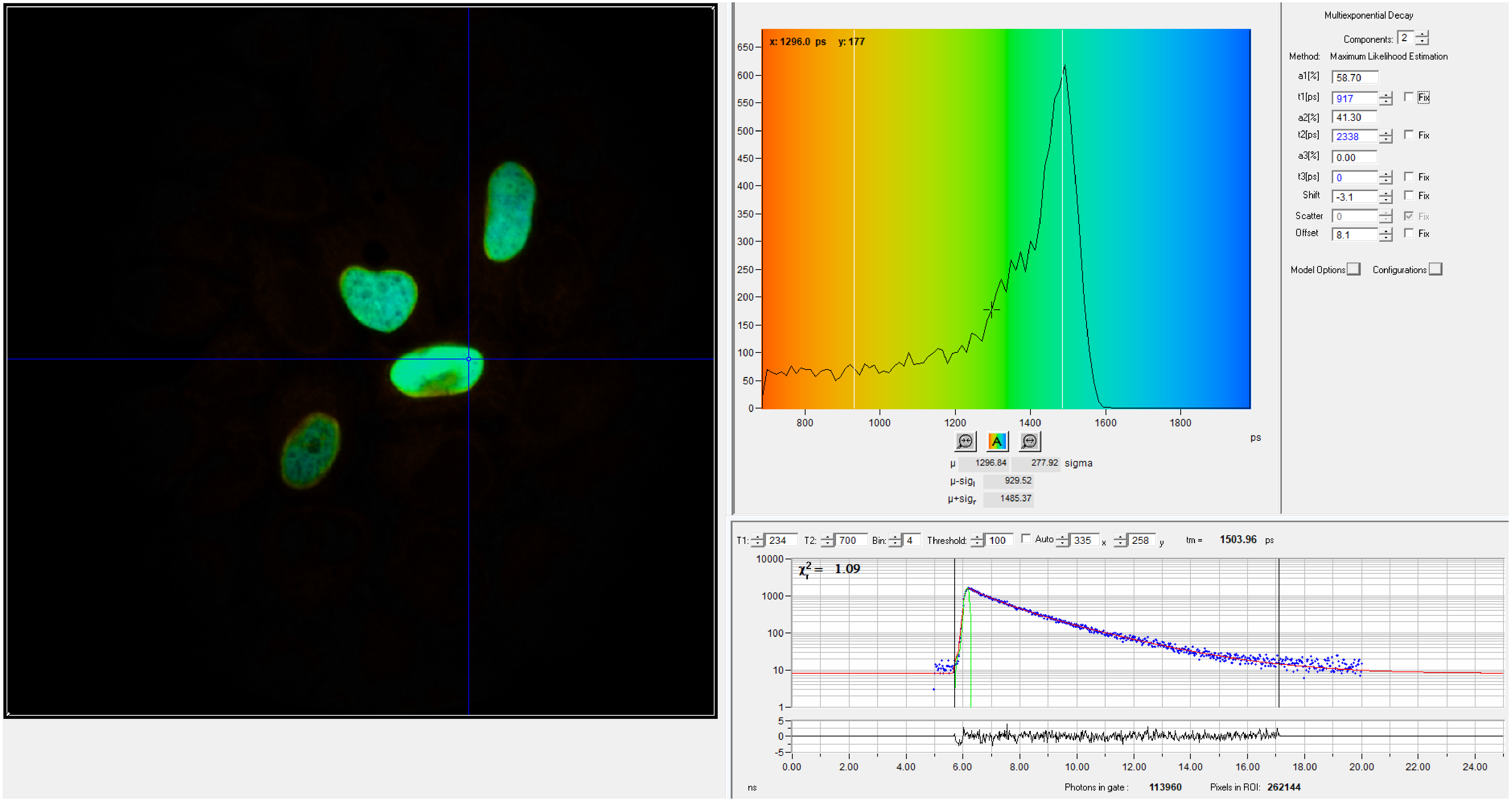

Figure S34. F62L FAST + **25DOM-HBI-2T**; biexponential fit; τ_m color-coding. FLIM scan and corresponding time-resolved fluorescence data analysis of live HeLa cells expressing the F62L FAST variant fused to histone-2B (H2B) and stained with the **25DOM-HBI-2T** fluorogen. A screenshot from Becker & Hickl SPCImage data acquisition and analysis window is shown. Biexponential fitting of decay data has been performed.On the left panel, there is a FLIM image of HeLa nuclei color-coded according to amplitude-weighted average fluorescence lifetime in each pixel (τ_m). A histogram on the upper right panel displays the distribution of τ_m and color legend. The table next to it (rightmost) represents a biexponential fitting model used to fit the data and fitting results. On the lower right panel, there are experimental decay data (blue dots), biexponential fit of the data (red line), instrument response function (IRF) (green line) and fitting residuals (shown in black below the main data plot).

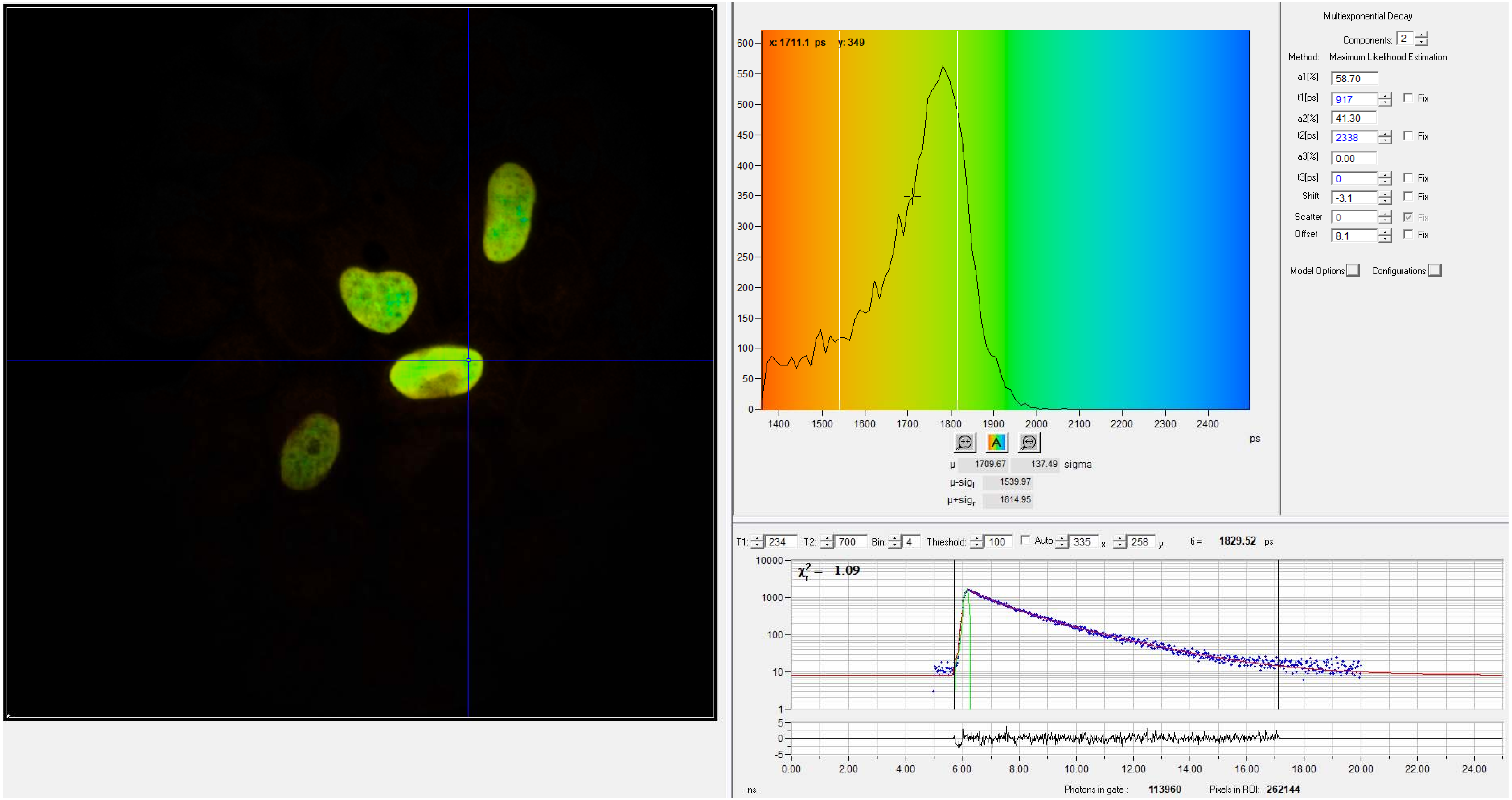

Figure S35. F62L FAST + **25DOM-HBI-2T**; biexponential fit; τ_i color-coding. FLIM scan and corresponding time-resolved fluorescence data analysis of live HeLa cells expressing the F62L FAST variant fused to histone-2B (H2B) and stained with the **25DOM-HBI-2T** fluorogen. A screenshot from Becker & Hickl SPCImage data acquisition and analysis window is shown. Biexponential fitting of decay data has been performed. On the left panel, there is a FLIM image of HeLa nuclei color-coded according to intensity-weighted average fluorescence lifetime in each pixel (τ_i). A histogram on the upper right panel displays the distribution of τ_i and color legend. The table next to it (rightmost) represents a biexponential fitting model used to fit the data and fitting results. On the lower right panel, there are experimental decay data (blue dots), biexponential fit of the data (red line), instrument response function (IRF) (green line) and fitting residuals (shown in black below the main data plot).

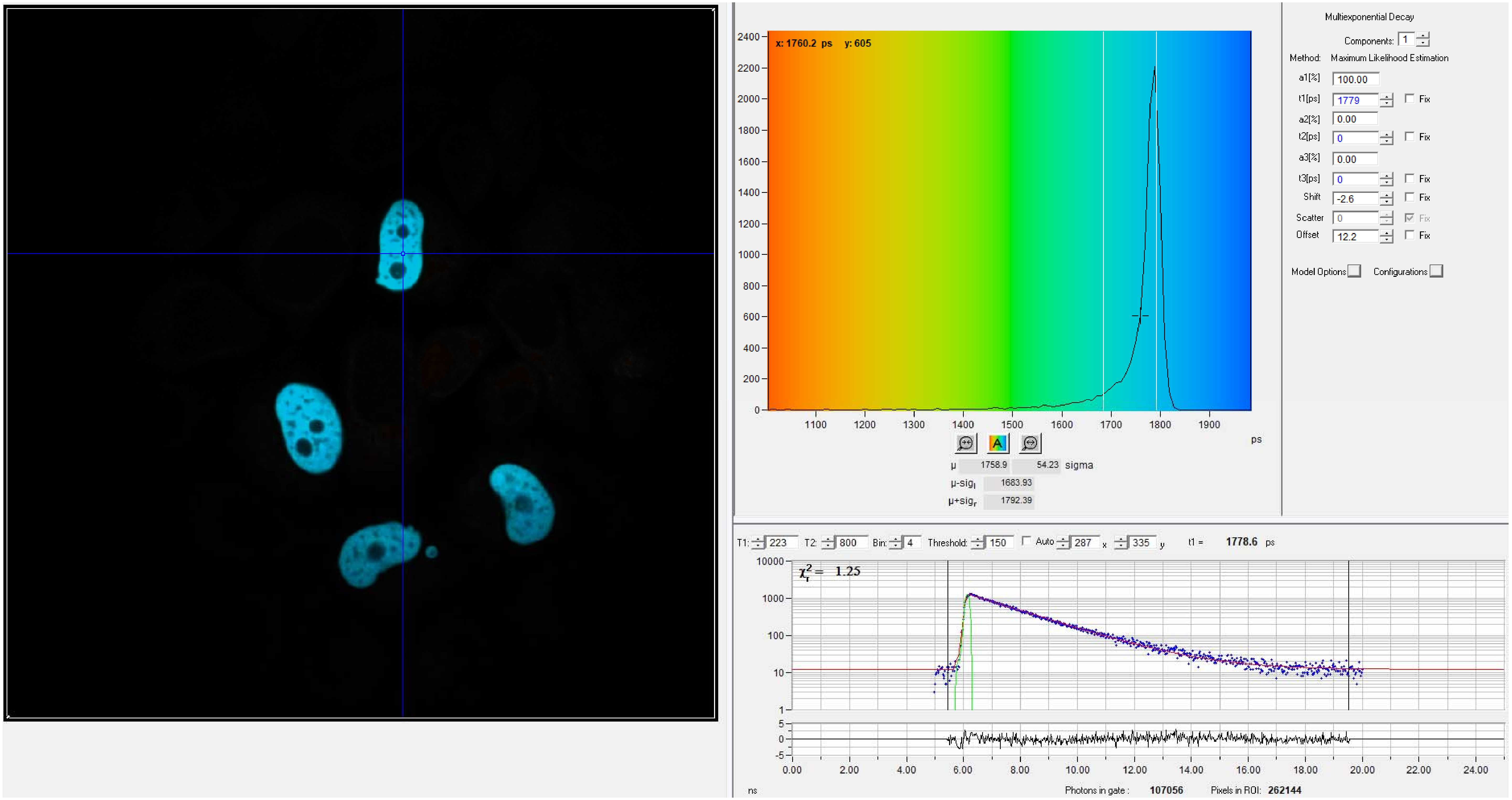

Figure S36. P68K FAST + **25DOM-HBI-2T**; monoexponential fit; τ color-coding. FLIM scan and corresponding time-resolved fluorescence data analysis of live HeLa cells expressing the P68K FAST variant fused to histone-2B (H2B) and stained with the **25DOM-HBI-2T** fluorogen. A screenshot from Becker & Hickl SPCImage data acquisition and analysis window is shown. Monoexponential fitting of decay data has been performed. On the left panel, there is a FLIM image of HeLa nuclei color-coded according to fluorescence lifetime in each pixel (τ). A histogram on the upper right panel displays the distribution of τ and color legend. The table next to it (rightmost) represents a monoexponential fitting model used to fit the data and fitting results. On the lower right panel, there are experimental decay data (blue dots), monoexponential fit of the data (red line), instrument response function (IRF) (green line) and fitting residuals (shown in black below the main data plot).

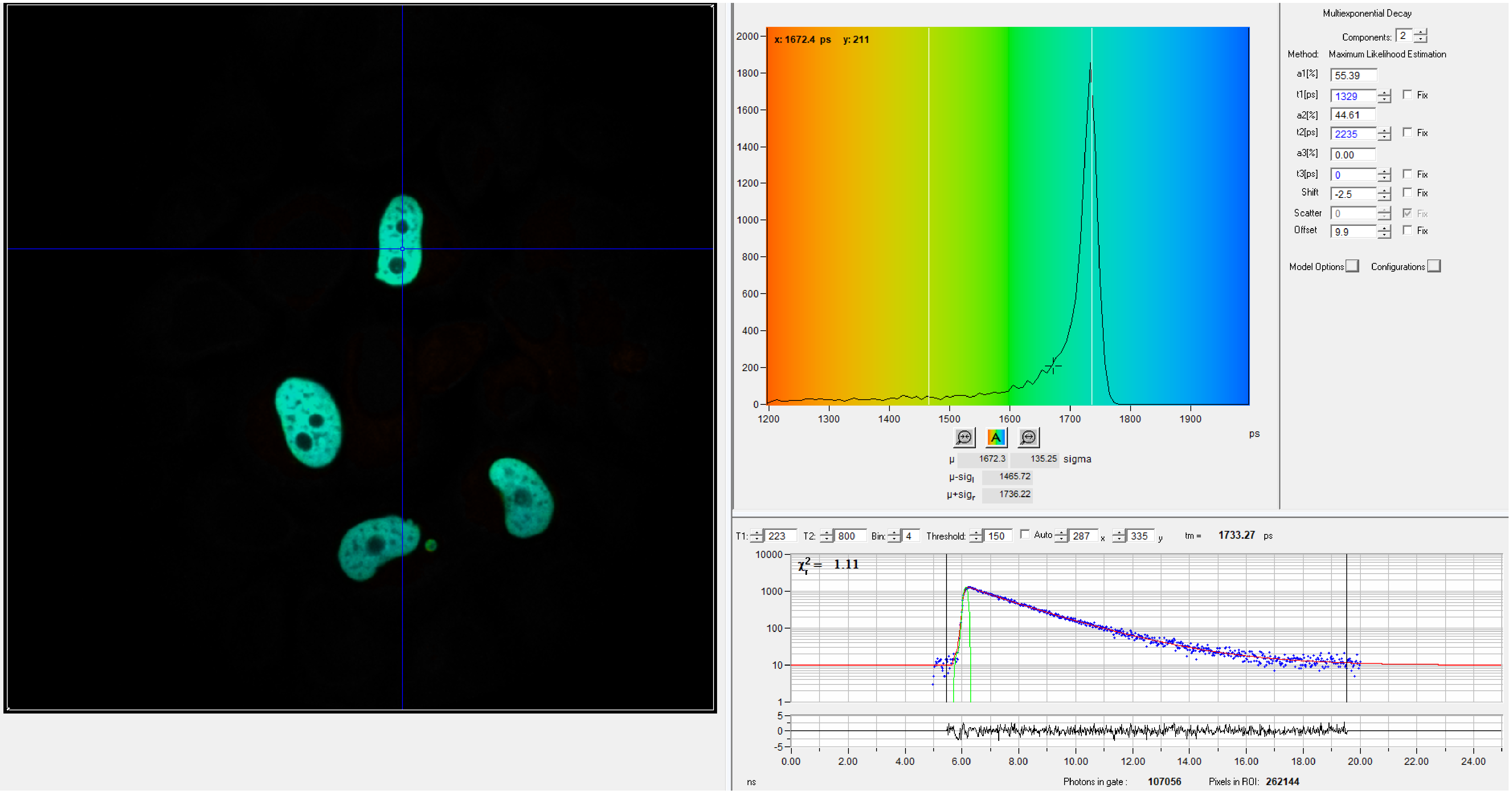

Figure S37. P68K FAST + **25DOM-HBI-2T**; biexponential fit; τ_m color-coding. FLIM scan and corresponding time-resolved fluorescence data analysis of live HeLa cells expressing the P68K FAST variant fused to histone-2B (H2B) and stained with the **25DOM-HBI-2T** fluorogen. A screenshot from Becker & Hickl SPCImage data acquisition and analysis window is shown. Biexponential fitting of decay data has been performed.On the left panel, there is a FLIM image of HeLa nuclei color-coded according to amplitude-weighted average fluorescence lifetime in each pixel (τ_m). A histogram on the upper right panel displays the distribution of τ_m and color legend. The table next to it (rightmost) represents a biexponential fitting model used to fit the data and fitting results. On the lower right panel, there are experimental decay data (blue dots), biexponential fit of the data (red line), instrument response function (IRF) (green line) and fitting residuals (shown in black below the main data plot).

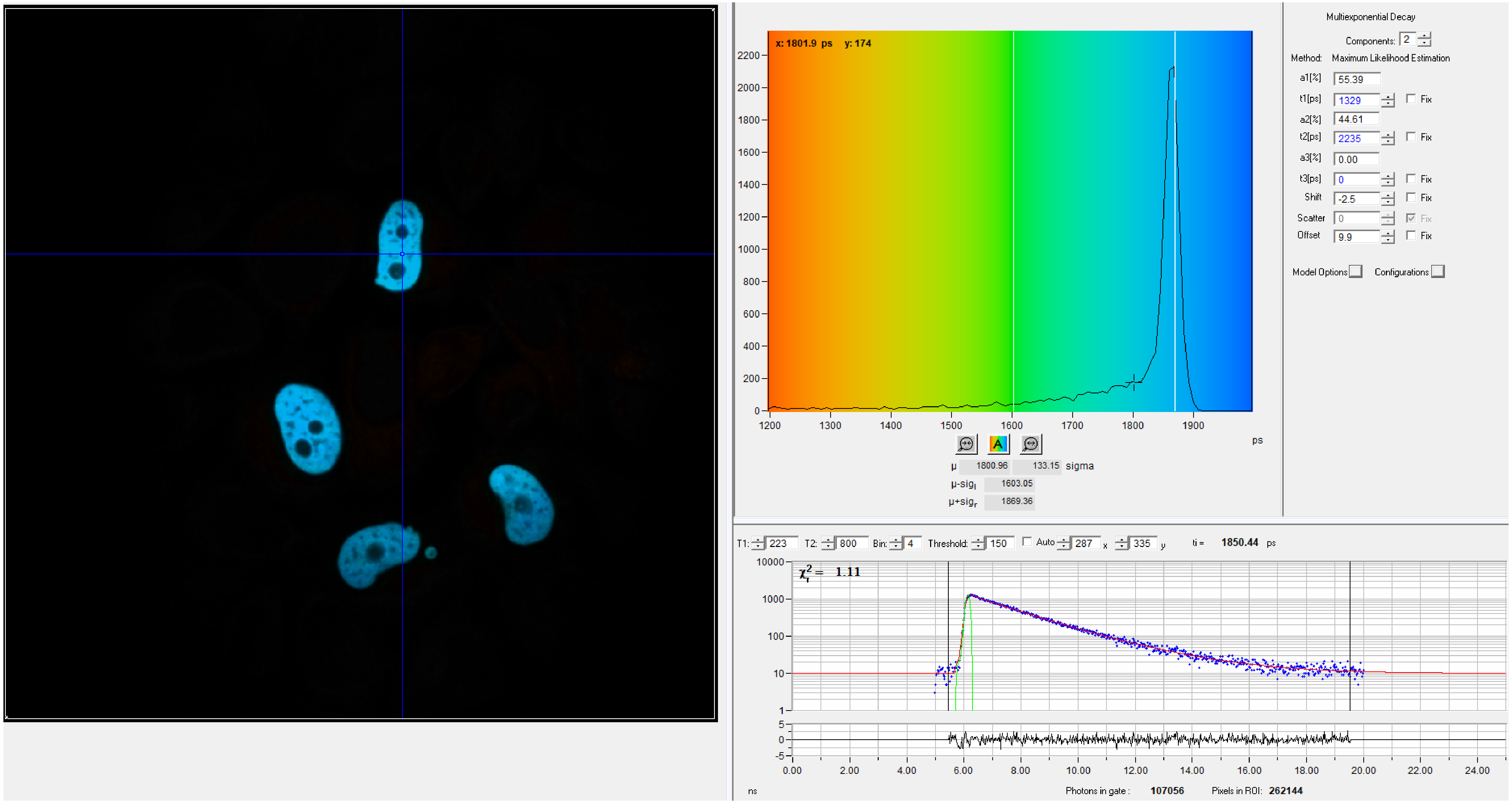

Figure S38. P68K FAST + **25DOM-HBI-2T**; biexponential fit; τ_i color-coding. FLIM scan and corresponding time-resolved fluorescence data analysis of live HeLa cells expressing the P68K FAST variant fused to histone-2B (H2B) and stained with the **25DOM-HBI-2T** fluorogen. A screenshot from Becker & Hickl SPCImage data acquisition and analysis window is shown. Biexponential fitting of decay data has been performed. On the left panel, there is a FLIM image of HeLa nuclei color-coded according to intensity-weighted average fluorescence lifetime in each pixel (τ_i). A histogram on the upper right panel displays the distribution of τ_i and color legend. The table next to it (rightmost) represents a biexponential fitting model used to fit the data and fitting results. On the lower right panel, there are experimental decay data (blue dots), biexponential fit of the data (red line), instrument response function (IRF) (green line) and fitting residuals (shown in black below the main data plot).

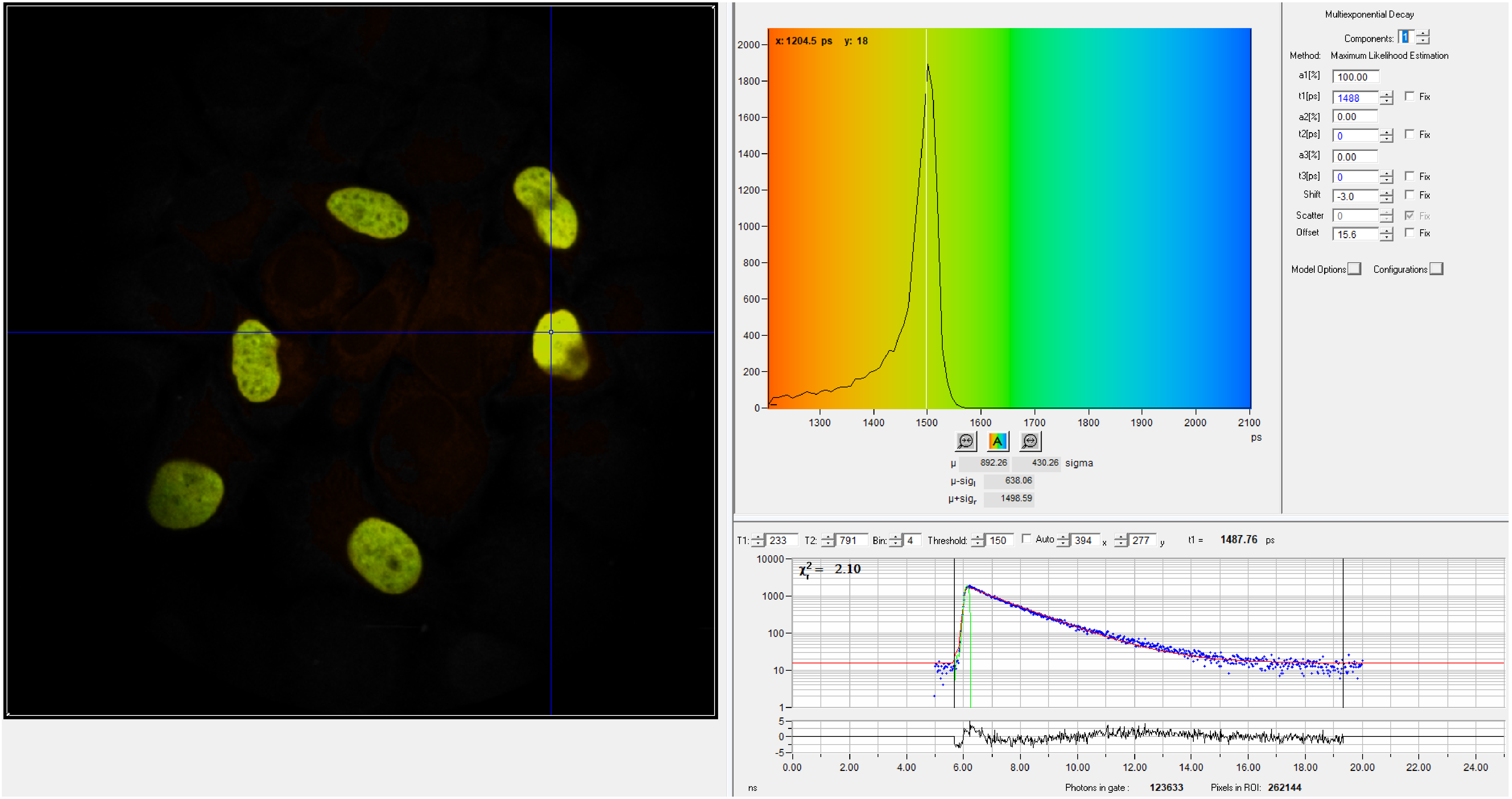

Figure S39. P68T FAST + **25DOM-HBI-2T**; monoexponential fit; τ color-coding. FLIM scan and corresponding time-resolved fluorescence data analysis of live HeLa cells expressing the P68T FAST variant fused to histone-2B (H2B) and stained with the **25DOM-HBI-2T** fluorogen. A screenshot from Becker & Hickl SPCImage data acquisition and analysis window is shown. Monoexponential fitting of decay data has been performed. On the left panel, there is a FLIM image of HeLa nuclei color-coded according to fluorescence lifetime in each pixel (τ). A histogram on the upper right panel displays the distribution of τ and color legend. The table next to it (rightmost) represents a monoexponential fitting model used to fit the data and fitting results. On the lower right panel, there are experimental decay data (blue dots), monoexponential fit of the data (red line), instrument response function (IRF) (green line) and fitting residuals (shown in black below the main data plot).

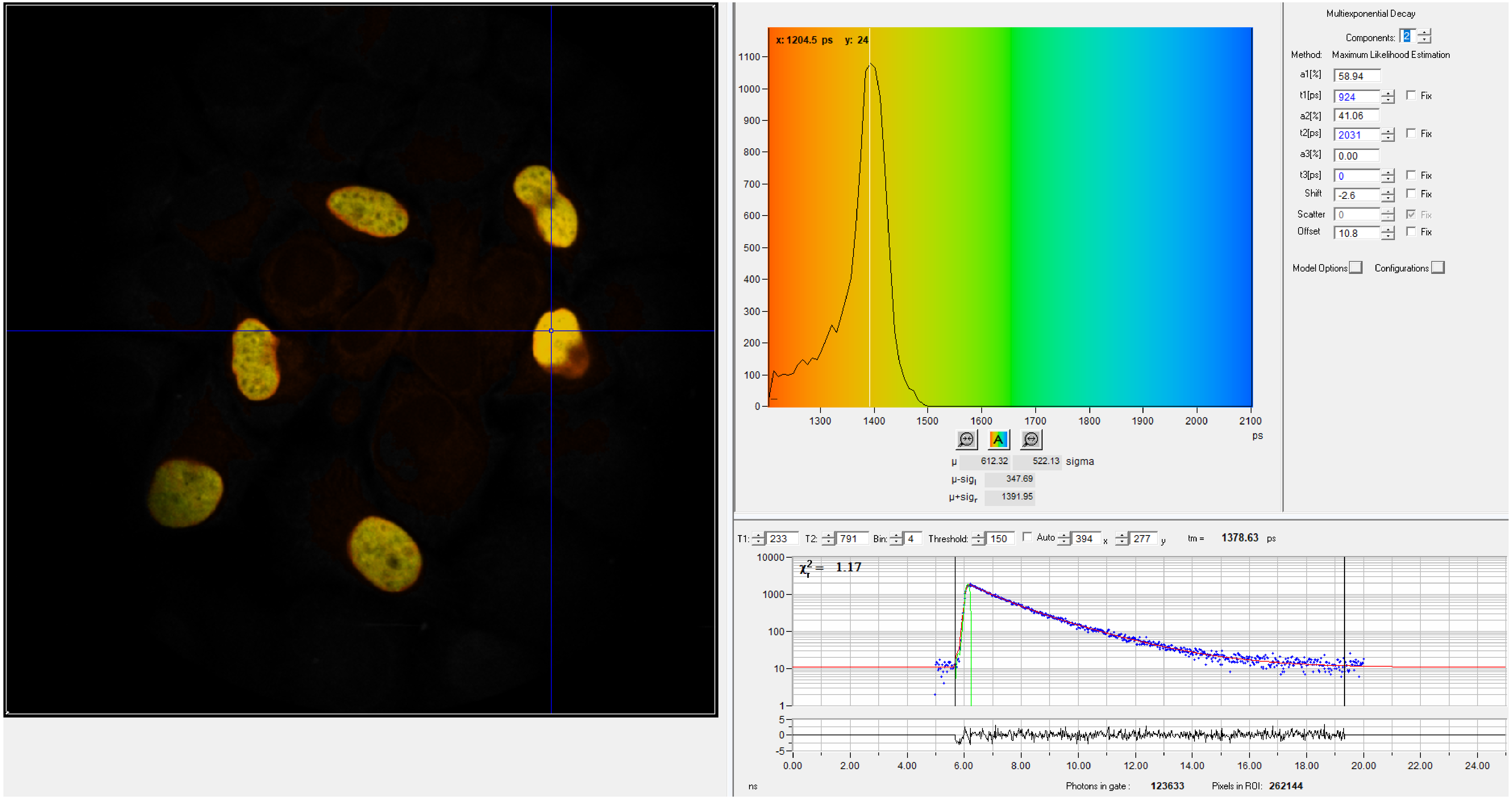

Figure S40. P68T FAST + **25DOM-HBI-2T**; biexponential fit; τ_m color-coding. FLIM scan and corresponding time-resolved fluorescence data analysis of live HeLa cells expressing the P68T FAST variant fused to histone-2B (H2B) and stained with the **25DOM-HBI-2T** fluorogen. A screenshot from Becker & Hickl SPCImage data acquisition and analysis window is shown. Biexponential fitting of decay data has been performed.On the left panel, there is a FLIM image of HeLa nuclei color-coded according to amplitude-weighted average fluorescence lifetime in each pixel (τ_m). A histogram on the upper right panel displays the distribution of τ_m and color legend. The table next to it (rightmost) represents a biexponential fitting model used to fit the data and fitting results. On the lower right panel, there are experimental decay data (blue dots), biexponential fit of the data (red line), instrument response function (IRF) (green line) and fitting residuals (shown in black below the main data plot).

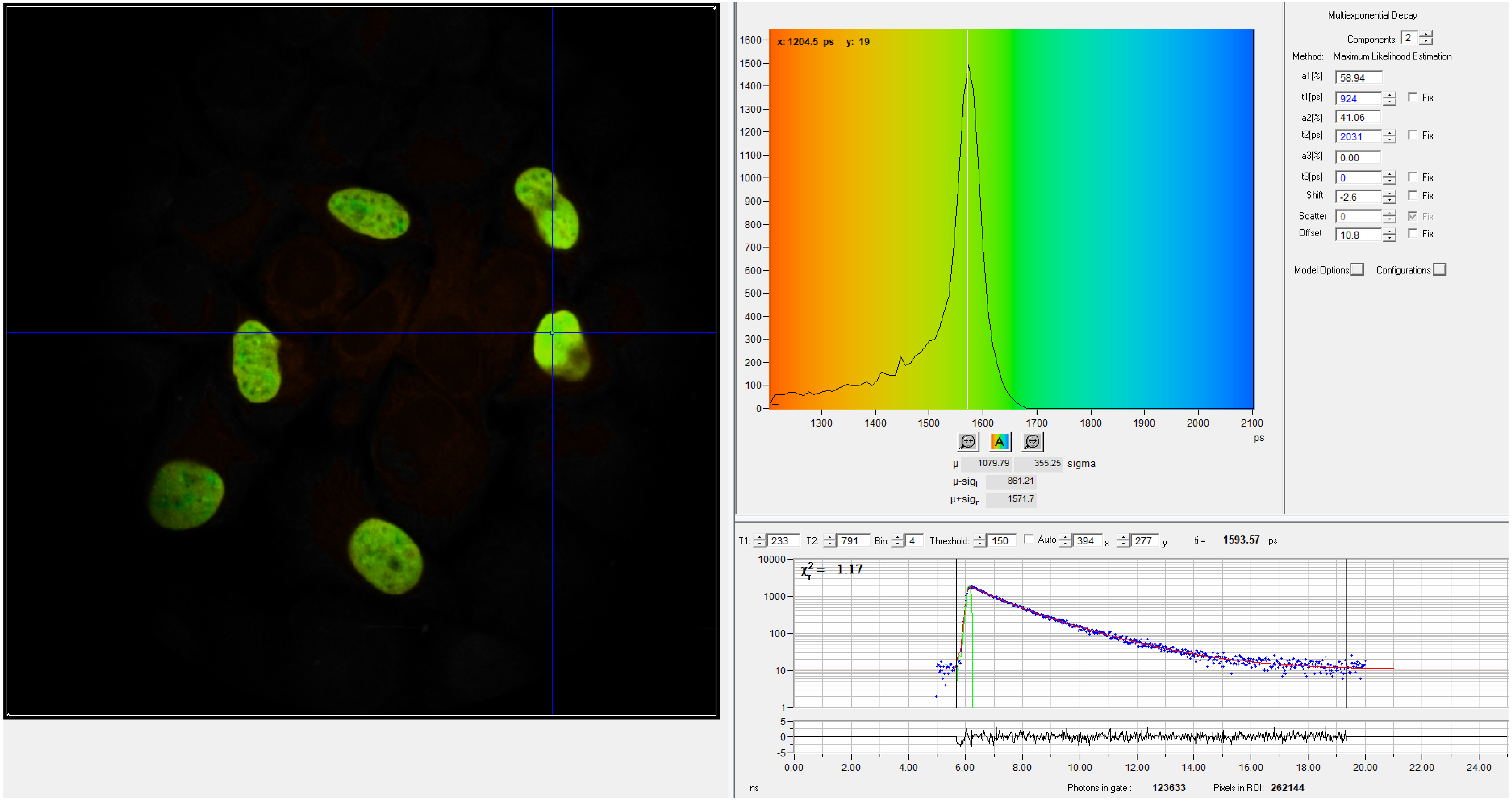

Figure S41. P68T FAST + **25DOM-HBI-2T**; biexponential fit; τ_i color-coding. FLIM scan and corresponding time-resolved fluorescence data analysis of live HeLa cells expressing the P68T FAST variant fused to histone-2B (H2B) and stained with the **25DOM-HBI-2T** fluorogen. A screenshot from Becker & Hickl SPCImage data acquisition and analysis window is shown. Biexponential fitting of decay data has been performed. On the left panel, there is a FLIM image of HeLa nuclei color-coded according to intensity-weighted average fluorescence lifetime in each pixel (τ_i). A histogram on the upper right panel displays the distribution of τ_i and color legend. The table next to it (rightmost) represents a biexponential fitting model used to fit the data and fitting results. On the lower right panel, there are experimental decay data (blue dots), biexponential fit of the data (red line), instrument response function (IRF) (green line) and fitting residuals (shown in black below the main data plot).

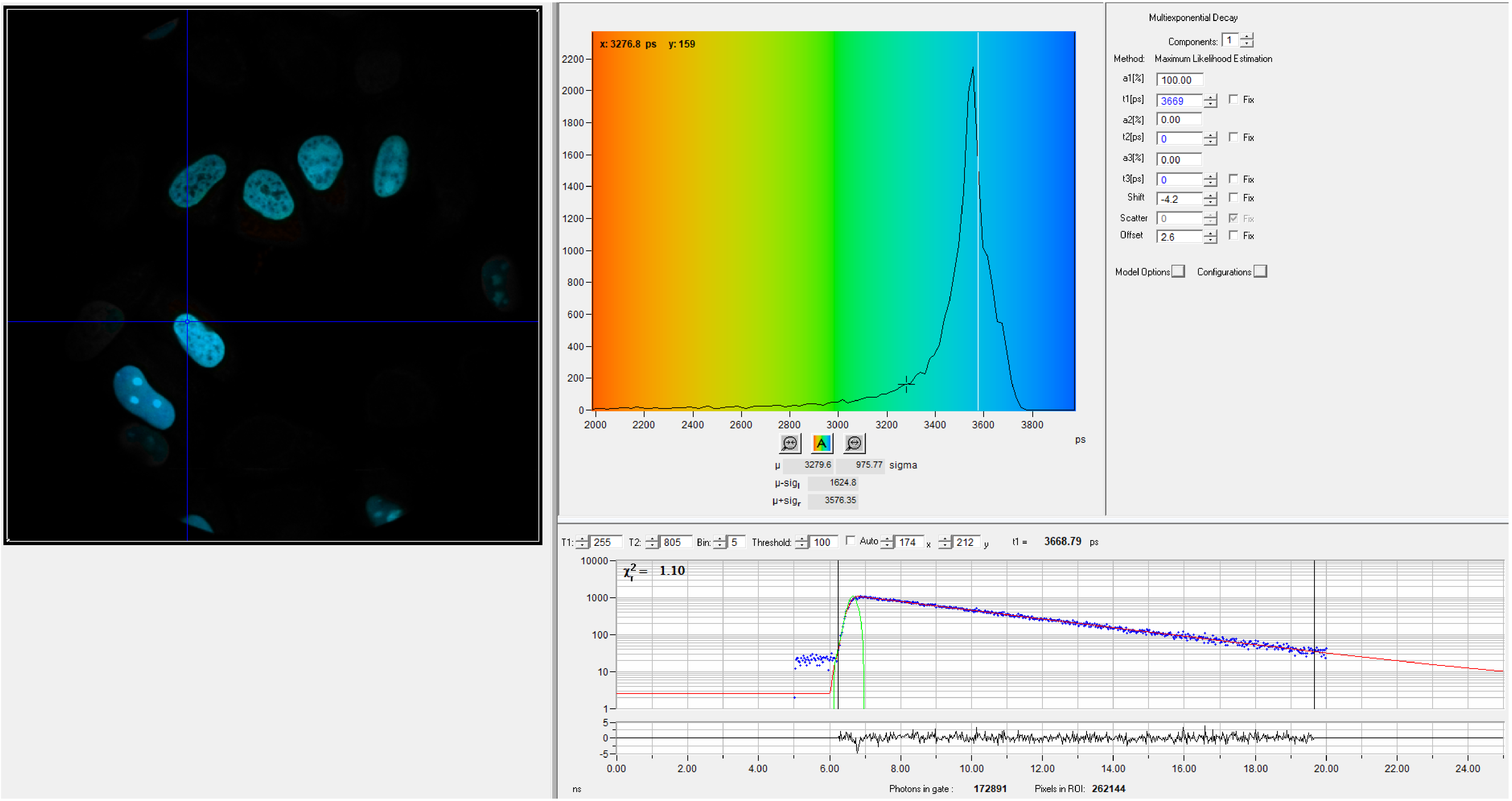

Figure S42. R52K FAST + **HBR-DOM2**; monoexponential fit; τ color-coding. FLIM scan and corresponding time-resolved fluorescence data analysis of live HeLa cells expressing the R52K FAST variant fused to histone-2B (H2B) and stained with the **HBR-DOM2** fluorogen. A screenshot from Becker & Hickl SPCImage data acquisition and analysis window is shown. Monoexponential fitting of decay data has been performed. On the left panel, there is a FLIM image of HeLa nuclei color-coded according to fluorescence lifetime in each pixel (τ). A histogram on the upper right panel displays the distribution of τ and color legend. The table next to it (rightmost) represents a monoexponential fitting model used to fit the data and fitting results. On the lower right panel, there are experimental decay data (blue dots), monoexponential fit of the data (red line), instrument response function (IRF) (green line) and fitting residuals (shown in black below the main data plot).

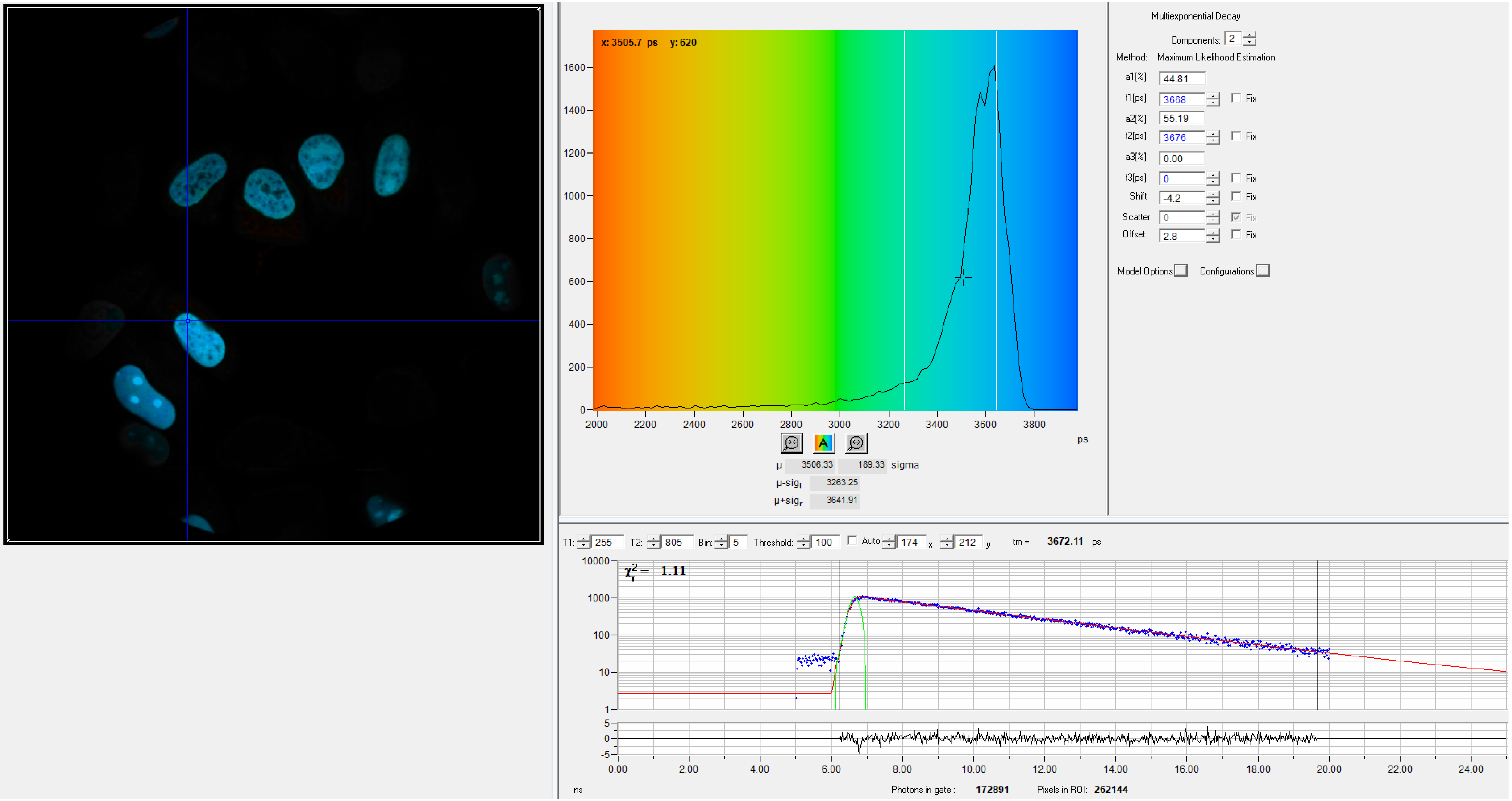

Figure S43. R52K FAST + **HBR-DOM2**; biexponential fit; τ_m color-coding. FLIM scan and corresponding time-resolved fluorescence data analysis of live HeLa cells expressing the R52K FAST variant fused to histone-2B (H2B) and stained with the **HBR-DOM2** fluorogen. A screenshot from Becker & Hickl SPCImage data acquisition and analysis window is shown. Biexponential fitting of decay data has been performed.On the left panel, there is a FLIM image of HeLa nuclei color-coded according to amplitude-weighted average fluorescence lifetime in each pixel (τ_m). A histogram on the upper right panel displays the distribution of τ_m and color legend. The table next to it (rightmost) represents a biexponential fitting model used to fit the data and fitting results. On the lower right panel, there are experimental decay data (blue dots), biexponential fit of the data (red line), instrument response function (IRF) (green line) and fitting residuals (shown in black below the main data plot).

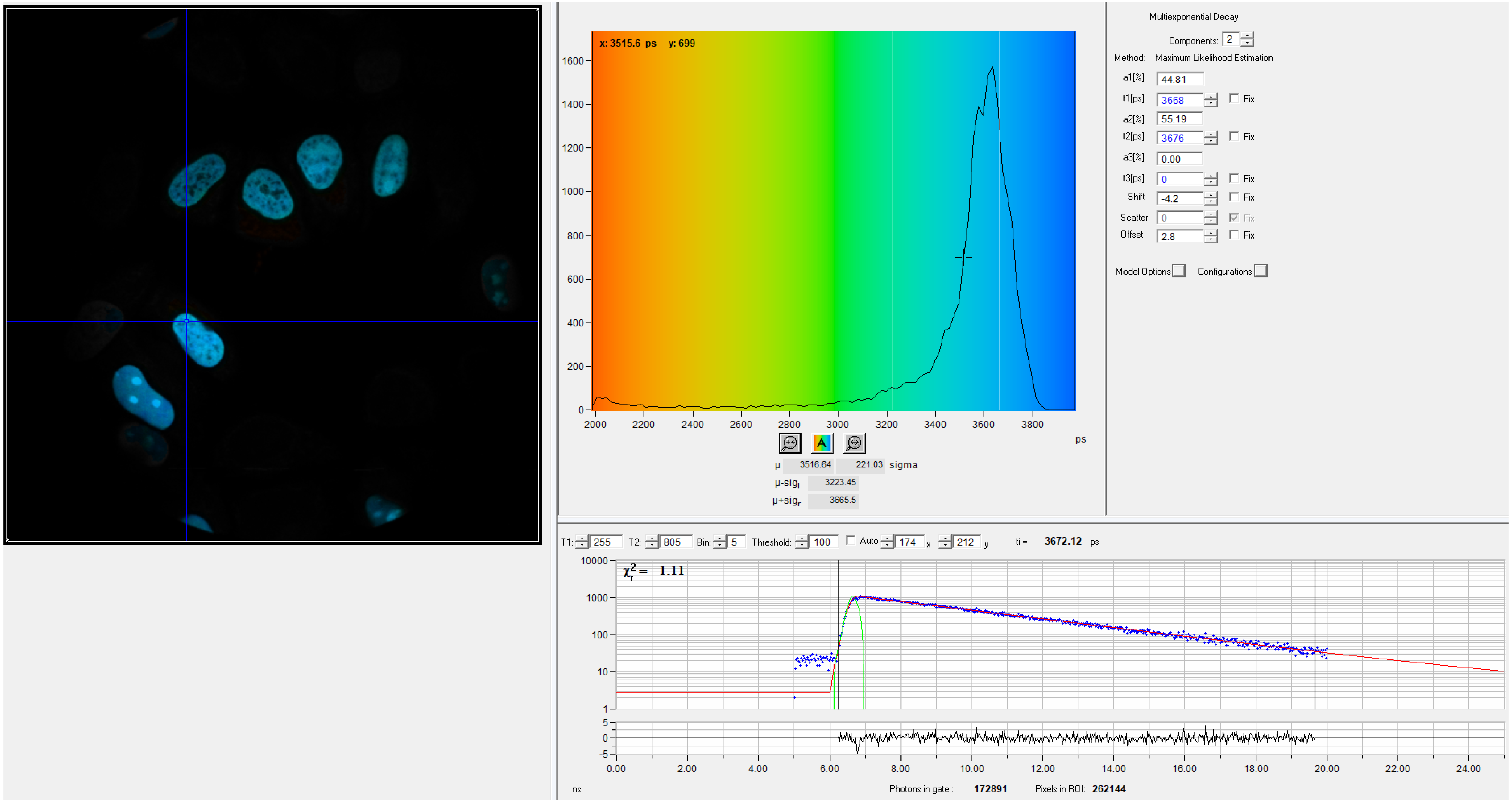

Figure S44. R52K FAST + **HBR-DOM2**; biexponential fit; τ_i color-coding. FLIM scan and corresponding time-resolved fluorescence data analysis of live HeLa cells expressing the R52K FAST variant fused to histone-2B (H2B) and stained with the **HBR-DOM2** fluorogen. A screenshot from Becker & Hickl SPCImage data acquisition and analysis window is shown. Biexponential fitting of decay data has been performed. On the left panel, there is a FLIM image of HeLa nuclei color-coded according to intensity-weighted average fluorescence lifetime in each pixel (τ_i). A histogram on the upper right panel displays the distribution of τ_i and color legend. The table next to it (rightmost) represents a biexponential fitting model used to fit the data and fitting results. On the lower right panel, there are experimental decay data (blue dots), biexponential fit of the data (red line), instrument response function (IRF) (green line) and fitting residuals (shown in black below the main data plot).

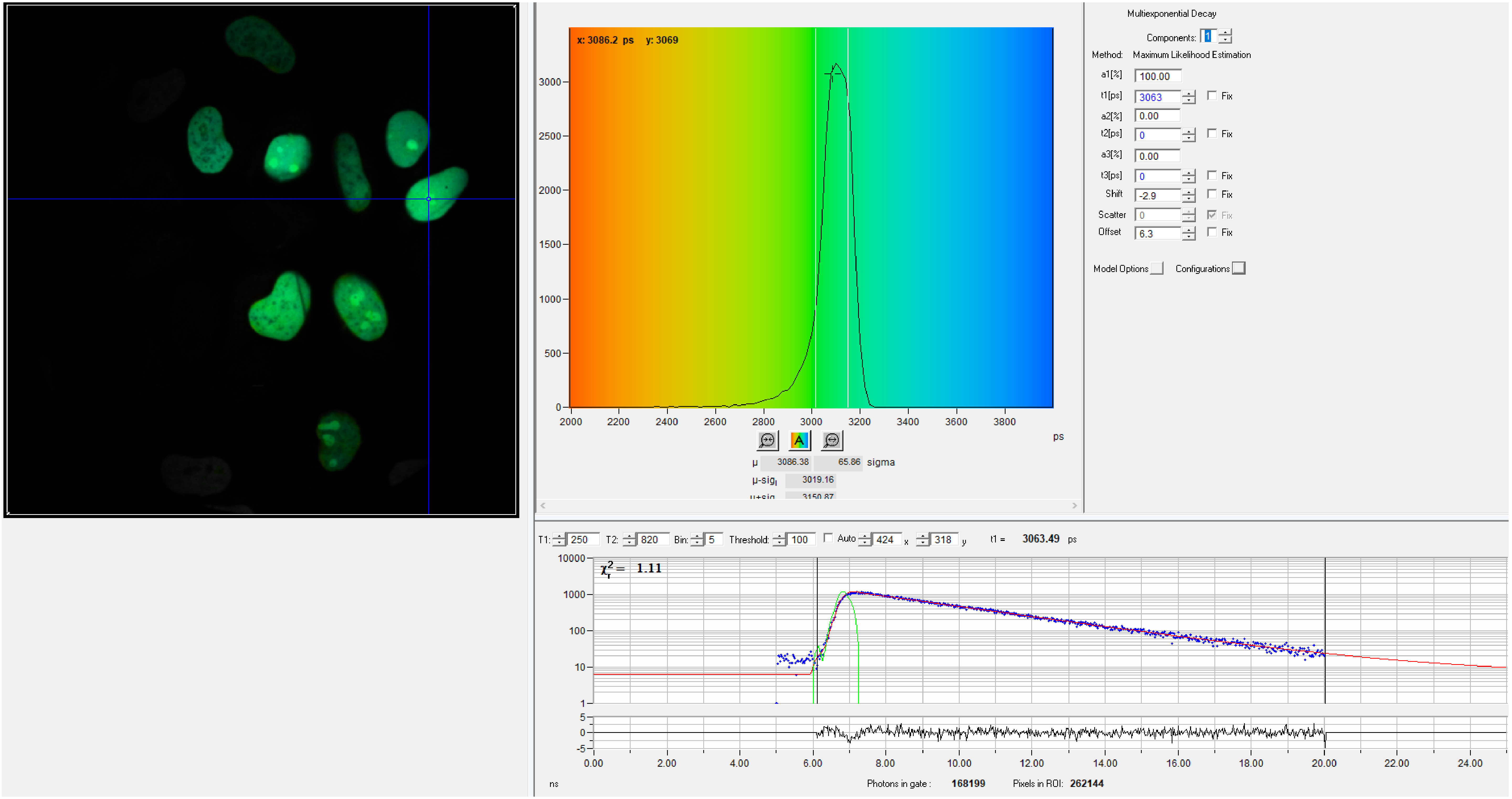

Figure S45. F62L FAST + **HBR-DOM2**; monoexponential fit; τ color-coding. FLIM scan and corresponding time-resolved fluorescence data analysis of live HeLa cells expressing the F62L FAST variant fused to histone-2B (H2B) and stained with the **HBR-DOM2** fluorogen. A screenshot from Becker & Hickl SPCImage data acquisition and analysis window is shown. Monoexponential fitting of decay data has been performed. On the left panel, there is a FLIM image of HeLa nuclei color-coded according to fluorescence lifetime in each pixel (τ). A histogram on the upper right panel displays the distribution of τ and color legend. The table next to it (rightmost) represents a monoexponential fitting model used to fit the data and fitting results. On the lower right panel, there are experimental decay data (blue dots), monoexponential fit of the data (red line), instrument response function (IRF) (green line) and fitting residuals (shown in black below the main data plot).

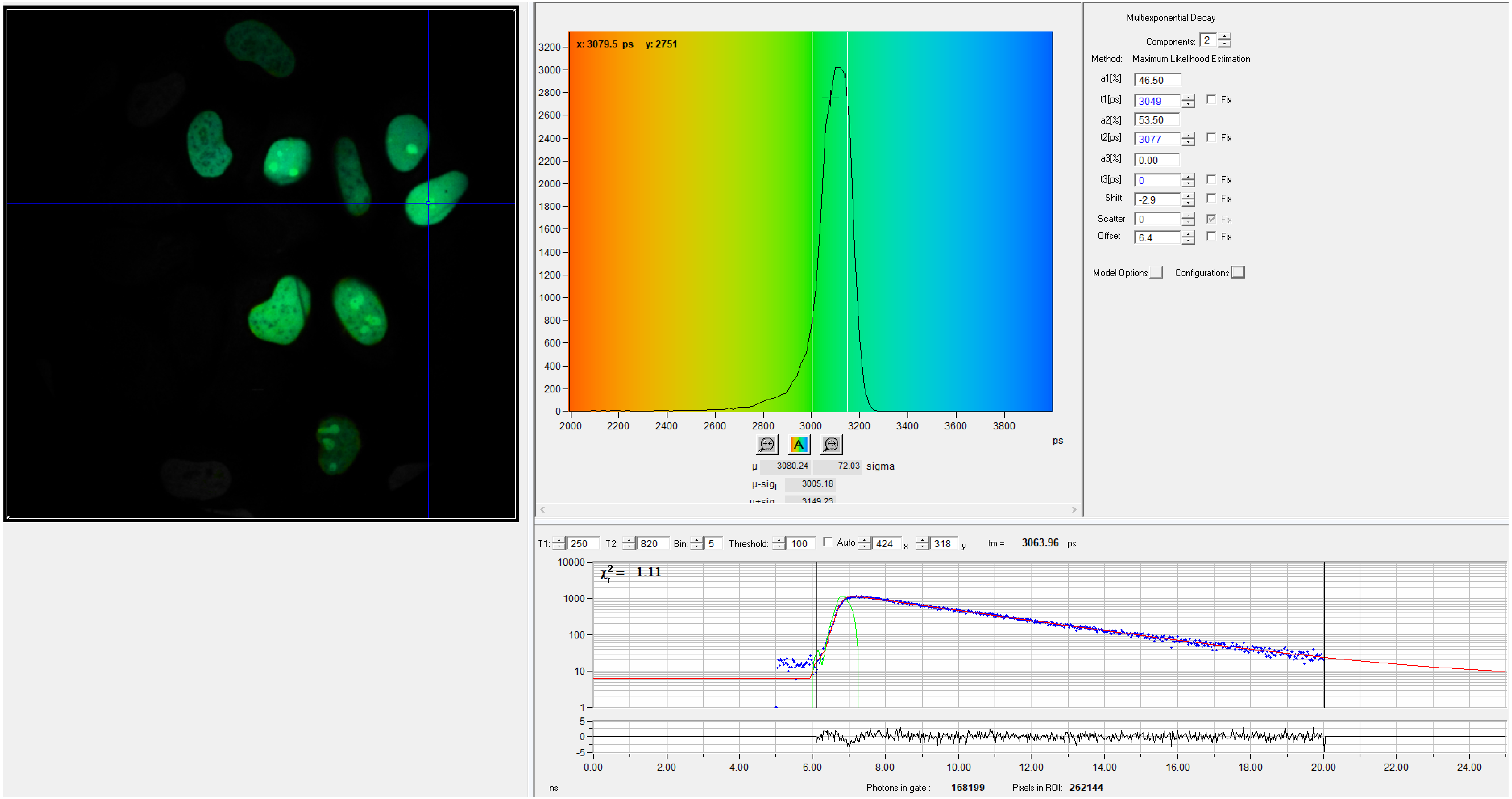

Figure S46. F62L FAST + **HBR-DOM2**; biexponential fit; τ_m color-coding. FLIM scan and corresponding time-resolved fluorescence data analysis of live HeLa cells expressing the F62L FAST variant fused to histone-2B (H2B) and stained with the **HBR-DOM2** fluorogen. A screenshot from Becker & Hickl SPCImage data acquisition and analysis window is shown. Biexponential fitting of decay data has been performed.On the left panel, there is a FLIM image of HeLa nuclei color-coded according to amplitude-weighted average fluorescence lifetime in each pixel (τ_m). A histogram on the upper right panel displays the distribution of τ_m and color legend. The table next to it (rightmost) represents a biexponential fitting model used to fit the data and fitting results. On the lower right panel, there are experimental decay data (blue dots), biexponential fit of the data (red line), instrument response function (IRF) (green line) and fitting residuals (shown in black below the main data plot).

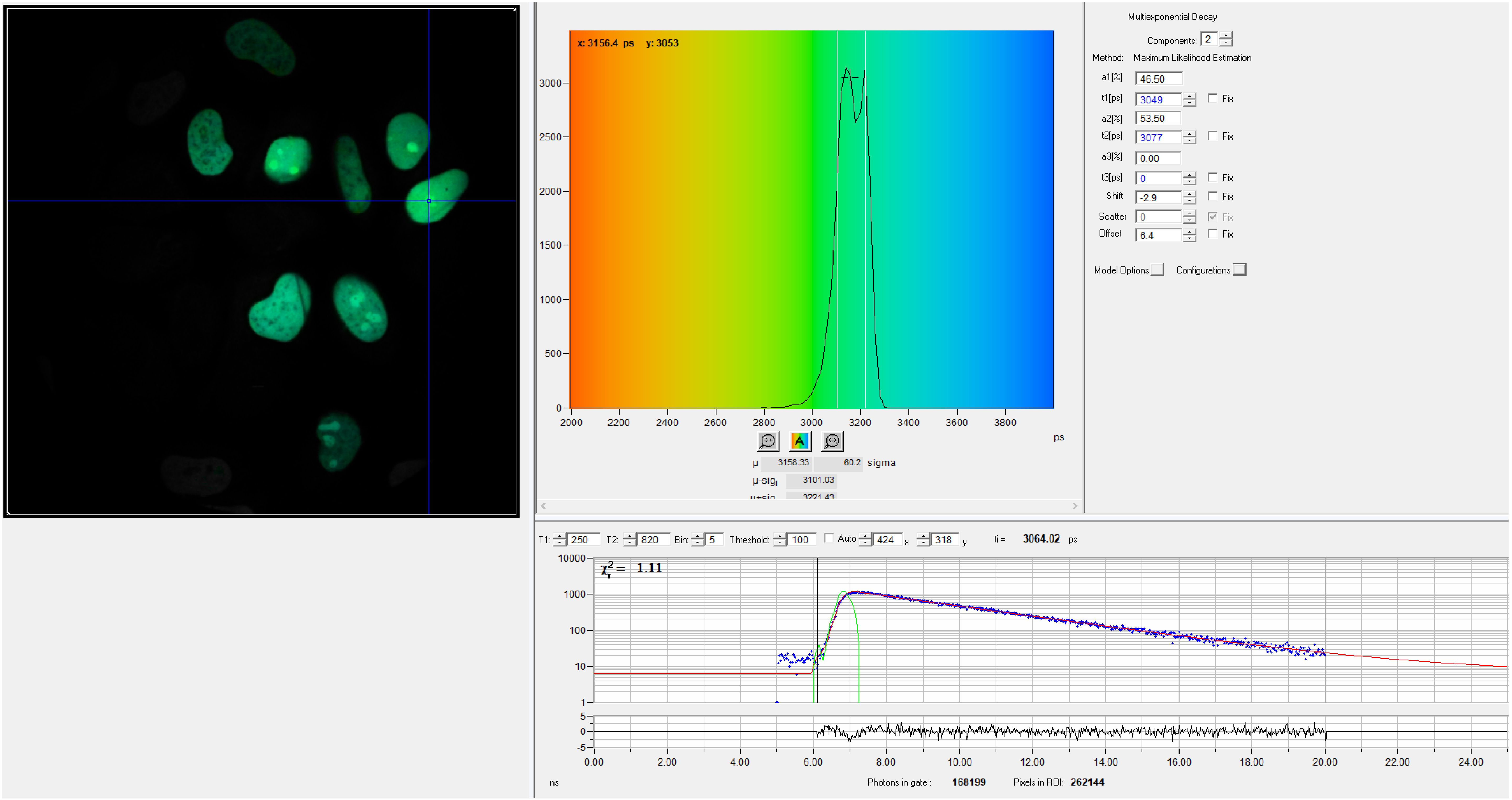

Figure S47. F62L FAST + **HBR-DOM2**; biexponential fit; τ_i color-coding. FLIM scan and corresponding time-resolved fluorescence data analysis of live HeLa cells expressing the F62L FAST variant fused to histone-2B (H2B) and stained with the **HBR-DOM2** fluorogen. A screenshot from Becker & Hickl SPCImage data acquisition and analysis window is shown. Biexponential fitting of decay data has been performed. On the left panel, there is a FLIM image of HeLa nuclei color-coded according to intensity-weighted average fluorescence lifetime in each pixel (τ_i). A histogram on the upper right panel displays the distribution of τ_i and color legend. The table next to it (rightmost) represents a biexponential fitting model used to fit the data and fitting results. On the lower right panel, there are experimental decay data (blue dots), biexponential fit of the data (red line), instrument response function (IRF) (green line) and fitting residuals (shown in black below the main data plot).

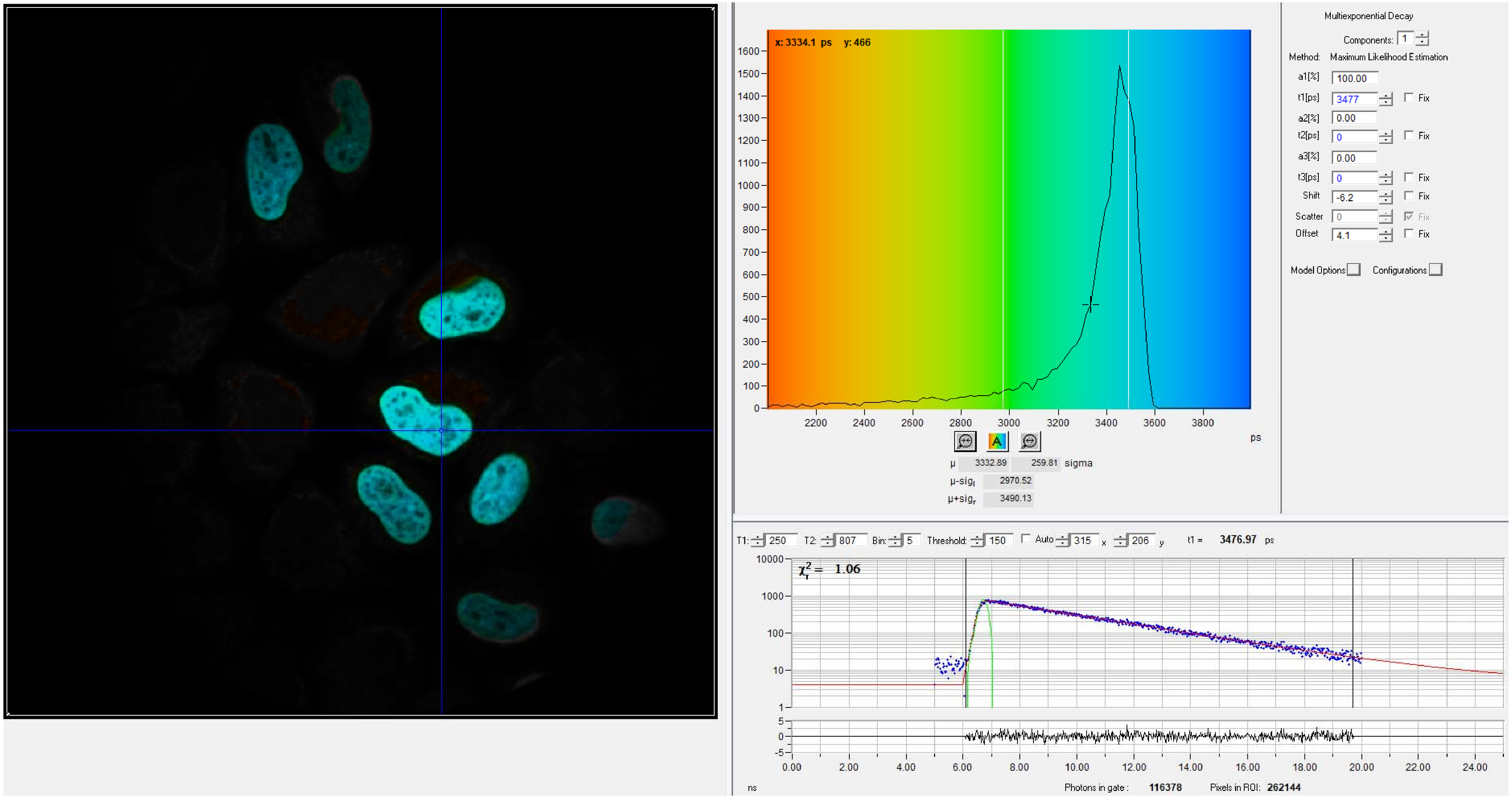

Figure S48. P68K FAST + **HBR-DOM2**; monoexponential fit; τ color-coding. FLIM scan and corresponding time-resolved fluorescence data analysis of live HeLa cells expressing the P68K FAST variant fused to histone-2B (H2B) and stained with the **HBR-DOM2** fluorogen. A screenshot from Becker & Hickl SPCImage data acquisition and analysis window is shown. Monoexponential fitting of decay data has been performed. On the left panel, there is a FLIM image of HeLa nuclei color-coded according to fluorescence lifetime in each pixel (τ). A histogram on the upper right panel displays the distribution of τ and color legend. The table next to it (rightmost) represents a monoexponential fitting model used to fit the data and fitting results. On the lower right panel, there are experimental decay data (blue dots), monoexponential fit of the data (red line), instrument response function (IRF) (green line) and fitting residuals (shown in black below the main data plot).

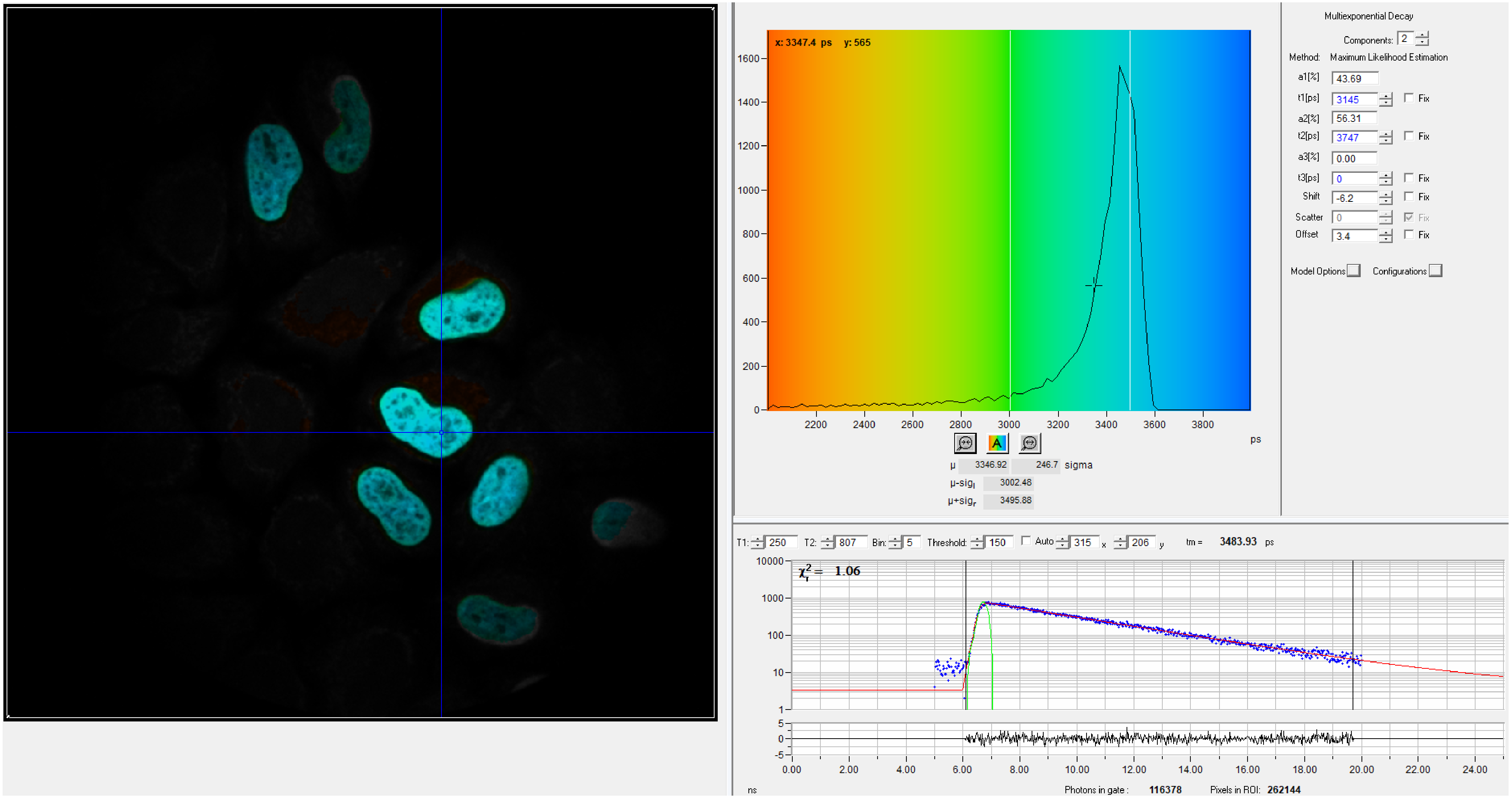

Figure S49. P68K FAST + **HBR-DOM2**; biexponential fit; τ_m color-coding. FLIM scan and corresponding time-resolved fluorescence data analysis of live HeLa cells expressing the P68K FAST variant fused to histone-2B (H2B) and stained with the **HBR-DOM2** fluorogen. A screenshot from Becker & Hickl SPCImage data acquisition and analysis window is shown. Biexponential fitting of decay data has been performed.On the left panel, there is a FLIM image of HeLa nuclei color-coded according to amplitude-weighted average fluorescence lifetime in each pixel (τ_m). A histogram on the upper right panel displays the distribution of τ_m and color legend. The table next to it (rightmost) represents a biexponential fitting model used to fit the data and fitting results. On the lower right panel, there are experimental decay data (blue dots), biexponential fit of the data (red line), instrument response function (IRF) (green line) and fitting residuals (shown in black below the main data plot).

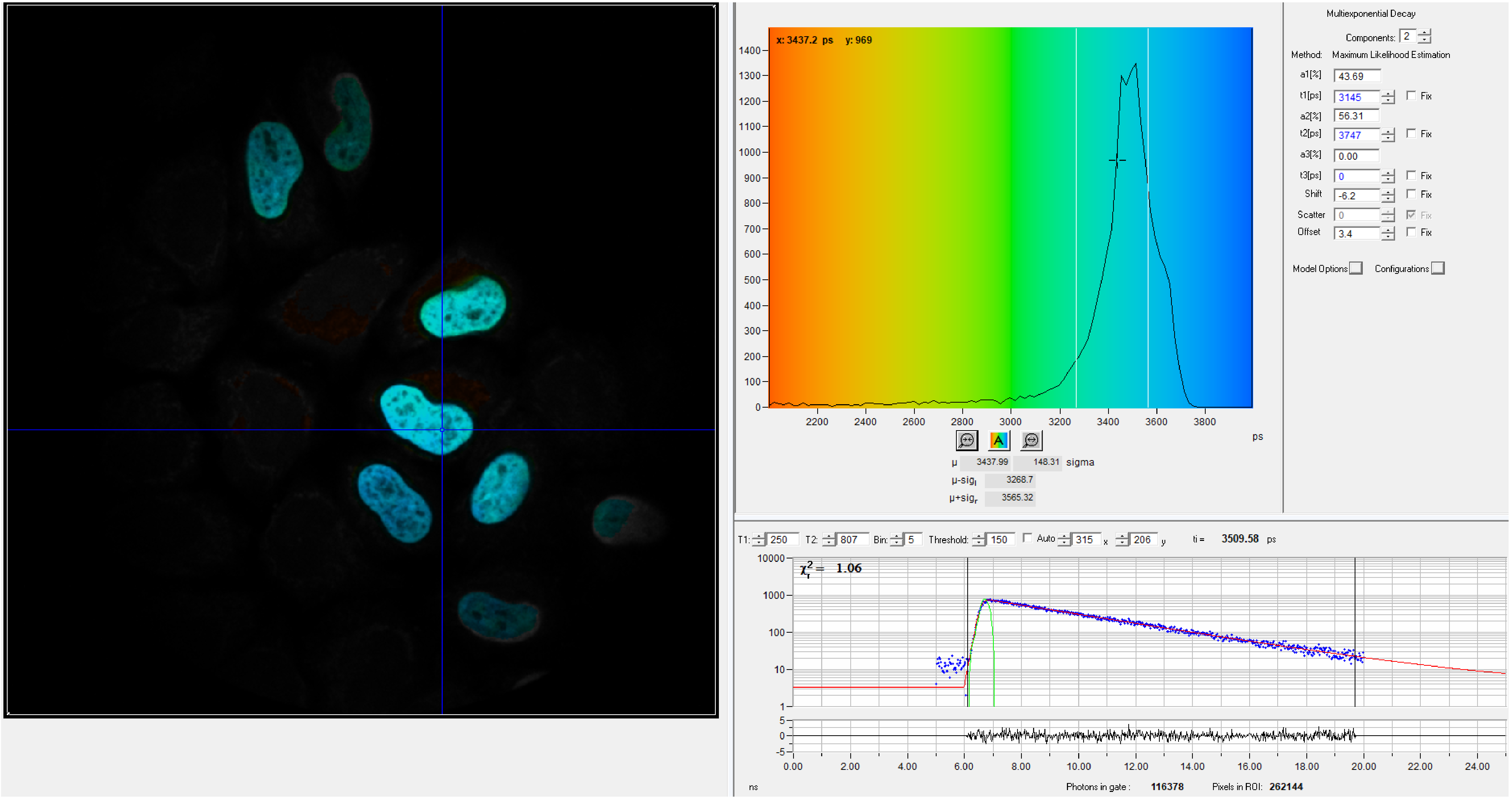

Figure S50. P68K FAST + **HBR-DOM2**; biexponential fit; τ_i color-coding. FLIM scan and corresponding time-resolved fluorescence data analysis of live HeLa cells expressing the P68K FAST variant fused to histone-2B (H2B) and stained with the **HBR-DOM2** fluorogen. A screenshot from Becker & Hickl SPCImage data acquisition and analysis window is shown. Biexponential fitting of decay data has been performed. On the left panel, there is a FLIM image of HeLa nuclei color-coded according to intensity-weighted average fluorescence lifetime in each pixel (τ_i). A histogram on the upper right panel displays the distribution of τ_i and color legend. The table next to it (rightmost) represents a biexponential fitting model used to fit the data and fitting results. On the lower right panel, there are experimental decay data (blue dots), biexponential fit of the data (red line), instrument response function (IRF) (green line) and fitting residuals (shown in black below the main data plot).

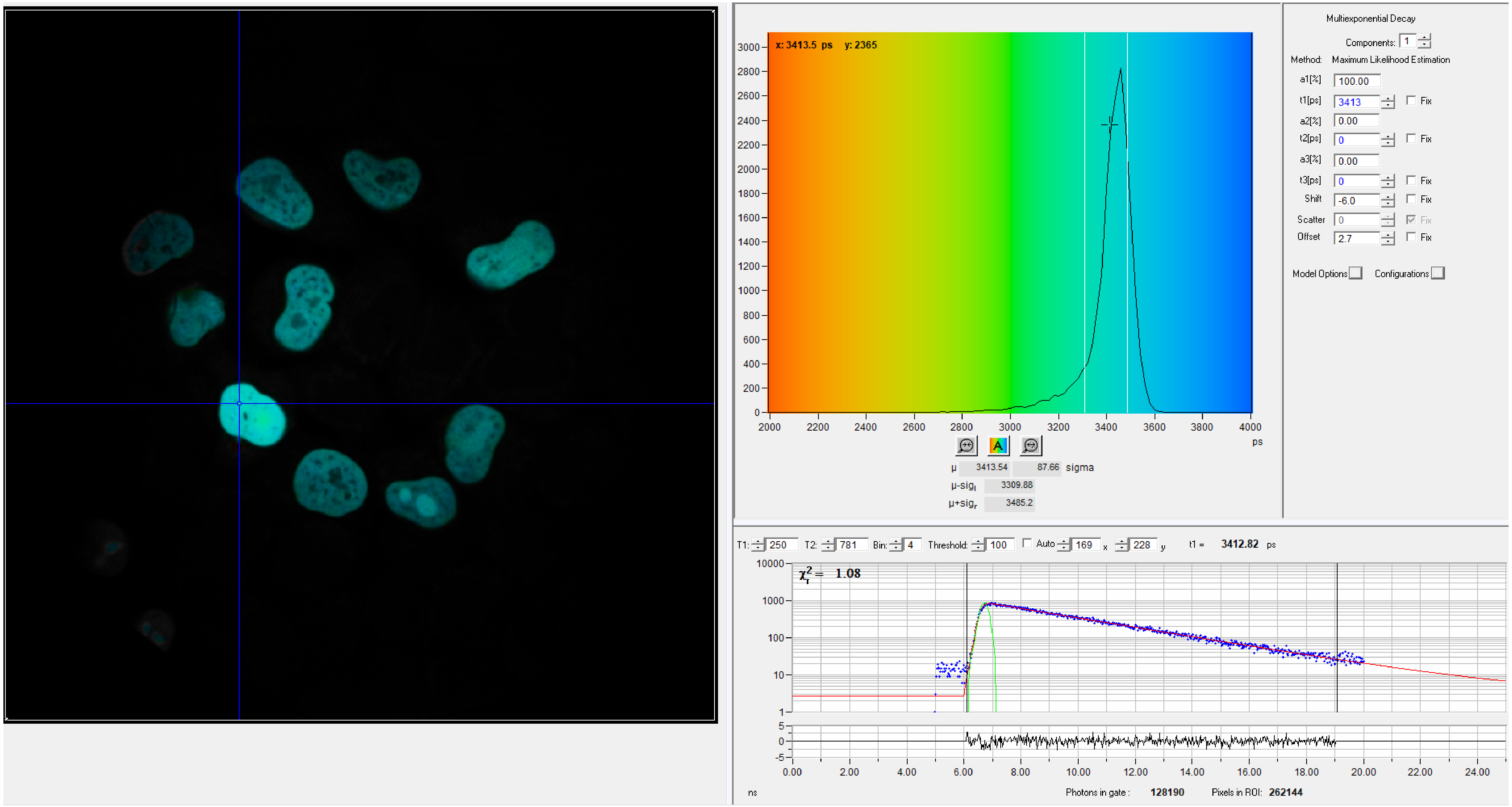

Figure S51. P68T FAST + **HBR-DOM2**; monoexponential fit; τ color-coding. FLIM scan and corresponding time-resolved fluorescence data analysis of live HeLa cells expressing the P68T FAST variant fused to histone-2B (H2B) and stained with the **HBR-DOM2** fluorogen. A screenshot from Becker & Hickl SPCImage data acquisition and analysis window is shown. Monoexponential fitting of decay data has been performed. On the left panel, there is a FLIM image of HeLa nuclei color-coded according to fluorescence lifetime in each pixel (τ). A histogram on the upper right panel displays the distribution of τ and color legend. The table next to it (rightmost) represents a monoexponential fitting model used to fit the data and fitting results. On the lower right panel, there are experimental decay data (blue dots), monoexponential fit of the data (red line), instrument response function (IRF) (green line) and fitting residuals (shown in black below the main data plot).

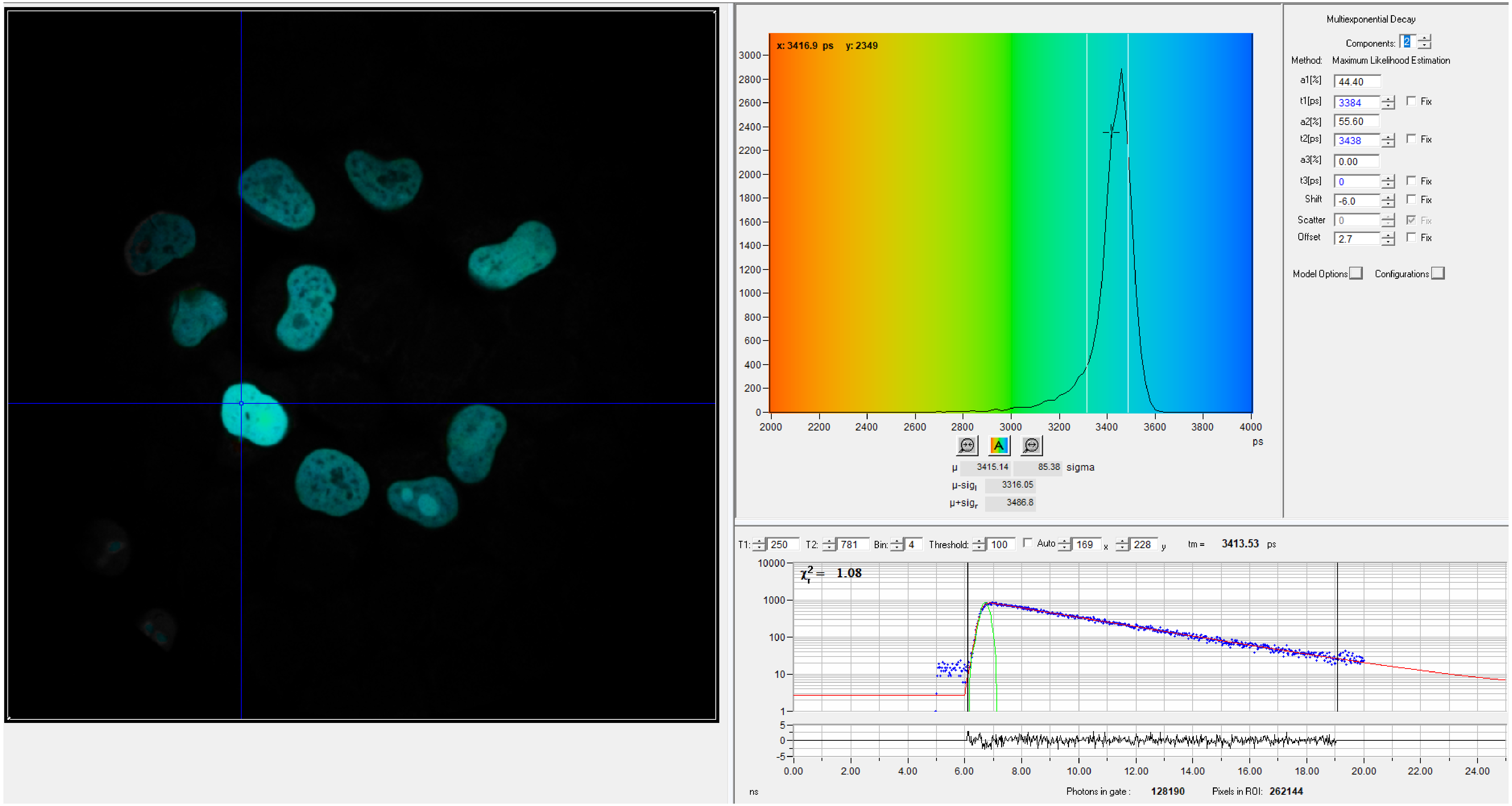

Figure S52. P68T FAST + **HBR-DOM2**; biexponential fit; τ_m color-coding. FLIM scan and corresponding time-resolved fluorescence data analysis of live HeLa cells expressing the P68T FAST variant fused to histone-2B (H2B) and stained with the **HBR-DOM2** fluorogen. A screenshot from Becker & Hickl SPCImage data acquisition and analysis window is shown. Biexponential fitting of decay data has been performed.On the left panel, there is a FLIM image of HeLa nuclei color-coded according to amplitude-weighted average fluorescence lifetime in each pixel (τ_m). A histogram on the upper right panel displays the distribution of τ_m and color legend. The table next to it (rightmost) represents a biexponential fitting model used to fit the data and fitting results. On the lower right panel, there are experimental decay data (blue dots), biexponential fit of the data (red line), instrument response function (IRF) (green line) and fitting residuals (shown in black below the main data plot).

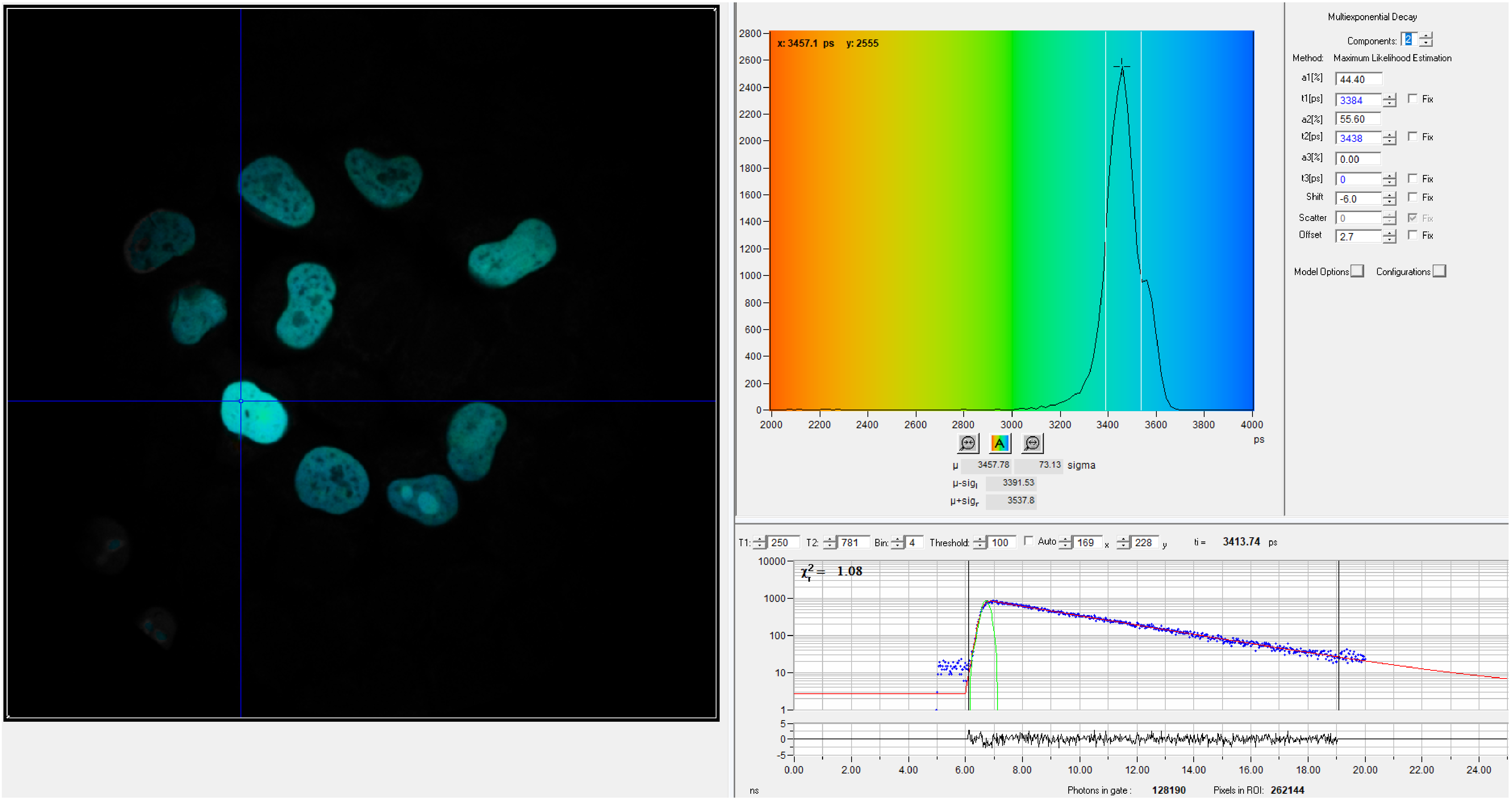

Figure S53. P68T FAST + **HBR-DOM2**; biexponential fit; τ_i color-coding. FLIM scan and corresponding time-resolved fluorescence data analysis of live HeLa cells expressing the P68T FAST variant fused to histone-2B (H2B) and stained with the **HBR-DOM2** fluorogen. A screenshot from Becker & Hickl SPCImage data acquisition and analysis window is shown. Biexponential fitting of decay data has been performed. On the left panel, there is a FLIM image of HeLa nuclei color-coded according to intensity-weighted average fluorescence lifetime in each pixel (τ_i). A histogram on the upper right panel displays the distribution of τ_i and color legend. The table next to it (rightmost) represents a biexponential fitting model used to fit the data and fitting results. On the lower right panel, there are experimental decay data (blue dots), biexponential fit of the data (red line), instrument response function (IRF) (green line) and fitting residuals (shown in black below the main data plot).

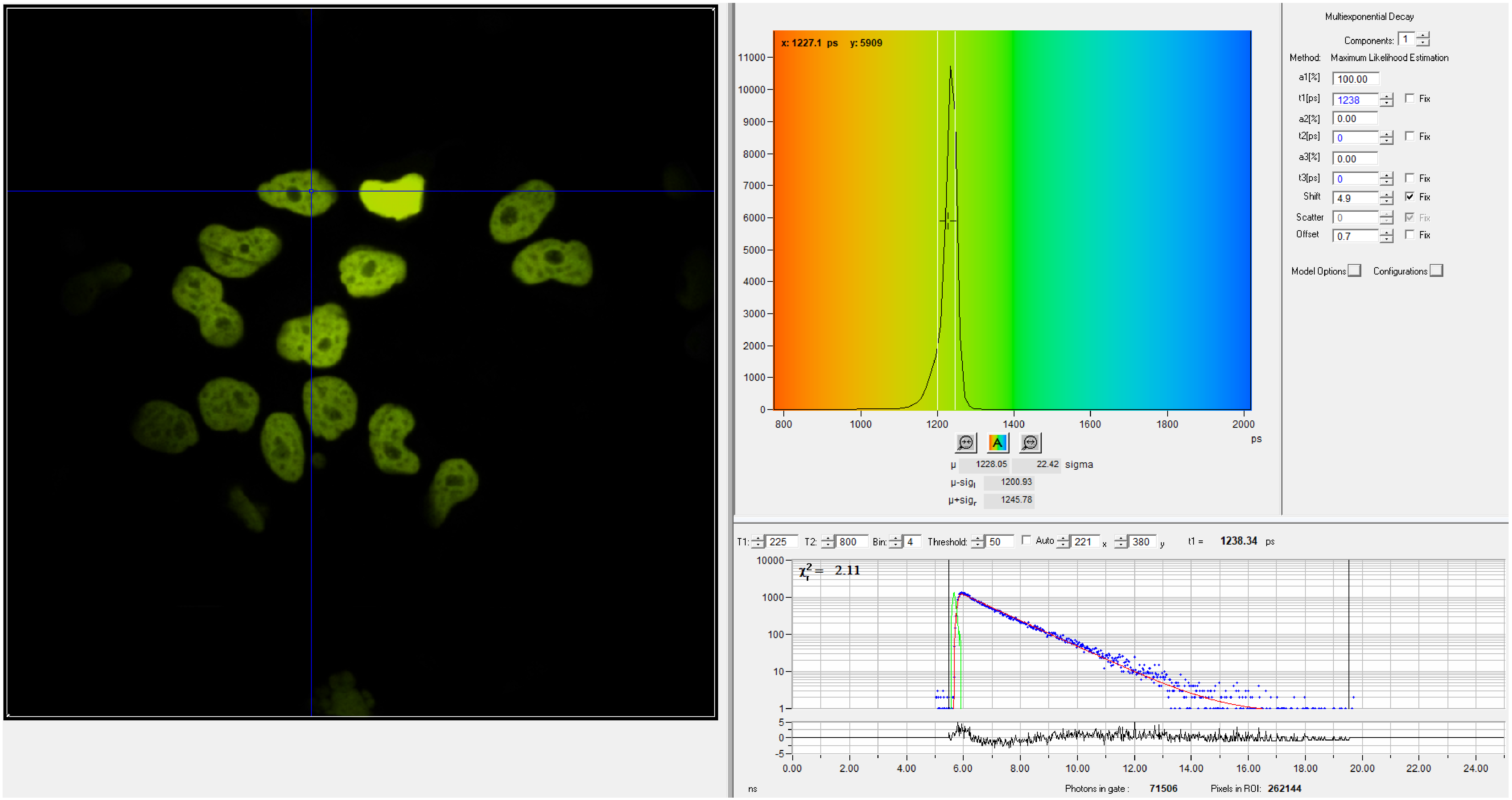

Figure S54. R52K FAST + **HMBR**; monoexponential fit; τ color-coding. FLIM scan and corresponding time-resolved fluorescence data analysis of live HeLa cells expressing the R52K FAST variant fused to histone-2B (H2B) and stained with the **HMBR** fluorogen. A screenshot from Becker & Hickl SPCImage data acquisition and analysis window is shown. Monoexponential fitting of decay data has been performed. On the left panel, there is a FLIM image of HeLa nuclei color-coded according to fluorescence lifetime in each pixel (τ). A histogram on the upper right panel displays the distribution of τ and color legend. The table next to it (rightmost) represents a monoexponential fitting model used to fit the data and fitting results. On the lower right panel, there are experimental decay data (blue dots), monoexponential fit of the data (red line), instrument response function (IRF) (green line) and fitting residuals (shown in black below the main data plot).

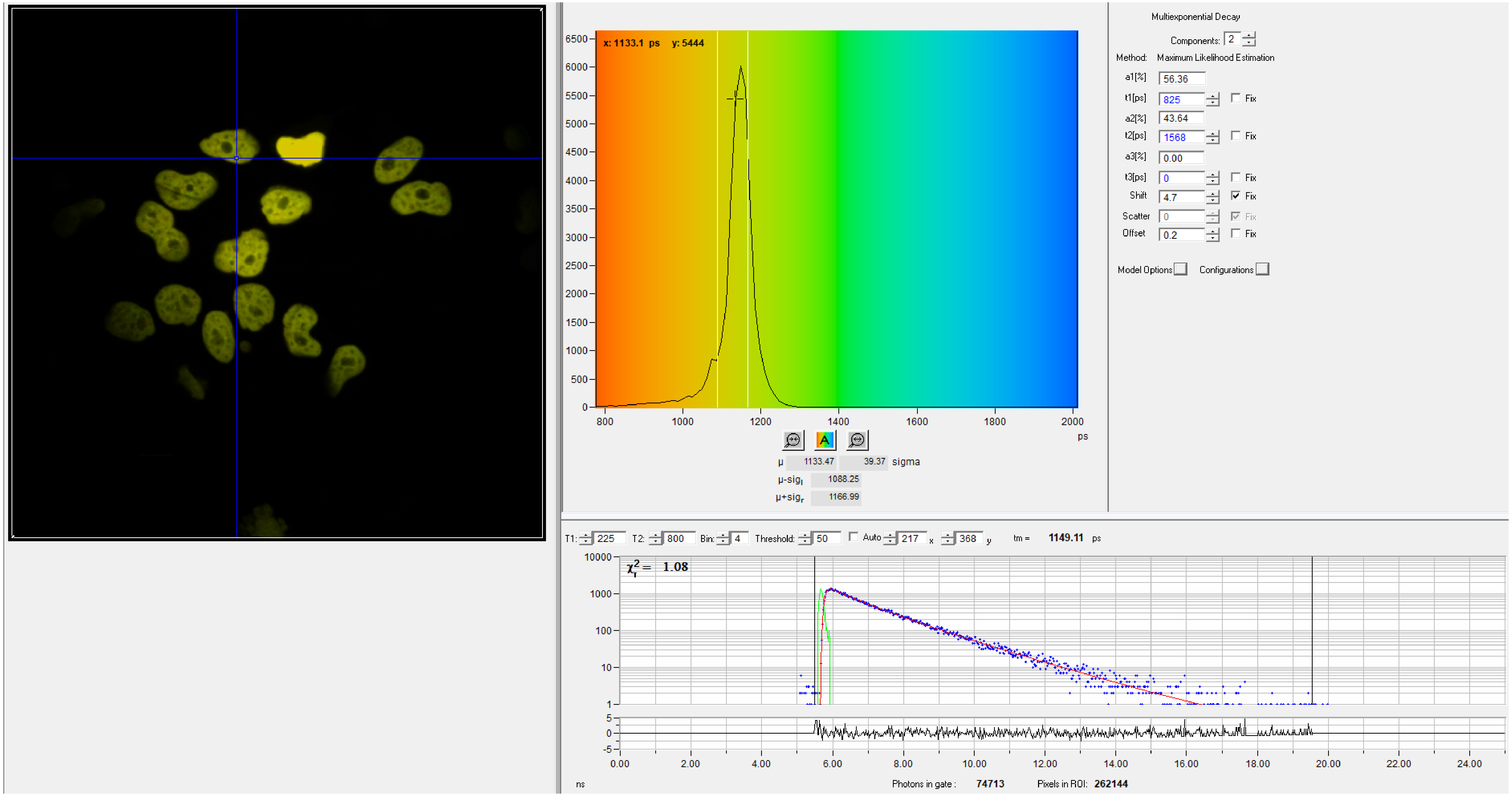

Figure S55. R52K FAST + **HMBR**; biexponential fit; τ_m color-coding. FLIM scan and corresponding time-resolved fluorescence data analysis of live HeLa cells expressing the R52K FAST variant fused to histone-2B (H2B) and stained with the **HMBR** fluorogen. A screenshot from Becker & Hickl SPCImage data acquisition and analysis window is shown. Biexponential fitting of decay data has been performed.On the left panel, there is a FLIM image of HeLa nuclei color-coded according to amplitude-weighted average fluorescence lifetime in each pixel (τ_m). A histogram on the upper right panel displays the distribution of τ_m and color legend. The table next to it (rightmost) represents a biexponential fitting model used to fit the data and fitting results. On the lower right panel, there are experimental decay data (blue dots), biexponential fit of the data (red line), instrument response function (IRF) (green line) and fitting residuals (shown in black below the main data plot).

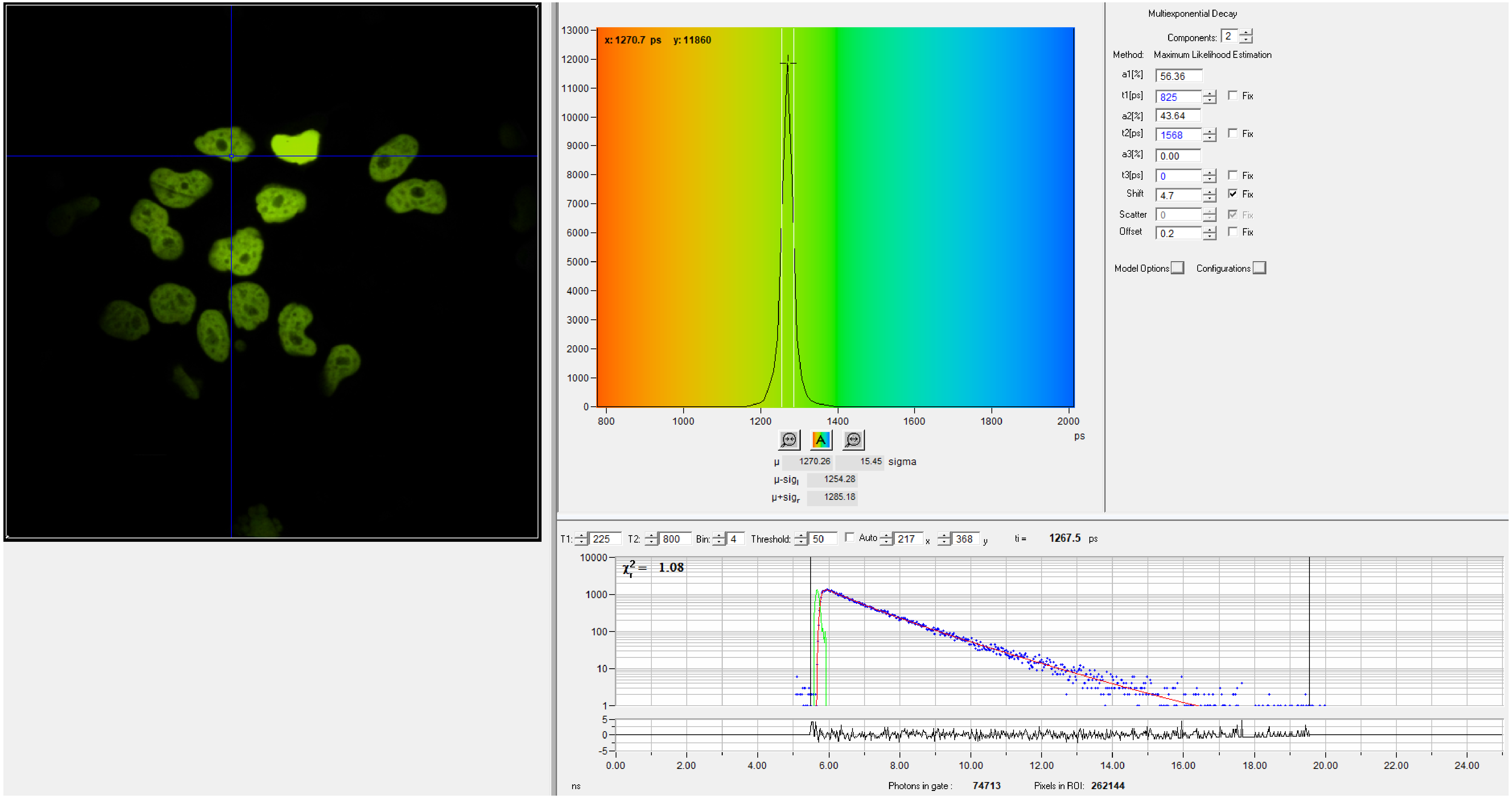

Figure S56. R52K FAST + **HMBR**; biexponential fit; τ_i color-coding. FLIM scan and corresponding time-resolved fluorescence data analysis of live HeLa cells expressing the R52K FAST variant fused to histone-2B (H2B) and stained with the **HMBR** fluorogen. A screenshot from Becker & Hickl SPCImage data acquisition and analysis window is shown. Biexponential fitting of decay data has been performed. On the left panel, there is a FLIM image of HeLa nuclei color-coded according to intensity-weighted average fluorescence lifetime in each pixel (τ_i). A histogram on the upper right panel displays the distribution of τ_i and color legend. The table next to it (rightmost) represents a biexponential fitting model used to fit the data and fitting results. On the lower right panel, there are experimental decay data (blue dots), biexponential fit of the data (red line), instrument response function (IRF) (green line) and fitting residuals (shown in black below the main data plot).

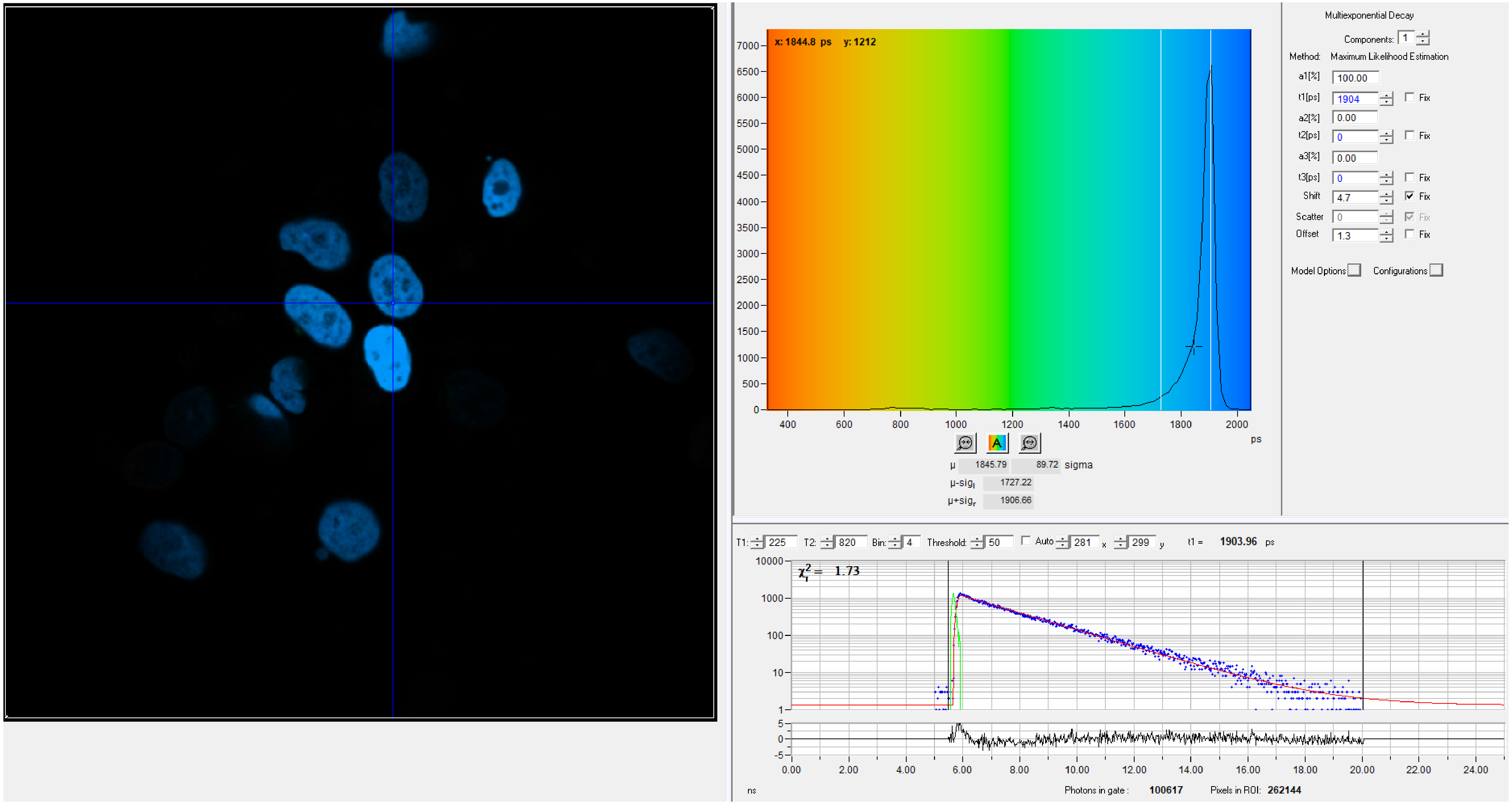

Figure S57. F62L FAST + **HMBR**; monoexponential fit; τ color-coding. FLIM scan and corresponding time-resolved fluorescence data analysis of live HeLa cells expressing the F62L FAST variant fused to histone-2B (H2B) and stained with the **HMBR** fluorogen. A screenshot from Becker & Hickl SPCImage data acquisition and analysis window is shown. Monoexponential fitting of decay data has been performed. On the left panel, there is a FLIM image of HeLa nuclei color-coded according to fluorescence lifetime in each pixel (τ). A histogram on the upper right panel displays the distribution of τ and color legend. The table next to it (rightmost) represents a monoexponential fitting model used to fit the data and fitting results. On the lower right panel, there are experimental decay data (blue dots), monoexponential fit of the data (red line), instrument response function (IRF) (green line) and fitting residuals (shown in black below the main data plot).

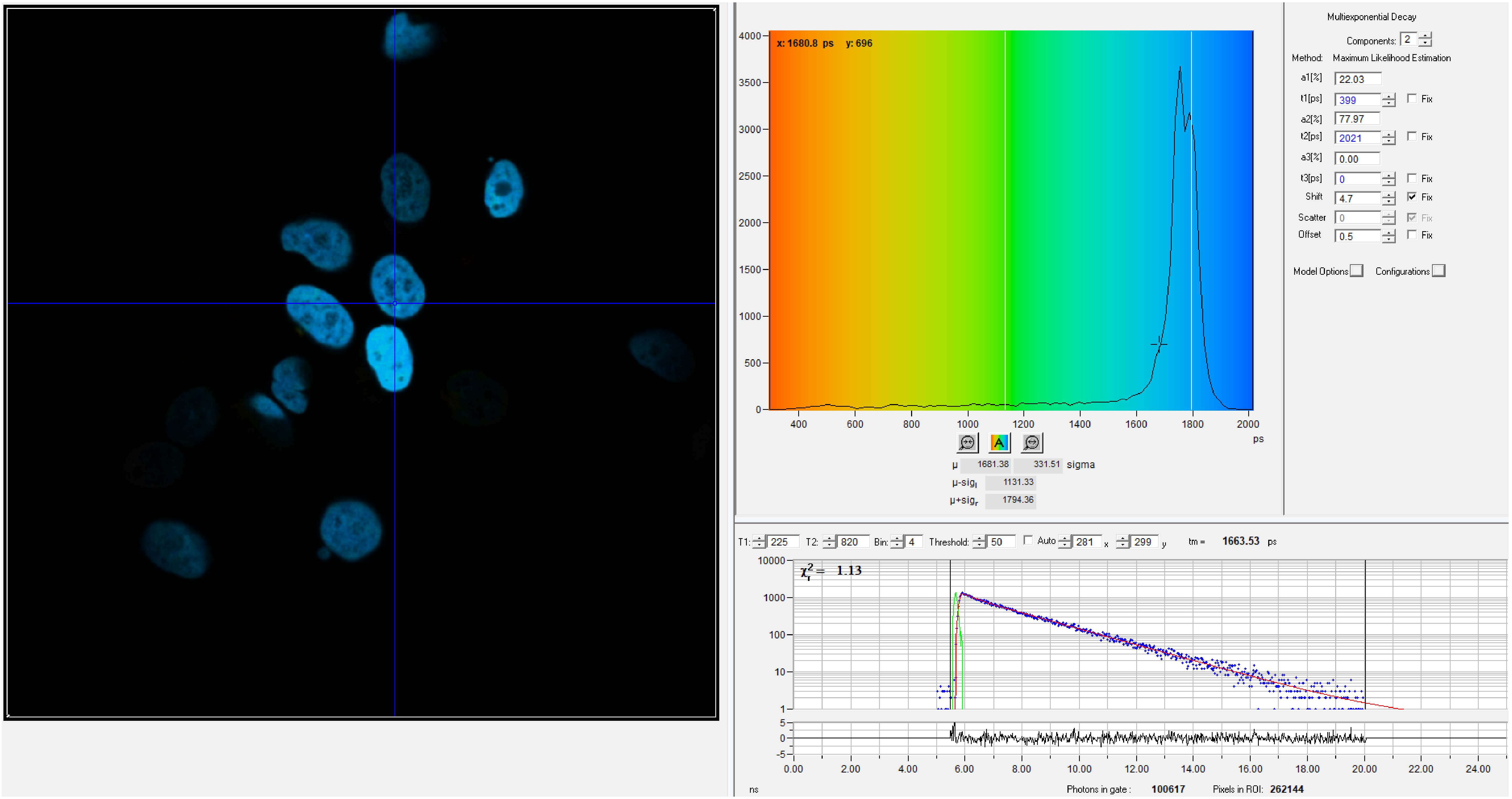

Figure S58. F62L FAST + **HMBR**; biexponential fit; τ_m color-coding. FLIM scan and corresponding time-resolved fluorescence data analysis of live HeLa cells expressing the F62L FAST variant fused to histone-2B (H2B) and stained with the **HMBR** fluorogen. A screenshot from Becker & Hickl SPCImage data acquisition and analysis window is shown. Biexponential fitting of decay data has been performed.On the left panel, there is a FLIM image of HeLa nuclei color-coded according to amplitude-weighted average fluorescence lifetime in each pixel (τ_m). A histogram on the upper right panel displays the distribution of τ_m and color legend. The table next to it (rightmost) represents a biexponential fitting model used to fit the data and fitting results. On the lower right panel, there are experimental decay data (blue dots), biexponential fit of the data (red line), instrument response function (IRF) (green line) and fitting residuals (shown in black below the main data plot).

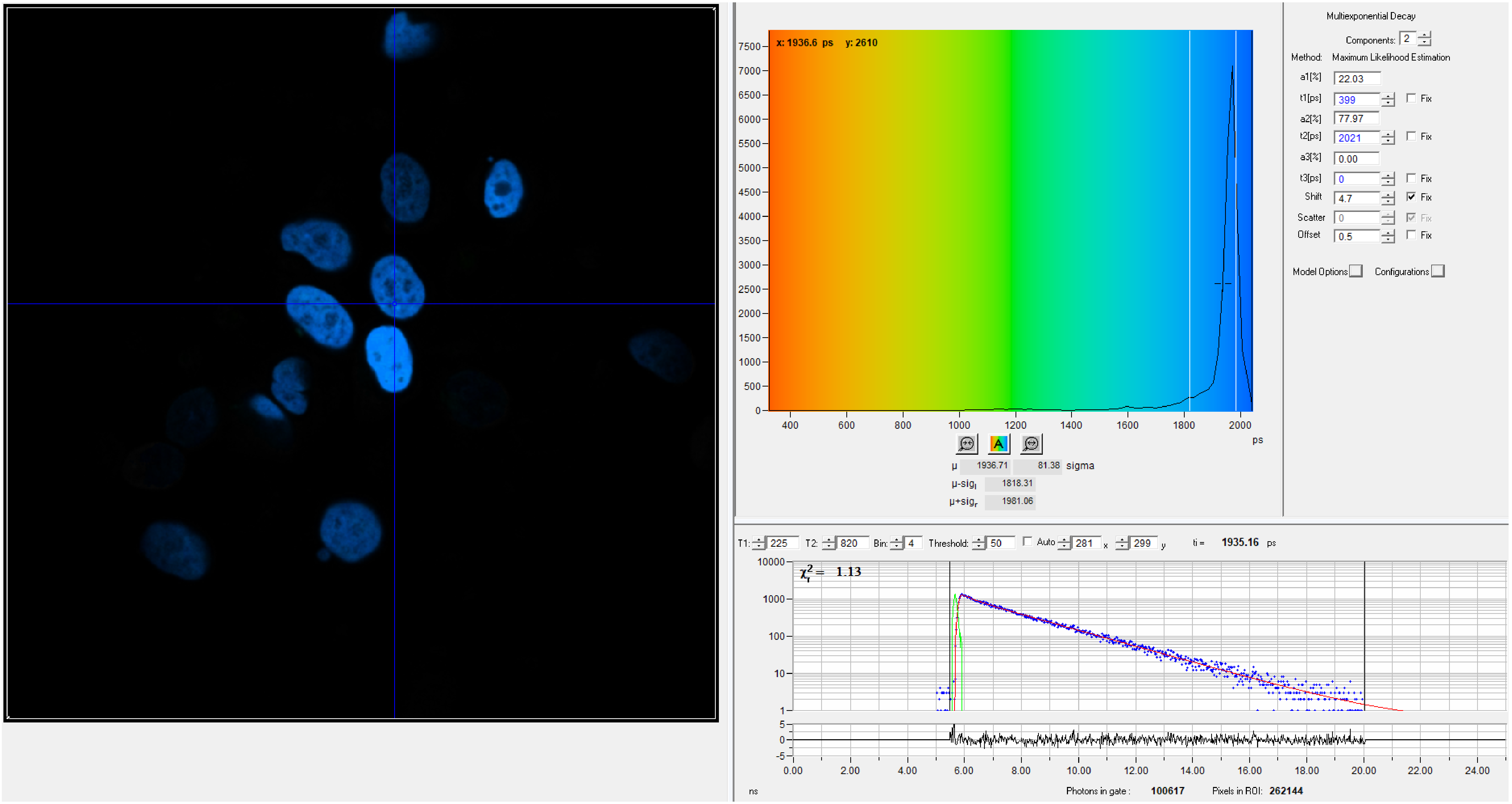

Figure S59. F62L FAST + **HMBR**; biexponential fit; τ_i color-coding. FLIM scan and corresponding time-resolved fluorescence data analysis of live HeLa cells expressing the F62L FAST variant fused to histone-2B (H2B) and stained with the **HMBR** fluorogen. A screenshot from Becker & Hickl SPCImage data acquisition and analysis window is shown. Biexponential fitting of decay data has been performed. On the left panel, there is a FLIM image of HeLa nuclei color-coded according to intensity-weighted average fluorescence lifetime in each pixel (τ_i). A histogram on the upper right panel displays the distribution of τ_i and color legend. The table next to it (rightmost) represents a biexponential fitting model used to fit the data and fitting results. On the lower right panel, there are experimental decay data (blue dots), biexponential fit of the data (red line), instrument response function (IRF) (green line) and fitting residuals (shown in black below the main data plot).

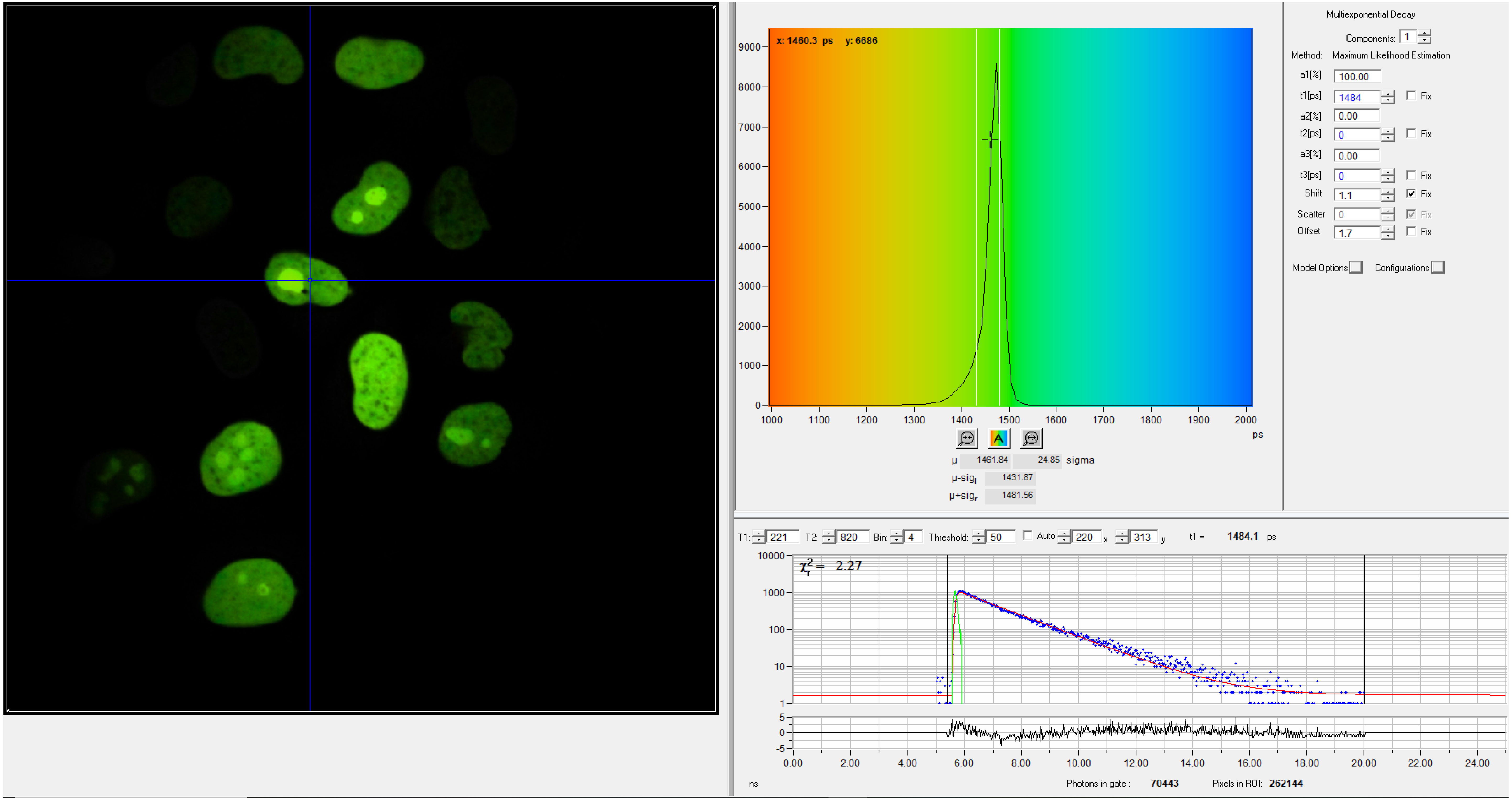

Figure S60. P68K FAST + **HMBR**; monoexponential fit; τ color-coding. FLIM scan and corresponding time-resolved fluorescence data analysis of live HeLa cells expressing the P68K FAST variant fused to histone-2B (H2B) and stained with the **HMBR** fluorogen. A screenshot from Becker & Hickl SPCImage data acquisition and analysis window is shown. Monoexponential fitting of decay data has been performed. On the left panel, there is a FLIM image of HeLa nuclei color-coded according to fluorescence lifetime in each pixel (τ). A histogram on the upper right panel displays the distribution of τ and color legend. The table next to it (rightmost) represents a monoexponential fitting model used to fit the data and fitting results. On the lower right panel, there are experimental decay data (blue dots), monoexponential fit of the data (red line), instrument response function (IRF) (green line) and fitting residuals (shown in black below the main data plot).

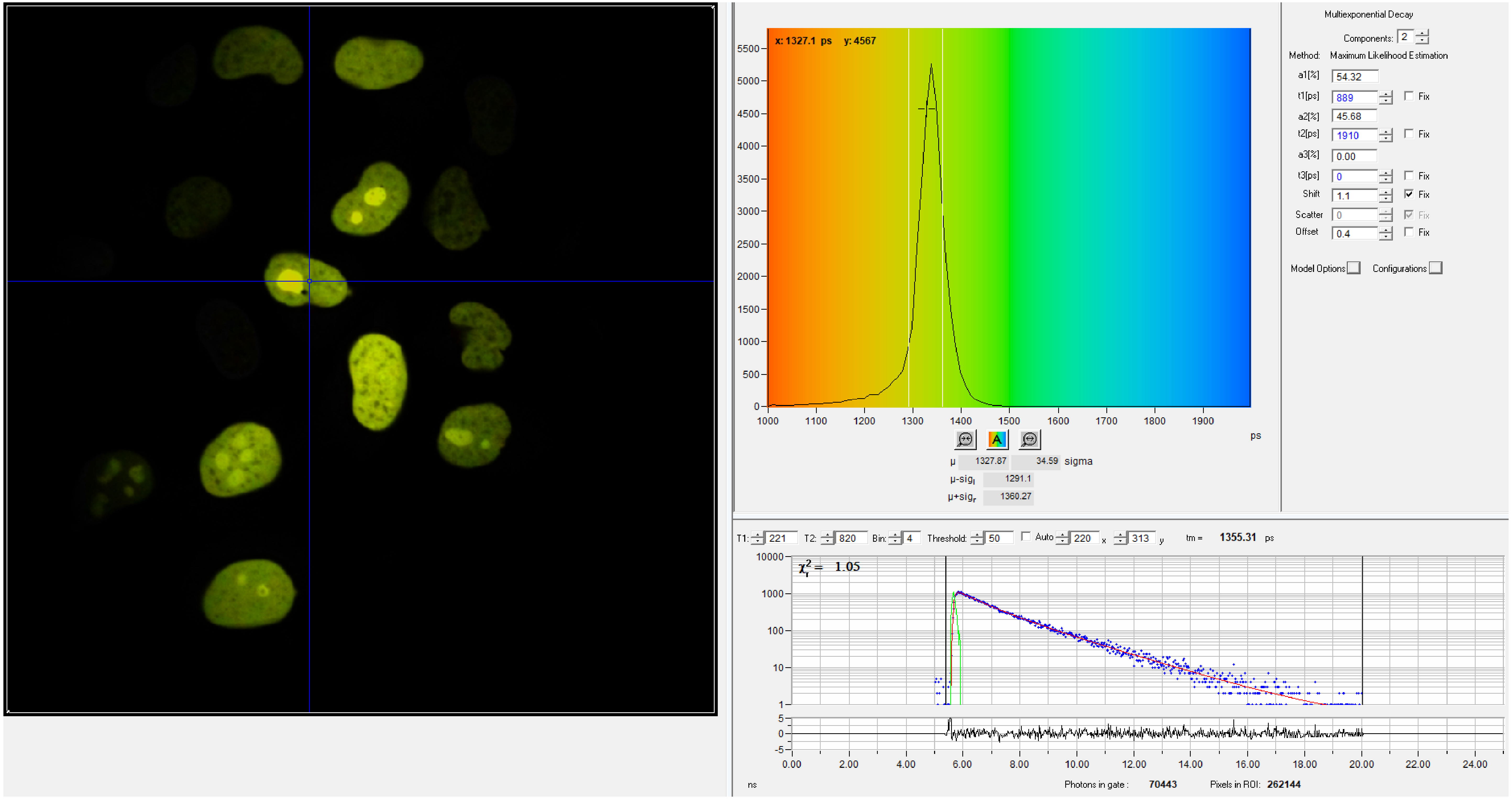

Figure S61. P68K FAST + **HMBR**; biexponential fit; τ_m color-coding. FLIM scan and corresponding time-resolved fluorescence data analysis of live HeLa cells expressing the P68K FAST variant fused to histone-2B (H2B) and stained with the **HMBR** fluorogen. A screenshot from Becker & Hickl SPCImage data acquisition and analysis window is shown. Biexponential fitting of decay data has been performed.On the left panel, there is a FLIM image of HeLa nuclei color-coded according to amplitude-weighted average fluorescence lifetime in each pixel (τ_m). A histogram on the upper right panel displays the distribution of τ_m and color legend. The table next to it (rightmost) represents a biexponential fitting model used to fit the data and fitting results. On the lower right panel, there are experimental decay data (blue dots), biexponential fit of the data (red line), instrument response function (IRF) (green line) and fitting residuals (shown in black below the main data plot).

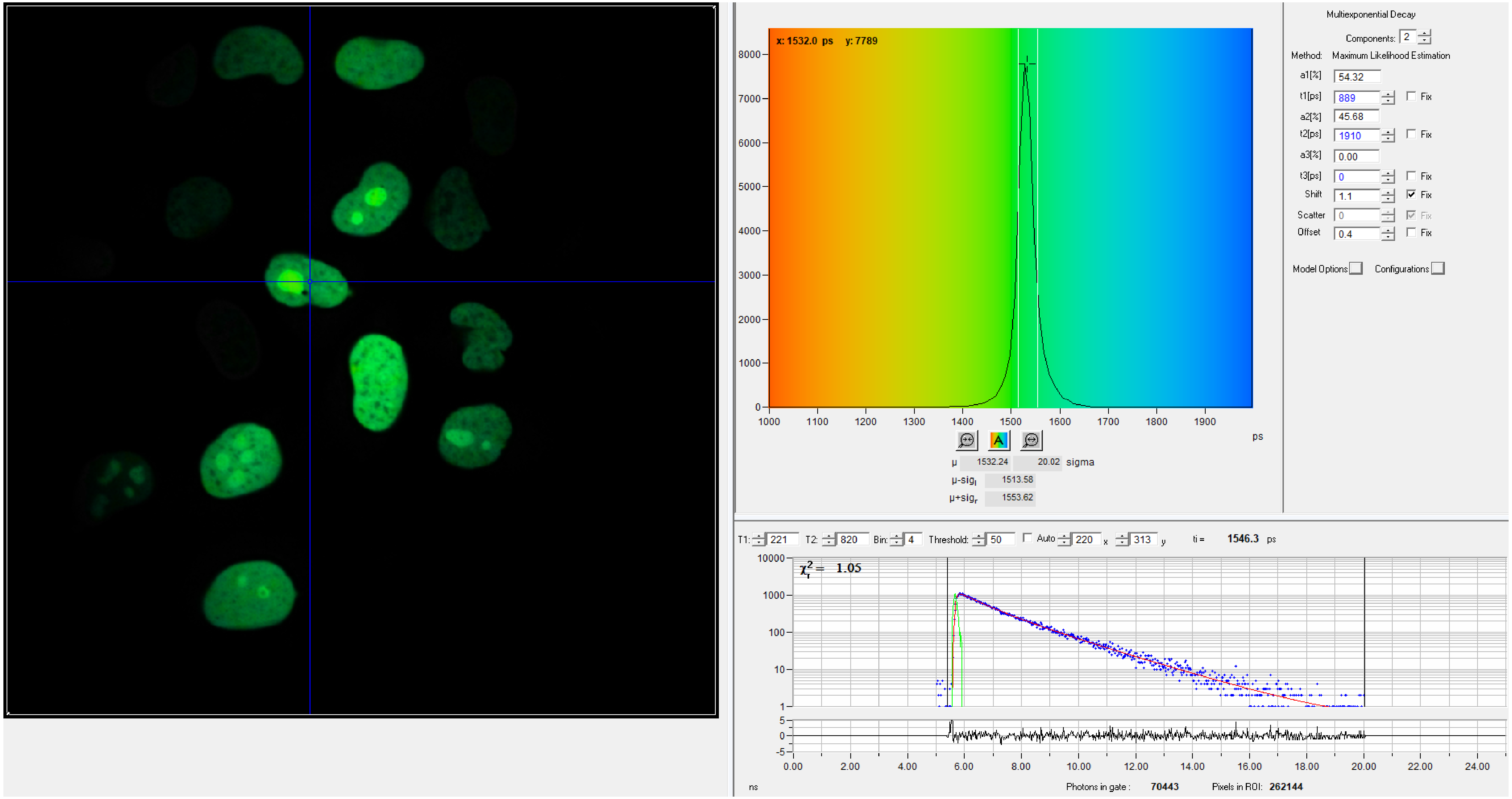

Figure S62. P68K FAST + **HMBR**; biexponential fit; τ_i color-coding. FLIM scan and corresponding time-resolved fluorescence data analysis of live HeLa cells expressing the P68K FAST variant fused to histone-2B (H2B) and stained with the **HMBR** fluorogen. A screenshot from Becker & Hickl SPCImage data acquisition and analysis window is shown. Biexponential fitting of decay data has been performed. On the left panel, there is a FLIM image of HeLa nuclei color-coded according to intensity-weighted average fluorescence lifetime in each pixel (τ_i). A histogram on the upper right panel displays the distribution of τ_i and color legend. The table next to it (rightmost) represents a biexponential fitting model used to fit the data and fitting results. On the lower right panel, there are experimental decay data (blue dots), biexponential fit of the data (red line), instrument response function (IRF) (green line) and fitting residuals (shown in black below the main data plot).

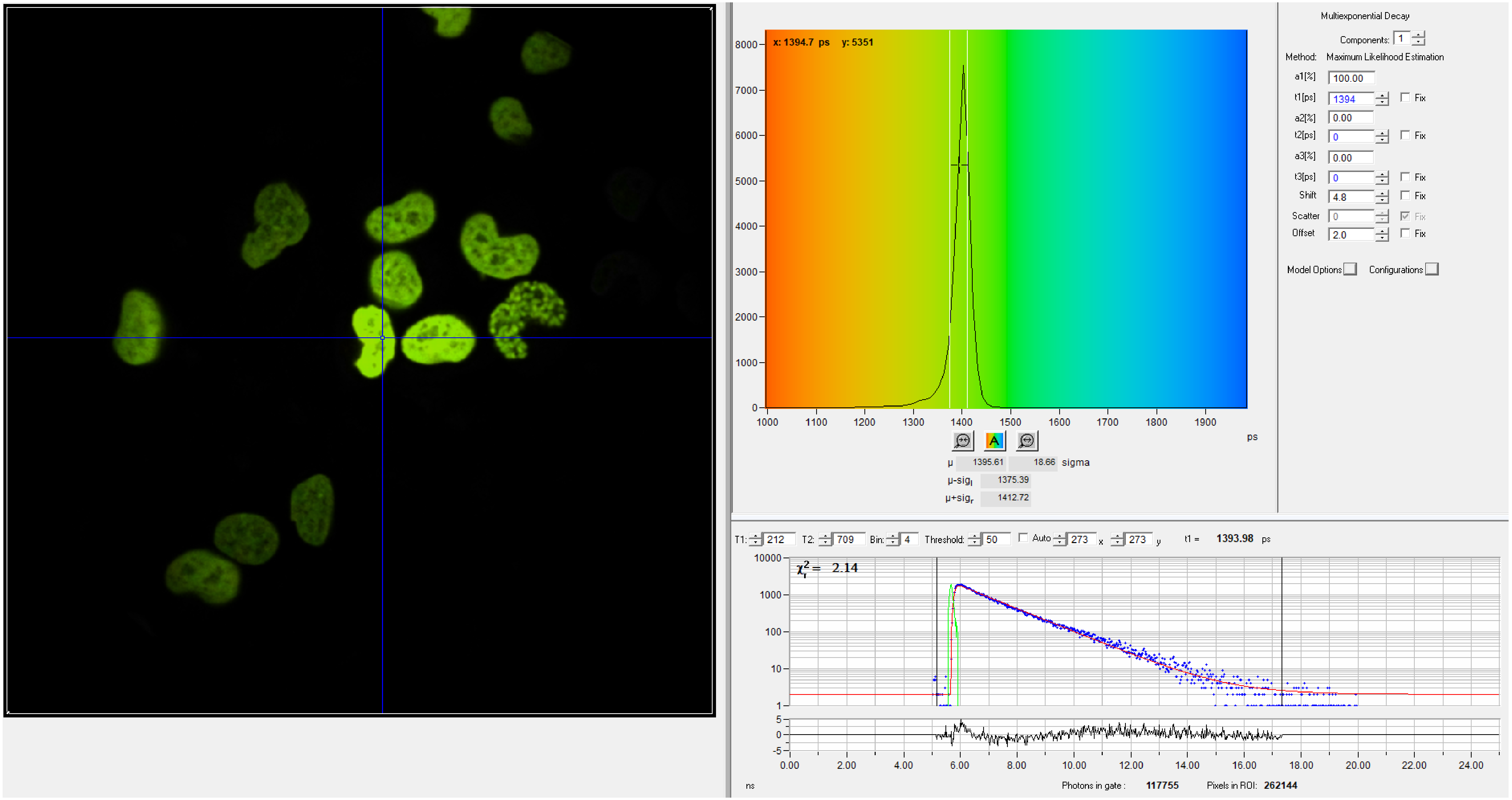

Figure S63. P68T FAST + **HMBR**; monoexponential fit; τ color-coding. FLIM scan and corresponding time-resolved fluorescence data analysis of live HeLa cells expressing the P68T FAST variant fused to histone-2B (H2B) and stained with the **HMBR** fluorogen. A screenshot from Becker & Hickl SPCImage data acquisition and analysis window is shown. Monoexponential fitting of decay data has been performed. On the left panel, there is a FLIM image of HeLa nuclei color-coded according to fluorescence lifetime in each pixel (τ). A histogram on the upper right panel displays the distribution of τ and color legend. The table next to it (rightmost) represents a monoexponential fitting model used to fit the data and fitting results. On the lower right panel, there are experimental decay data (blue dots), monoexponential fit of the data (red line), instrument response function (IRF) (green line) and fitting residuals (shown in black below the main data plot).

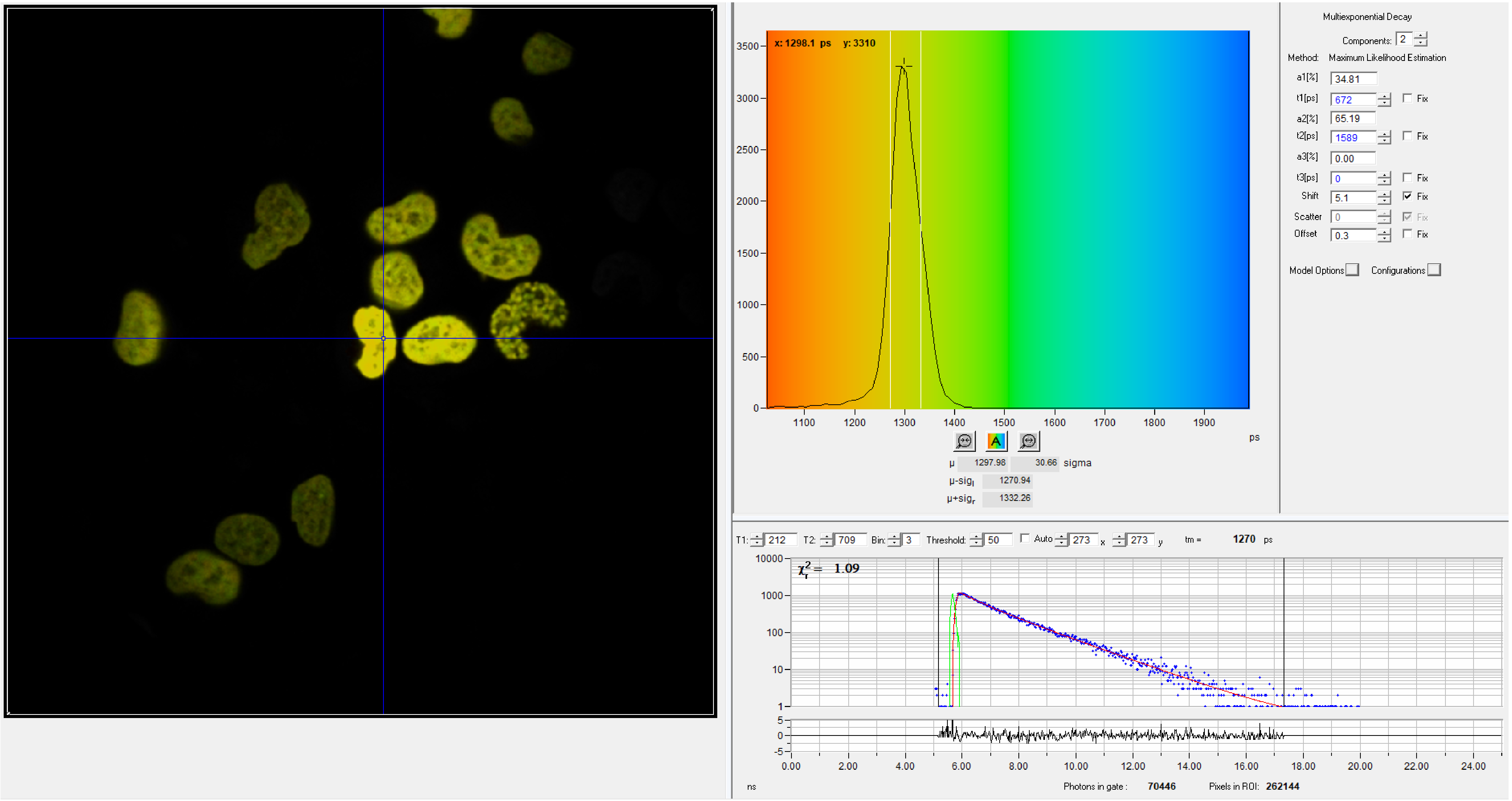

Figure S64. P68T FAST + **HMBR**; biexponential fit; τ_m color-coding. FLIM scan and corresponding time-resolved fluorescence data analysis of live HeLa cells expressing the P68T FAST variant fused to histone-2B (H2B) and stained with the **HMBR** fluorogen. A screenshot from Becker & Hickl SPCImage data acquisition and analysis window is shown. Biexponential fitting of decay data has been performed.On the left panel, there is a FLIM image of HeLa nuclei color-coded according to amplitude-weighted average fluorescence lifetime in each pixel (τ_m). A histogram on the upper right panel displays the distribution of τ_m and color legend. The table next to it (rightmost) represents a biexponential fitting model used to fit the data and fitting results. On the lower right panel, there are experimental decay data (blue dots), biexponential fit of the data (red line), instrument response function (IRF) (green line) and fitting residuals (shown in black below the main data plot).

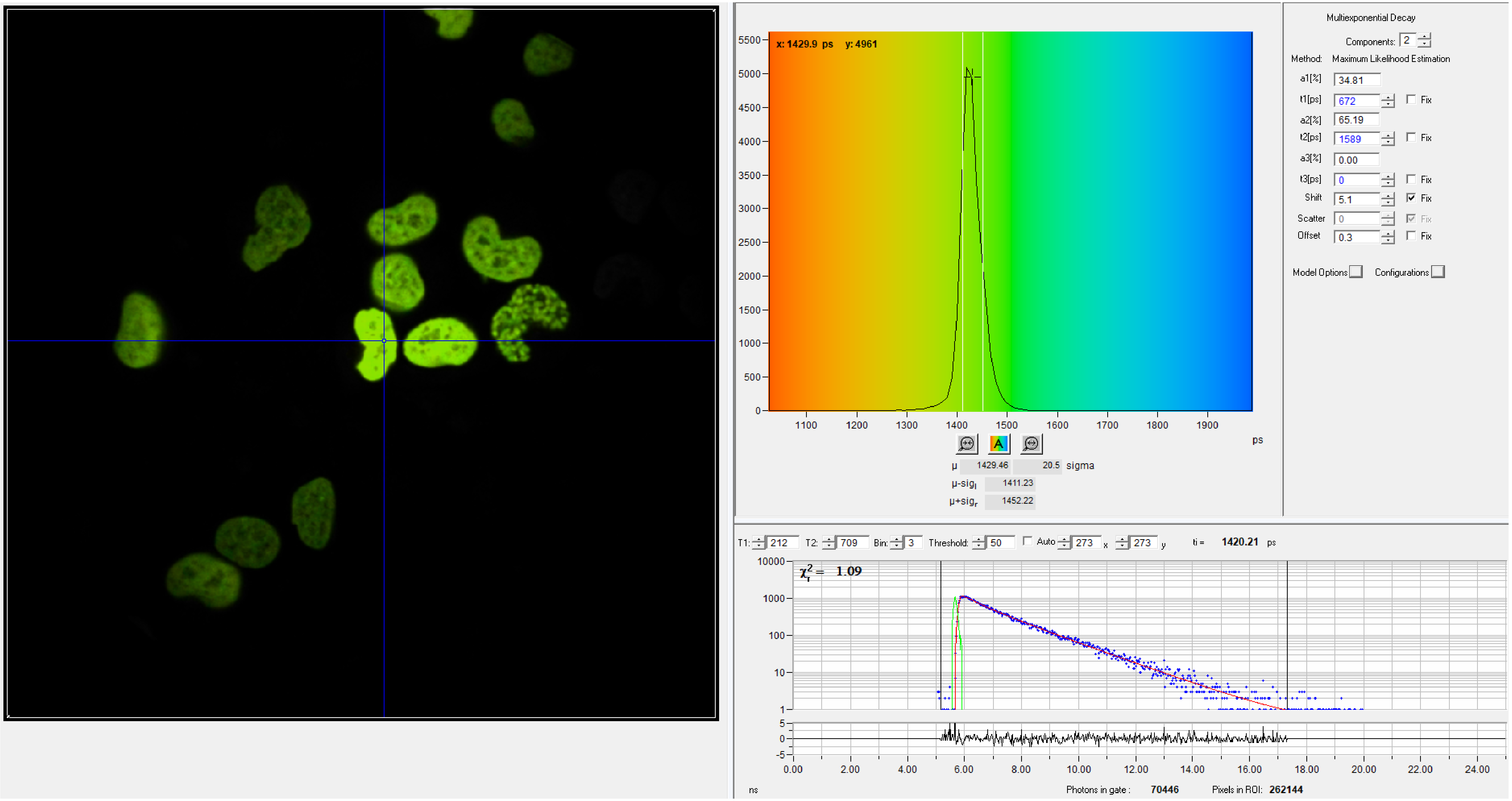

Figure S65. P68T FAST + **HMBR**; biexponential fit; τ_i color-coding. FLIM scan and corresponding time-resolved fluorescence data analysis of live HeLa cells expressing the P68T FAST variant fused to histone-2B (H2B) and stained with the **HMBR** fluorogen. A screenshot from Becker & Hickl SPCImage data acquisition and analysis window is shown. Biexponential fitting of decay data has been performed. On the left panel, there is a FLIM image of HeLa nuclei color-coded according to intensity-weighted average fluorescence lifetime in each pixel (τ_i). A histogram on the upper right panel displays the distribution of τ_i and color legend. The table next to it (rightmost) represents a biexponential fitting model used to fit the data and fitting results. On the lower right panel, there are experimental decay data (blue dots), biexponential fit of the data (red line), instrument response function (IRF) (green line) and fitting residuals (shown in black below the main data plot).

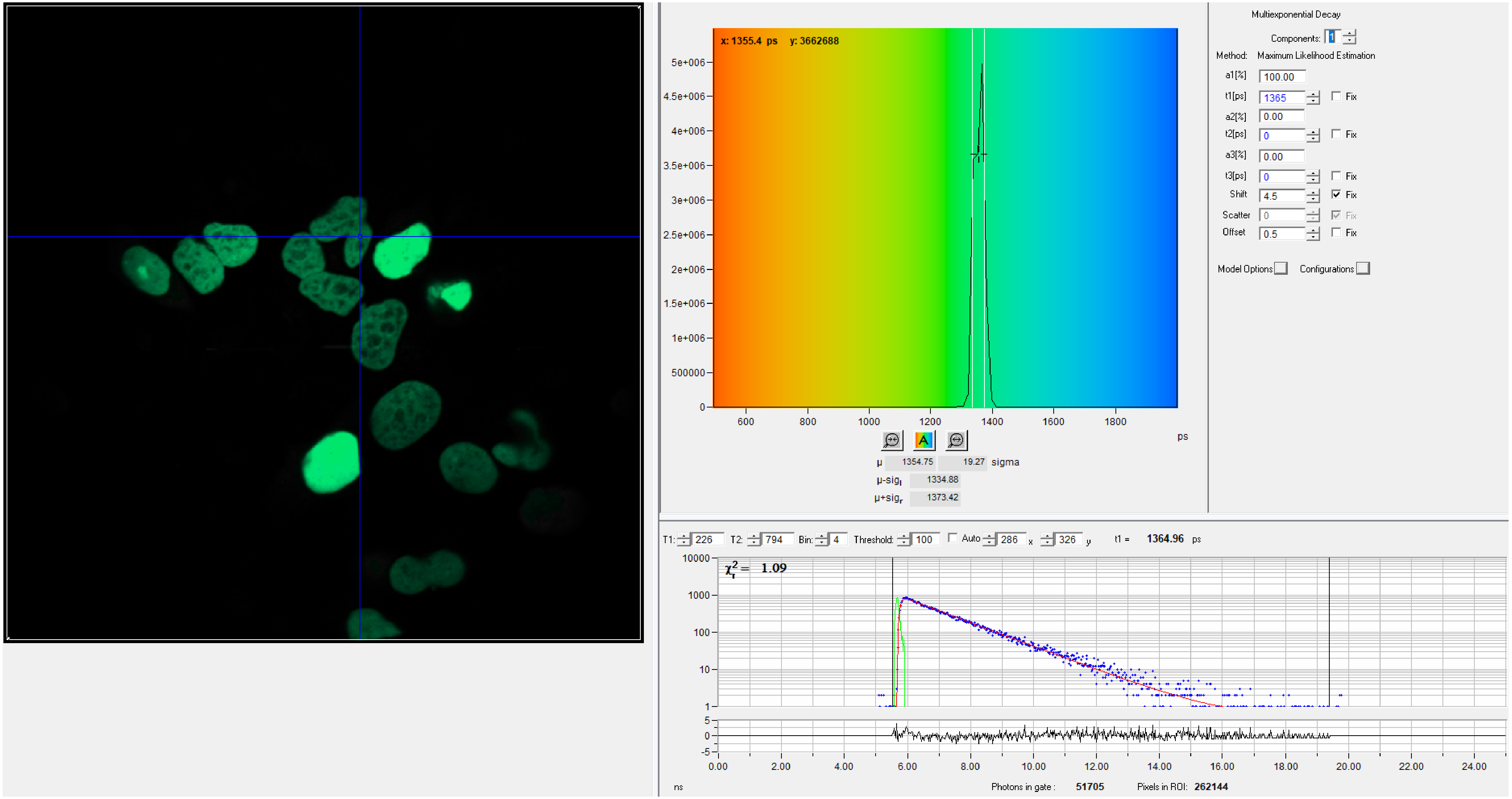

Figure S66. R52K FAST + **HBR-2,5-DM**; monoexponential fit; τ color-coding. FLIM scan and corresponding time-resolved fluorescence data analysis of live HeLa cells expressing the R52K FAST variant fused to histone-2B (H2B) and stained with the **HBR-2,5-DM** fluorogen. A screenshot from Becker & Hickl SPCImage data acquisition and analysis window is shown. Monoexponential fitting of decay data has been performed. On the left panel, there is a FLIM image of HeLa nuclei color-coded according to fluorescence lifetime in each pixel (τ). A histogram on the upper right panel displays the distribution of τ and color legend. The table next to it (rightmost) represents a monoexponential fitting model used to fit the data and fitting results. On the lower right panel, there are experimental decay data (blue dots), monoexponential fit of the data (red line), instrument response function (IRF) (green line) and fitting residuals (shown in black below the main data plot).

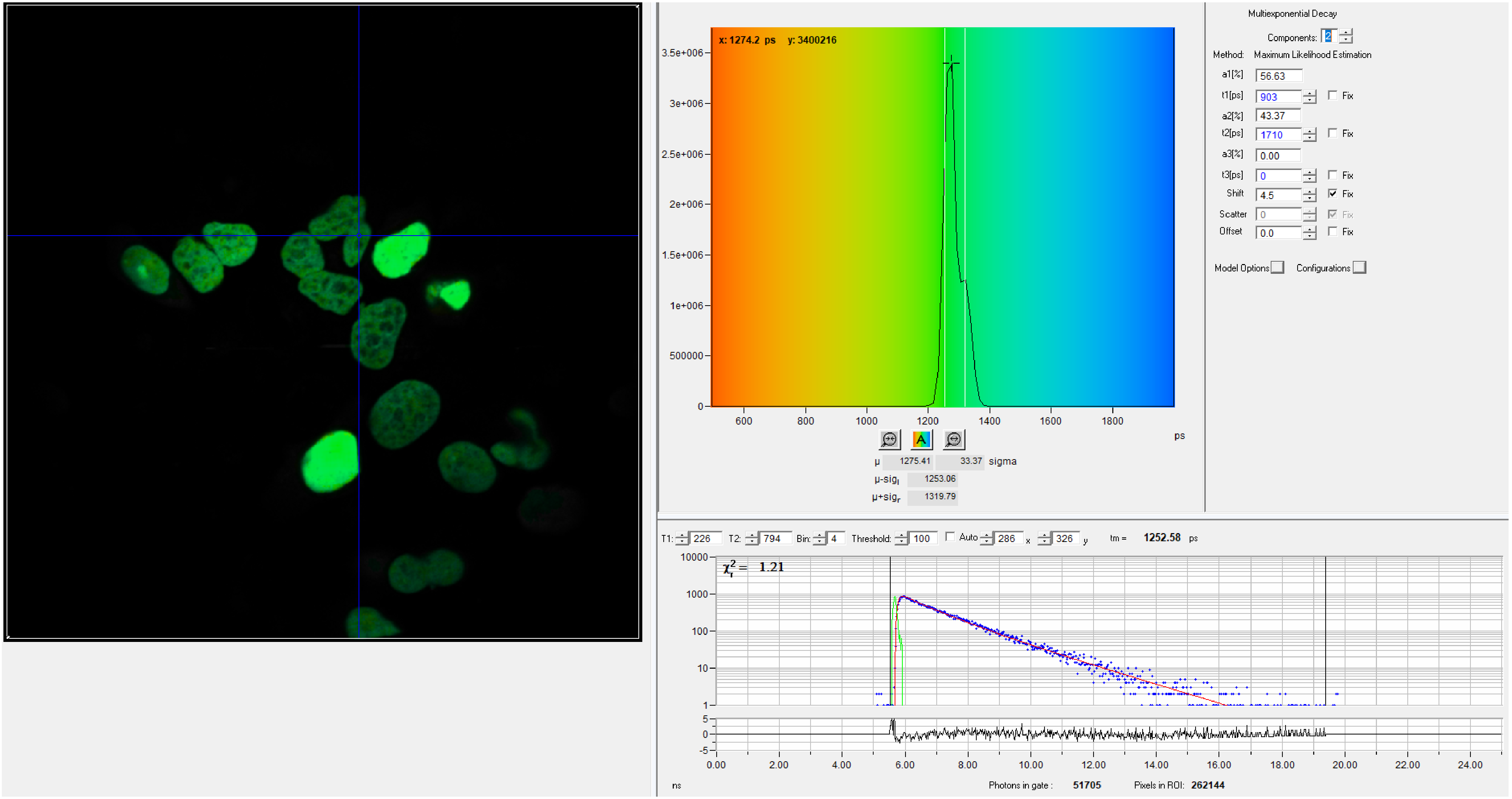

Figure S67. R52K FAST + **HBR-2,5-DM**; biexponential fit; τ_m color-coding. FLIM scan and corresponding time-resolved fluorescence data analysis of live HeLa cells expressing the R52K FAST variant fused to histone-2B (H2B) and stained with the **HBR-2,5-DM** fluorogen. A screenshot from Becker & Hickl SPCImage data acquisition and analysis window is shown. Biexponential fitting of decay data has been performed.On the left panel, there is a FLIM image of HeLa nuclei color-coded according to amplitude-weighted average fluorescence lifetime in each pixel (τ_m). A histogram on the upper right panel displays the distribution of τ_m and color legend. The table next to it (rightmost) represents a biexponential fitting model used to fit the data and fitting results. On the lower right panel, there are experimental decay data (blue dots), biexponential fit of the data (red line), instrument response function (IRF) (green line) and fitting residuals (shown in black below the main data plot).

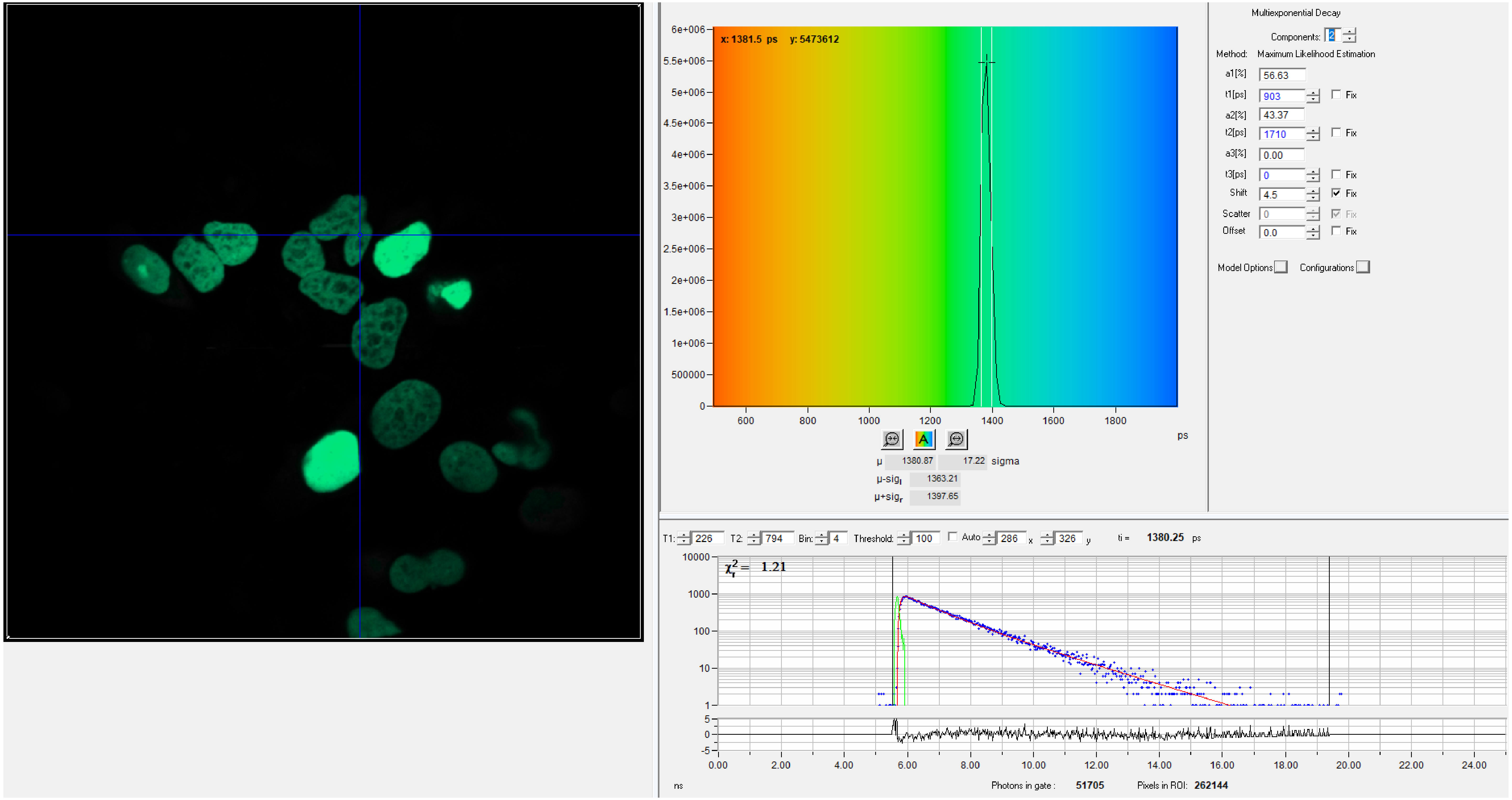

Figure S68. R52K FAST + **HBR-2,5-DM**; biexponential fit; τ_i color-coding. FLIM scan and corresponding time-resolved fluorescence data analysis of live HeLa cells expressing the R52K FAST variant fused to histone-2B (H2B) and stained with the **HBR-2,5-DM** fluorogen. A screenshot from Becker & Hickl SPCImage data acquisition and analysis window is shown. Biexponential fitting of decay data has been performed. On the left panel, there is a FLIM image of HeLa nuclei color-coded according to intensity-weighted average fluorescence lifetime in each pixel (τ_i). A histogram on the upper right panel displays the distribution of τ_i and color legend. The table next to it (rightmost) represents a biexponential fitting model used to fit the data and fitting results. On the lower right panel, there are experimental decay data (blue dots), biexponential fit of the data (red line), instrument response function (IRF) (green line) and fitting residuals (shown in black below the main data plot).

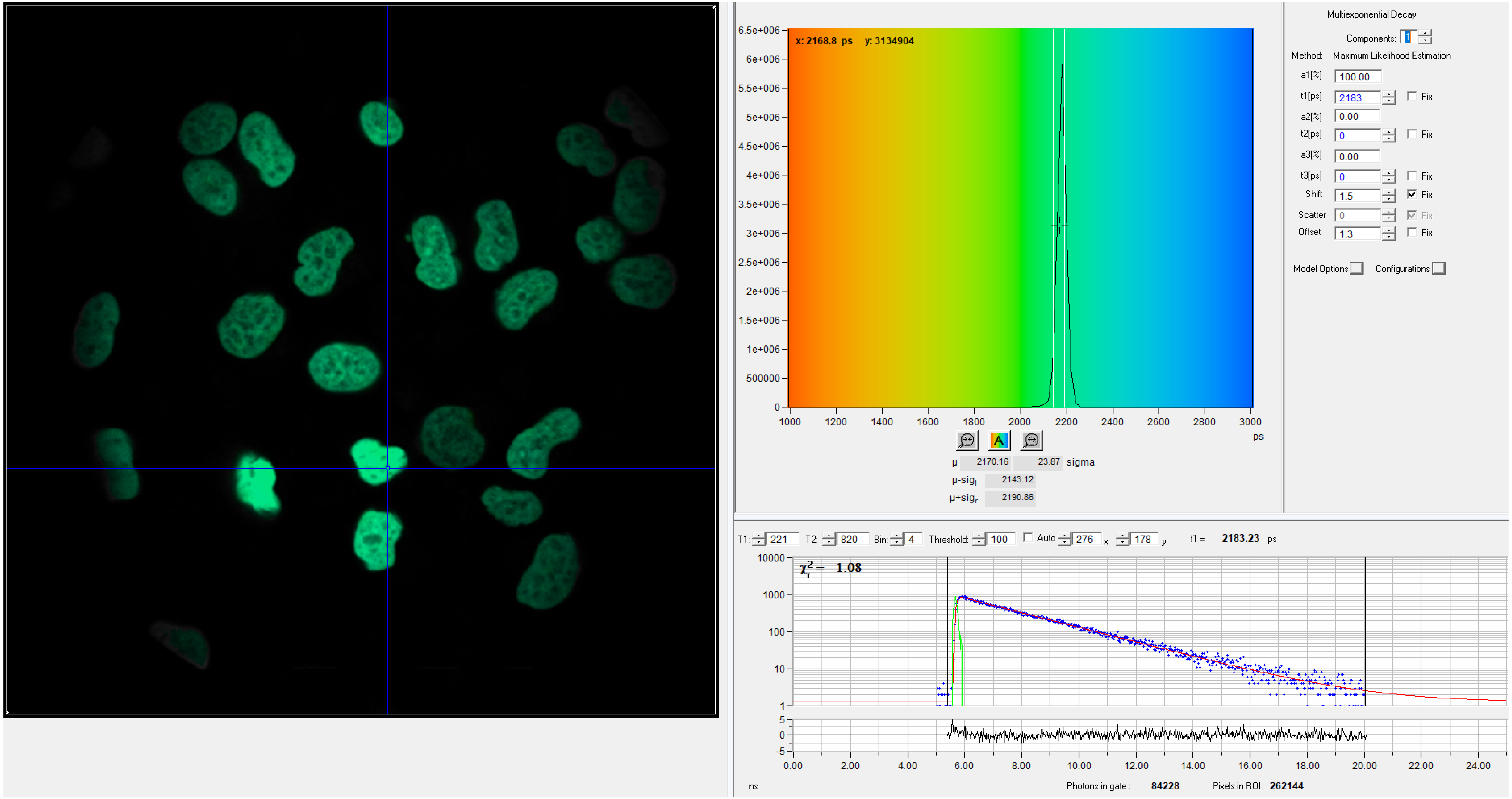

Figure S69. F62L FAST + **HBR-2,5-DM**; monoexponential fit; τ color-coding. FLIM scan and corresponding time-resolved fluorescence data analysis of live HeLa cells expressing the F62L FAST variant fused to histone-2B (H2B) and stained with the **HBR-2,5-DM** fluorogen. A screenshot from Becker & Hickl SPCImage data acquisition and analysis window is shown. Monoexponential fitting of decay data has been performed. On the left panel, there is a FLIM image of HeLa nuclei color-coded according to fluorescence lifetime in each pixel (τ). A histogram on the upper right panel displays the distribution of τ and color legend. The table next to it (rightmost) represents a monoexponential fitting model used to fit the data and fitting results. On the lower right panel, there are experimental decay data (blue dots), monoexponential fit of the data (red line), instrument response function (IRF) (green line) and fitting residuals (shown in black below the main data plot).

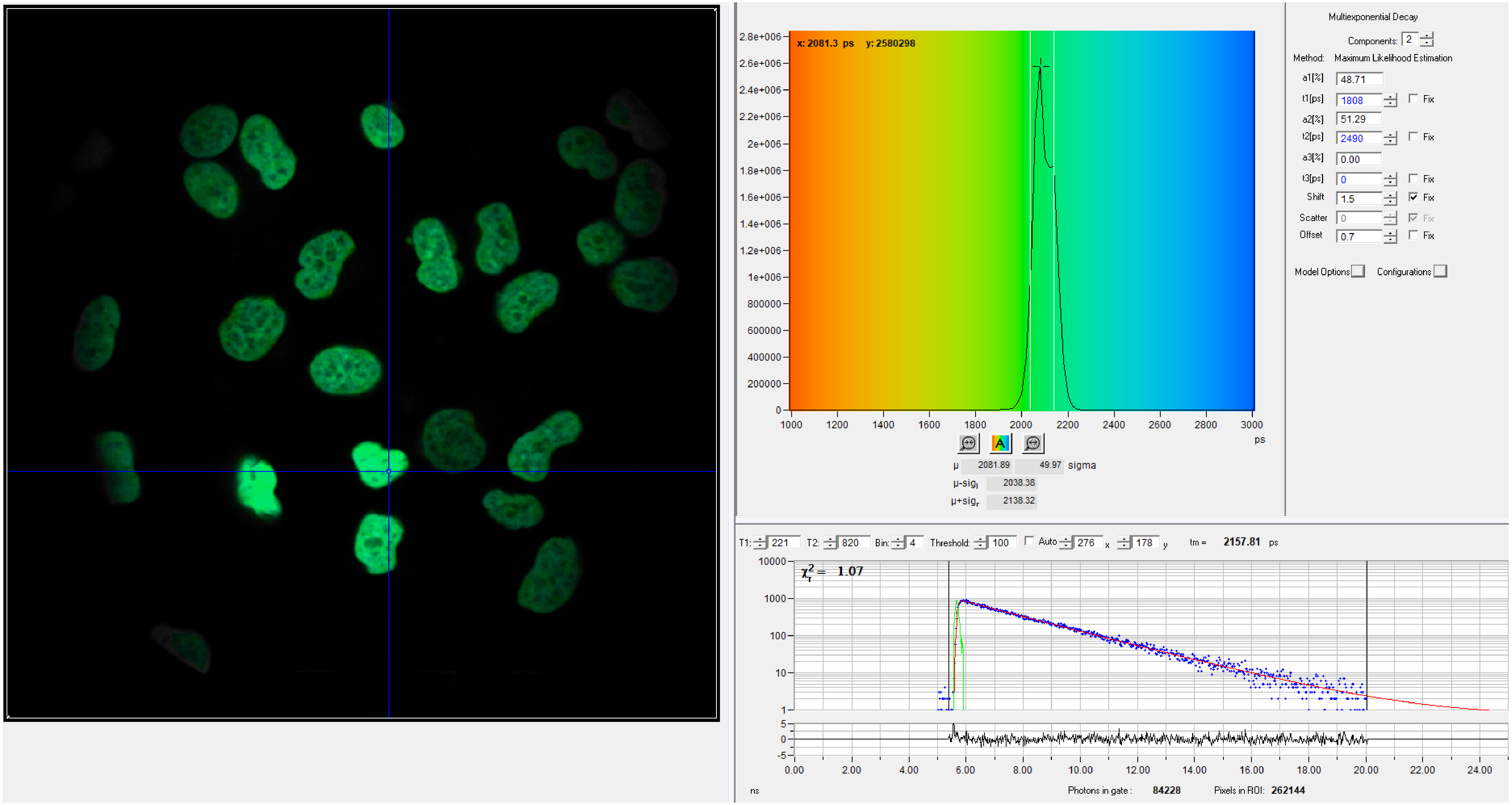

Figure S70. F62L FAST + **HBR-2,5-DM**; biexponential fit; τ_m color-coding. FLIM scan and corresponding time-resolved fluorescence data analysis of live HeLa cells expressing the F62L FAST variant fused to histone-2B (H2B) and stained with the **HBR-2,5-DM** fluorogen. A screenshot from Becker & Hickl SPCImage data acquisition and analysis window is shown. Biexponential fitting of decay data has been performed.On the left panel, there is a FLIM image of HeLa nuclei color-coded according to amplitude-weighted average fluorescence lifetime in each pixel (τ_m). A histogram on the upper right panel displays the distribution of τ_m and color legend. The table next to it (rightmost) represents a biexponential fitting model used to fit the data and fitting results. On the lower right panel, there are experimental decay data (blue dots), biexponential fit of the data (red line), instrument response function (IRF) (green line) and fitting residuals (shown in black below the main data plot).

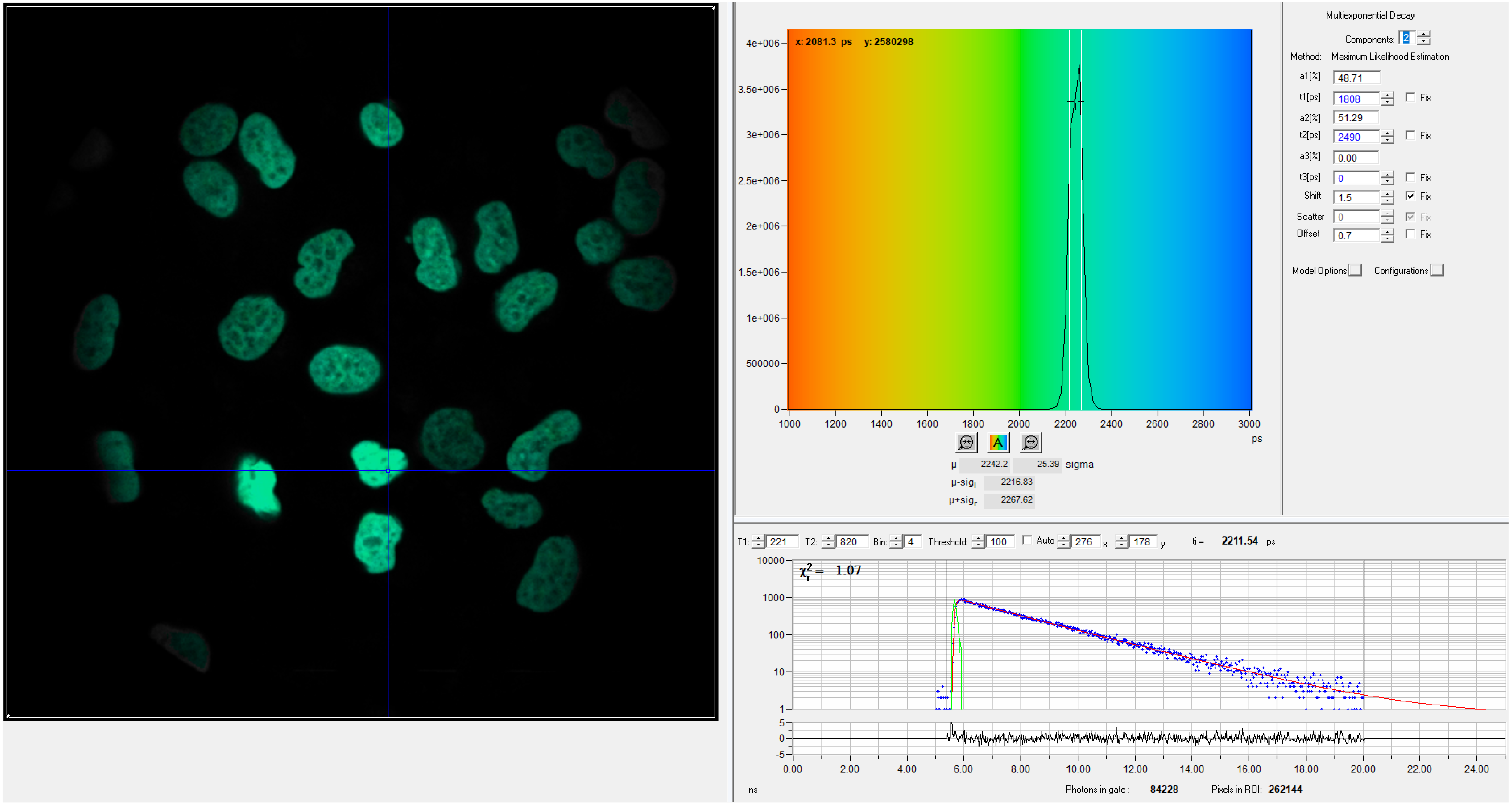

Figure S71. F62L FAST + **HBR-2,5-DM**; biexponential fit; τ_i color-coding. FLIM scan and corresponding time-resolved fluorescence data analysis of live HeLa cells expressing the F62L FAST variant fused to histone-2B (H2B) and stained with the **HBR-2,5-DM** fluorogen. A screenshot from Becker & Hickl SPCImage data acquisition and analysis window is shown. Biexponential fitting of decay data has been performed. On the left panel, there is a FLIM image of HeLa nuclei color-coded according to intensity-weighted average fluorescence lifetime in each pixel (τ_i). A histogram on the upper right panel displays the distribution of τ_i and color legend. The table next to it (rightmost) represents a biexponential fitting model used to fit the data and fitting results. On the lower right panel, there are experimental decay data (blue dots), biexponential fit of the data (red line), instrument response function (IRF) (green line) and fitting residuals (shown in black below the main data plot).

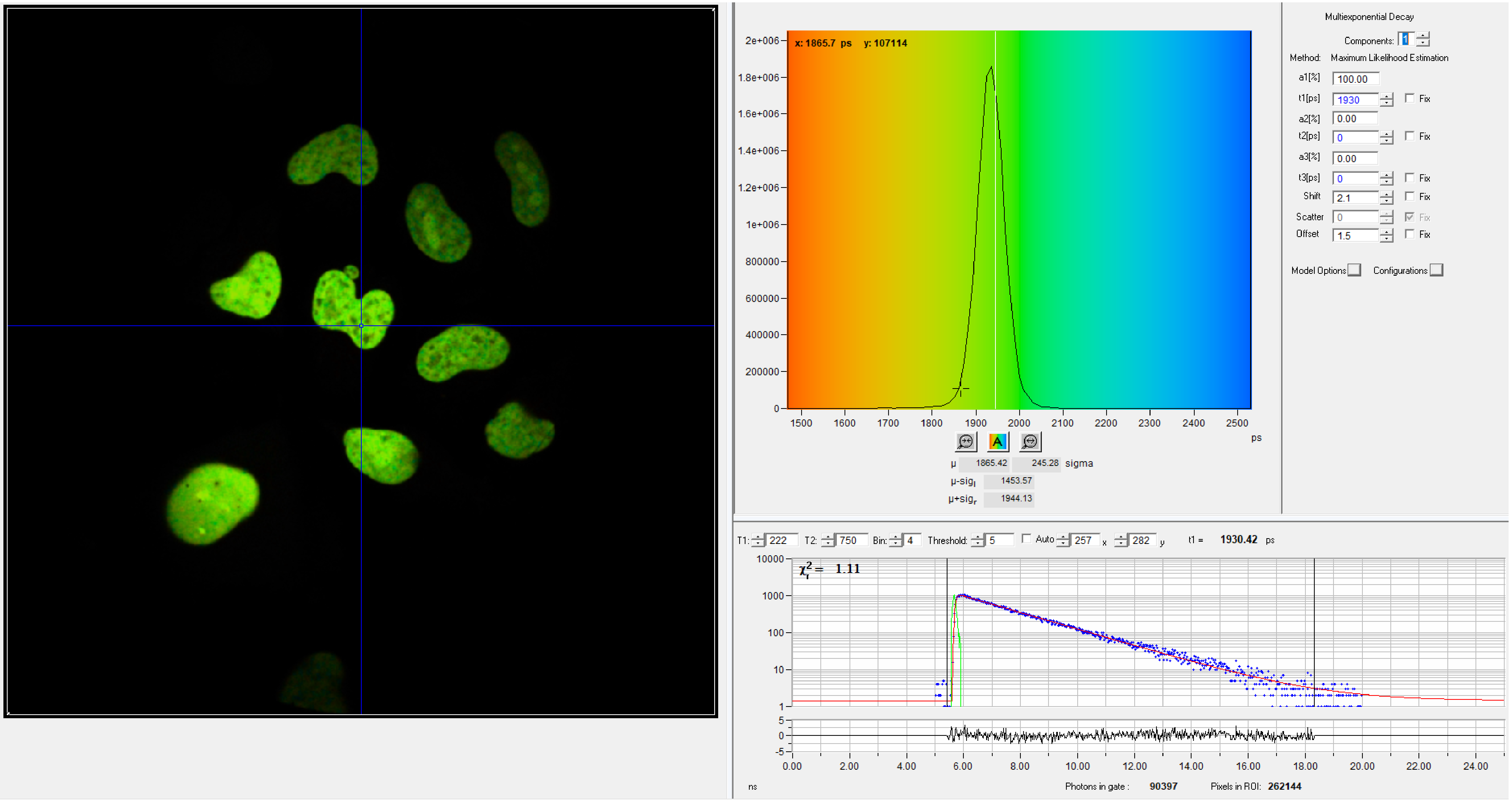

Figure S72. P68K FAST + **HBR-2,5-DM**; monoexponential fit; τ color-coding. FLIM scan and corresponding time-resolved fluorescence data analysis of live HeLa cells expressing the P68K FAST variant fused to histone-2B (H2B) and stained with the **HBR-2,5-DM** fluorogen. A screenshot from Becker & Hickl SPCImage data acquisition and analysis window is shown. Monoexponential fitting of decay data has been performed. On the left panel, there is a FLIM image of HeLa nuclei color-coded according to fluorescence lifetime in each pixel (τ). A histogram on the upper right panel displays the distribution of τ and color legend. The table next to it (rightmost) represents a monoexponential fitting model used to fit the data and fitting results. On the lower right panel, there are experimental decay data (blue dots), monoexponential fit of the data (red line), instrument response function (IRF) (green line) and fitting residuals (shown in black below the main data plot).

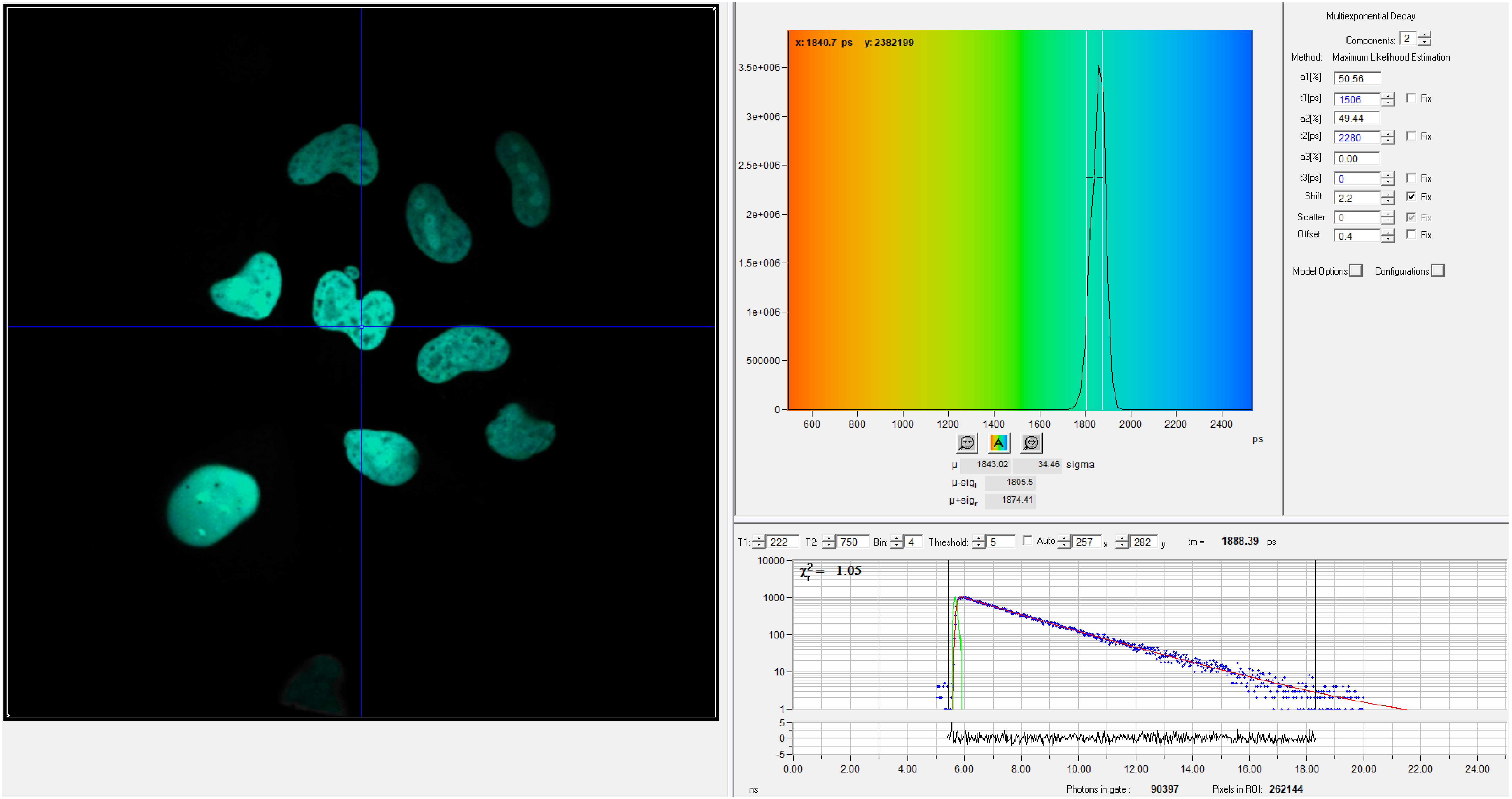

Figure S73. P68K FAST + **HBR-2,5-DM**; biexponential fit; τ_m color-coding. FLIM scan and corresponding time-resolved fluorescence data analysis of live HeLa cells expressing the P68K FAST variant fused to histone-2B (H2B) and stained with the **HBR-2,5-DM** fluorogen. A screenshot from Becker & Hickl SPCImage data acquisition and analysis window is shown. Biexponential fitting of decay data has been performed.On the left panel, there is a FLIM image of HeLa nuclei color-coded according to amplitude-weighted average fluorescence lifetime in each pixel (τ_m). A histogram on the upper right panel displays the distribution of τ_m and color legend. The table next to it (rightmost) represents a biexponential fitting model used to fit the data and fitting results. On the lower right panel, there are experimental decay data (blue dots), biexponential fit of the data (red line), instrument response function (IRF) (green line) and fitting residuals (shown in black below the main data plot).

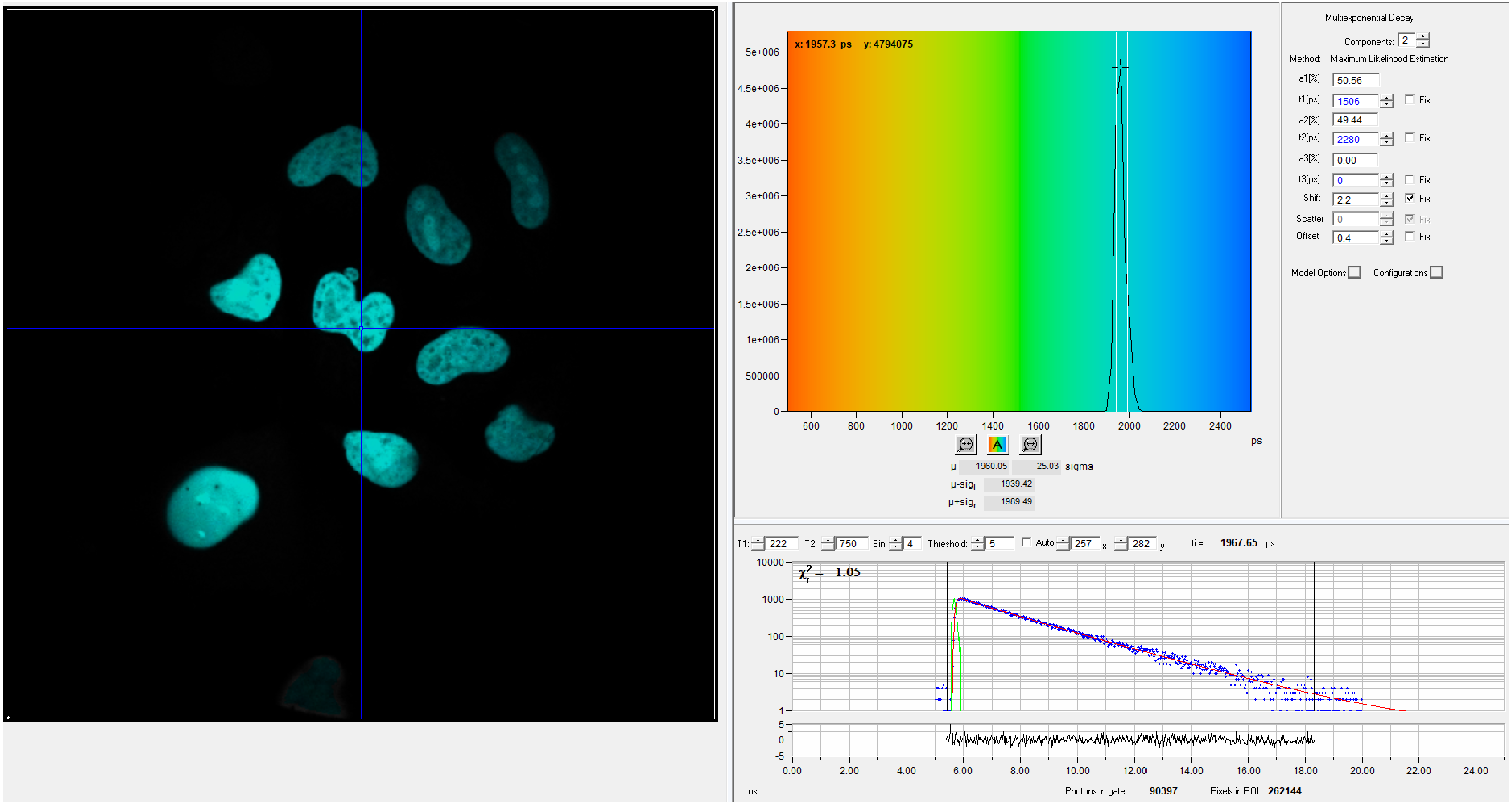

Figure S74. P68K FAST + **HBR-2,5-DM**; biexponential fit; τ_i color-coding. FLIM scan and corresponding time-resolved fluorescence data analysis of live HeLa cells expressing the P68K FAST variant fused to histone-2B (H2B) and stained with the **HBR-2,5-DM** fluorogen. A screenshot from Becker & Hickl SPCImage data acquisition and analysis window is shown. Biexponential fitting of decay data has been performed. On the left panel, there is a FLIM image of HeLa nuclei color-coded according to intensity-weighted average fluorescence lifetime in each pixel (τ_i). A histogram on the upper right panel displays the distribution of τ_i and color legend. The table next to it (rightmost) represents a biexponential fitting model used to fit the data and fitting results. On the lower right panel, there are experimental decay data (blue dots), biexponential fit of the data (red line), instrument response function (IRF) (green line) and fitting residuals (shown in black below the main data plot).

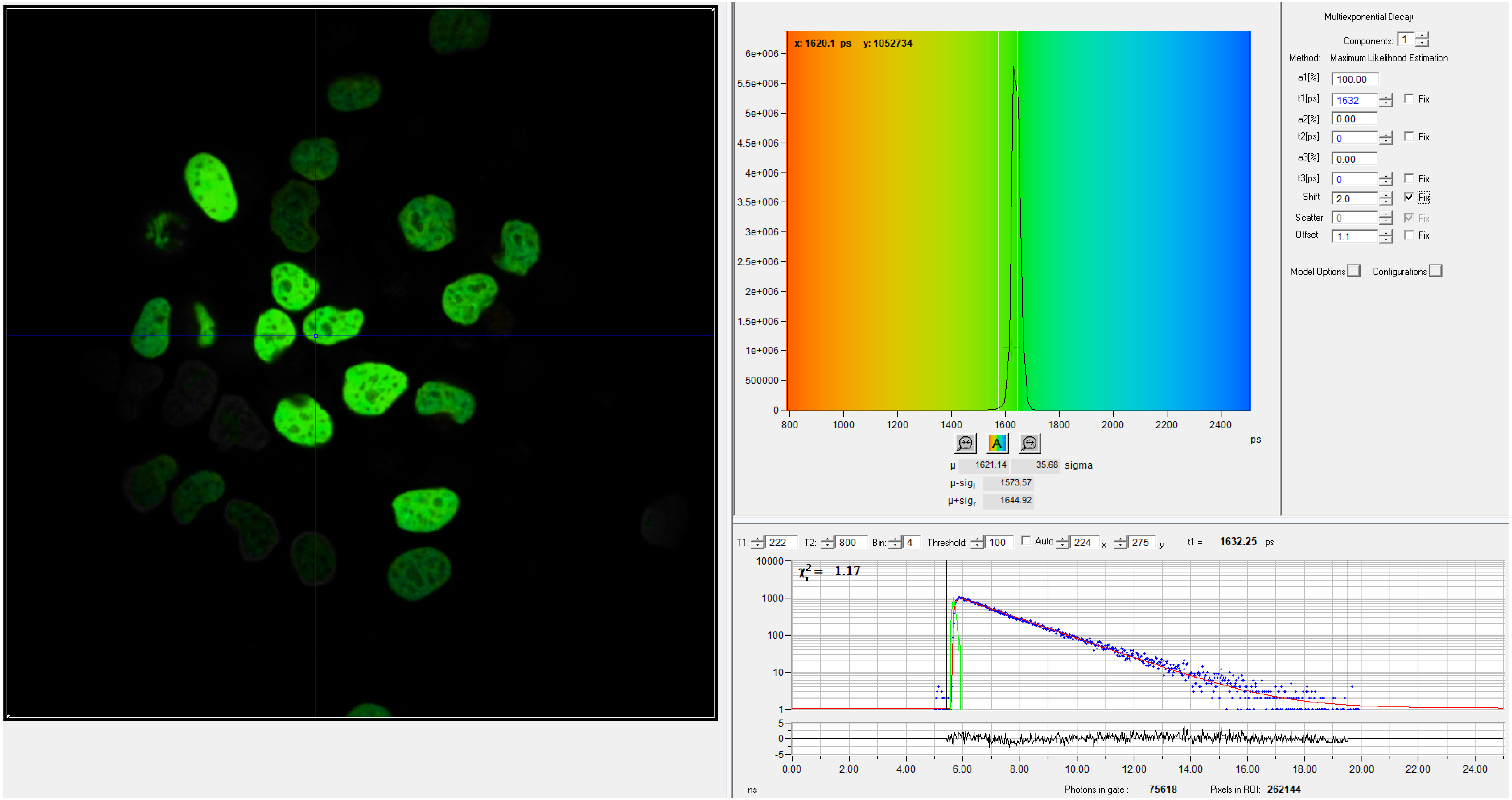

Figure S75. P68T FAST + **HBR-2,5-DM**; monoexponential fit; τ color-coding. FLIM scan and corresponding time-resolved fluorescence data analysis of live HeLa cells expressing the P68T FAST variant fused to histone-2B (H2B) and stained with the **HBR-2,5-DM** fluorogen. A screenshot from Becker & Hickl SPCImage data acquisition and analysis window is shown. Monoexponential fitting of decay data has been performed. On the left panel, there is a FLIM image of HeLa nuclei color-coded according to fluorescence lifetime in each pixel (τ). A histogram on the upper right panel displays the distribution of τ and color legend. The table next to it (rightmost) represents a monoexponential fitting model used to fit the data and fitting results. On the lower right panel, there are experimental decay data (blue dots), monoexponential fit of the data (red line), instrument response function (IRF) (green line) and fitting residuals (shown in black below the main data plot).

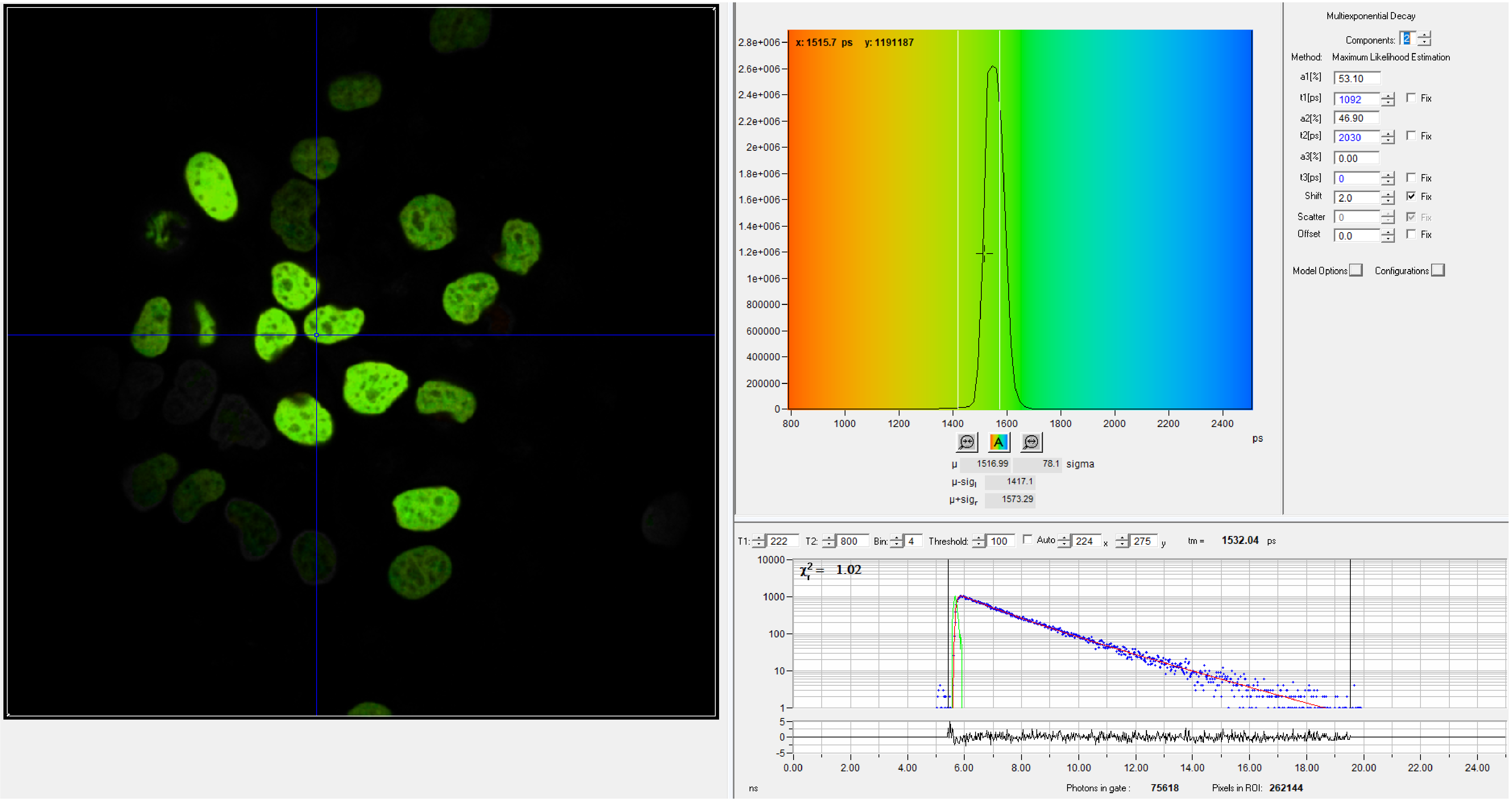

Figure S76. P68T FAST + **HBR-2,5-DM**; biexponential fit; τ_m color-coding. FLIM scan and corresponding time-resolved fluorescence data analysis of live HeLa cells expressing the P68T FAST variant fused to histone-2B (H2B) and stained with the **HBR-2,5-DM** fluorogen. A screenshot from Becker & Hickl SPCImage data acquisition and analysis window is shown. Biexponential fitting of decay data has been performed.On the left panel, there is a FLIM image of HeLa nuclei color-coded according to amplitude-weighted average fluorescence lifetime in each pixel (τ_m). A histogram on the upper right panel displays the distribution of τ_m and color legend. The table next to it (rightmost) represents a biexponential fitting model used to fit the data and fitting results. On the lower right panel, there are experimental decay data (blue dots), biexponential fit of the data (red line), instrument response function (IRF) (green line) and fitting residuals (shown in black below the main data plot).

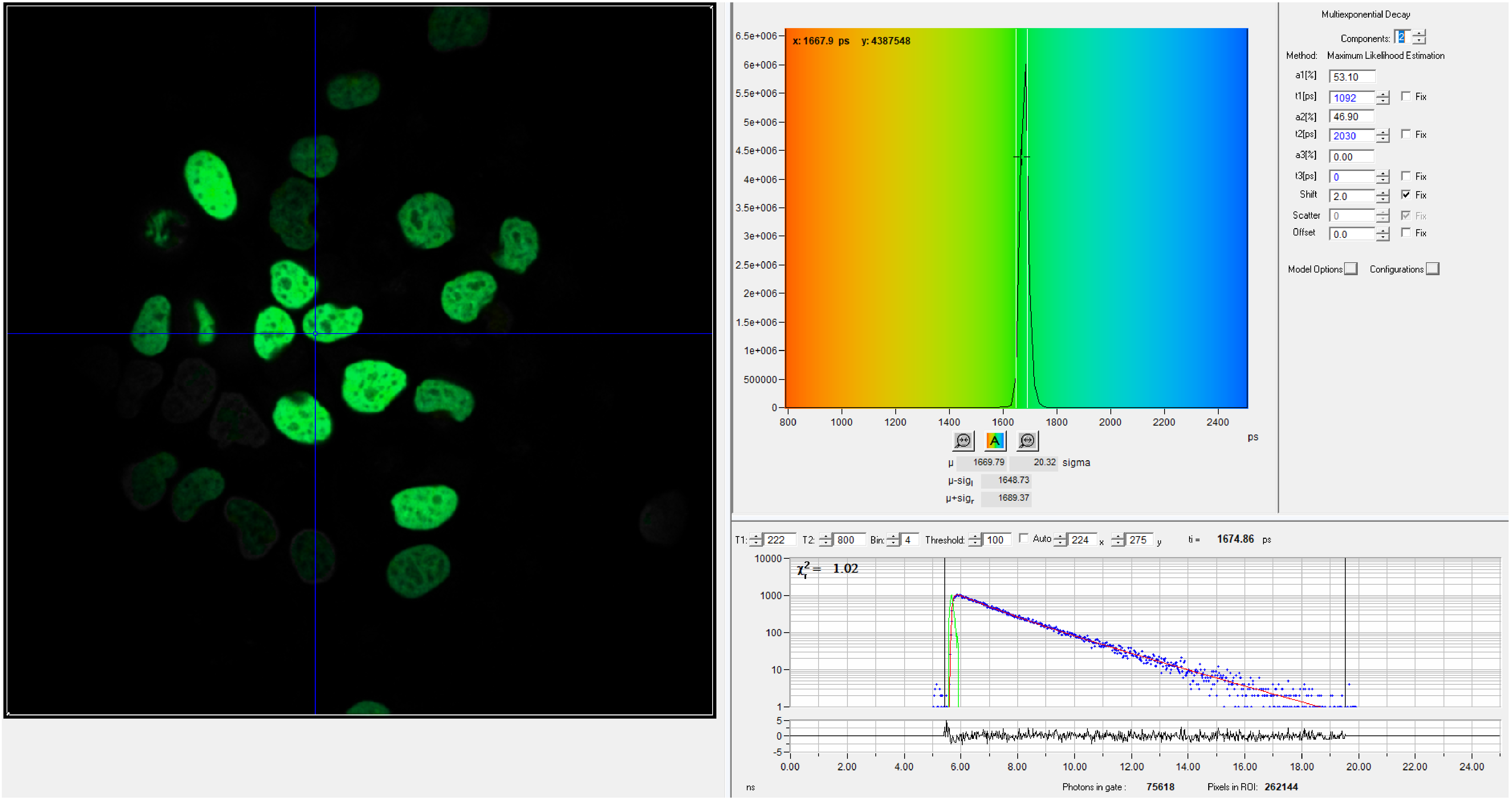

Figure S77. P68T FAST + **HBR-2,5-DM**; biexponential fit; τ_i color-coding. FLIM scan and corresponding time-resolved fluorescence data analysis of live HeLa cells expressing the P68T FAST variant fused to histone-2B (H2B) and stained with the **HBR-2,5-DM** fluorogen. A screenshot from Becker & Hickl SPCImage data acquisition and analysis window is shown. Biexponential fitting of decay data has been performed. On the left panel, there is a FLIM image of HeLa nuclei color-coded according to intensity-weighted average fluorescence lifetime in each pixel (τ_i). A histogram on the upper right panel displays the distribution of τ_i and color legend. The table next to it (rightmost) represents a biexponential fitting model used to fit the data and fitting results. On the lower right panel, there are experimental decay data (blue dots), biexponential fit of the data (red line), instrument response function (IRF) (green line) and fitting residuals (shown in black below the main data plot).

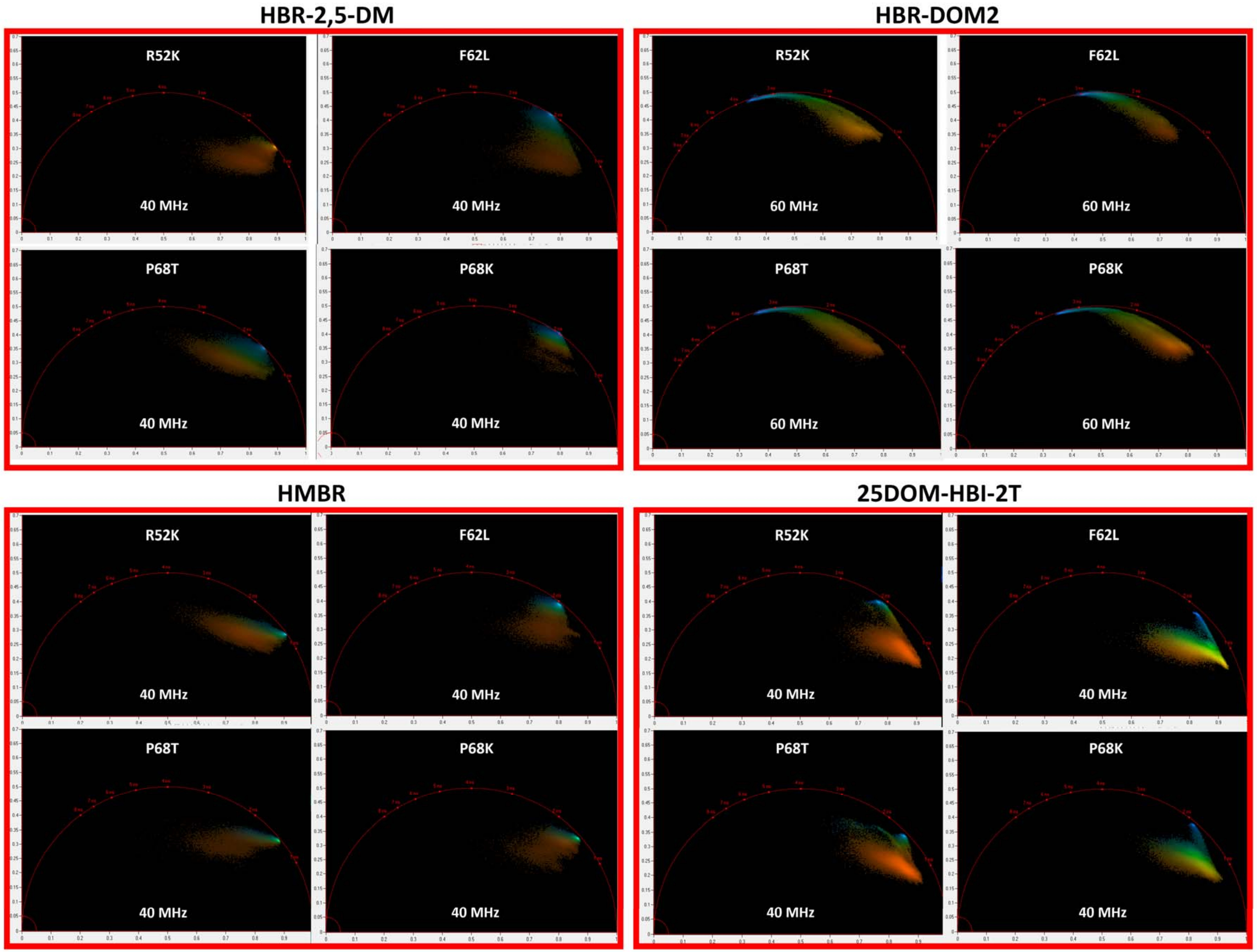

Figure S78. Phasor plots of the all fluorogen-FAST variant pairs tested in the study.

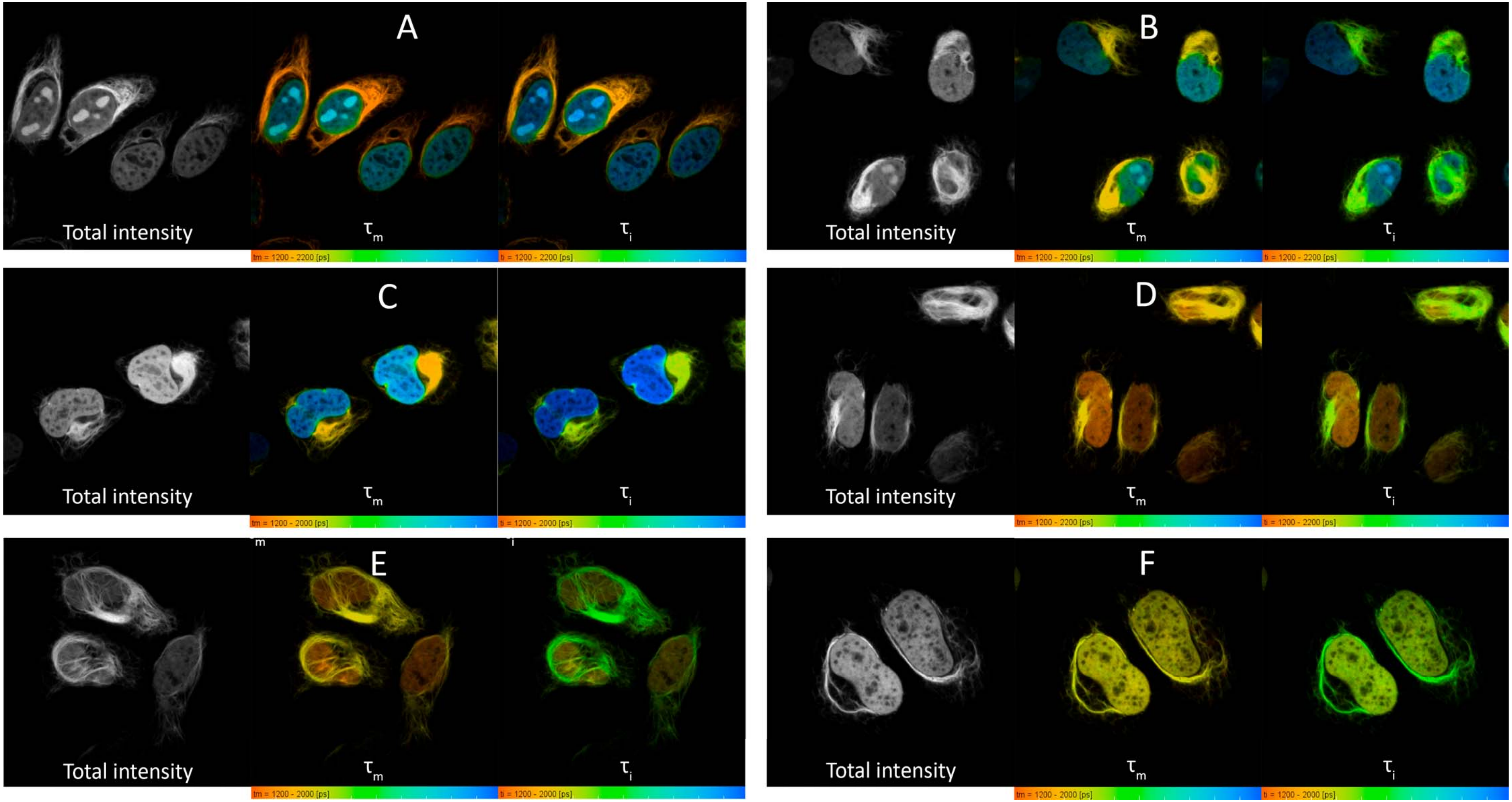

Figure S79. Color-coded FLIM images of **HMBR** in complexes with two FAST variants expressed simultaneously in live Hela cells as H2B and vimentin fusions. Total intensity, amplitude-weighted average lifetime (τ_m) and intensity weighted average lifetime (τ_i) in each pixel is presented. Color-coding was applied to full pixel intensity. Color-code range is specified below each image. A - H2B-F62L and vimentin-R52K (representative image, similar result in *n*=21 cells), B - H2B-F62L and vimentin-P68K (representative image, similar result in *n*=21 cells), C - H2B-F62L and vimentin-P68T (representative image, similar result in *n*=18 cells), D – H2B-R52K and vimentin-P68K (representative image, similar result in *n*=23 cells), E – H2B- R52K and vimentin-P68T (representative image, similar result in *n*=24 cells), F - H2B-P68T and vimentin-P68K (representative image, similar result in *n*=15 cells).

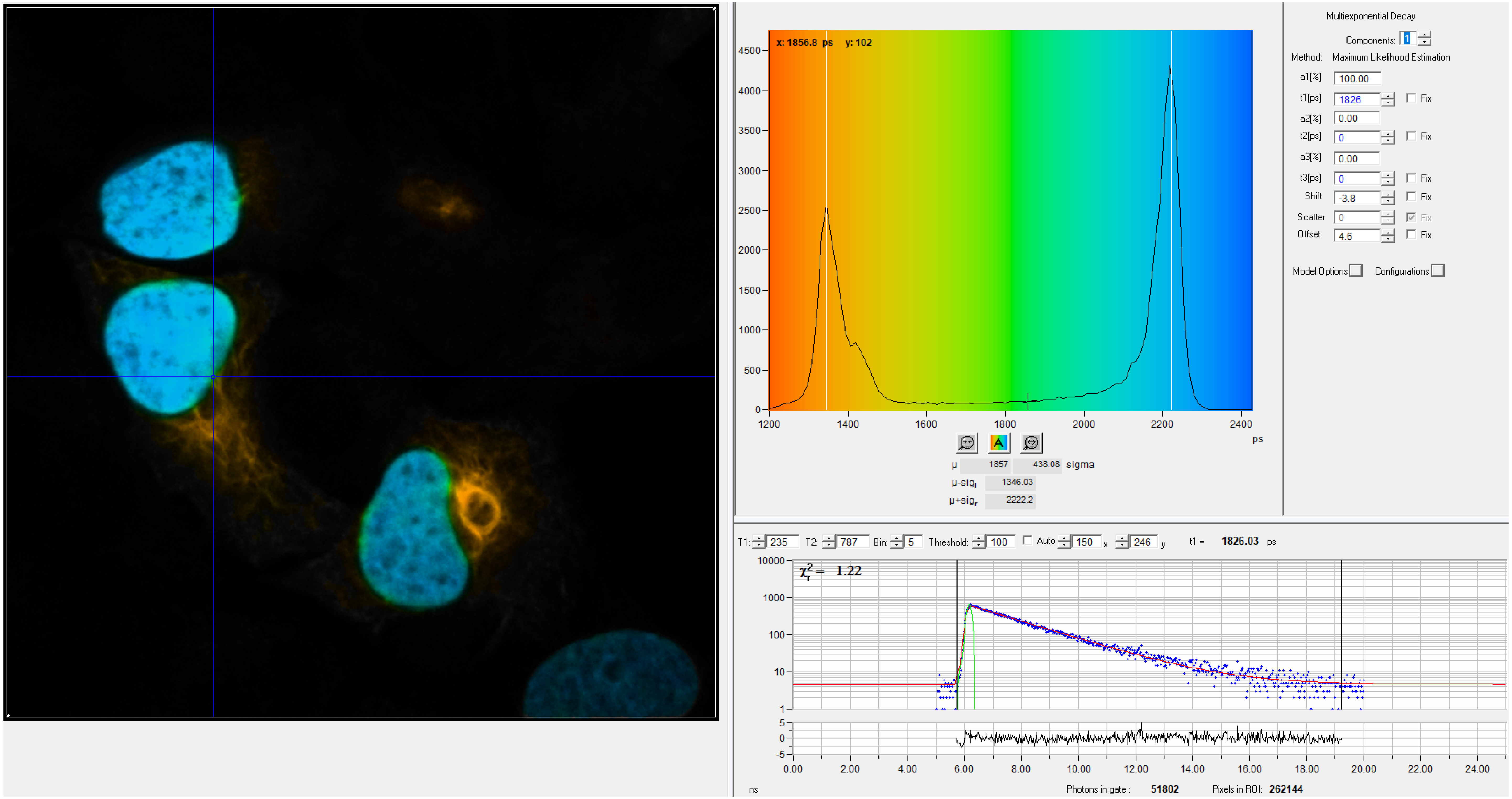

Figure S80. H2B-F62L and R52K-vimentin FAST + **HBR-2,5-DM**; monoexponential fit; τ color-coding. FLIM scan and corresponding time-resolved fluorescence data analysis of live HeLa cells expressing the H2B-F62L and R52K-vimentin FAST variants simultaneously and stained with the **HBR-2,5-DM** fluorogen. A screenshot from Becker & Hickl SPCImage data acquisition and analysis window is shown. Monoexponential fitting of decay data has been performed. On the left panel, there is a FLIM image of HeLa nuclei color-coded according to fluorescence lifetime in each pixel (τ). A histogram on the upper right panel displays the distribution of τ and color legend. The table next to it (rightmost) represents a monoexponential fitting model used to fit the data and fitting results. On the lower right panel, there are experimental decay data (blue dots), monoexponential fit of the data (red line), instrument response function (IRF) (green line) and fitting residuals (shown in black below the main data plot).

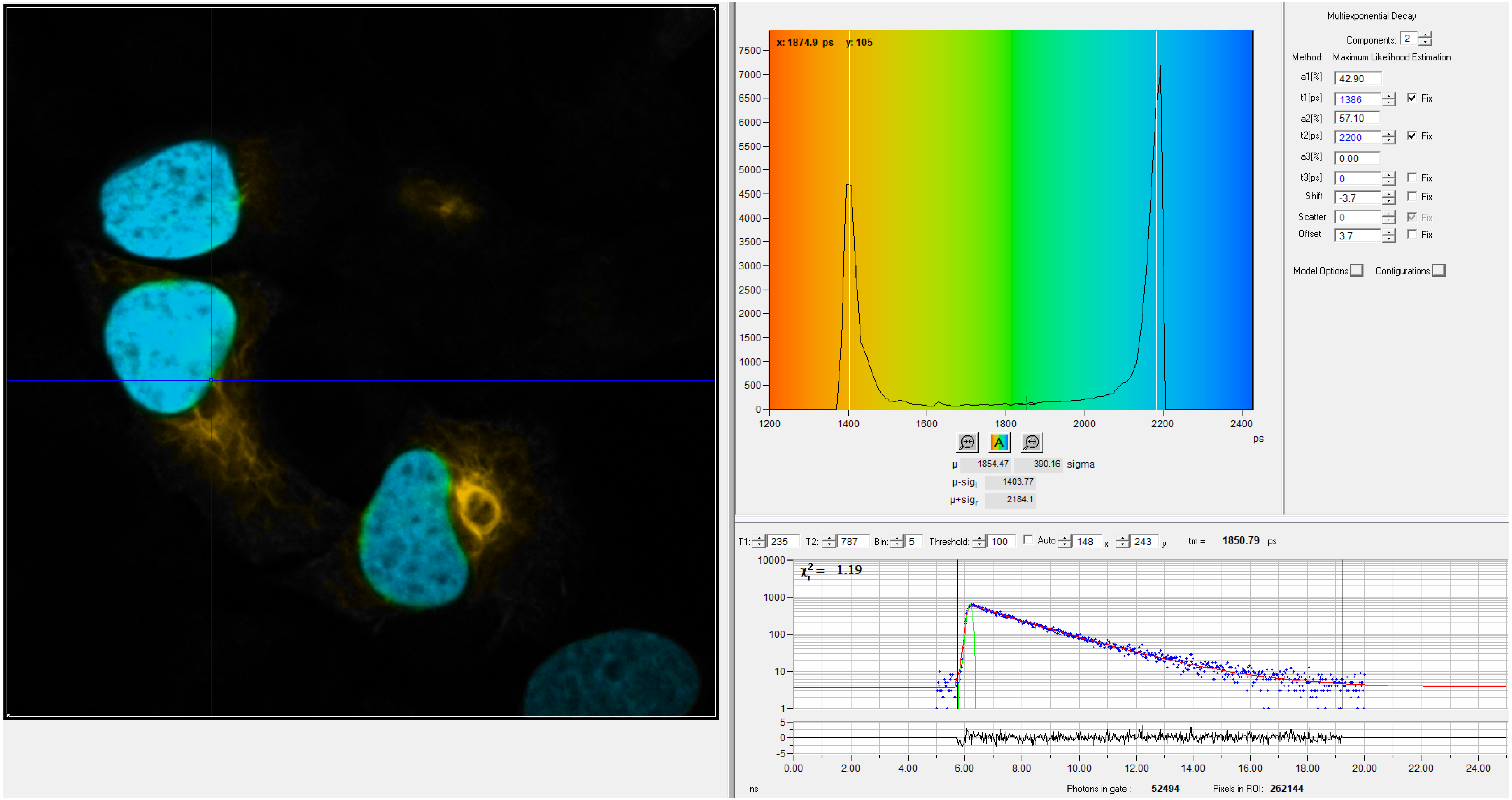

Figure S81. H2B-F62L and R52K-vimentin FAST + **HBR-2,5-DM**; biexponential fit; τ_m color-coding. FLIM scan and corresponding time-resolved fluorescence data analysis of live HeLa cells expressing the H2B-F62L and R52K-vimentin FAST variants simultaneously and stained with the **HBR-2,5-DM** fluorogen. A screenshot from Becker & Hickl SPCImage data acquisition and analysis window is shown. Biexponential fitting of decay data with both exponent components fixation at known values has been performed. On the left panel, there is a FLIM image of HeLa nuclei color-coded according to amplitude-weighted average fluorescence lifetime in each pixel (τ_m). A histogram on the upper right panel displays the distribution of τ_m and color legend. The table next to it (rightmost) represents a biexponential fitting model used to fit the data and fitting results. On the lower right panel, there are experimental decay data (blue dots), biexponential fit of the data (red line), instrument response function (IRF) (green line) and fitting residuals (shown in black below the main data plot).

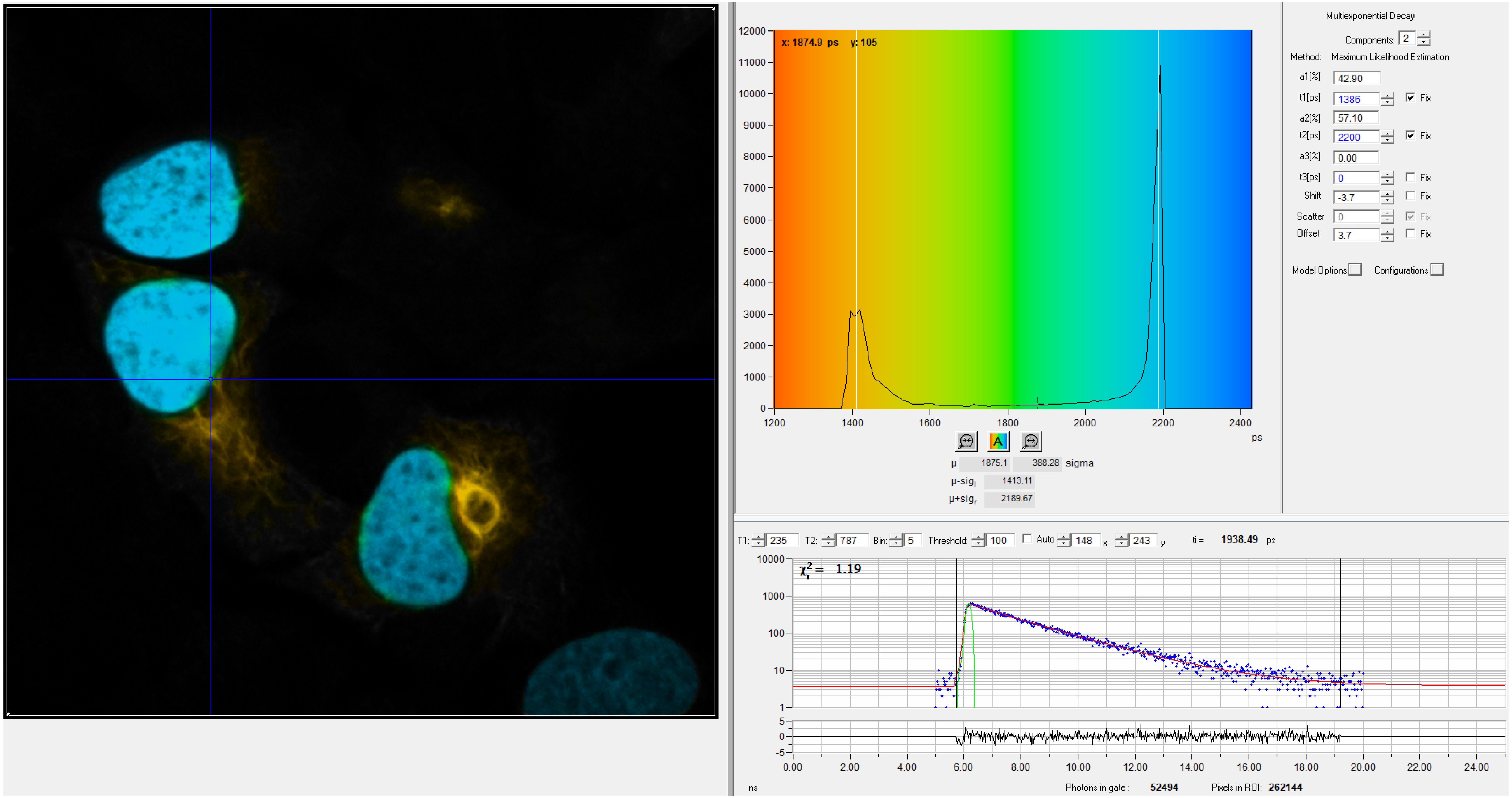

Figure S82. H2B-F62L and R52K-vimentin FAST + **HBR-2,5-DM**; biexponential fit; τ_i color-coding. FLIM scan and corresponding time-resolved fluorescence data analysis of live HeLa cells expressing the H2B-F62L and R52K-vimentin FAST variants simultaneously and stained with the **HBR-2,5-DM** fluorogen. A screenshot from Becker & Hickl SPCImage data acquisition and analysis window is shown. Biexponential fitting of decay data with both exponent components fixation at known values has been performed. On the left panel, there is a FLIM image of HeLa nuclei color-coded according to intensity-weighted average fluorescence lifetime in each pixel (τ_i). A histogram on the upper right panel displays the distribution of τ_i and color legend. The table next to it (rightmost) represents a biexponential fitting model used to fit the data and fitting results. On the lower right panel, there are experimental decay data (blue dots), biexponential fit of the data (red line), instrument response function (IRF) (green line) and fitting residuals (shown in black below the main data plot).

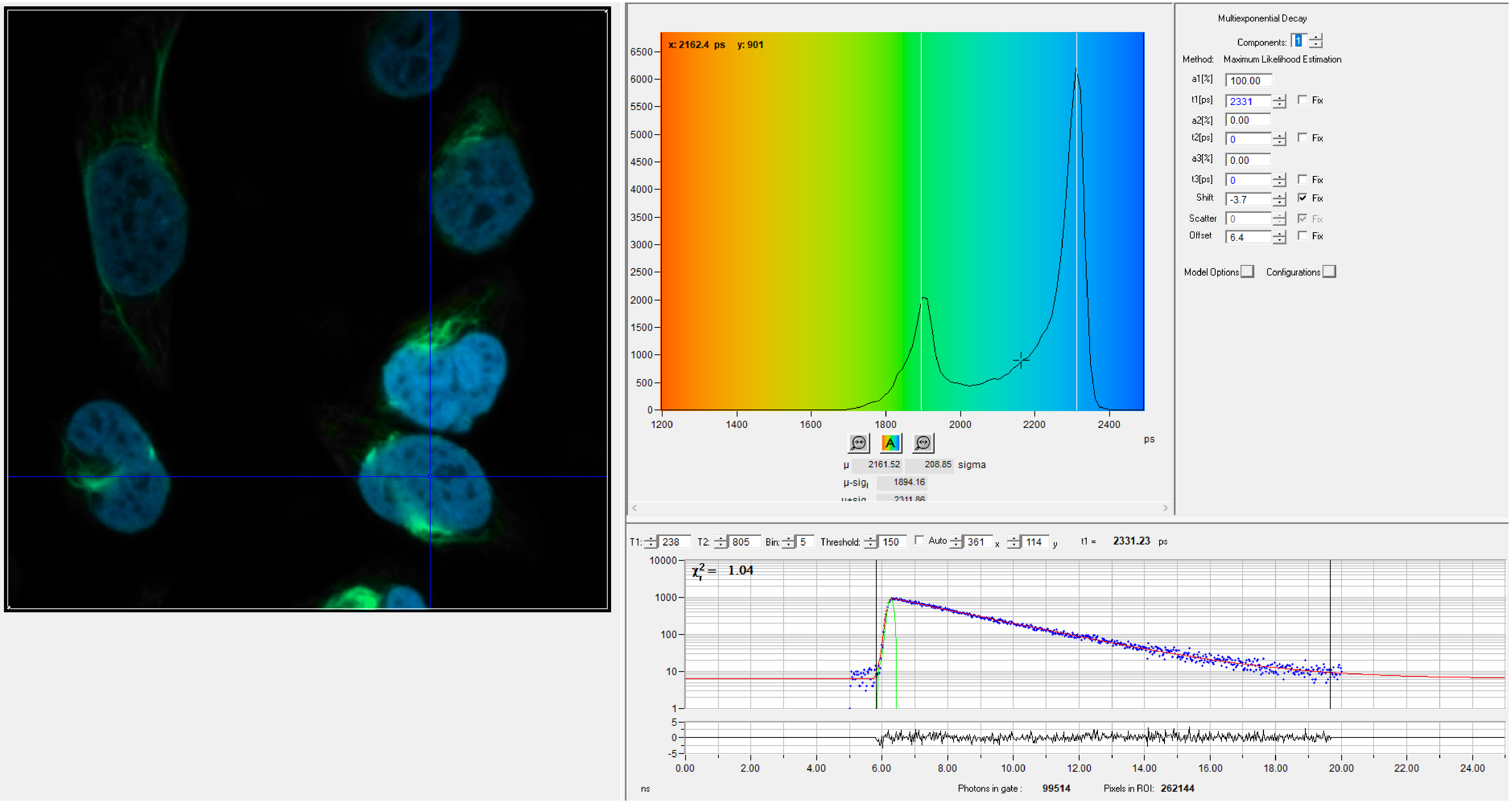

Figure S83. H2B-F62L and P68K-vimentin FAST + **HBR-2,5-DM**; monoexponential fit; τ color-coding. FLIM scan and corresponding time-resolved fluorescence data analysis of live HeLa cells expressing the H2B-F62L and P68K-vimentin FAST variants simultaneously and stained with the **HBR-2,5-DM** fluorogen. A screenshot from Becker & Hickl SPCImage data acquisition and analysis window is shown. Monoexponential fitting of decay data has been performed. On the left panel, there is a FLIM image of HeLa nuclei color-coded according to fluorescence lifetime in each pixel (τ). A histogram on the upper right panel displays the distribution of τ and color legend. The table next to it (rightmost) represents a monoexponential fitting model used to fit the data and fitting results. On the lower right panel, there are experimental decay data (blue dots), monoexponential fit of the data (red line), instrument response function (IRF) (green line) and fitting residuals (shown in black below the main data plot).

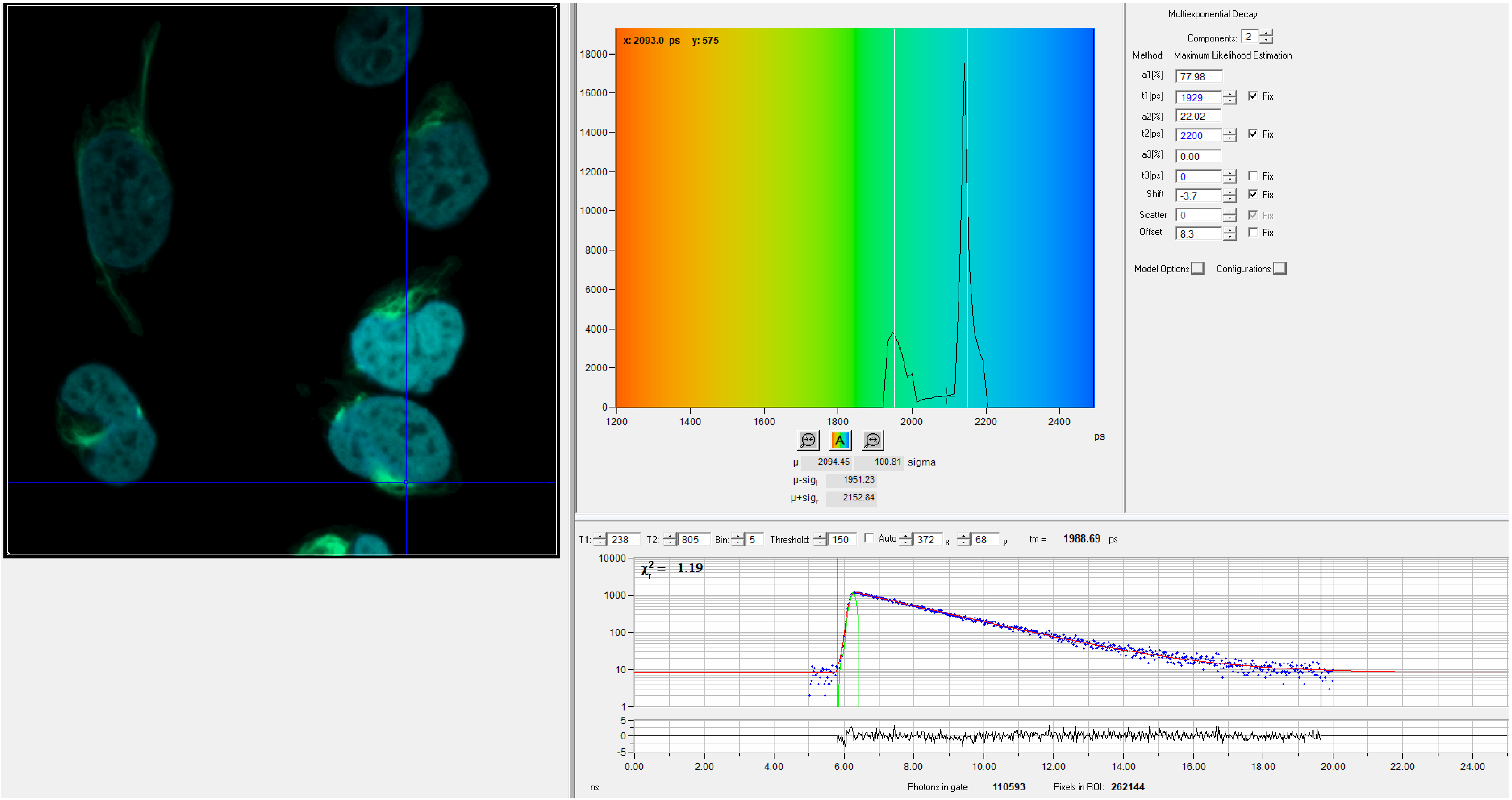

Figure S84. H2B-F62L and P68K-vimentin FAST + **HBR-2,5-DM**; biexponential fit; τ_m color-coding. FLIM scan and corresponding time-resolved fluorescence data analysis of live HeLa cells expressing the H2B-F62L and P68K-vimentin FAST variants simultaneously and stained with the **HBR-2,5-DM** fluorogen. A screenshot from Becker & Hickl SPCImage data acquisition and analysis window is shown. Biexponential fitting of decay data with both exponent components fixation at known values has been performed. On the left panel, there is a FLIM image of HeLa nuclei color-coded according to amplitude-weighted average fluorescence lifetime in each pixel (τ_m). A histogram on the upper right panel displays the distribution of τ_m and color legend. The table next to it (rightmost) represents a biexponential fitting model used to fit the data and fitting results. On the lower right panel, there are experimental decay data (blue dots), biexponential fit of the data (red line), instrument response function (IRF) (green line) and fitting residuals (shown in black below the main data plot).

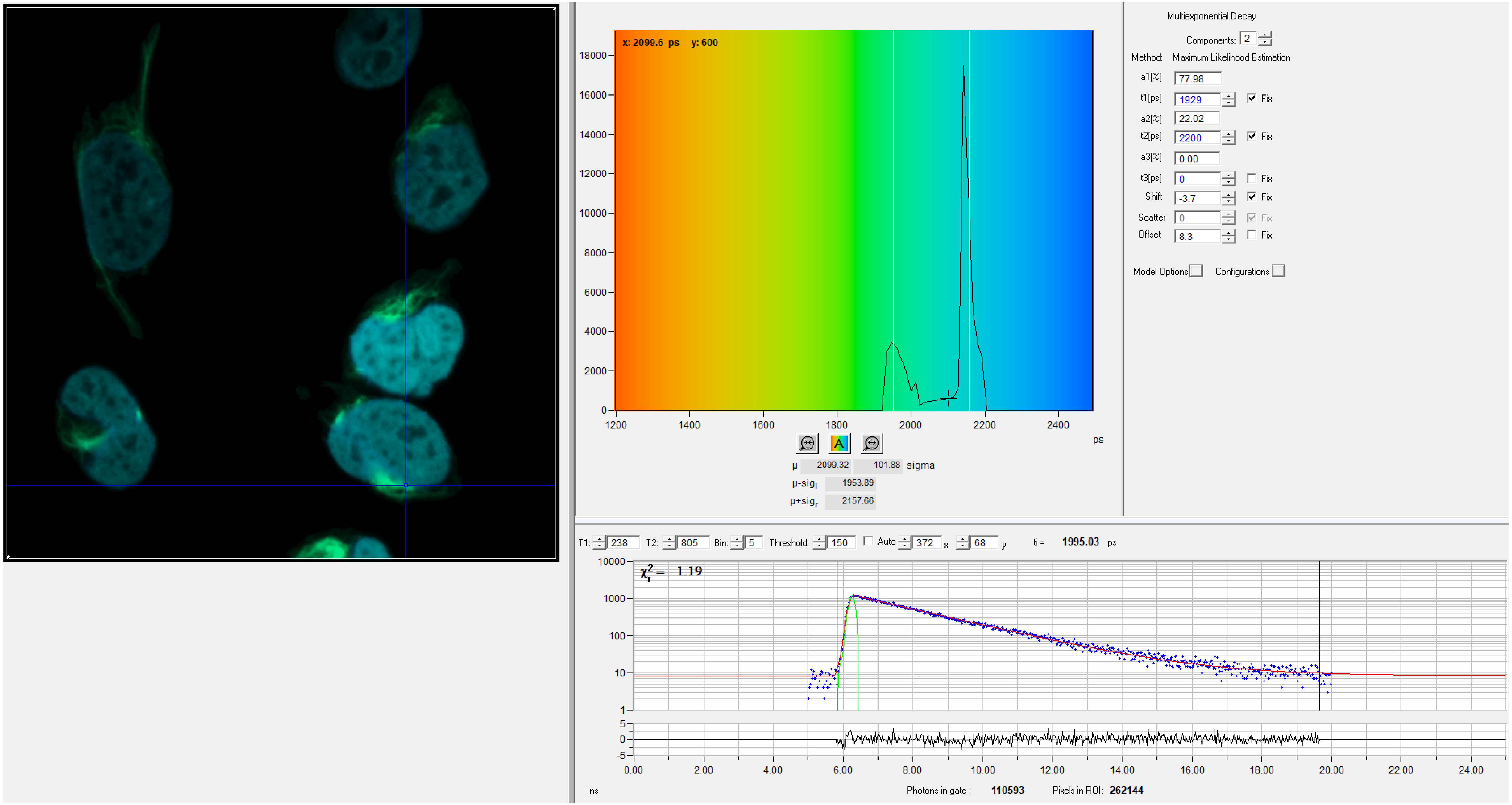

Figure S85. H2B-F62L and P68K-vimentin FAST + **HBR-2,5-DM**; biexponential fit; τ_i color-coding. FLIM scan and corresponding time-resolved fluorescence data analysis of live HeLa cells expressing the H2B-F62L and P68K-vimentin FAST variants simultaneously and stained with the **HBR-2,5-DM** fluorogen. A screenshot from Becker & Hickl SPCImage data acquisition and analysis window is shown. Biexponential fitting of decay data with both exponent components fixation at known values has been performed. On the left panel, there is a FLIM image of HeLa nuclei color-coded according to intensity-weighted average fluorescence lifetime in each pixel (τ_i). A histogram on the upper right panel displays the distribution of τ_i and color legend. The table next to it (rightmost) represents a biexponential fitting model used to fit the data and fitting results. On the lower right panel, there are experimental decay data (blue dots), biexponential fit of the data (red line), instrument response function (IRF) (green line) and fitting residuals (shown in black below the main data plot).

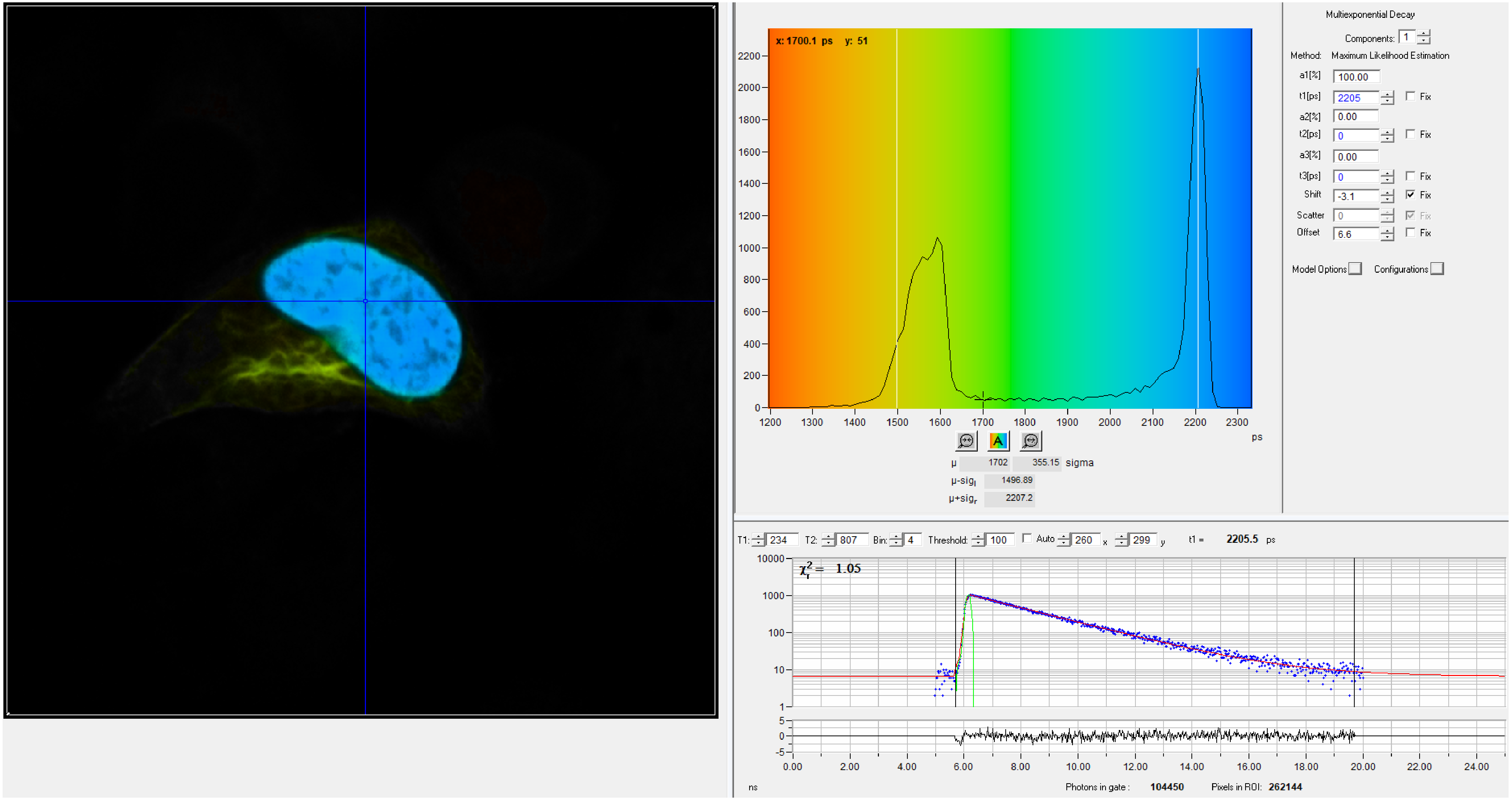

Figure S86. H2B-F62L and P68T-vimentin FAST + **HBR-2,5-DM**; monoexponential fit; τ color-coding. FLIM scan and corresponding time-resolved fluorescence data analysis of live HeLa cells expressing the H2B-F62L and P68T-vimentin FAST variants simultaneously and stained with the **HBR-2,5-DM** fluorogen. A screenshot from Becker & Hickl SPCImage data acquisition and analysis window is shown. Monoexponential fitting of decay data has been performed. On the left panel, there is a FLIM image of HeLa nuclei color-coded according to fluorescence lifetime in each pixel (τ). A histogram on the upper right panel displays the distribution of τ and color legend. The table next to it (rightmost) represents a monoexponential fitting model used to fit the data and fitting results. On the lower right panel, there are experimental decay data (blue dots), monoexponential fit of the data (red line), instrument response function (IRF) (green line) and fitting residuals (shown in black below the main data plot).

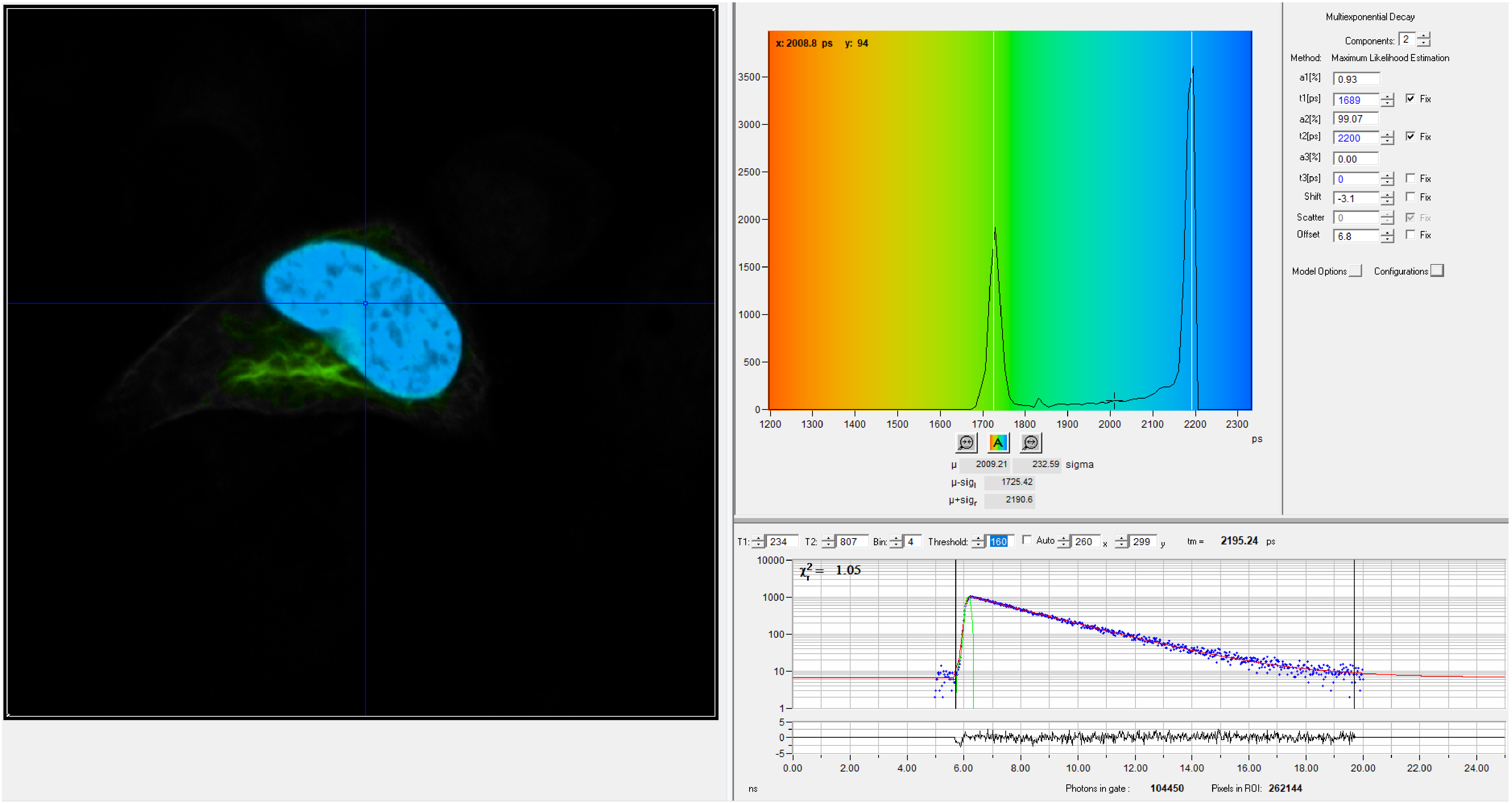

Figure S87. H2B-F62L and P68T-vimentin FAST + **HBR-2,5-DM**; biexponential fit; τ_m color-coding. FLIM scan and corresponding time-resolved fluorescence data analysis of live HeLa cells expressing the H2B-F62L and P68T-vimentin FAST variants simultaneously and stained with the **HBR-2,5-DM** fluorogen. A screenshot from Becker & Hickl SPCImage data acquisition and analysis window is shown. Biexponential fitting of decay data with both exponent components fixation at known values has been performed. On the left panel, there is a FLIM image of HeLa nuclei color-coded according to amplitude-weighted average fluorescence lifetime in each pixel (τ_m). A histogram on the upper right panel displays the distribution of τ_m and color legend. The table next to it (rightmost) represents a biexponential fitting model used to fit the data and fitting results. On the lower right panel, there are experimental decay data (blue dots), biexponential fit of the data (red line), instrument response function (IRF) (green line) and fitting residuals (shown in black below the main data plot).

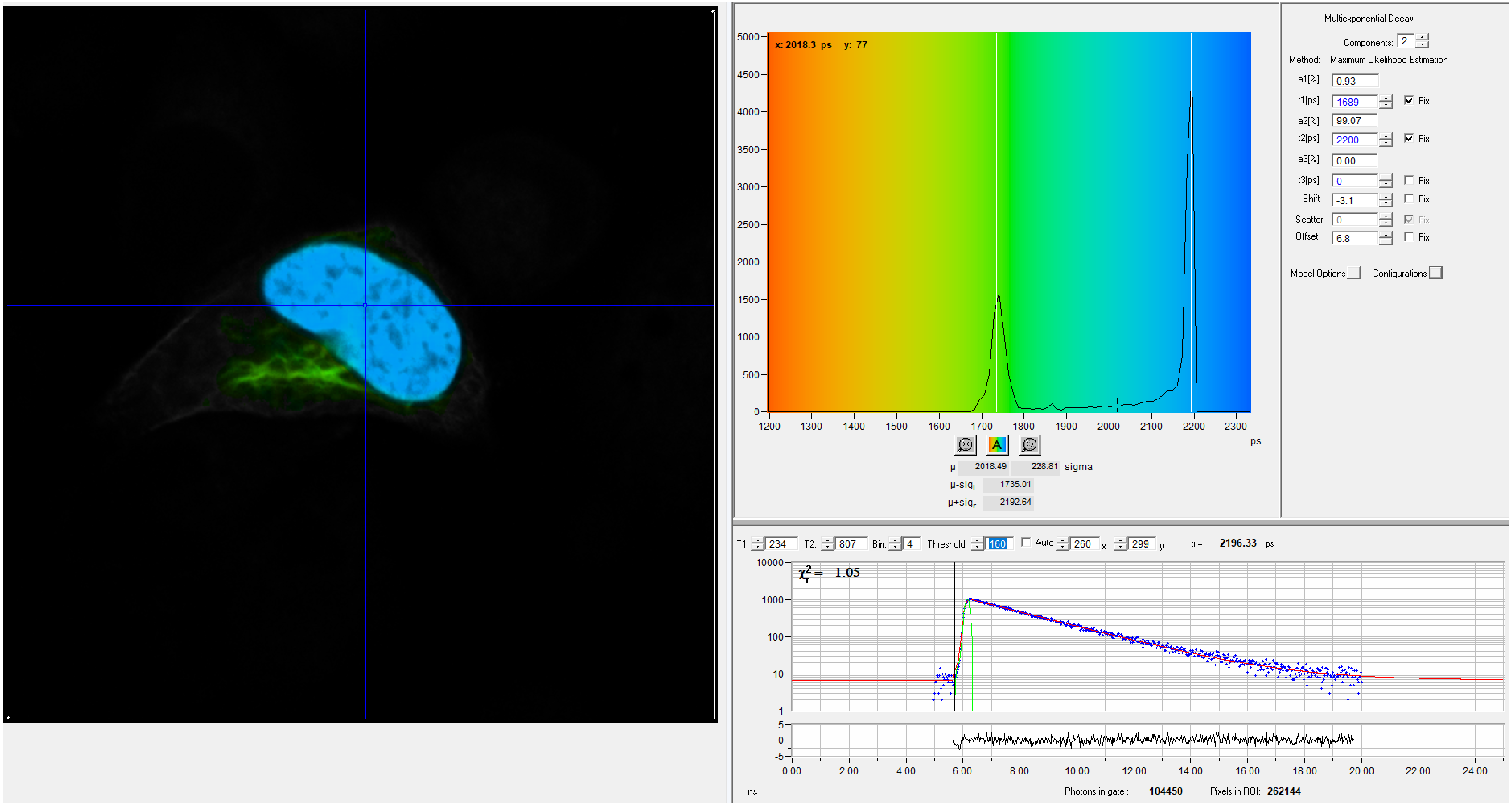

Figure S88. H2B-F62L and P68T-vimentin FAST + **HBR-2,5-DM**; biexponential fit; τ_i color-coding. FLIM scan and corresponding time-resolved fluorescence data analysis of live HeLa cells expressing the H2B-F62L and P68T-vimentin FAST variants simultaneously and stained with the **HBR-2,5-DM** fluorogen. A screenshot from Becker & Hickl SPCImage data acquisition and analysis window is shown. Biexponential fitting of decay data with both exponent components fixation at known values has been performed. On the left panel, there is a FLIM image of HeLa nuclei color-coded according to intensity-weighted average fluorescence lifetime in each pixel (τ_i). A histogram on the upper right panel displays the distribution of τ_i and color legend. The table next to it (rightmost) represents a biexponential fitting model used to fit the data and fitting results. On the lower right panel, there are experimental decay data (blue dots), biexponential fit of the data (red line), instrument response function (IRF) (green line) and fitting residuals (shown in black below the main data plot).

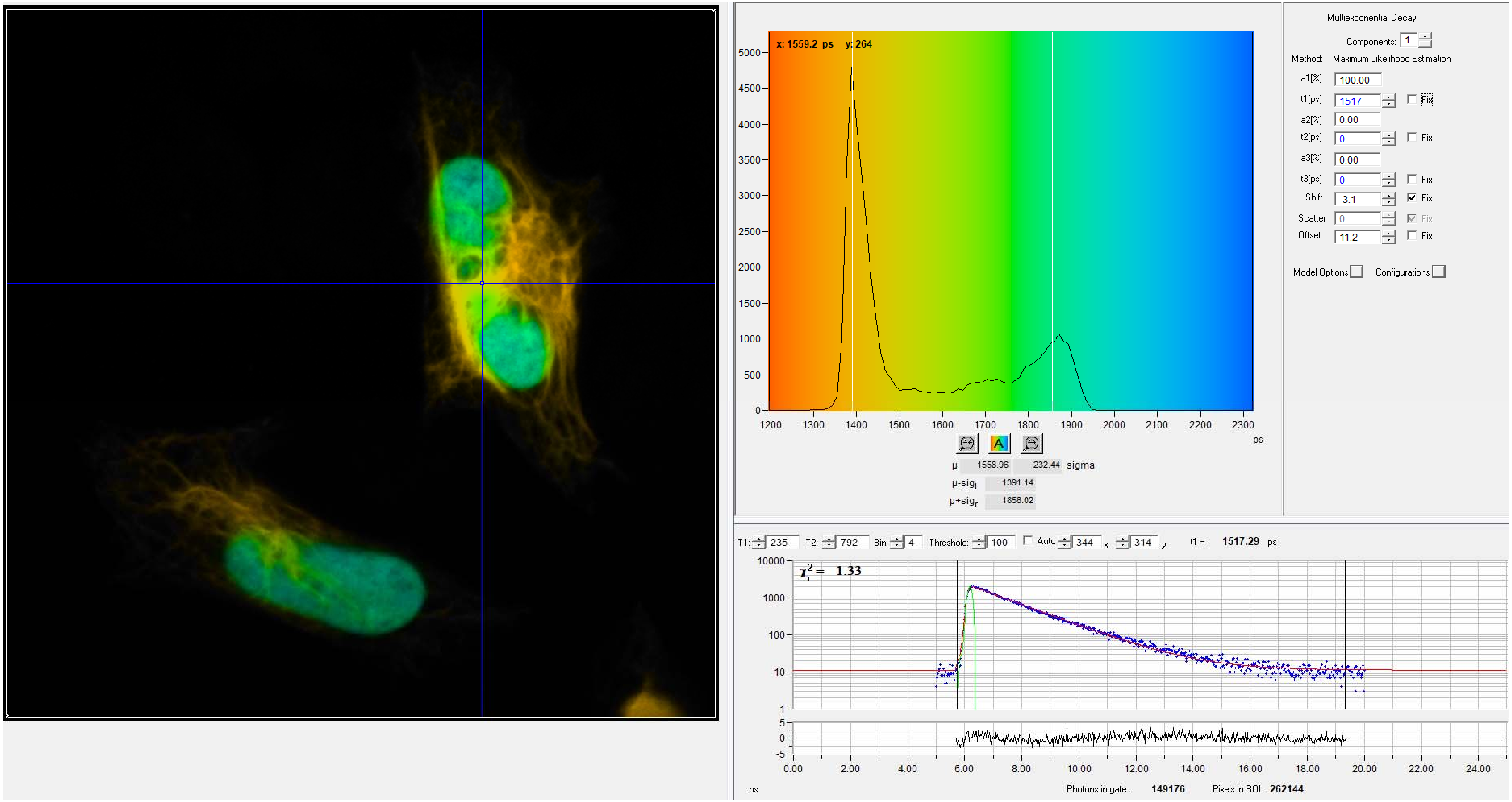

Figure S89. H2B-P68K and R52K-vimentin FAST + **HBR-2,5-DM**; monoexponential fit; τ color-coding. FLIM scan and corresponding time-resolved fluorescence data analysis of live HeLa cells expressing the H2B-P68K and R52K-vimentin FAST variants simultaneously and stained with the **HBR-2,5-DM** fluorogen. A screenshot from Becker & Hickl SPCImage data acquisition and analysis window is shown. Monoexponential fitting of decay data has been performed. On the left panel, there is a FLIM image of HeLa nuclei color-coded according to fluorescence lifetime in each pixel (τ). A histogram on the upper right panel displays the distribution of τ and color legend. The table next to it (rightmost) represents a monoexponential fitting model used to fit the data and fitting results. On the lower right panel, there are experimental decay data (blue dots), monoexponential fit of the data (red line), instrument response function (IRF) (green line) and fitting residuals (shown in black below the main data plot).

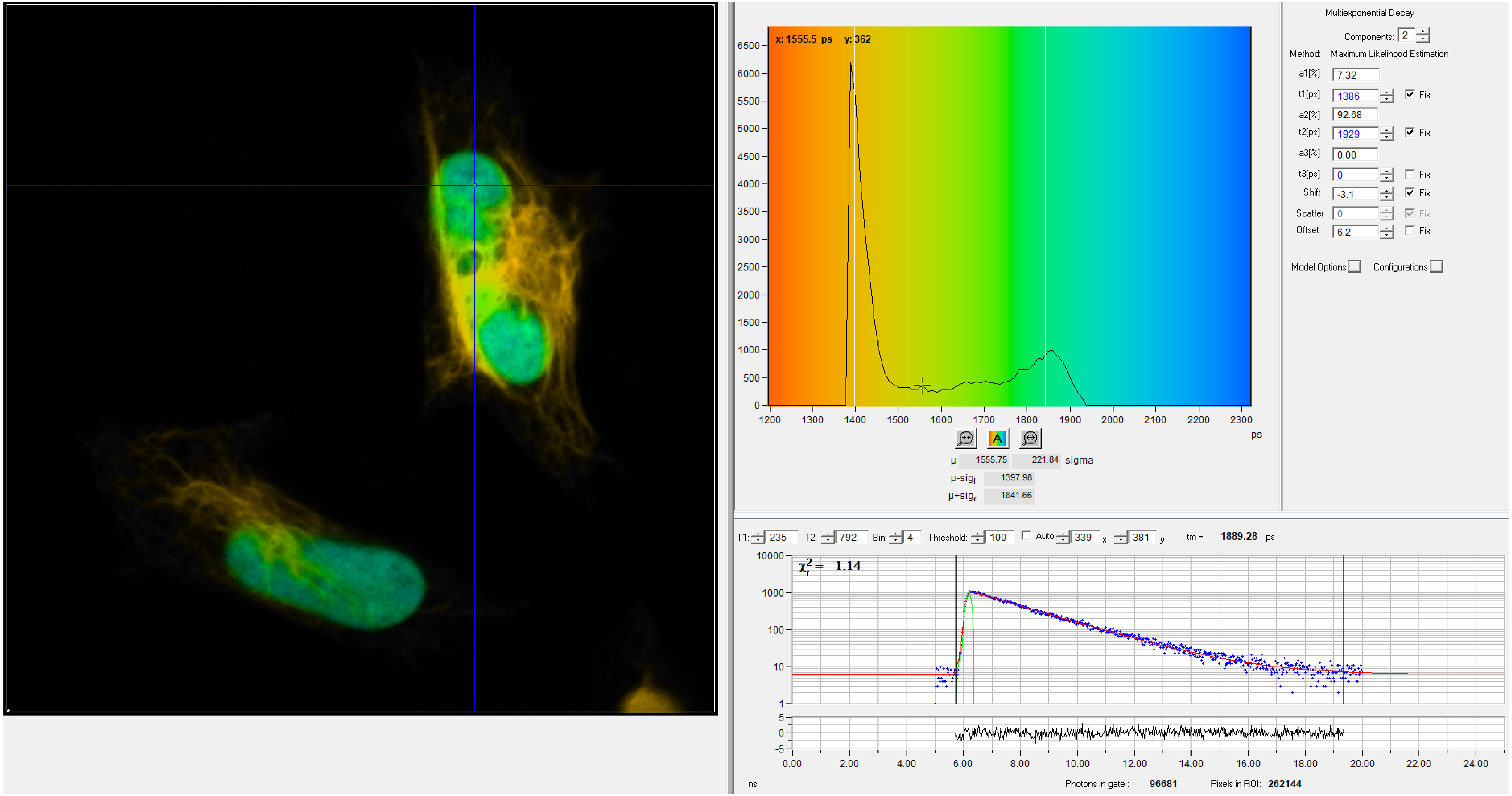

Figure S90. H2B-P68K and R52K -vimentin FAST + **HBR-2,5-DM**; biexponential fit; τ_m color-coding. FLIM scan and corresponding time-resolved fluorescence data analysis of live HeLa cells expressing the H2B-P68K and R52K-vimentin FAST variants simultaneously and stained with the **HBR-2,5-DM** fluorogen. A screenshot from Becker & Hickl SPCImage data acquisition and analysis window is shown. Biexponential fitting of decay data with both exponent components fixation at known values has been performed. On the left panel, there is a FLIM image of HeLa nuclei color-coded according to amplitude-weighted average fluorescence lifetime in each pixel (τ_m). A histogram on the upper right panel displays the distribution of τ_m and color legend. The table next to it (rightmost) represents a biexponential fitting model used to fit the data and fitting results. On the lower right panel, there are experimental decay data (blue dots), biexponential fit of the data (red line), instrument response function (IRF) (green line) and fitting residuals (shown in black below the main data plot).

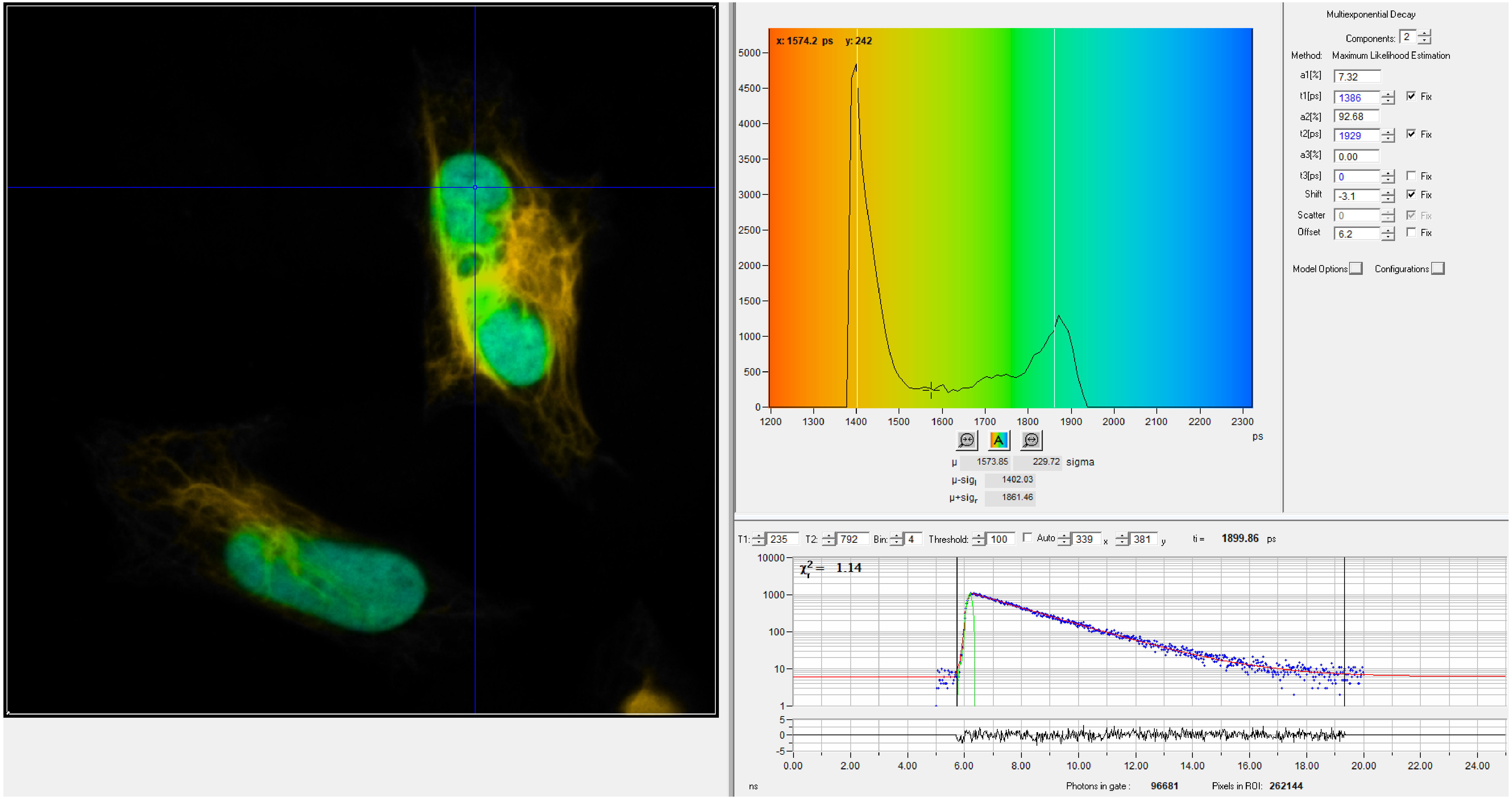

Figure S91. H2B-P68K and R52K -vimentin FAST + **HBR-2,5-DM**; biexponential fit; τ_i color-coding. FLIM scan and corresponding time-resolved fluorescence data analysis of live HeLa cells expressing the H2B-P68K and R52K-vimentin FAST variants simultaneously and stained with the **HBR-2,5-DM** fluorogen. A screenshot from Becker & Hickl SPCImage data acquisition and analysis window is shown. Biexponential fitting of decay data with both exponent components fixation at known values has been performed. On the left panel, there is a FLIM image of HeLa nuclei color-coded according to intensity-weighted average fluorescence lifetime in each pixel (τ_i). A histogram on the upper right panel displays the distribution of τ_i and color legend. The table next to it (rightmost) represents a biexponential fitting model used to fit the data and fitting results. On the lower right panel, there are experimental decay data (blue dots), biexponential fit of the data (red line), instrument response function (IRF) (green line) and fitting residuals (shown in black below the main data plot).

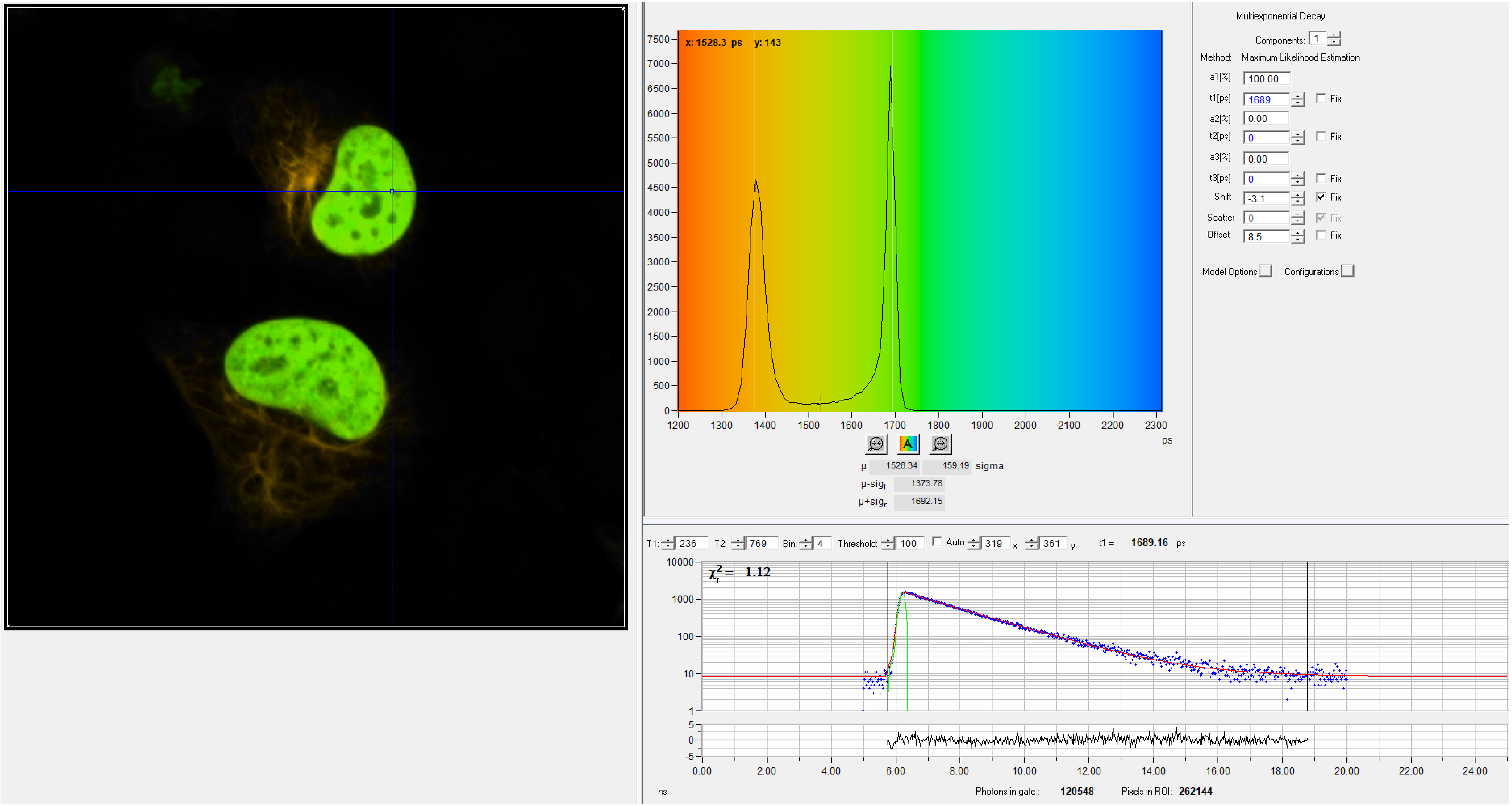

Figure S92. H2B-P68T and R52K-vimentin FAST + **HBR-2,5-DM**; monoexponential fit; τ color-coding. FLIM scan and corresponding time-resolved fluorescence data analysis of live HeLa cells expressing the H2B-P68T and R52K-vimentin FAST variants simultaneously and stained with the **HBR-2,5-DM** fluorogen. A screenshot from Becker & Hickl SPCImage data acquisition and analysis window is shown. Monoexponential fitting of decay data has been performed. On the left panel, there is a FLIM image of HeLa nuclei color-coded according to fluorescence lifetime in each pixel (τ). A histogram on the upper right panel displays the distribution of τ and color legend. The table next to it (rightmost) represents a monoexponential fitting model used to fit the data and fitting results. On the lower right panel, there are experimental decay data (blue dots), monoexponential fit of the data (red line), instrument response function (IRF) (green line) and fitting residuals (shown in black below the main data plot).

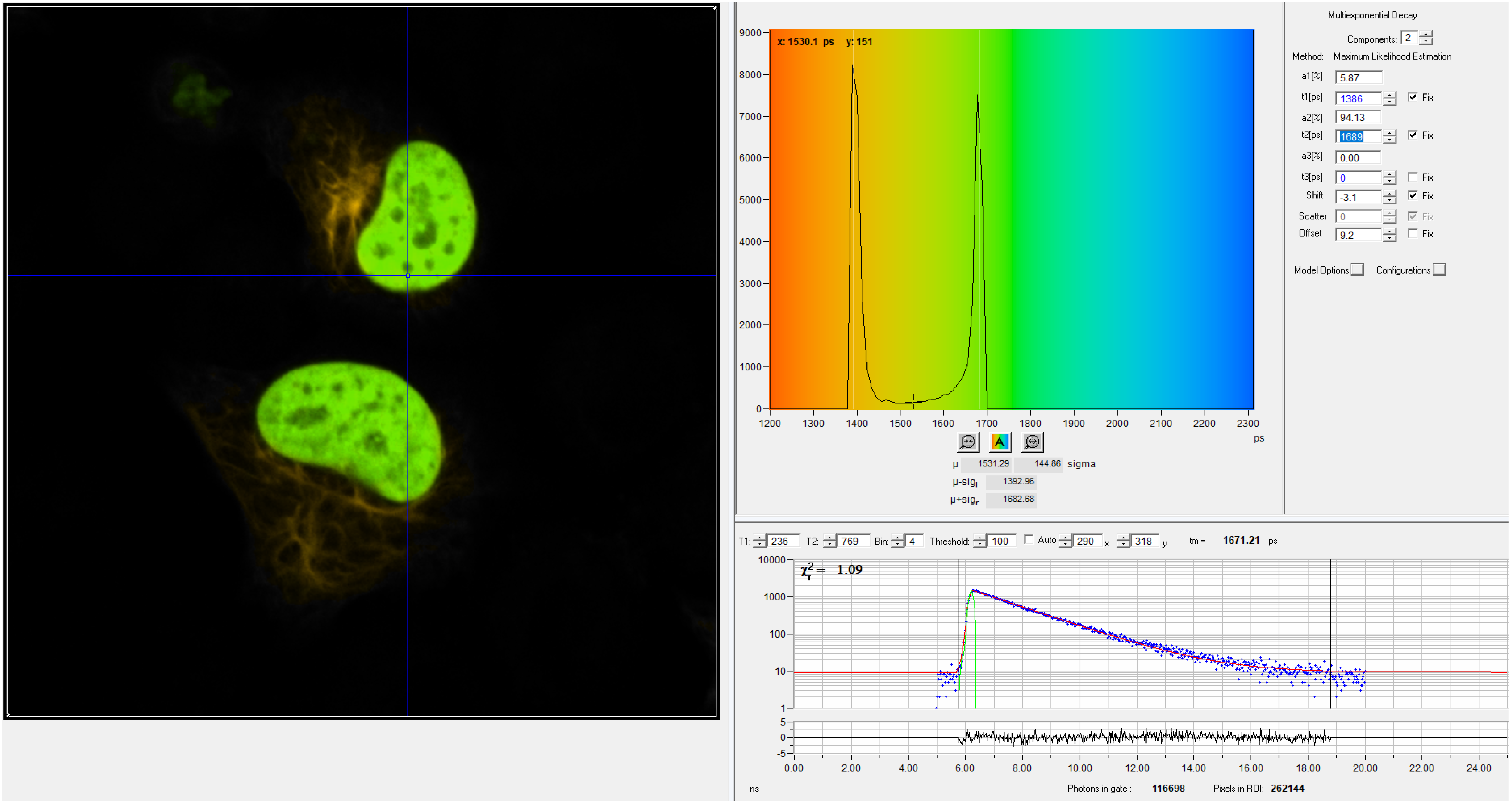

Figure S93. H2B-P68T and R52K -vimentin FAST + **HBR-2,5-DM**; biexponential fit; τ_m color-coding. FLIM scan and corresponding time-resolved fluorescence data analysis of live HeLa cells expressing the H2B-P68T and R52K-vimentin FAST variants simultaneously and stained with the **HBR-2,5-DM** fluorogen. A screenshot from Becker & Hickl SPCImage data acquisition and analysis window is shown. Biexponential fitting of decay data with both exponent components fixation at known values has been performed. On the left panel, there is a FLIM image of HeLa nuclei color-coded according to amplitude-weighted average fluorescence lifetime in each pixel (τ_m). A histogram on the upper right panel displays the distribution of τ_m and color legend. The table next to it (rightmost) represents a biexponential fitting model used to fit the data and fitting results. On the lower right panel, there are experimental decay data (blue dots), biexponential fit of the data (red line), instrument response function (IRF) (green line) and fitting residuals (shown in black below the main data plot).

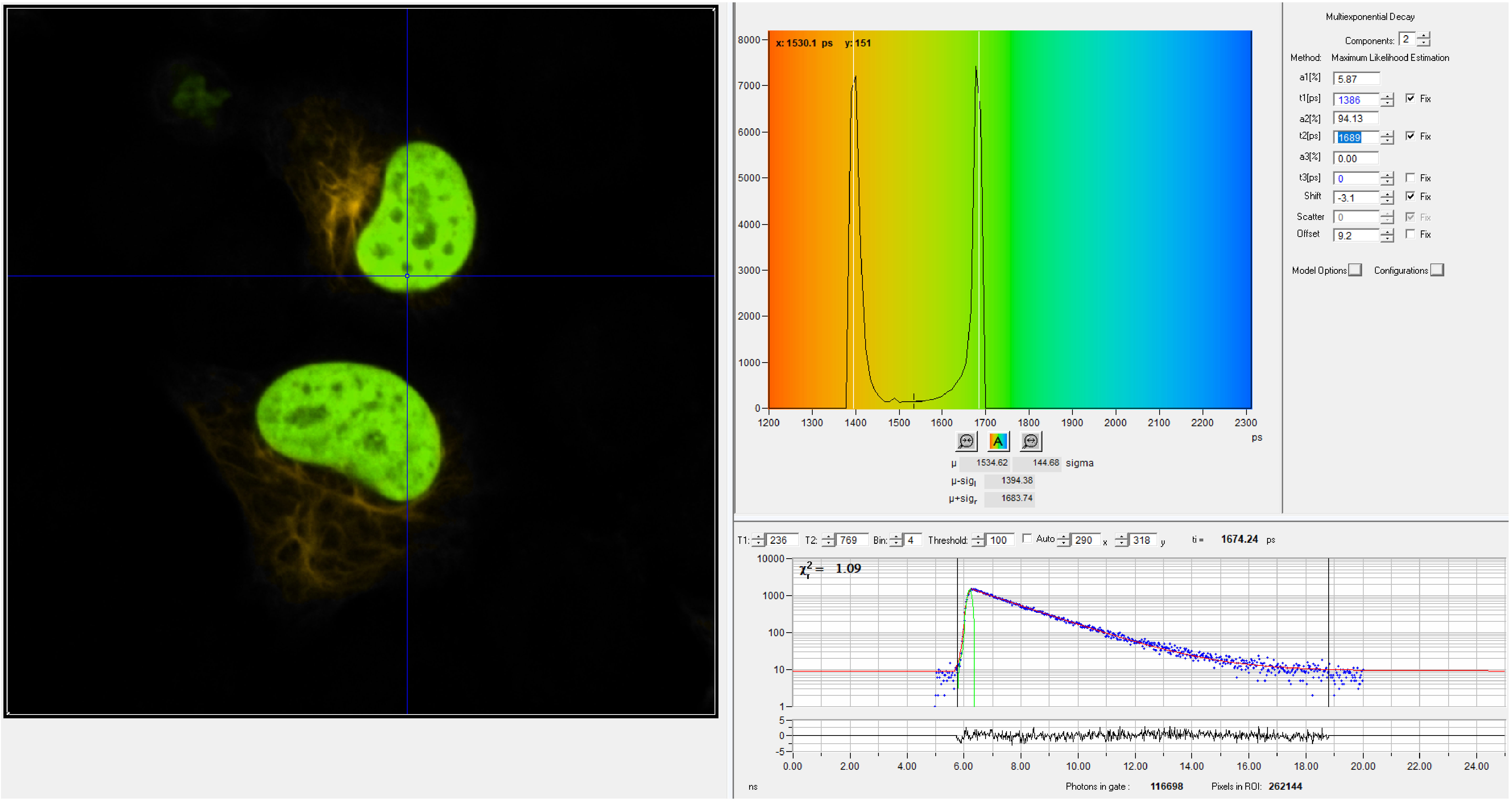

Figure S94. H2B-P68T and R52K -vimentin FAST + **HBR-2,5-DM**; biexponential fit; τ_i color-coding. FLIM scan and corresponding time-resolved fluorescence data analysis of live HeLa cells expressing the H2B-P68T and R52K-vimentin FAST variants simultaneously and stained with the **HBR-2,5-DM** fluorogen. A screenshot from Becker & Hickl SPCImage data acquisition and analysis window is shown. Biexponential fitting of decay data with both exponent components fixation at known values has been performed. On the left panel, there is a FLIM image of HeLa nuclei color-coded according to intensity-weighted average fluorescence lifetime in each pixel (τ_i). A histogram on the upper right panel displays the distribution of τ_i and color legend. The table next to it (rightmost) represents a biexponential fitting model used to fit the data and fitting results. On the lower right panel, there are experimental decay data (blue dots), biexponential fit of the data (red line), instrument response function (IRF) (green line) and fitting residuals (shown in black below the main data plot).

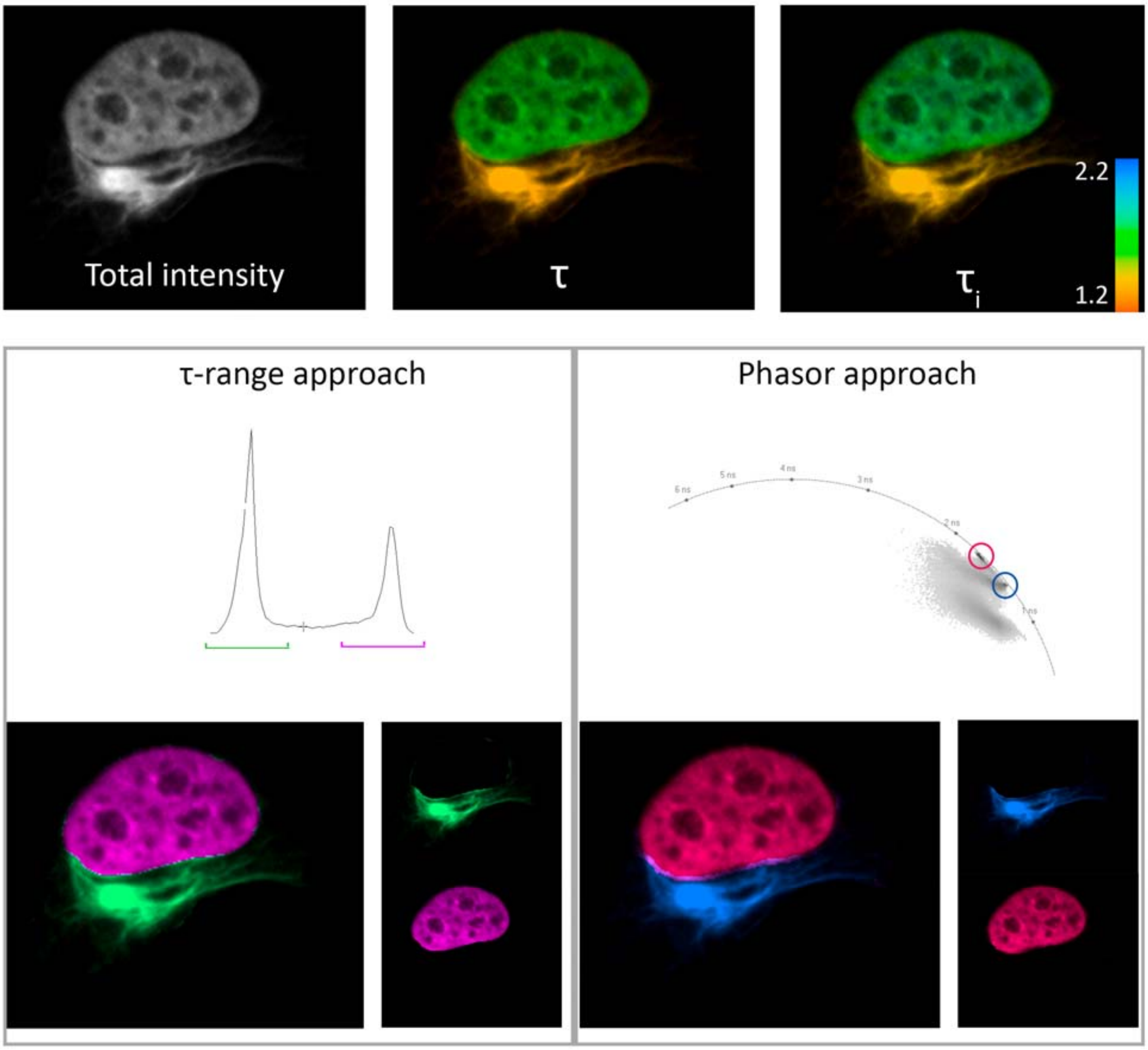

Figure S95. Time-resolved fluorescence data analysis of live HeLa cells expressing the H2B-P68T and R52K-vimentin FAST variants simultaneously and stained with the **HBR-2,5-DM** fluorogen. Representative total intensity, FLIM image color-coded according to τ (monoexponential fitting) and τ_i (biexponential fitting) are given in the first row. Two grey boxes below contain composite and individual images with the separated structures obtained by fitting-based (τ-ranged approach) and non-fitting based (phasor approach) methods. τ-range for P68T variant was taken as 1.6-1.8 ns, for R52K τ-range was taken as 1.3-1.6 ns.

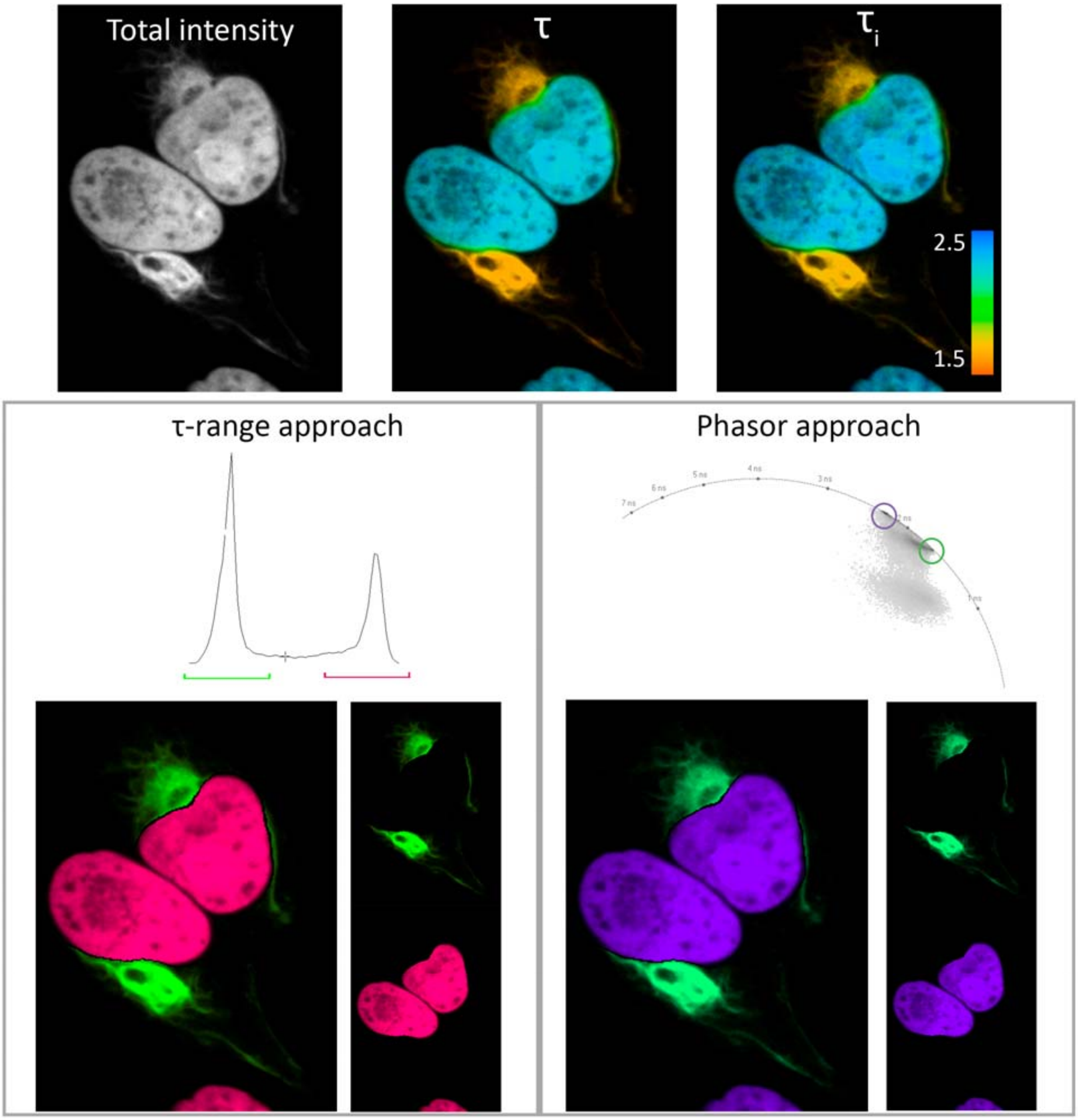

Figure S96. Time-resolved fluorescence data analysis of live HeLa cells expressing the H2B-F62L and P68T-vimentin FAST variants simultaneously and stained with the **HBR-2,5-DM** fluorogen. Representative total intensity, FLIM image color-coded according to τ (monoexponential fitting) and τ_i (biexponential fitting) are given in the first row. Two grey boxes below contain composite and individual images with the separated structures obtained by fitting-based (τ-ranged approach) and non-fitting based (phasor approach) methods. τ-range for P68T variant was taken as 1.6-1.8 ns, for F62L τ-range was taken as 1.8-2.3 ns.

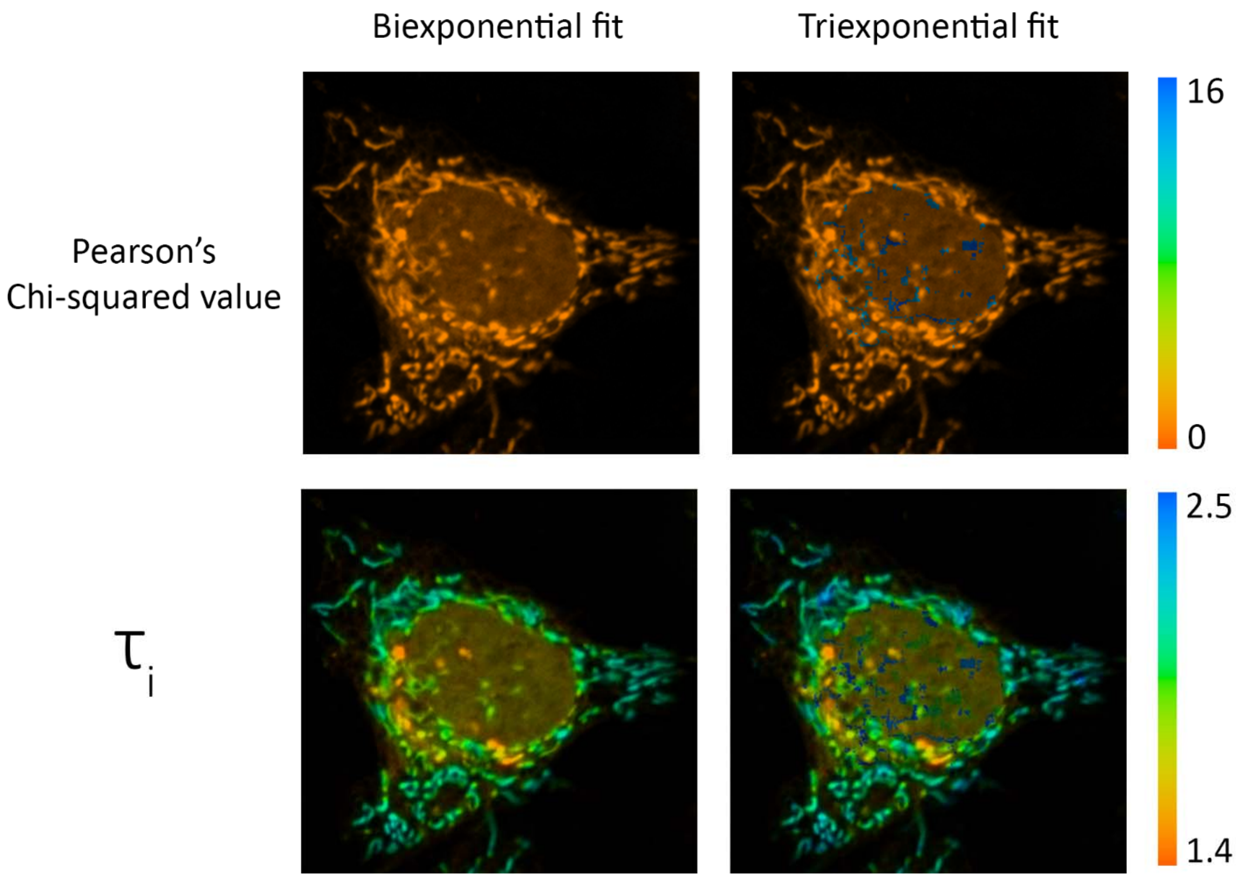

Figure S97. FLIM images with bi- or triexponential fit of **HBR-2,5-DM** in complexes with three FAST variants expressed simultaneously in live HeLa cells as H2B-P68T, IMS-F62L (mitochondrial intermembrane space), and β4Gal-T1-R52K (Golgi apparatus) fuses. Top: Color-coding represents Pearson's chi-squared test value. Blue areas of very high (>10) chi-square values are presented in the image fitted with the triexponential function, but not in that fitted with the biexponential one. Bottom: Color-coding represents τ_i. Coloration artifacts are seen at the areas corresponding to high chi-square values.

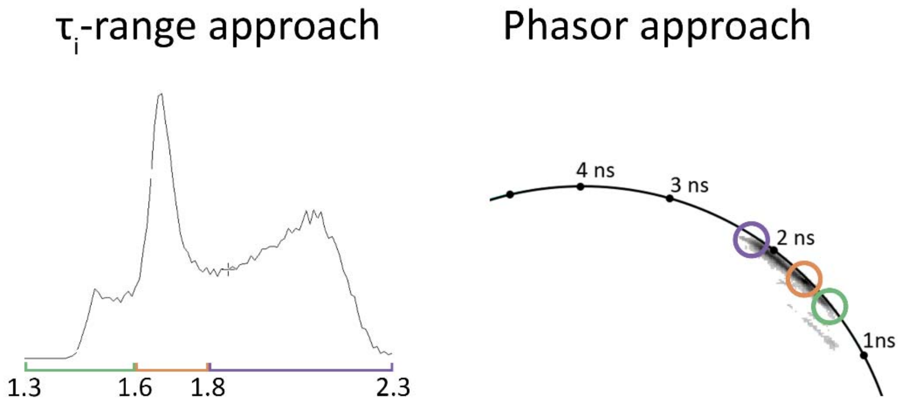

Figure S98. τ_i histogram and phasor plot with color-coding of τi ranges and phasor clusters corresponds to Figure 6 in the main text.

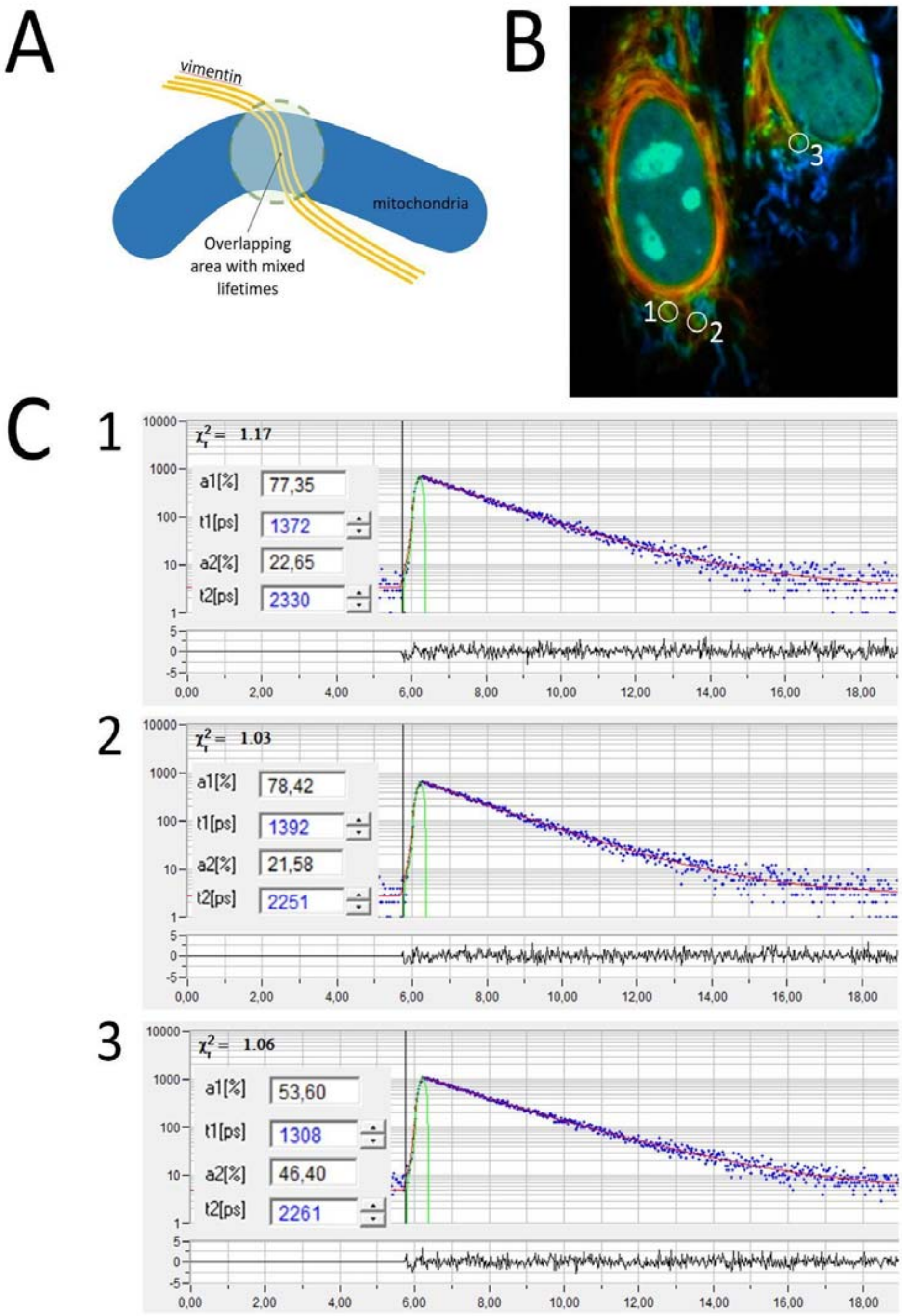

Figure S99. Example of the fitting-based analysis of the time-resolved fluorescence signals produced by the spatially overlapped FAST-fluorogen labels. A – Schematic depicting a principle of the spatial overlapping between vimentin- and mitochondrion-targeted fluorescent probes; B – Color-coded FLIM-scan of the cells stained with the **HBR-2,5-DM** fluorogen and co-expressing three differentially targeted FAST variants (vimentin-R52K, IMS-F62L and H2B-P68K). Encircled areas represent the three regions of interest (ROIs), where the signals from vimentin-R52K and IMS- F62L are overlapped; C – Consecutively placed screenshots from the Becker & Hickl SPCImage data acquisition and analysis window showing the fluorescence decay data analysis for the chosen ROIs. In all cases, a two-component fitting model without values fixation adequately describes the decay data, and gives the lifetime values similar to those determined for the **HBR-2,5-DM**-R52K and **HBR-2,5-DM**-F62L single probe labeling.

Table S1. Amino acid sequences

**FAST**<br>(original)	(M)EHVAFGSEDIENTLAKMDDGQLDGLAFGAIQLDGDGNILQYNAAEGDITGRDP<br>KQVIGKNFFKDVAPGTDSPEFYGKFKEGVASGNLNTMFEWMIPTSRGPTKVKVHM<br>KKALSGDSYWVFVKRV(GGGHHHHHH)
**F62L**	(M)EHVAFGSEDIENTLAKMDDGQLDGLAFGAIQLDGDGNILQYNAAEGDITGRDP<br>KQVIGKN**L**FKDVAPGTDSPEFYGKFKEGVASGNLNTMFEWMIPTSRGPTKVKVHM<br>KKALSGDSYWVFVKRV(GGGHHHHHH)
**P68K**	(M)EHVAFGSEDIENTLAKMDDGQLDGLAFGAIQLDGDGNILQYNAAEGDITGRDP<br>KQVIGKNFFKDVA**K**GTDSPEFYGKFKEGVASGNLNTMFEWMIPTSRGPTKVKVHM<br>KKALSGDSYWVFVKRV(GGGHHHHHH)
**P68T**	(M)EHVAFGSEDIENTLAKMDDGQLDGLAFGAIQLDGDGNILQYNAAEGDITGRDP<br>KQVIGKNFFKDVA**T**GTDSPEFYGKFKEGVASGNLNTMFEWMIPTSRGPTKVKVHM<br>KKALSGDSYWVFVKRV(GGGHHHHHH)
**R52K**	(M)EHVAFGSEDIENTLAKMDDGQLDGLAFGAIQLDGDGNILQYNAAEGDITG**K**DP<br>KQVIGKNFFKDVAPGTDSPEFYGKFKEGVASGNLNTMFEWMIPTSRGPTKVKVHM<br>KKALSGDSYWVFVKRV(GGGHHHHHH)
**D65K**	(M)EHVAFGSEDIENTLAKMDDGQLDGLAFGAIQLDGDGNILQYNAAEGDITGRDP<br>KQVIGKNFFK**K**VAPGTDSPEFYGKFKEGVASGNLNTMFEWMIPTSRGPTKVKVHM<br>KKALSGDSYWVFVKRV(GGGHHHHHH)
**D65R**	(M)EHVAFGSEDIENTLAKMDDGQLDGLAFGAIQLDGDGNILQYNAAEGDITGRDP<br>KQVIGKNFFK**R**VAPGTDSPEFYGKFKEGVASGNLNTMFEWMIPTSRGPTKVKVHM<br>KKALSGDSYWVFVKRV(GGGHHHHHH)
**P68R**	(M)EHVAFGSEDIENTLAKMDDGQLDGLAFGAIQLDGDGNILQYNAAEGDITGRDP<br>KQVIGKNFFKDVA**R**GTDSPEFYGKFKEGVASGNLNTMFEWMIPTSRGPTKVKVHM<br>KKALSGDSYWVFVKRV(GGGHHHHHH)
**P73S**	(M)EHVAFGSEDIENTLAKMDDGQLDGLAFGAIQLDGDGNILQYNAAEGDITGRDP<br>KQVIGKNFFKDVAPGTDS**S**EFYGKFKEGVASGNLNTMFEWMIPTSRGPTKVKVHM<br>KKALSGDSYWVFVKRV(GGGHHHHHH)
**P97T**	(M)EHVAFGSEDIENTLAKMDDGQLDGLAFGAIQLDGDGNILQYNAAEGDITGRDP<br>KQVIGKNFFKDVAPGTDSPEFYGKFKEGVASGNLNTMFEWMI**T**TSRGPTKVKVHM<br>KKALSGDSYWVFVKRV(GGGHHHHHH)
**P97T/T98G**	(M)EHVAFGSEDIENTLAKMDDGQLDGLAFGAIQLDGDGNILQYNAAEGDITGRDP<br>KQVIGKNFFKDVAPGTDSPEFYGKFKEGVASGNLNTMFEWMI**TG**SRGPTKVKVHM<br>KKALSGDSYWVFVKRV(GGGHHHHHH)
**R52A**	(M)EHVAFGSEDIENTLAKMDDGQLDGLAFGAIQLDGDGNILQYNAAEGDITG**A**DP<br>KQVIGKNFFKDVAPGTDSPEFYGKFKEGVASGNLNTMFEWMIPTSRGPTKVKVHM<br>KKALSGDSYWVFVKRV(GGGHHHHHH)
**R52E**	(M)EHVAFGSEDIENTLAKMDDGQLDGLAFGAIQLDGDGNILQYNAAEGDITG**E**DP<br>KQVIGKNFFKDVAPGTDSPEFYGKFKEGVASGNLNTMFEWMIPTSRGPTKVKVHM<br>KKALSGDSYWVFVKRV(GGGHHHHHH)
**R52E/D65R**	(M)EHVAFGSEDIENTLAKMDDGQLDGLAFGAIQLDGDGNILQYNAAEGDITG**E**DP<br>KQVIGKNFFK**R**VAPGTDSPEFYGKFKEGVASGNLNTMFEWMIPTSRGPTKVKVHM<br>KKALSGDSYWVFVKRV(GGGHHHHHH)
**R52F**	(M)EHVAFGSEDIENTLAKMDDGQLDGLAFGAIQLDGDGNILQYNAAEGDITG**F**DP<br>KQVIGKNFFKDVAPGTDSPEFYGKFKEGVASGNLNTMFEWMIPTSRGPTKVKVHM<br>KKALSGDSYWVFVKRV(GGGHHHHHH)

**R52L**	(M)EHVAFGSEDIENTLAKMDDGQLDGLAFGAIQLDGDGNILQYNAAEGDITG**L**DP KQVIGKNFFKDVAPGTDSPEFYGKFKEGVASGNLNTMFEWMIPTSRGPTKVKVHM KKALSGDSYWVFVKRV(GGGHHHHHH)
**R52Y**	(M)EHVAFGSEDIENTLAKMDDGQLDGLAFGAIQLDGDGNILQYNAAEGDITG**Y**DP KQVIGKNFFKDVAPGTDSPEFYGKFKEGVASGNLNTMFEWMIPTSRGPTKVKVHM KKALSGDSYWVFVKRV(GGGHHHHHH)
**R52D**	(M)EHVAFGSEDIENTLAKMDDGQLDGLAFGAIQLDGDGNILQYNAAEGDITG**D**DP KQVIGKNFFKDVAPGTDSPEFYGKFKEGVASGNLNTMFEWMIPTSRGPTKVKVHM KKALSGDSYWVFVKRV(GGGHHHHHH)
**S99E**	(M)EHVAFGSEDIENTLAKMDDGQLDGLAFGAIQLDGDGNILQYNAAEGDITGRDP KQVIGKNFFKDVAPGTDSPEFYGKFKEGVASGNLNTMFEWMIPT**E**RGPTKVKVHM KKALSGDSYWVFVKRV(GGGHHHHHH)
**S99K**	(M)EHVAFGSEDIENTLAKMDDGQLDGLAFGAIQLDGDGNILQYNAAEGDITGRDP KQVIGKNFFKDVAPGTDSPEFYGKFKEGVASGNLNTMFEWMIPT**K**RGPTKVKVHM KKALSGDSYWVFVKRV(GGGHHHHHH)
**S99R**	(M)EHVAFGSEDIENTLAKMDDGQLDGLAFGAIQLDGDGNILQYNAAEGDITGRDP KQVIGKNFFKDVAPGTDSPEFYGKFKEGVASGNLNTMFEWMIPT**R**RGPTKVKVHM KKALSGDSYWVFVKRV(GGGHHHHHH)
**V107I**	(M)EHVAFGSEDIENTLAKMDDGQLDGLAFGAIQLDGDGNILQYNAAEGDITGRDP KQVIGKNFFKDVAPGTDSPEFYGKFKEGVASGNLNTMFEWMIPTSRGPTKVK**I**HM KKALSGDSYWVFVKRV(GGGHHHHHH)
**nanoFAST***	(M)FGAIQLDGDGNILQYNAAEGDITGRDPKQ VIGKNFFKDVAPGTDSPEFYGKFKEGVASGNLNTMFEWMIPTSRGPTKVKVHMKK ALSGDSYWVFVKRV

* nanoFAST protein was not used in the present work, the sequence presented for comparison.

Table S2. Brightness and fluorescence lifetime data of FAST variants with **HBR-2,5-DM** fluorogen. τ_φ – average phase lifetime ± SD, τ_m – average modulation lifetime ± SD.

**FAST variant**	**Brightness, r.u.**	**τ_φ, ns**	**τ_m, ns**
R52L	2711±80	2.23±0.08	2.87±0.42
P73S	3998±183	2.65±0.05	2.87±0.31
R52E D65R	2377±59	2.55±0.07	3.19±0.3
S99E	4750±326	2.64±0.06	2.82±0.33
P97T	1308±63	2.93±0.12	4.11±0.24
D65K	3796±311	2.56±0.06	2.77±0.36
D65R	3597±144	2.65±0.06	2.91±0.35
R52A	3582±113	2.43±0.07	2.79±0.38
S99R	4283±404	2.65±0.05	2.86±0.33
R52E	3455±82	2.45±0.05	2.87±0.34
R52Y	2513±89	2.49±0.07	3.14±0.36
F62L	4059±302	2.70±0.05	3.09±0.29
P68K	3727±198	2.48±0.06	2.95±0.38
S99K	4216±221	2.59±0.06	2.95±0.35
R52D	1594±38	3.13±0.09	3.97±0.2
R52K	3360±126	2.18±0.06	2.99±0.4
P97T T98G	1069±25	-[a]	-[a]
P68R	3590±280	2.43±0.06	2.96±0.41
V107I	4594±365	2.61±0.06	2.95±0.35
P68T	3535±188	2.30±0.11	2.90±0.44
R52F	2382±71	2.47±0.08	3.38±0.41
FAST (original)	4630±285	2.80±0.06	3.08±0.31

a - Low brightness, fluorescence lifetime cannot be determined accurately.

Table S3. Brightness and fluorescence lifetime data of FAST variants with **HMBR** fluorogen. τ_φ – average phase lifetime ± SD, τ_m – average modulation lifetime ± SD.

**FAST variant**	**Brightness, r.u.**	**τ_φ, ns**	**τ_m, ns**
R52L	2281±195	2.25±0.08	2.83±0.41
P73S	3175±125	2.41±0.06	2.71±0.38
R52E D65R	1422±88	2.86±0.11	3.67±0.27
S99E	3807±169	2.43±0.06	2.68±0.38
P97T	954±94	-[a]	-[a]
D65K	3089±354	2.38±0.06	2.67±0.41
D65R	2928±125	2.46±0.06	2.80±0.39
R52A	2658±121	2.39±0.06	2.89±0.42
S99R	3875±191	2.36±0.05	2.59±0.38
R52E	2478±111	2.51±0.07	3.00±0.40
R52Y	1700±63	2.57±0.08	3.33±0.34
F62L	3081±128	2.52±0.06	3.00±0.39
P68K	3421±147	2.16±0.06	2.79±0.42
S99K	3578±391	2.41±0.07	2.93±0.39
R52D	1197±38	-[a]	-[a]
R52K	2824±137	2.13±0.07	3.00±0.42
P97T T98G	751±89	-[a]	-[a]
P68R	2644±258	2.19±0.10	2.96±0.46
V107I	3533±316	2.35±0.07	2.88±0.41
P68T	3157±205	2.06±0.08	2.84±0.44
R52F	1825±129	2.47±0.09	3.48±0.32
FAST (original)	4703±179	2.60±0.05	2.97±0.31

a - Low brightness, fluorescence lifetime cannot be determined accurately.

Table S4. Brightness and fluorescence lifetime data of FAST variants with **HBR-DOM2** fluorogen. τ_{φ} – average phase lifetime ± SD, τ_{m} – average modulation lifetime ± SD.

**FAST variant**	**Brightness, r.u.**	**τ_{φ}, ns**	**τ_{m}, ns**
R52L	2741±234	3.54±0.09	3.90±0.16
P73S	3432±411	3.54±0.09	3.73±0.15
R52E D65R	2987±323	3.55±0.09	3.83±0.16
S99E	3758±561	3.54±0.10	3.73±0.15
P97T	1572±117	2.60±0.13	3.75±0.30
D65K	3209±665	3.49±0.12	3.74±0.20
D65R	3104±332	3.50±0.08	3.70±0.18
R52A	3150±273	3.47±0.08	3.70±0.15
S99R	3691±593	3.48±0.11	3.64±0.17
R52E	3030±314	3.57±0.09	3.85±0.15
R52Y	3290±592	3.53±0.10	3.82±0.17
F62L	3232±470	3.15±0.09	3.51±0.22
P68K	3385±503	3.41±0.10	3.67±0.20
S99K	3441±605	3.49±0.11	3.71±0.17
R52D	2473±244	3.58±0.09	3.93±0.15
R52K	3196±509	3.50±0.11	3.82±0.16
P97T T98G	1503±62	2.63±0.12	3.79±0.28
P68R	3383±435	3.38±0.10	3.68±0.19
V107I	3169±524	3.33±0.11	3.64±0.19
P68T	3478±532	3.39±0.12	3.67±0.20
R52F	2894±305	3.57±0.10	3.90±0.16
FAST (original)	3589±437	3.64±0.11	3.90±0.14

Table S5. Brightness and fluorescence lifetime data of FAST variants with **25DOM-HBI-2T** fluorogen. τ_{φ} – average phase lifetime ± SD, τ_m – average modulation lifetime ± SD.

**FAST variant**	**Brightness, r.u.**	**τ_{φ}, ns**	**τ_m, ns**
R52L	2214±62	2.45±0.08	3.35±0.33
P73S	2317±140	2.36±0.08	3.14±0.35
R52E D65R	1521±54	2.89±0.10	3.84±0.24
S99E	3074±91	2.36±0.07	2.99±0.37
P97T	1134±48	-[a]	-[a]
D65K	1974±84	2.49±0.09	3.35±0.35
D65R	1496±60	2.86±0.12	3.75±0.29
R52A	2748±119	2.38±0.07	3.04±0.34
S99R	2506±109	2.49±0.07	3.18±0.35
R52E	2249±83	2.64±0.07	3.37±0.30
R52Y	1755±51	2.67±0.08	3.63±0.26
**F62L**	1568±113	2.74±0.12	3.80±0.30
**P68K**	2705±74	2.37±0.07	3.15±0.36
S99K	2600±83	2.40±0.08	3.22±0.35
R52D	1289±40	3.13±0.12	4.23±0.19
**R52K**	2989±120	2.47±0.07	3.29±0.35
P97T T98G	959±35	-[a]	-[a]
P68R	2443±152	2.29±0.08	3.20±0.40
V107I	1599±68	2.56±0.10	3.69±0.30
**P68T**	2361±110	2.19±0.10	3.24±0.41
R52F	1530±90	2.64±0.13	3.81±0.31
FAST (original)	2735±170	2.70±0.09	3.41±0.31

a - Low brightness, fluorescence lifetime cannot be determined accurately.

Table S6. Fluorescence lifetimes (τ) of **HBR-2,5-DM**, **HBR-DOM2**, **HMBR** and **25DOM-HBI-2T** fluorogens in complexes with FAST variants. τ is the fluorescence lifetime of the corresponding exponential component; A is a relative contribution of the exponential decay component; χ^2 is a value of the Pearson's chi-squared test. Unless otherwise mentioned, deconvolution with the Instrument Response Function (IRF) was used to fit the data.

Fluorogen	FAST variant	τ_1 ± SD, ns	A_1, %	τ_2 ± SD, ns	A_2, %	χ^2
**HBR-2,5-DM**	FAST (original)	2.479 ± 0.004	95	0.131 ± 0.007	5	1.09
	R52K	1.429 ± 0.004	89	0.152 ± 0.009	11	1.25
	F62L	2.331 ± 0.005	94	0.158 ± 0.011	6	1.18
	P68K	2.002 ± 0.005	92	0.192 ± 0.012	8	1.13
	P68T	1.752 ± 0.005	91	0.168 ± 0.010	9	1.17
**HBR-DOM2**	FAST (original)	3.742 ± 0.007	98	0.429 ± 0.030	2	1.14
	R52K	3.545 ± 0.007	98	0.219 ± 0.023	2	1.12
	F62L	2.878 ± 0.008	97	0.460 ± 0.042	3	1.15
	P68K	3.528 ± 0.006	100			1.09*
	P68T	3.300 ± 0.006	100			1.29
**HMBR**	FAST (original)	2.190 ± 0.004	90	0.206 ± 0.007	10	1.213
	R52K	1.362 ± 0.005	89	0.214 ± 0.012	11	1.11
	F62L	2.051 ± 0.005	93	0.182 ± 0.012	7	1.17
	P68K	1.743 ± 0.008	83	0.421 ± 0.014	17	1.16
	P68T	1.546 ± 0.006	89	0.280 ± 0.015	11	1.14
**25DOM-HBI-2T**	FAST (original)	2.915 ± 0.078	47**	1.701 ± 0.067	44**	1.241
	R52K	2.320 ± 0.007	88	0.284 ± 0.010	12	1.27
	F62L	1.984 ± 0.006	84	0.187 ± 0.006	16	1.30
	P68K	1.961 ± 0.006	88	0.210 ± 0.009	12	1.29
	P68T	1.721 ± 0.005	85	0.185 ± 0.007	15	1.24

* Tail fit.

** Additional third component with τ_3 = 0.145±0.007 ns (9%) was found.

Table S7. Dissociation constants values of complexes [protein-fluorogen]

Fluorogen	Structure	FAST variant	K_d, µM
**25DOM-HBI-2T**	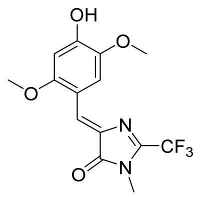	**R52K**	0.44±0.05
		**F62L**	0.97±0.10
		**P68K**	0.34±0.01
		**P68T**	0.52±0.05
**HMBR**	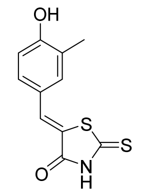	**R52K**	0.13±0.01
		**F62L**	0.25±0.01
		**P68K**	0.16±0.01
		**P68T**	0.19±0.02
**HBR-2,5-DM**	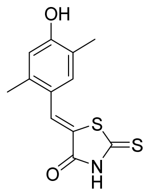	**R52K**	0.046±0.004
		**F62L**	0.12±0.02
		**P68K**	0.084±0.007
		**P68T**	0.090±0.007
**HBR-DOM2**	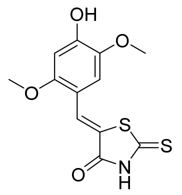	**R52K**	0.035±0.001
		**F62L**	0.036±0.001
		**P68K**	0.030±0.001
		**P68T**	0.036±0.001

Table S8. Optical properties of proposed fluorogens in complexes with FAST variants.

Fluorogen	FAST varian	K_d, µM [a]	ε, $M^{-1}·cm^{-1}$ [b]	FQY, % [c]	Brightness	Abs, nm[d]	Em, nm[d]
25DOM-HBI-2T	FAST[1]	0.52	92000	21	19300	523	539
	R52K	0.44±0.05	70500±1060	29±1.8	20350±1550	524	540
	F62L	0.97±0.10	69000±1040	20±0.7	13450±710	522	539
	P68K	0.34±0.01	69500±1040	26±1.5	18200±1280	524	540
	P68T	0.52±0.05	68000±1020	20±0.4	13350±480	524	540
HMBR	FAST[2]	0.13±0.02	45000±680	31±0.6	13900±480	483	540
	R52K	0.13±0.01	40000±600	22±0.7	8850±420	479	542
	F62L	0.25±0.01	39500±590	33±1.7	12850±870	481	544
	P68K	0.16±0.01	39500±590	30±1.7	11950±850	482	542
	P68T	0.19±0.02	41500±620	34±1.3	14200±750	478	539
HBR-2,5-DM	FAST[3]	0.008	50000	29	14500	494	552
	R52K	0.046±0.004	37500±560	25±0.6	9350±380	495	550
	F62L	0.12±0.02	40500±610	43±0.9	17250±630	498	549
	P68K	0.084±0.007	38500±580	34±0.7	13150±450	497	551
	P68T	0.090±0.007	38500±580	31±0.9	11800±520	494	549
HBR-DOM2	FAST[4]	0.021±0.001	30500±460	54±3.4	16500±1300	510	566
	R52K	0.035±0.001	35500±530	50±1.4	17700±740	524	567
	F62L	0.036±0.001	31000±470	46±1.7	14400±750	502	565
	P68K	0.030±0.001	30500±460	47±2.5	14400±980	505	568
	P68T	0.036±0.001	30000±450	52±4.0	15600±1420	504	565

a – represented as mean ± SD (n = 3);
b – represented as result of single measurement ± the precision of the measuring instruments (weighing and pipetting errors);
c – fluorescence quantum yield, represented as mean ± SD (n = 9);
d – maxima position.

Table S9. Fluorescence lifetimes of **HBR-2,5-DM**, **HBR-DOM2**, **HMBR** and **25DOM-HBI-2T** fluorogens in complexes with FAST variants expressed in Hela Kyoto cells as an H2B fusion. $\tau_{1/2}$ is the fluorescence lifetime of the corresponding exponential component in biexponential fitting; A is a relative contribution of the exponential decay component in biexponential fitting; τ_m is an amplitude-weighted average lifetime in biexponential fitting; τ_i is an intensity-weighted average lifetime in biexponential fitting; χ^2 is a characteristic value of the Pearson's chi-squared test; τ is a fluorescence lifetime in monoexponential fitting; the number of individually analyzed nuclei is 16. SD is standard deviation.

		Biexponential fit							Monoexponential fit	
Fluorogen	FAST variant	τ_1 ± SD, ns	A_1± SD, %	τ_2 ± SD, ns	A_2± SD, %	τ_m± SD	τ_i± SD	χ^{2*}	τ± SD	χ^2
**HBR-2,5-DM**	H2B-R52K	1.257±0.043	66.6±8.3	1.641±0.106	33.4±8.3	1.375±0.0118	1.401±0.013	<1.2	1.386±0.010	<1.2
	H2B-F62L	1.368±0.279	20.2±6.3	2.357±0.033	79.8±6.3	2.170±0.028	2.239±0.012	<1.2	2.200±0.009	<1.2
	H2B-P68K	1.286±0.172	33.9±11	2.197±0.093	66.1±11	1.894±0.036	1.987±0.028	<1.2	1.929±0.021	<1.2
	H2B-P68T	1.475±0.037	81.9±5.3	2.577±0.194	18.1±5.3	1.667±0.022	1.769±0.027	<1.2	1.689±0.022	<1.2
**HBR-DOM2**	H2B-R52K	3.312±0.279	49.6±5.9	3.972±0.267	50.4±5.9	3.652±0.052	3.698±0.112	>1.2	3.623±0.048	<1.2
	H2B-F62L	2.806±0.234	54±9.1	3.534±0.391	46±9.1	3.103±0.070	3.165±0.078	>1.2	3.074±0.070	<1.2
	H2B-P68K	3.160±0.345	48.9±6.4	3.915±0.339	51.1±6.4	3.545±0.074	3.612±0.125	>1.2	3.513±0.077	<1.2
	H2B-P68T	3.280±0.170	51.2±5.7	3.698±0.172	48.8±5.7	3.480±0.055	3.499±0.058	<1.2	3.464±0.055	<1.2
**HMBR**	H2B-R52K	0.699 ± 0.076	42.7±7.8	1.472 ± 0.057	57.3±7.8	1.143 ± 0.012	1.268±0.009	<1.2	1.243±0.010	>1.2
	H2B-F62L	0.558 ± 0.161	22.0±3.4	2.036 ± 0.031	77.6±3.4	1.706 ± 0.051	1.930±0.015	<1.2	1.896±0.016	>1.2
	H2B-P68K	0.799 ± 0.048	48.9±4.6	1.836 ± 0.047	51.1±4.6	1.328 ± 0.012	1.529±0.007	<1.2	1.473±0.009	>1.2
	H2B-P68T	0.679 ± 0.077	31.9±6.6	1.568 ± 0.047	68.1±6.6	1.286 ± 0.027	1.417±0.018	<1.2	1.397±0.016	>1.2
**25DOM-HBI-2T**	H2B-R52K	1.212 ± 0.057	54.0±2.5	2.669 ± 0.052	46.0±2.5	1.882 ± 0.024	2.16±0.021	<1.2	1.974±0.019	>1.2
	H2B-F62L	0.690 ± 0.089	43.9±5.1	2.056 ± 0.093	56.1±5.1	1.456 ± 0.033	1.769±0.025	<1.2	1.655±0.017	>2.0
	H2B-P68K	1.283 ± 0.105	58.2±8.7	2.360 ± 0.137	41.2±8.7	1.715 ± 0.041	1.885±0.024	<1.2	1.782±0.017	>1.2
	H2B-P68T	0.958 ± 0.057	55.6±5.9	1.993 ± 0.063	44.4	1.417 ± 0.025	1.601±0.017	<1.2	1.514±0.020	>1.2

* For exact values for individual measurements, see the tables below.

Table S10. Monoexponential fitting of fluorescence decay data without binning (binning value was set to 0 when fitting in Becker & Hickl SPCImage software) of **HBR-2,5-DM** fluorogen in complexes with FAST variants expressed in Hela Kyoto cells as an H2B fusion. τ is the fluorescence lifetime; χ^2 is a value of the Pearson's chi-squared test. Each value corresponds to the individual analyzed nucleus.

R52K		F62L		P68K		P68T	
τ, ns	χ^{2*}	τ, ns	χ^{2*}	τ, ns	χ^{2*}	τ, ns	χ^{2*}
1.36	1.14	2.16	0.93	1.977	1.14	1.623	1.16
1.329	0.92	2.203	1.09	1.929	0.91	1.625	1.13
1.341	1.13	2.155	1.02	1.904	1.03	1.559	1.06
1.344	0.97	2.179	1.08	1.945	0.94	1.51	0.82
1.311	1.14	2.194	1	1.87	0.99	1.5	1.04
1.339	1.18	2.175	1.1	1.867	0.98	1.501	1.06
1.33	1.08	2.205	1.07	1.9	0.99	1.539	1.14
1.327	1.02	2.15	0.97	1.913	1.07	1.535	1.08
1.31	1.17	2.202	1.02	1.941	0.95	1.516	1.16
1.319	1.04	2.195	0.97	1.915	1.13	1.612	0.9
1.346	1.08	2.191	0.96	1.927	0.94	1.576	1.16
1.318	1	2.154	1.03	1.935	1.1	1.627	1.04
1.348	1.19	2.206	0.86	1.929	0.92	1.573	1.16
1.335	0.88	2.171	0.88	1.825	1.1	1.59	0.97
1.321	1.07	2.193	0.84	1.896	1.04	1.573	0.93
1.36	0.9	2.16	1	1.977	1.06	1.623	1.13
1.329	1.11	2.203	0.96	1.929	1.07	1.625	0.97
1.341	0.89	2.155	1.18	1.904	0.99	1.559	0.83

*Pearson's test value <0.95 can indicate 'overfitting' due to low photon count.

Table S11. Comparison of bi- and monoexponential fitting of the same fluorescence decay data of **HBR-2,5-DM** fluorogen in complexes with R52K FAST variant expressed in Hela Kyoto cells as an H2B fusion. $\tau_{1/2}$ is the fluorescence lifetime of the corresponding exponential component in biexponential fitting; A is a relative contribution of the exponential decay component in biexponential fitting; τ_m is an amplitude-weighted average lifetime in biexponential fitting; τ_i is an intensity-weighted average lifetime in biexponential fitting; χ^2 is a value of the Pearson's chi-squared test; τ is a fluorescence lifetime in monoexponential fitting. Each row corresponds to the individual analyzed nucleus.

R52K								
Biexponential fitting							Monoexponential fitting	
τ_1, ns	A_1, %	τ_2, ns	A_2, %	τ_m, ns	τ_i, ns	χ^2	τ, ns	χ^2
1.238	71	1.663	29	1.361	1.389	1.1	1.371	1.12
1.228	67	1.637	33	1.363	1.390	1.11	1.376	1.11
1.313	60	1.512	40	1.393	1.399	1.07	1.395	1.06
1.239	76	1.802	24	1.374	1.416	1.08	1.39	1.13
1.216	65	1.704	35	1.387	1.426	1.08	1.402	1.12
1.244	68	1.599	32	1.358	1.378	1.08	1.367	1.09
1.219	74	1.784	26	1.366	1.411	1.05	1.386	1.11
1.385	42	1.393	58	1.390	1.390	1.1	1.39	1.09
1.266	65	1.626	35	1.392	1.413	1.13	1.402	1.13
1.269	68	1.614	32	1.379	1.398	1.12	1.387	1.13
1.224	70	1.73	30	1.376	1.415	1.06	1.391	1.11
1.267	62	1.566	38	1.381	1.396	1.09	1.388	1.1
1.245	70	1.69	30	1.379	1.409	1.02	1.388	1.05
1.26	65	1.586	35	1.374	1.392	1.05	1.38	1.06
1.249	76	1.714	24	1.361	1.390	1.14	1.374	1.17
1.083	54	1.601	46	1.320	1.370	1.06	1.366	1.05

Table S12. Comparison of bi- and monoexponential fitting of the same fluorescence decay data of **HBR-2,5-DM** fluorogen in complexes with F62L FAST variant expressed in Hela Kyoto cells as an H2B fusion. $\tau_{1/2}$ is the fluorescence lifetime of the corresponding exponential component in biexponential fitting; A is a relative contribution of the exponential decay component in biexponential fitting; τ_m is an amplitude-weighted average lifetime in biexponential fitting; τ_i is an intensity-weighted average lifetime in biexponential fitting; χ^2 is a value of the Pearson's chi-squared test; τ is a fluorescence lifetime in monoexponential fitting. Each row corresponds to the individual analyzed nucleus.

F62L								
Biexponential fitting							Monoexponential fitting	
τ_1, ns	A_1, %	τ_2, ns	A_2, %	τ_m, ns	τ_i, ns	χ^2	τ, ns	χ^2
1.331	18	2.352	82	2.168	2.239	1.18	2.197	1.16
1.406	22	2.379	78	2.165	2.240	1.07	2.194	1.09
1.16	14	2.34	86	2.175	2.252	1.18	2.206	1.12
1.242	18	2.359	82	2.158	2.243	1.13	2.201	1.12
1.592	23	2.353	77	2.178	2.225	1.09	2.196	1.08
1.509	23	2.4	77	2.195	2.259	1.13	2.219	1.12
1.822	35	2.394	65	2.194	2.228	1.03	2.21	1.03
0.712	10	2.272	90	2.116	2.220	1.15	2.184	1.18
1.235	13	2.323	87	2.182	2.243	1.16	2.195	1.08
1.316	14	2.346	86	2.202	2.260	1.18	2.212	1.09
1.539	25	2.39	75	2.177	2.240	1.09	2.2	1.1
0.979	17	2.328	83	2.099	2.221	1.12	2.189	1.22
1.482	22	2.37	78	2.175	2.237	1.07	2.193	1.07
1.608	23	2.371	77	2.196	2.242	1.04	2.208	1.03
1.585	26	2.381	74	2.174	2.230	1.11	2.199	1.13
1.862	47	2.449	53	2.172	2.212	1.15	2.191	1.17

Table S13. Comparison of bi- and monoexponential fitting of the same fluorescence decay data of **HBR-2,5-DM** fluorogen in complexes with P68K FAST variant expressed in Hela Kyoto cells as an H2B fusion. $\tau_{1/2}$ is the fluorescence lifetime of the corresponding exponential component in biexponential fitting; A is a relative contribution of the exponential decay component in biexponential fitting; τ_m is an amplitude-weighted average lifetime in biexponential fitting; τ_i is an intensity-weighted average lifetime in biexponential fitting; χ^2 is a value of the Pearson's chi-squared test; τ is a fluorescence lifetime in monoexponential fitting. Each row corresponds to the individual analyzed nucleus.

P68K								
Biexponential fitting							Monoexponential fitting	
τ_1, ns	A_1, %	τ_2, ns	A_2, %	τ_m, ns	τ_i, ns	χ^2	τ, ns	χ^2
1.375	38	2.214	62	1.895	1.983	1.16	1.926	1.12
1.245	34	2.202	66	1.877	1.986	1.17	1.918	1.18
1.307	35	2.254	65	1.923	2.029	1.12	1.958	1.16
1.384	41	2.304	59	1.927	2.033	1.16	1.954	1.18
1.465	39	2.246	61	1.941	2.016	1.17	1.962	1.18
1.549	54	2.37	46	1.927	2.014	1.1	1.945	1.15
1.22	13	2.04	87	1.933	1.973	1.26	1.934	1.19
1.235	36	2.176	64	1.837	1.948	1.02	1.888	1.19
1.469	43	2.205	57	1.889	1.959	1.05	1.911	1.1
1.396	38	2.206	62	1.898	1.980	1.09	1.932	1.16
1.35	33	2.208	67	1.925	2.009	1.05	1.952	1.08
0.946	11	2.011	89	1.894	1.952	1.22	1.915	1.13
1.192	33	2.183	67	1.856	1.973	1.25	1.914	1.13
1.195	37	2.24	63	1.853	1.991	1.01	1.913	1.14
0.966	23	2.094	77	1.835	1.957	1.07	1.912	1.22
1.506	51	2.280	49	1.888	1.968	1.05	1.930	1.11

Table S14. Comparison of bi- and monoexponential fitting of the same fluorescence decay data of **HBR-2,5-DM** fluorogen in complexes with P68T FAST variant expressed in Hela Kyoto cells as an H2B fusion. $\tau_{1/2}$ is the fluorescence lifetime of the corresponding exponential component in biexponential fitting; A is a relative contribution of the exponential decay component in biexponential fitting; τ_m is an amplitude-weighted average lifetime in biexponential fitting; τ_i is an intensity-weighted average lifetime in biexponential fitting; χ^2 is a value of the Pearson's chi-squared test; τ is a fluorescence lifetime in monoexponential fitting. Each row corresponds to the individual analyzed nucleus.

P68T								
Biexponential fitting							Monoexponential fitting	
τ_1, ns	A_1, %	τ_2, ns	A_2, %	τ_m, ns	τ_i, ns	χ^2	τ, ns	χ^2
1.489	74	2.341	26	1.711	1.792	1.07	1.726	1.15
1.448	79	2.493	21	1.667	1.776	1.05	1.689	1.19
1.517	86	2.724	14	1.686	1.790	1.01	1.717	1.09
1.532	90	2.93	10	1.672	1.777	1.13	1.707	1.16
1.459	77	2.396	23	1.675	1.767	1.03	1.691	1.11
1.455	83	2.711	17	1.669	1.802	1.02	1.684	1.16
1.54	90	2.757	10	1.662	1.742	1.17	1.696	1.16
1.494	86	2.74	14	1.668	1.780	1.02	1.698	1.12
1.444	83	2.768	17	1.669	1.817	1.03	1.696	1.18
1.418	80	2.535	20	1.641	1.763	1	1.668	1.15
1.487	89	2.716	11	1.622	1.713	1.01	1.65	1.02
1.451	78	2.389	22	1.657	1.748	1.11	1.672	1.18
1.425	80	2.466	20	1.633	1.739	1.01	1.648	1.12
1.484	76	2.327	24	1.686	1.763	1.03	1.702	1.1
1.483	77	2.367	23	1.686	1.768	1.02	1.698	1.09
1.108	53	2.047	47	1.550	1.691	1.13	1.648	1.05

Table S15. Comparison of bi- and monoexponential fitting of the same fluorescence decay data of **HBR-DOM2** fluorogen in complexes with R52K FAST variant expressed in Hela Kyoto cells as an H2B fusion. $\tau_{1/2}$ is the fluorescence lifetime of the corresponding exponential component in biexponential fitting; A is a relative contribution of the exponential decay component in biexponential fitting; τ_m is an amplitude-weighted average lifetime in biexponential fitting; τ_i is an intensity-weighted average lifetime in biexponential fitting; χ^2 is a value of the Pearson's chi-squared test; τ is a fluorescence lifetime in monoexponential fitting. Each row corresponds to the individual analyzed nucleus.

R52K								
Biexponential fitting							Monoexponential fitting	
τ_1, ns	A_1, %	τ_2, ns	A_2, %	τ_m, ns	τ_i, ns	χ^2	τ, ns	χ^2
3.386	44	3.982	56	3.720	3.743	1.29	3.684	1.15
3.376	44	3.765	56	3.594	3.604	1.22	3.583	1.19
3.483	45	3.724	55	3.616	3.620	1.25	3.561	1.07
3.077	47	4.103	53	3.621	3.693	1.18	3.597	1.18
3.323	56	3.875	44	3.566	3.587	1.12	3.535	1.04
3.067	47	4.153	53	3.643	3.723	1.11	3.612	1.12
3.564	56	3.758	44	3.649	3.652	1.14	3.646	1.13
3.458	56	3.933	44	3.667	3.682	1.17	3.65	1.13
2.549	41	4.571	59	3.742	4.006	1.88	3.666	1.19
3.013	52	4.513	48	3.733	3.883	1.45	3.683	1.2
3.392	45	3.751	55	3.589	3.598	1.19	3.56	1.12
3.486	56	3.855	44	3.648	3.658	1.16	3.636	1.13
3.413	56	3.958	44	3.653	3.673	1.23	3.627	1.14
3.649	43	3.701	57	3.679	3.679	1.2	3.678	1.19
3.438	56	3.936	44	3.657	3.674	1.17	3.634	1.11
3.650	56	3.671	44	3.659	3.659	1.16	3.658	1.16

Table S16. Comparison of bi- and monoexponential fitting of the same fluorescence decay data of **HBR-DOM2** fluorogen in complexes with F62L FAST variant expressed in Hela Kyoto cells as an H2B fusion. $\tau_{1/2}$ is the fluorescence lifetime of the corresponding exponential component in biexponential fitting; A is a relative contribution of the exponential decay component in biexponential fitting; τ_m is an amplitude-weighted average lifetime in biexponential fitting; τ_i is an intensity-weighted average lifetime in biexponential fitting; χ^2 is a value of the Pearson's chi-squared test; τ is a fluorescence lifetime in monoexponential fitting. Each row corresponds to the individual analyzed nucleus.

F62L								
Biexponential fitting							Monoexponential fitting	
τ_1, ns	A_1, %	τ_2, ns	A_2, %	τ_m, ns	τ_i, ns	χ^2	τ, ns	χ^2
2.613	68	4.12	32	3.095	3.255	1.3	3.064	1.06
2.512	66	4.288	34	3.116	3.343	1.59	3.057	1.14
2.925	54	3.37	46	3.130	3.145	1.2	3.107	1.11
3.051	47	3.25	53	3.156	3.160	1.45	3.11	1.21
2.973	45	3.373	55	3.193	3.205	1.43	3.155	1.17
2.993	55	3.445	45	3.196	3.212	1.26	3.174	1.18
2.971	55	3.301	45	3.120	3.128	1.19	3.107	1.14
2.964	46	3.352	54	3.174	3.185	1.27	3.156	1.19
2.854	46	3.354	54	3.124	3.144	1.37	3.091	1.2
2.752	46	3.215	54	3.002	3.020	1.33	2.959	1.09
2.939	54	3.202	46	3.060	3.066	1.24	3.053	1.22
2.847	45	3.358	55	3.128	3.149	1.32	3.091	1.11
2.915	56	3.28	44	3.076	3.086	1.22	3.061	1.16
2.205	53	3.798	47	2.954	3.168	1.15	2.93	1.15
2.57	74	4.304	26	3.021	3.212	1.25	2.992	1.12
3.105	55	3.111	45	3.107	3.107	1.15	3.107	1.15

Table S17. Comparison of bi- and monoexponential fitting of the same fluorescence decay data of **HBR-DOM2** fluorogen in complexes with P68K FAST variant expressed in Hela Kyoto cells as an H2B fusion. $\tau_{1/2}$ is the fluorescence lifetime of the corresponding exponential component in biexponential fitting; A is a relative contribution of the exponential decay component in biexponential fitting; τ_m is an amplitude-weighted average lifetime in biexponential fitting; τ_i is an intensity-weighted average lifetime in biexponential fitting; χ^2 is a value of the Pearson's chi-squared test; τ is a fluorescence lifetime in monoexponential fitting. Each row corresponds to the individual analyzed nucleus.

P68K								
Biexponential fitting							Monoexponential fitting	
τ_1, ns	A_1, %	τ_2, ns	A_2, %	τ_m, ns	τ_i, ns	χ^2	τ, ns	χ^2
3.319	44	3.806	56	3.592	3.608	1.17	3.564	1.1
3.252	44	3.889	56	3.609	3.636	1.32	3.572	1.2
2.716	43	3.829	57	3.350	3.441	1.05	3.334	1.06
2.918	60	4.524	40	3.560	3.734	1.4	3.5	1.1
3.354	56	3.813	44	3.556	3.571	1.11	3.534	1.05
3.438	56	3.639	44	3.526	3.529	1.01	3.522	1
3.214	45	3.613	55	3.433	3.445	1.24	3.417	1.2
3.256	44	3.819	56	3.571	3.593	1.14	3.563	1.14
3.441	56	3.749	44	3.577	3.583	1.11	3.57	1.09
3.202	45	3.872	55	3.571	3.602	1.27	3.535	1.14
2.469	42	4.405	58	3.592	3.846	2.22	3.512	1.19
2.499	53	4.685	47	3.526	3.864	2.34	3.402	1.16
3.333	44	3.586	56	3.475	3.479	1.14	3.468	1.13
3.522	45	3.676	55	3.607	3.608	1.24	3.583	1.17
3.468	56	3.827	44	3.626	3.635	1.2	3.614	1.17
3.145	44	3.747	56	3.484	3.509	1.06	3.477	1.06

Table S18. Comparison of bi- and monoexponential fitting of the same fluorescence decay data of **HBR-DOM2** fluorogen in complexes with P68T FAST variant expressed in Hela Kyoto cells as an H2B fusion. $\tau_{1/2}$ is the fluorescence lifetime of the corresponding exponential component in biexponential fitting; A is a relative contribution of the exponential decay component in biexponential fitting; τ_m is an amplitude-weighted average lifetime in biexponential fitting; τ_i is an intensity-weighted average lifetime in biexponential fitting; χ^2 is a value of the Pearson's chi-squared test; τ is a fluorescence lifetime in monoexponential fitting. Each row corresponds to the individual analyzed nucleus.

P68T								
Biexponential fitting							Monoexponential fitting	
τ_1, ns	A_1, %	τ_2, ns	A_2, %	τ_m, ns	τ_i, ns	χ^2	τ, ns	χ^2
3.229	45	3.733	55	3.506	3.524	1.21	3.477	1.12
3.53	43	3.554	57	3.544	3.544	1.11	3.543	1.1
3.41	56	3.596	44	3.492	3.494	1.06	3.488	1.05
3.21	45	3.742	55	3.503	3.523	1.25	3.478	1.17
3.147	45	3.743	55	3.475	3.500	1.12	3.443	1
3.257	56	3.716	44	3.459	3.474	1.22	3.44	1.16
2.919	57	4.214	43	3.476	3.594	1.2	3.44	1.07
3.346	56	3.753	44	3.525	3.537	1.25	3.509	1.2
3.377	55	3.781	45	3.559	3.570	1.21	3.541	1.16
2.992	46	3.594	54	3.317	3.344	1.1	3.31	1.1
3.253	55	3.706	45	3.457	3.472	1.23	3.437	1.18
3.459	56	3.479	44	3.468	3.468	1.16	3.467	1.15
3.284	55	3.65	45	3.449	3.458	1.07	3.434	1.04
3.471	43	3.494	57	3.484	3.484	1.03	3.484	1.03
3.313	55	3.711	45	3.492	3.503	1.13	3.476	1.09
3.384	44	3.438	56	3.414	3.414	1.08	3.413	1.08

Table S19. Comparison of bi- and monoexponential fitting of the same fluorescence decay data of **HMBR** fluorogen in complexes with R52K FAST variant expressed in Hela Kyoto cells as an H2B fusion. $\tau_{1/2}$ is the fluorescence lifetime of the corresponding exponential component in biexponential fitting; A is a relative contribution of the exponential decay component in biexponential fitting; τ_m is an amplitude-weighted average lifetime in biexponential fitting; τ_i is an intensity-weighted average lifetime in biexponential fitting; χ^2 is a value of the Pearson's chi-squared test; τ is a fluorescence lifetime in monoexponential fitting. Each row corresponds to the individual analyzed nucleus.

R52K								
Biexponential fitting							Monoexponential fitting	
τ_1, ns	A_1, %	τ_2, ns	A_2, %	τ_m, ns	τ_i, ns	χ^2	τ, ns	χ^2
0.825	56	1.568	44	1.149	1.267	1.08	1.234	2.27
0.717	43	1.477	57	1.153	1.275	1.18	1.247	1.93
0.675	40	1.424	60	1.124	1.244	1.01	1.221	1.76
0.673	38	1.44	62	1.150	1.270	1.12	1.243	1.59
0.545	29	1.396	71	1.145	1.277	1.06	1.258	1.73
0.672	42	1.457	58	1.127	1.260	1.07	1.235	2.1
0.811	57	1.586	43	1.142	1.271	1.1	1.237	2.35
0.728	41	1.459	59	1.156	1.268	1	1.245	1.72
0.683	41	1.445	59	1.133	1.257	1.09	1.232	2.01
0.83	56	1.584	44	1.159	1.280	1.19	1.248	2.13
0.723	43	1.472	57	1.152	1.271	1.14	1.248	2.1
0.671	43	1.469	57	1.125	1.264	1.04	1.238	2.25
0.723	43	1.47	57	1.150	1.269	1.11	1.247	2.12
0.65	34	1.425	66	1.159	1.276	1.04	1.258	1.75
0.618	38	1.444	62	1.132	1.273	1.07	1.254	2.64
0.64	38	1.441	62	1.139	1.271	1	1.249	2.17

Table S20. Comparison of bi- and monoexponential fitting of the same fluorescence decay data of **HMBR** fluorogen in complexes with F62L FAST variant expressed in Hela Kyoto cells as an H2B fusion. $\tau_{1/2}$ is the fluorescence lifetime of the corresponding exponential component in biexponential fitting; A is a relative contribution of the exponential decay component in biexponential fitting; τ_m is an amplitude-weighted average lifetime in biexponential fitting; τ_i is an intensity-weighted average lifetime in biexponential fitting; χ^2 is a value of the Pearson's chi-squared test; τ is a fluorescence lifetime in monoexponential fitting. Each row corresponds to the individual analyzed nucleus.

F62L								
Biexponential fitting							Monoexponential fitting	
τ_1, ns	A_1, %	τ_2, ns	A_2, %	τ_m, ns	τ_i, ns	χ^2	τ, ns	χ^2
0.633	28	2.061	72	1.667	1.911	1.05	1.865	1.76
0.585	21	2.012	79	1.718	1.912	1.13	1.879	1.62
0.356	25	2.039	75	1.621	1.947	1.14	1.912	1.82
0.383	25	2.044	75	1.621	1.944	1	1.908	1.79
1.001	28	2.118	72	1.810	1.948	1.12	1.898	1.36
0.399	22	2.021	78	1.664	1.935	1.13	1.904	1.73
0.562	25	2.063	75	1.692	1.940	1.17	1.908	1.78
0.645	27	2.042	73	1.671	1.899	1.1	1.859	2.11
0.451	19	1.991	81	1.700	1.914	1.06	1.889	1.45
0.551	20	2.026	80	1.735	1.934	1.2	1.905	1.65
0.794	23	2.072	77	1.775	1.939	1.19	1.892	1.61
0.466	20	2.01	80	1.700	1.925	1.2	1.898	1.67
0.53	20	2.017	80	1.715	1.924	1.08	1.897	1.51
0.53	17	2.001	83	1.751	1.925	1.19	1.902	1.42
0.532	19	2.027	81	1.738	1.939	1.09	1.91	1.42
0.509	20	2.027	80	1.725	1.938	1.19	1.911	1.55

Table S21. Comparison of bi- and monoexponential fitting of the same fluorescence decay data of **HMBR** fluorogen in complexes with P68K FAST variant expressed in Hela Kyoto cells as an H2B fusion. $\tau_{1/2}$ is the fluorescence lifetime of the corresponding exponential component in biexponential fitting; A is a relative contribution of the exponential decay component in biexponential fitting; τ_m is an amplitude-weighted average lifetime in biexponential fitting; τ_i is an intensity-weighted average lifetime in biexponential fitting; χ^2 is a value of the Pearson's chi-squared test; τ is a fluorescence lifetime in monoexponential fitting. Each row corresponds to the individual analyzed nucleus.

P68K								
Biexponential fitting							Monoexponential fitting	
τ_1, ns	A_1, %	τ_2, ns	A_2, %	τ_m, ns	τ_i, ns	χ^2	τ, ns	χ^2
0.889	54	1.91	46	1.355	1.546	1.05	1.484	2.27
0.866	54	1.894	46	1.338	1.534	1.04	1.47	2.31
0.851	54	1.878	46	1.319	1.517	1.19	1.457	3.14
0.762	46	1.8	54	1.319	1.522	1.15	1.472	2.6
0.78	47	1.804	53	1.326	1.522	1.02	1.469	1.97
0.792	46	1.814	54	1.340	1.534	1.07	1.481	2.15
0.799	49	1.838	51	1.324	1.528	1.11	1.47	2.6
0.8	48	1.824	52	1.335	1.531	1.07	1.474	2.26
0.697	39	1.749	61	1.335	1.533	1.12	1.491	1.93
0.737	41	1.756	59	1.338	1.526	1.1	1.48	1.99
0.835	54	1.894	46	1.317	1.529	1.08	1.464	3.46
0.793	50	1.85	50	1.326	1.537	1.12	1.477	2.67
0.769	46	1.808	54	1.325	1.528	1.07	1.473	2.52
0.797	52	1.85	48	1.306	1.518	1.11	1.46	3.77
0.817	53	1.876	47	1.318	1.530	1.04	1.467	3.74
0.796	48	1.825	52	1.329	1.528	1.1	1.473	2.68

Table S22. Comparison of bi- and monoexponential fitting of the same fluorescence decay data of **HMBR** fluorogen in complexes with P68T FAST variant expressed in Hela Kyoto cells as an H2B fusion. $\tau_{1/2}$ is the fluorescence lifetime of the corresponding exponential component in biexponential fitting; A is a relative contribution of the exponential decay component in biexponential fitting; τ_m is an amplitude-weighted average lifetime in biexponential fitting; τ_i is an intensity-weighted average lifetime in biexponential fitting; χ^2 is a value of the Pearson's chi-squared test; τ is a fluorescence lifetime in monoexponential fitting. Each row corresponds to the individual analyzed nucleus.

P68T								
Biexponential fitting							Monoexponential fitting	
τ_1, ns	A_1, %	τ_2, ns	A_2, %	τ_m, ns	τ_i, ns	χ^2	τ, ns	χ^2
0.598	28	1.533	72	1.275	1.412	1.05	1.39	1.65
0.601	23	1.516	77	1.306	1.420	1.02	1.4	1.42
0.621	26	1.544	74	1.301	1.428	1.01	1.405	1.42
0.716	36	1.582	64	1.267	1.404	1.16	1.374	1.93
0.54	27	1.531	73	1.266	1.418	1.17	1.397	1.72
0.677	27	1.533	73	1.298	1.411	1.17	1.388	1.58
0.678	31	1.512	69	1.252	1.371	1.07	1.403	1.45
0.738	35	1.587	65	1.292	1.419	1.08	1.394	1.57
0.659	37	1.577	63	1.237	1.396	1.06	1.368	1.74
0.621	31	1.553	69	1.264	1.411	1.08	1.391	1.78
0.624	28	1.536	72	1.285	1.414	1.04	1.395	1.43
0.732	30	1.576	70	1.327	1.438	1.06	1.417	1.5
0.683	30	1.579	70	1.312	1.440	1.09	1.42	1.6
0.759	31	1.595	69	1.336	1.448	1.09	1.428	1.66
0.791	43	1.657	57	1.287	1.430	1.14	1.397	1.66
0.818	48	1.678	52	1.264	1.410	1.05	1.382	2.66

Table S23. Comparison of bi- and monoexponential fitting of the same fluorescence decay data of **25DOM-HBI-2T** fluorogen in complexes with R52K FAST variant expressed in Hela Kyoto cells as an H2B fusion. $\tau_{1/2}$ is the fluorescence lifetime of the corresponding exponential component in biexponential fitting; A is a relative contribution of the exponential decay component in biexponential fitting; τ_m is an amplitude-weighted average lifetime in biexponential fitting; τ_i is an intensity-weighted average lifetime in biexponential fitting; χ^2 is a value of the Pearson's chi-squared test; τ is a fluorescence lifetime in monoexponential fitting. Each row corresponds to the individual analyzed nucleus.

R52K								
Biexponential fitting							Monoexponential fitting	
τ_1, ns	A_1, %	τ_2, ns	A_2, %	τ_m, ns	τ_i, ns	χ^2	τ, ns	χ^2
1.258	53	2.643	47	1.913	2.163	1.18	1.995	1.61
1.049	46	2.519	54	1.848	2.138	1.07	1.981	1.75
1.223	53	2.627	47	1.882	2.143	1.12	1.971	1.56
1.167	54	2.7	46	1.874	2.186	1.18	1.982	1.81
1.207	55	2.652	45	1.859	2.137	1.13	1.953	1.9
1.242	54	2.672	46	1.895	2.163	1.08	1.981	1.46
1.242	55	2.711	45	1.900	2.181	1.19	1.986	1.6
1.163	55	2.716	45	1.869	2.189	1.16	1.977	1.76
1.254	54	2.701	46	1.921	2.191	1.16	2.007	1.67
1.183	56	2.667	44	1.840	2.135	1.12	1.934	1.91
1.208	55	2.655	45	1.866	2.144	1.15	1.956	1.74
1.236	55	2.647	45	1.874	2.137	1.11	1.956	1.51
1.206	54	2.651	46	1.868	2.145	1.1	1.958	1.67
1.306	58	2.75	42	1.917	2.183	1.12	1.984	1.49
1.251	55	2.686	45	1.895	2.164	1.12	1.973	1.71
1.202	54	2.7	46	1.889	2.184	1.08	1.991	1.71

Table S24. Comparison of bi- and monoexponential fitting of the same fluorescence decay data of **25DOM-HBI-2T** fluorogen in complexes with F62L FAST variant expressed in Hela Kyoto cells as an H2B fusion. $\tau_{1/2}$ is the fluorescence lifetime of the corresponding exponential component in biexponential fitting; A is a relative contribution of the exponential decay component in biexponential fitting; τ_m is an amplitude-weighted average lifetime in biexponential fitting; τ_i is an intensity-weighted average lifetime in biexponential fitting; χ^2 is a value of the Pearson's chi-squared test; τ is a fluorescence lifetime in monoexponential fitting. Each row corresponds to the individual analyzed nucleus.

F62L								
Biexponential fitting							Monoexponential fitting	
τ_1, ns	A_1, %	τ_2, ns	A_2, %	τ_m, ns	τ_i, ns	χ^2	τ, ns	χ^2
0.636	45	2.057	55	1.415	1.768	1.15	1.655	2.34
0.571	44	1.994	56	1.371	1.734	1.19	1.63	2.36
0.706	44	2.077	56	1.470	1.786	1.11	1.672	2.13
0.899	58	2.347	42	1.509	1.848	1.19	1.67	2.63
0.702	43	2.048	57	1.464	1.768	1.19	1.645	2.23
0.74	44	2.057	56	1.481	1.769	1.15	1.643	2.09
0.785	51	2.142	49	1.448	1.766	1.18	1.608	2.75
0.649	42	2.04	58	1.451	1.776	1.15	1.654	2.31
0.633	38	1.965	62	1.453	1.742	1.13	1.66	1.81
0.613	41	2.002	59	1.428	1.756	1.14	1.661	2.3
0.784	46	2.092	54	1.496	1.780	1.18	1.669	2.01
0.706	42	2.023	58	1.469	1.757	1.09	1.653	1.84
0.655	42	2.017	58	1.447	1.759	1.17	1.655	2
0.756	46	2.097	54	1.479	1.781	1.11	1.659	2.04
0.648	41	2.009	59	1.458	1.764	1.15	1.665	1.97
0.557	35	1.934	65	1.451	1.749	1.03	1.675	1.74

Table S25. Comparison of bi- and monoexponential fitting of the same fluorescence decay data of **25DOM-HBI-2T** fluorogen in complexes with P68K FAST variant expressed in Hela Kyoto cells as an H2B fusion. $\tau_{1/2}$ is the fluorescence lifetime of the corresponding exponential component in biexponential fitting; A is a relative contribution of the exponential decay component in biexponential fitting; τ_m is an amplitude-weighted average lifetime in biexponential fitting; τ_i is an intensity-weighted average lifetime in biexponential fitting; χ^2 is a value of the Pearson's chi-squared test; τ is a fluorescence lifetime in monoexponential fitting. Each row corresponds to the individual analyzed nucleus.

P68K								
Biexponential fitting							Monoexponential fitting	
τ_1, ns	A_1, %	τ_2, ns	A_2, %	τ_m, ns	τ_i, ns	χ^2	τ, ns	χ^2
1.284	54	2.259	46	1.732	1.868	1.12	1.786	1.38
1.431	73	2.595	27	1.744	1.897	1.08	1.784	1.35
1.284	47	2.32	43	1.604	1.932	1.16	1.791	1.61
1.329	55	2.235	45	1.733	1.850	1.11	1.779	1.25
1.441	73	2.634	27	1.760	1.918	1.13	1.801	1.52
1.279	55	2.321	45	1.746	1.900	1.16	1.804	1.48
1.383	64	2.431	36	1.757	1.900	1.11	1.8	1.35
1.368	63	2.422	37	1.761	1.909	1.1	1.811	1.34
1.326	60	2.352	40	1.739	1.884	1.12	1.789	1.39
1.03	40	2.11	60	1.682	1.848	1.05	1.769	1.42
1.199	55	2.306	45	1.695	1.874	1.16	1.766	1.52
1.198	56	2.303	44	1.686	1.864	1.12	1.756	1.55
1.251	57	2.31	43	1.710	1.871	1.15	1.773	1.44
1.329	68	2.545	32	1.717	1.904	1.02	1.77	1.49
1.229	55	2.293	45	1.703	1.867	1.12	1.772	1.44
1.166	56	2.319	44	1.676	1.872	1.17	1.756	1.63

Table S26. Comparison of bi- and monoexponential fitting of the same fluorescence decay data of **25DOM-HBI-2T** fluorogen in complexes with P68T FAST variant expressed in Hela Kyoto cells as an H2B fusion. $\tau_{1/2}$ is the fluorescence lifetime of the corresponding exponential component in biexponential fitting; A is a relative contribution of the exponential decay component in biexponential fitting; τ_m is an amplitude-weighted average lifetime in biexponential fitting; τ_i is an intensity-weighted average lifetime in biexponential fitting; χ^2 is a value of the Pearson's chi-squared test; τ is a fluorescence lifetime in monoexponential fitting. Each row corresponds to the individual analyzed nucleus.

P68T								
Biexponential fitting							Monoexponential fitting	
τ_1, ns	A_1, %	τ_2, ns	A_2, %	τ_m, ns	τ_i, ns	χ^2	τ, ns	χ^2
0.986	56	2.018	44	1.440	1.623	1.18	1.535	1.71
0.776	34	1.772	66	1.435	1.590	1.01	1.541	1.43
1.026	57	2.021	43	1.456	1.623	1.17	1.54	1.76
1.025	57	2.034	43	1.461	1.632	1.19	1.546	1.77
0.948	59	2.013	42	1.390	1.588	1.16	1.493	1.72
0.964	58	2.018	42	1.409	1.601	1.11	1.511	1.74
0.962	55	1.977	45	1.417	1.597	1.1	1.513	1.53
0.995	57	2.009	43	1.429	1.605	1.11	1.521	1.63
0.964	58	2.009	42	1.399	1.589	1.16	1.495	1.86
0.955	56	1.973	44	1.404	1.586	1.14	1.5	1.59
0.992	58	2.024	42	1.429	1.611	1.14	1.518	1.63
0.981	58	2.042	42	1.430	1.623	1.13	1.522	1.63
0.924	59	2.031	41	1.379	1.594	1.17	1.488	2.1
0.942	58	1.983	42	1.379	1.570	1.05	1.477	1.65
0.944	55	1.983	45	1.415	1.604	1.14	1.516	1.76
0.944	56	1.976	44	1.402	1.590	1.09	1.501	1.63

Table S27. Fluorescence lifetimes (τ) of **HBR-2,5-DM** fluorogen in complexes with FAST variants expressed in Hela Kyoto cells as an H2B, vimentin, IMS (intermembrane space of mitochondria) or β4Gal-T1 fusions. Monoexponential fitting model. *n* is a number of individual analyzed cells. SD is standard deviation.

FAST variant	H2B,<br>τ ± SD, ns	Vimentin,<br>τ ± SD, ns	IMS,<br>τ ± SD, ns	β4Gal-T1,<br>τ ± SD, ns
R52K	1.374 ± 0.007<br>*n* = 22	1.316 ± 0.019<br>*n* = 24	1.306 ± 0.015<br>*n* = 24	1.290 ± 0.020<br>*n* = 23
F62L	2.200 ± 0.033<br>*n* = 23	2.195 ± 0.026<br>*n* = 23	2.170 ± 0.020<br>*n* = 25	2.237 ± 0.036<br>*n* = 24
P68K	1.912 ± 0.022<br>*n* = 21	1.879 ± 0.012<br>*n* = 21	1.873 ± 0.023<br>*n* = 23	1.806 ± 0.019<br>*n* = 20
P68T	1.665 ± 0.010<br>*n* = 24	1.676 ± 0.025<br>*n* = 27	1.656 ± 0.040<br>*n* = 22	1.587 ± 0.031<br>*n* = 21

References

[1] C. Chen, S. R. Tachibana, N. S. Baleeva, I. N. Myasnyanko, A. M. Bogdanov, A. S. Gavrikov, A. S. Mishin, K. K. Malyshevskaya, M. S. Baranov, C. Fang, *Chem. Eur. J.,* **2021**, 27, 8946.

[2] M.V. Goncharuk, N.S. Baleeva, D.E. Nolde, A.S. Gavrikov, A.V. Mishin, A.S. Mishin, A.Y. Sosorev, A.S. Arseniev, S.A. Goncharuk, V.I. Borshchevskiy, R.G. Efremov, .K.S. Mineev, M.S. Baranov. *Commun. Biol*., **2022**, 5, 706.

[3] C. Li, M. A. Plamont, H. L. Sladitschek, V. Rodrigues, I. Aujard, P. Neveu, T. Saux, L. Jullien, A. Gautier, *Chem. Sci*., **2017**, 8, 5598

[4] K.S. Mineev, S.A. Goncharuk, M.V. Goncharuk, N.V. Povarova, A.I. Sokolov, N.S. Baleeva, A.Y. Smirnov, I.N. Myasnyanko, D.A. Ruchkin, S. Bukhdruker, A. Remeeva, A. Mishin, V. Borshchevskiy, V. Gordeliy, A.S. Arseniev, D.A. Gorbachev, A.S. Gavrikov, A.S. Mishin, M.S. Baranov. *Chem Sci.*, **2021**, 12(19), 6719.
