# Supplementary material for: Fluorescence lifetime multiplexing with fluorogen activating protein FAST variants
Source: Commun Biol. 2024 Jul 2;7:799. doi: 10.1038/s42003-024-06501-1 (PMC11219735; doi:10.1038/s42003-024-06501-1)
Supplement: Supplementary file 2 — Description of Additional Supplementary Materials [file 42003_2024_6501_MOESM2_ESM.docx]

**Description of Additional Supplementary Files**

**File name:** Supplementary Data 1

**Description:** Source data for all figures form main text and SI
